# A high-quality haplotype genome of *Michelia alba* DC reveals differences in methylation patterns and flower characteristics

**DOI:** 10.1186/s43897-024-00098-z

**Published:** 2024-05-29

**Authors:** Sirong Jiang, Meiling Zou, Chenji Zhang, wanfeng Ma, Chengcai Xia, Zixuan Li, Long Zhao, Qi Liu, Fen Yu, Dongyi Huang, Zhiqiang Xia

**Affiliations:** 1 Sanya Nanfan Research Institute of Hainan University, Hainan Yazhou Bay Seed Laboratory, Sanya, China; 2 College of Tropical Crops, Hainan University, Haikou, China; 3 China Agricultural University, Beijing, China; 4 Qinghai University, Xining, China

**Keywords:** *Michelia* L, Haplotype genome assembly, DNA methylation, Allele genes, Flower

## Abstract

*Michelia alba* DC is a highly valuable ornamental plant of the Magnoliaceae family. This evergreen tropical tree commonly grows in Southeast Asia and is adored for its delightful fragrance. Our study assembled the *M. alba* haplotype genome MC and MM by utilizing Nanopore ultralong reads, Pacbio Hifi long reads and parental second-generation data. Moreover, the first methylation map of Magnoliaceae was constructed based on the methylation site data obtained using Nanopore data. Metabolomic datasets were generated from the flowers of three different species to assess variations in pigment and volatile compound accumulation. Finally, transcriptome data were generated to link genomic, methylation, and morphological patterns to reveal the reasons underlying the differences between *M. alba* and its parental lines in petal color, flower shape, and fragrance. We found that the AP1 and AP2 genes are crucial in *M. alba* petal formation, while the 4CL, PAL, and C4H genes control petal color. The data generated in this study serve as a foundation for future physiological and biochemical research on *M. alba*, facilitate the targeted improvement of *M. alba* varieties, and offer a theoretical basis for molecular research on *Michelia* L.

**Supplementary Information:**

The online version contains supplementary material available at 10.1186/s43897-024-00098-z.

## Core

Our study assembled the *Michelia alba* haplotype genome MC and MM by utilizing Nanopore reads, Pacbio Hifi reads and parental second-generation data. Moreover, the first methylation map of Magnoliaceae was constructed based on the methylation site data obtained using Nanopore data. The transcriptome and metabolome data were also generated, which were integrated with genomic, methylation, and morphological patterns to elucidate the underlying mechanisms behind the variations in petal color, flower shape, and fragrance between Michelia leucoides and its parent lines.

## Gene and accession numbers

All the raw sequencing data generated during this study have been deposited at the National Genomics Data Center as a BioProject under accession PRJCA008087. Transcriptome sequence reads have been deposited in the GSA database under BioProject numbers CRA006224, CRA006225, and CRA006110. The genome assemblies and annotation files are available at the website https://ngdc.cncb.ac.cn/.

## Introduction

The family Magnoliaceae Juss is distributed in north temperate to tropical regions. This family is considered one of the earliest lineages of flowering plants (Nie et al. 2008). Linnaeus (Linnaeus 1753) defined *Michelia* L. for the first time using *Michelia champaca* Linn, which has axillary flowers, as the type species. Notably, in Magnoliaceae, except for the Magnolia genus, which has multiple ploidy types, all other genera are diploid (Janaki Ammal EK. 1952). Chen summarized the research on the number of chromosomes in Magnoliaceae species (Chen et al. 1998). All 11 genera and 61 species grown in China (including 29 species and 3 varieties of *Michelia* L.) have a haploid chromosome number x = 19. The *Parakmeria* and *Magnolia* genera have multiple ploidy types, while the other genera are all diploid. Interspecific hybridization of *Michelia* L. frequently occurs so that high morphological variation and high genome heterozygosity are prevalent.

*Michelia alba* is an evergreen tree of the Magnoliaceae family that is widely distributed in subtropical and tropical regions. The flowers have white petals and a strong fragrance. It has a long flowering period, and its leaves are dark green. It is a popular garden ornamental tree species, mostly planted as a tree growing on the side of the streets. The flowers can be used for essence extraction (Songsamoe et al. 2021) or for tea infusions and to make extracts for medicinal purposes. *M. alba* is commonly considered a hybrid between *M. champaca* and *M. montana*. It is native to Java, Indonesia and is now widely cultivated in Southeast Asia (Nooteboom 1985). It is commonly found in Fujian, Guangdong, Guangxi, Yunnan, and other provinces in China. Many potted plants are grown in various provinces and regions in the Yangtze River Basin. *M. alba* is not cold tolerant and generally overwinters in a greenhouse. *Michelia champaca*, *Michelia figo*, *Michelia macclurei*, etc., are used as *M. alba* rootstocks for grafting and breeding.

The *M. alba* genome is highly heterozygous and allodiploid. Due to technological limitations, few *M. alba* genome characterization studies have been conducted. However, with the rapid development of genome sequencing technology, the assembly of allodiploid genomes has become feasible. Previously, *M. alba* chloroplast genome assembly (Hinsinger and Strijk 2017) showed that *M. alba* is closely related to *M. odora*, with the genus *Michelia* being a subgenus of *Magnolia.* However, it did not resolve *M. alba's* evolutionary position in the plant kingdom. As this study characterized, for the first time, the genome of *Michelia* L., it will provide insights for the classification of Magnolia species and a data basis for the study of evergreen woody plants. In addition, research on *M. alba* metabolites (Xia et al. 2010) has been quite comprehensive. Thus, genomic data can greatly accelerate research on *M. alba* and the utilization of this species.

In plants, DNA methyltransferases are responsible for extensive methylation of plant genomes, primarily at cytosine bases (Lucibelli et al. 2022). Cytosine DNA methylation (5mCs) in plants can occur in three different sequence contexts: CpG, CHG, and CHH (where H = A, C, or T). These different sequence contexts have distinct roles in regulating various biological processes. Various DNA methylation detection methods have been developed for different experimental purposes. These methods include bisulfite conversion sequencing (BS-Seq), MeDIP-Seq, RRBS-Seq, WGBS, MBD-Seq, SMRT, etc., and newer methods such as Nanopore sequencing. DNA modifications can be detected as changes in the Oxford Nanopore Technologies (ONT) MinION's ionic current signal (Ni et al. 2021). The electrical signal of Nanopore sequencing is more sensitive to nucleotide base modifications. Several algorithms have been utilized to identify methylation sites by Nanopore sequencing, and its utilization makes it an important tool for DNA methylation identification.

In this study, to understand the methylation pattern of the *M. alba* genome at the haplotype level, the methylation sites in the DNA from the flowers and leaves of the two parents were determined using *M. alba* Nanopore sequencing data. Methylation models of *M. champaca* and *M. montana* were constructed. The mechanisms underlying the evolution of important traits such as flower color, shape, and floral fragrance in *M. champaca* and *M. montana* were analyzed by joint analysis with differential allelic expression. Our results provide insights and a wealth of data for the improvement of *Michelia* L.

## Results

### Assembly and annotation of a high-quality, chromosome-scale *M. alba* haplotype genome

Based on the FISH (Fluorescence In Situ Hybridization) results using blue and green light comparison, it was observed that the chromosomes of *Michelia champaca* and *M. alba* were fully covered (Figure. S1). This indicates the presence of a close kinship between them. The complete coverage of chromosomes suggests a similarity in the DNA sequence composition and structure between *Michelia champaca* and *M. alba*.

**Fig.1:**
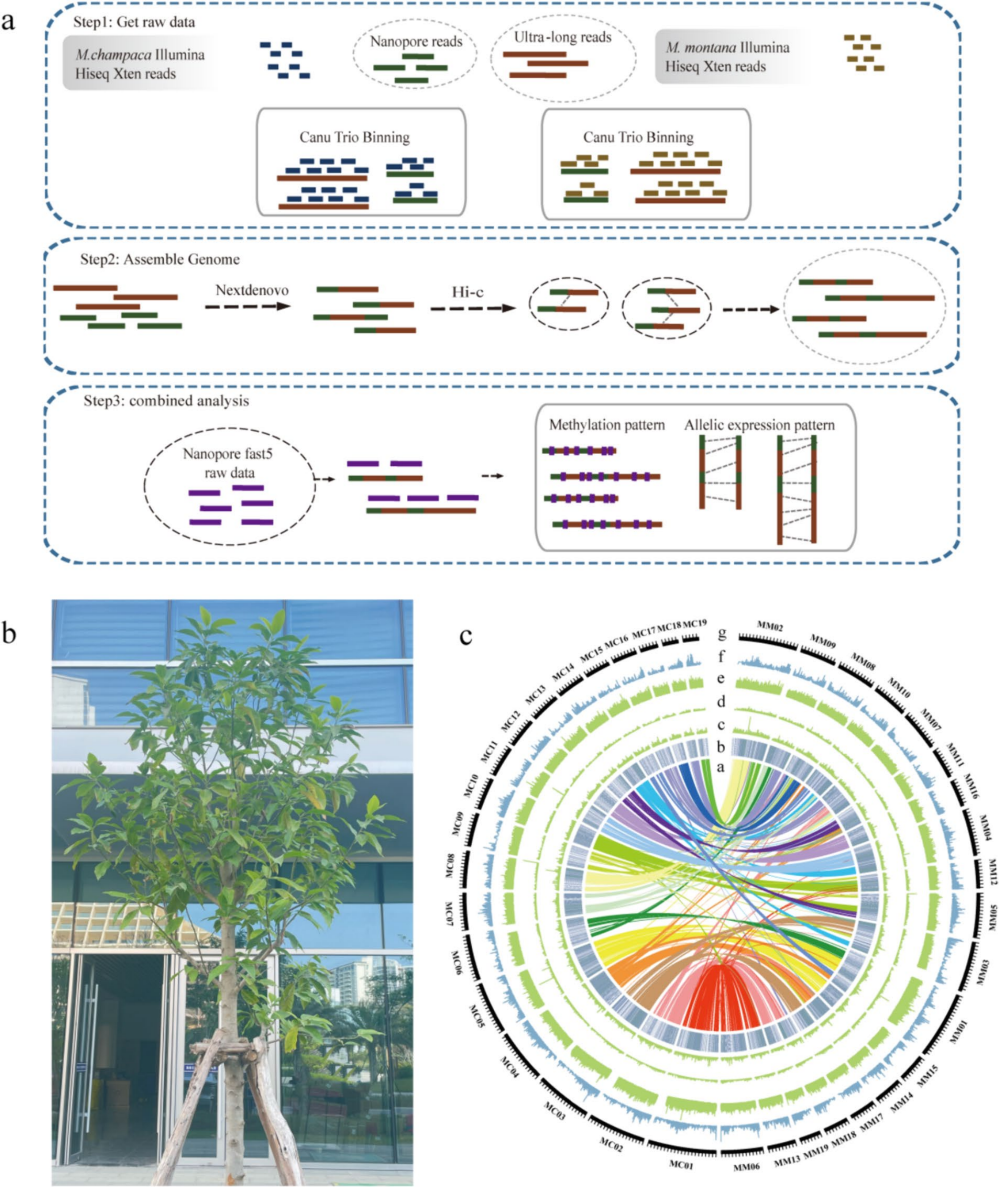
Overview of the *Michelia alba* haplotype genome assembly and features. **a**. Assembly flow chart. **b**. A mature *Michelia alba* tree (**c**). a. Collinearity between subgenomes. b. Gene density. c-e. Distribution of three repeat types in the genome: DNA transposons, LINEs, and LTRs. f. Flower RNA-seq expression

The *M. alba* genome was sequenced using the Illumina HiSeq Xten sequencing platform. K-mer analysis indicated that *M. alba* has a large genome of approximately 1.8 Gb with 59.1% repetitive elements and is highly heterozygous (4.76%) (Figure. S2). At the same time, the parents were sequenced using the Illumina HiSeq Xten sequencing platform. *M. champaca* has a large genome of approximately 2.24 Gb but a degree of low heterozygosity (0.38%) (Figure. S3). *M. montana* has a large genome of approximately 1.47 Gb and has low heterozygosity (0.95%) (Figure. S4). We used the parental Illumina reads to bin the 261 Gb Nanopore reads of the hybrid based on parental origin. A total of 112 G Nanopore reads were assigned to *M. champaca*, and 130 G Nanopore reads were assigned to *M. montana* for subsequent haplotype genome assembly (Table S1). A total of 61 G Pacbio Hifi reads were assigned to *M. champaca*, and 86 G Pacbio Hifi reads were assigned to *M. montana* for subsequent haplotype genome assembly. The assembled original genome sizes were 2.55 Gb MC (*M. champaca*) and 2.42 Gb MM (*M. montana*). respectively. Deduplication was performed by Purge_dups, The obtained genome sizes were 2.23 and 2.19, respectively. Then the raw sequencing data of Pacbio were used for Polish. Using minimap2 + racon strategy to complete three rounds of polish process. The final genome size is 2.19 and 2.13. The contigs and scaffolds of the MC (*M. champaca*) and MM (*M. montana*) subgenomes were further scaffolded into 19 chromosomes by Hi-C technology, and the anchored genomes were 2.03 Gb (97.19%) and 2.06 Gb (97.96%), respectively (Figures. S5, S6, Fig. 1a, Table 1). The second-generation genome data of *M. alba* were mapped to the haplotype genomes MC and MM, with mapping rates of 99.19% and 98.32%, respectively. As a reference, we mapped the second-generation sequencing data of *Litchi chinensis*, *Zea mays*, *Magnolia biondii,* and *Liriodendron chinense* against the *M. alba* genome, and the mapping rates were 5.57%, 11.61%, 86.20% and 51.98%, respectively. This indicated a high similarity between *M. alba* and the MC and MM haplotype genomes. Both assemblies are contiguous (mean Contig N50: 12.425Mb, Scaffold N50:116.01 Mb) and complete (mean BUSCO completeness: 95.5%) (Table S2, Fig. 1c). The corresponding second-generation genome data were compared with the genome data via the Burrows‒Wheeler Aligner (BWA). The mapping rate of the haplotype genome and the Illumina data was 95.68% (MC) and 96.72% (MM). Merqury was used to assess the consensus quality value (QV) and the k-mer completeness of the *M. alba* genome assembly, which were 34.03 and 96.22%, respectively. These results suggested that the assembled *M. alba* genome had high completeness and accuracy.

**Table 1:** Major indicators of the MC and MM subgenome

Assembly feature Statistic	MC	MM
Contig N50 (Mb)	15.08	9.77
Scaffold N50 (Mb)	113.08	118.94
Longest scaffold (Mb)	208.22	207.91
Assembled genome size (Mb)	2,100.886	2,156.539
GC_content (%)	40.33	40.22
Assembly of genome (%)	97.19	97.96
Repeat region of assembly (%)	74.02	71.73
Average coding sequence length (bp)	1,782.14	1407.95
Average exons per gene length (bp)	152.9	148.5
Gene number	64,070	68,392

We used the MAKER pipeline and evidence-based and ab initio gene predictions to predict 64,070 protein-coding genes in the MC subgenome assembly and 68,392 protein-coding genes in the MM subgenome assembly. The mean values of gene length, number of exons, and coding sequence length in MC were 1,782.14 bp, 4.6, and 152.929 bp, respectively, and in MM, they were 1,40.95 bp, 4.3, and 148.526 bp, respectively. We also identified 115 and 108 microRNAs (miRNAs), 444 and 492 transfer RNAs (tRNAs), 2,367 and 2,098 small nuclear RNAs (snRNAs), and 174 and 330 ribosomal RNAs (rRNAs) in the subgenomes, respectively. The protein-coding genes in MC and MM had an average gene length of 1,782 bp and 1408 bp, and the average coding DNA sequence (CDS) length was 153 bp and 149 bp, respectively.

The repetitive element percentages in the MC and MM subgenomes were over 74.02% and 71.73%, respectively, comparable to that of *Magnolia biondii* (Dong et al. 2021) (66.48%) and higher than that of *Chimonanthus praecox *(Shang et al. 2020a) (~ 45.73%). The percentage of various types of repeats was similar in the haplotype genomes, with the most abundant being Long terminal repeats (LTRs) (34.48% and 30.32%, respectively), followed by long interspersed nuclear elements (LINEs) (0.86% and 1.23%, respectively). The least abundant DNA transposons (0.91% and 0.79%, respectively). (Table S3). Furthermore, using the iTAK tool, we discovered 828 and 859 transcription factors in the MC and MM subgenomes, respectively. (Table S4).

Based on the subgenome, we compared the gene expression differences and structural variations between the two parents and their offspring. The results showed that in the three tissues of petals, stamens and leaves, the number of differential genes in *M. champaca* and *M. alba* were 2,702, 1,250 and 2,094, respectively. The number of differentially expressed genes in *M. montana* and *M. alba* were 3,322, 2,674, 2,454, respectively. By comparing the second-generation sequencing data of *M. alba* with the two subgenomes, it can be found that 1,251 insertions and 13,601 deletions were identified between *M. champaca* and *M. alba*, while 731 insertions and 13,681 deletions were identified between *M. montana* and *M. alba*. It can be seen that the information from the two subgenomes is not significantly different.

### Genome evolution and whole-genome duplication

**Fig.2:**
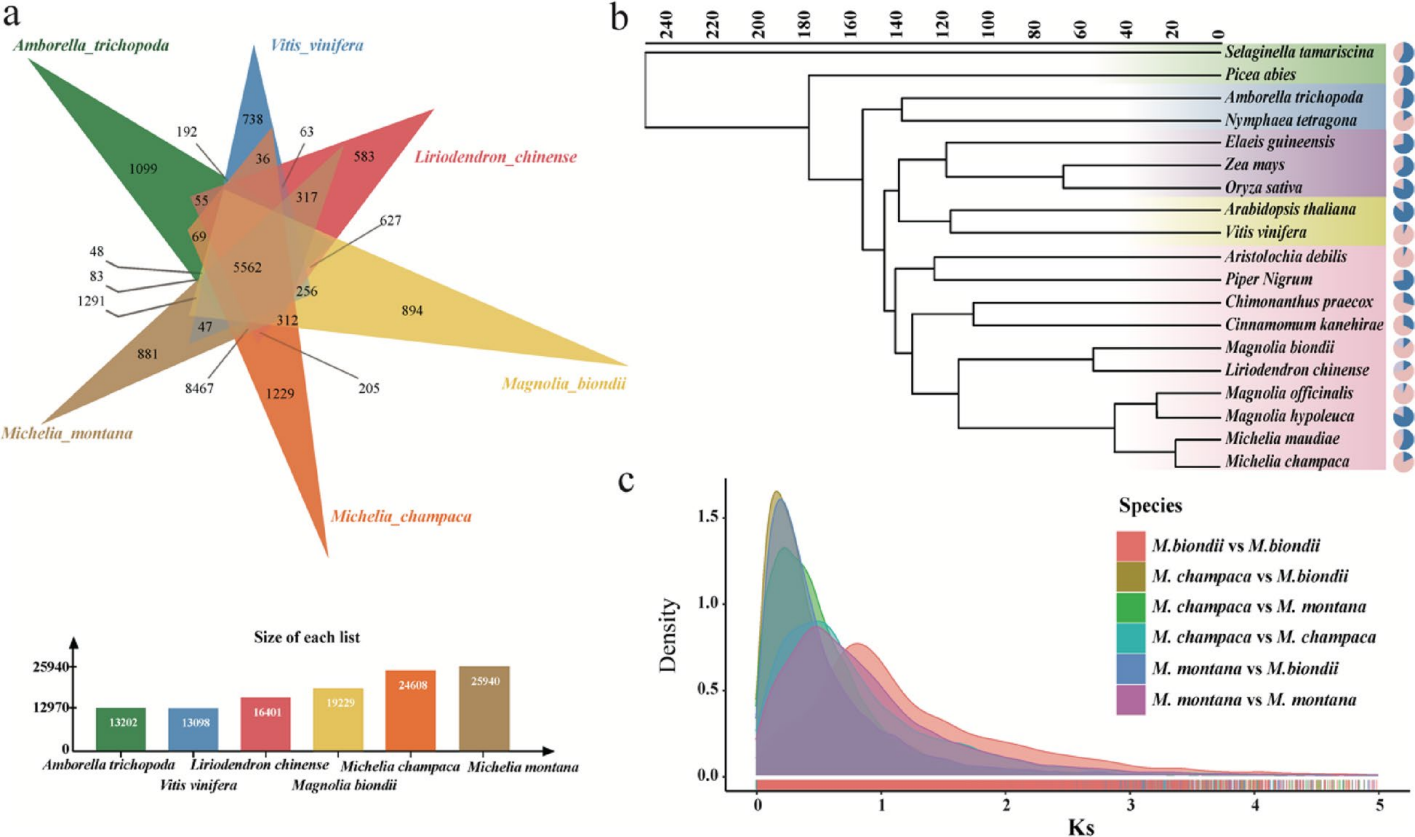
**a**. Gene family analysis in six species (*M. champaca, M. montana, A. trichopoda, V. vinifera, L. chinense* and *M. biondii*). **b**. A phylogenetic tree of 19 species constructed using single-copy orthologous genes. **c**. Frequency distributions of synonymous substitution rates (Ks) between homologous gene pairs in syntenic blocks of *M. champaca*-*M. champaca*, *M. montana*-*M. montana*, *M. bindii-M. biondii*, *M. champaca*-*M. biondii*, *M. montana*-*M. biondii*, and *M. champaca*-*M. montana*

The gene families of 6 species, including *M. champaca* and *M. montana,* were analyzed. The other four species included were the ancient species *Amborella trichopoda *(Resource and (TAIR): improved gene annotation and new tools. 2011), *Vitis vinifera *(Liang et al. 2019)*, Liriodendron chinense *(Chen et al. 2019), and *Magnolia biondii*. All protein-coding genes from the 6 genomes were clustered into 33,676 gene families (two or more members), of which 5,562 were common to all groups. A total of 1,229 and 881 gene families were found to be specific to *M. champaca* and *M. montana*. (Fig. 2a).

We assessed the functional enrichment of specific gene families in the two subgenomes to understand the functional direction of the two subgenomes during speciation. KEGG (Kyoto Encyclopedia of Genes and Genomes) analysis of the MC-specific gene families revealed marked enrichment in genes involved in the biosynthesis of secondary metabolites, starch and sucrose metabolism, glutathione metabolism, biosynthesis of amino acids, fatty acid metabolism and MAPK signaling pathway—plant pathways. On the other hand, MM-specific gene families exhibited a marked enrichment in genes involved in metabolic pathways, carbon metabolism, protein processing in the endoplasmic reticulum, amino sugar and nucleotide sugar metabolism, and glycerophospholipid metabolism (Table S6, Figure. S7).

The position of Magnoliaceae in plant evolution has been extensively studied, but there have been no accurate conclusions. We selected 2 dicot plant species (*Vitis vinifera, Arabidopsis thaliana*), 3 monocot plant species *Elaeis guineensis *(Yang et al. 2023), *Zea mays *(Gui et al. 2020), *Oryza sativa *(Sakai et al. 2013), 10 Magnoliids species (*M. montana, M. champaca, Magnolia biondii*, *Chimonanthus praecox *(Shang et al. 2020b), *Cinnamomum kanehirae *(Chaw et al. 2019a), *Amborella trichopoda *(Qin et al. 2021), *L. chinense*, *Piper nigrum *(Hu et al. 2019), *Magnolia hypoleuca *(Zhou et al. 2023) and *Magnolia officinalis *(Yin et al. 2021), an early angiosperm species (*Nymphaea tetragona*), an ancient Pteridophyta species (*Selaginella tamariscina*), and an ancient gymnosperm (*Picea abies*) for homologous gene analysis to more accurately depict the phylogenetic relationships. After thorough evaluation and selection, we utilized single-copy gene familys to construct the phylogenetic tree. Our findings indicated that monocots and dicots were co-located on one branch, while Magnoliids were in a separate branch of the phylogenetic tree. Furthermore, the phylogenetic tree revealed that *M. montana* and *M. champaca* share a close genetic relationship (Table S7, Fig. 2b). In addition, we selected low-copy homologous genes of these 19 species to construct a phylogenetic tree. We reached the same conclusion as the low-copy homologous genes (Figure. S8).

According to our research, the divergence between *M. montana* and *M. champaca* occurred approximately 18 million years ago. Additionally, the split between *L. chinense* and *M. montana* and *M. champaca* occurred approximately 122 million years ago. Our investigation also delved into genome evolution, specifically examining whole-genome duplication (WGD) events. We determined the synonymous substitutions per synonymous site (Ks) for various pairs of *Magnolia* species, including *M. champaca* vs *M. champaca*, *M. montana* vs *M. montana*, and *M. biondii* vs *M. biondii*, as well as comparisons between species such as *M. champaca* vs *M. biondii*, *M. montana* vs *M. biondii*, and *M. champaca* vs *M. montana*. By analyzing the distribution of Ks values, we identified peaks corresponding to genome-wide duplication events. Furthermore, we observed that the Ks distributions of *M. champaca* and *M. montana* subgenome paralogues indicated a single WGD event.

### Different tissue methylation patterns of the MC and MM subgenomes

**Fig.3:**
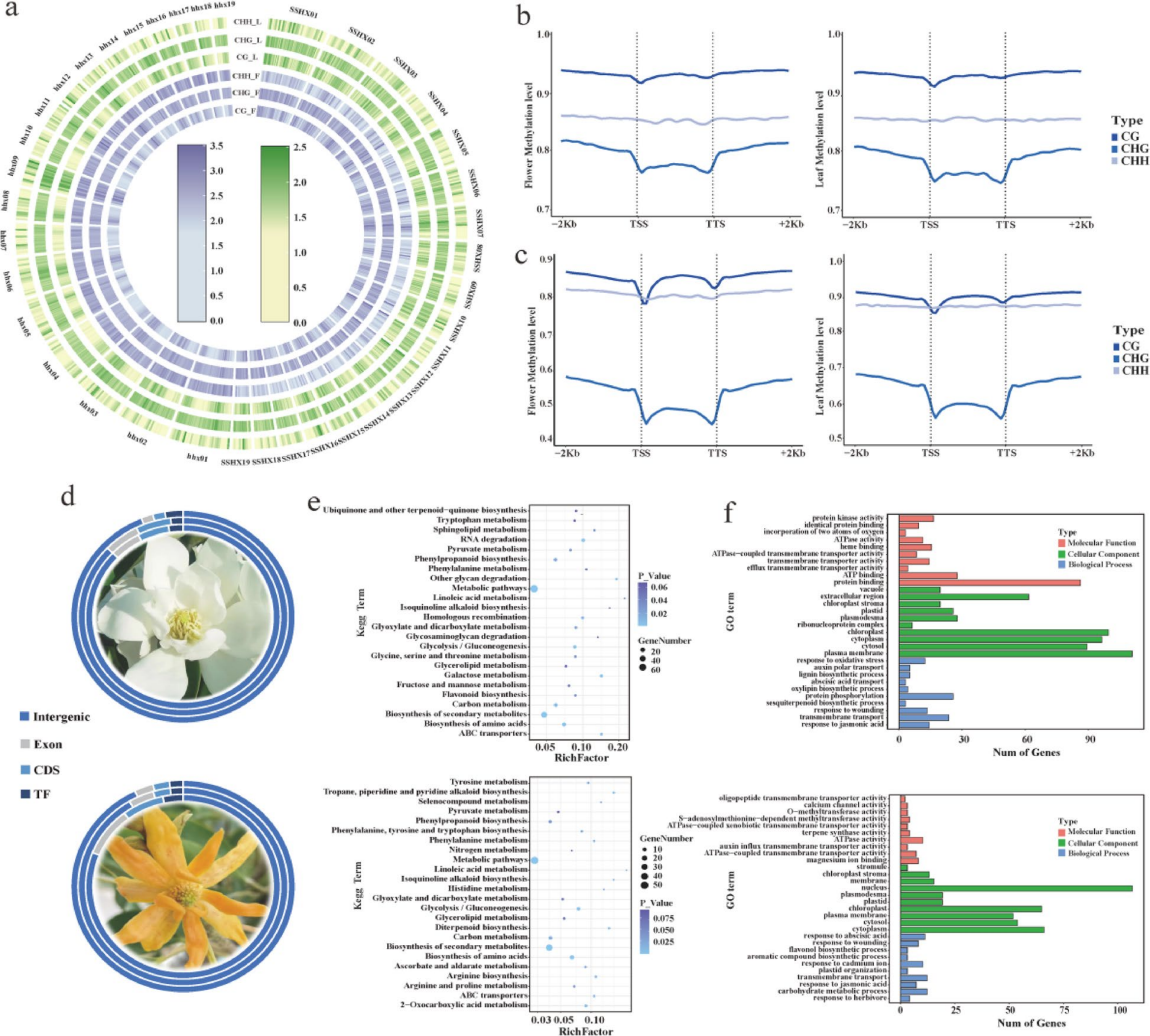
Methylation Pattern Analysis in *M. champaca* and *M. montana*. **a**. Genome distribution of the three methylation patterns in the flowers and leaves of *M. champaca* and *M. montana*. **b**. *M. montana*. methylation levels in the gene bodies and the 2 kb upstream from TSS and 2 kb downstream from the TTS flanking regions. **c**. *M. champaca* methylation levels in the gene bodies and the 1 kb upstream from TSS and 1 kb downstream from TTS flanking regions. **d**. Distribution of the three types of differentially methylated regions in the genome, CHH, CHG, and CG, inward to outwards: CHH, CHG, and CG, respectively. **e**. From top to bottom in this figure, KEGG enrichment analysis of differentially methylated regions in the flowers and leaves of *M. montana* and *M. champaca*, respectively. **f**. From top to bottom, GO enrichment analysis of differentially methylated regions in the flowers and leaves of *M. montana* and *M. champaca*, respectively

To investigate the genome-wide cytosine methylation of the two *M. alba* haplotypes in various tissues, we analyzed the methylation sites in DNA from both the flowers and leaves of *M. champaca* and *M. montana* (as depicted in Fig. 3a). To gain insights into the DNA methylation patterns across different genomic regions, we examined the methylation levels in the gene body (from the transcription start point (TSS) to the transcription termination point (TTS)), as well as the flanking regions 2 kb upstream of the TSS and downstream of the TTS (Fig. 3b and c).

The methylation level of *M. champaca* flowers and leaves was the highest in the GC sequences, followed by CHH and CHG. Notably, there were few differences in the methylation levels of flowers and leaves. The highest CG methylation levels in flowers and leaves were observed in *M. montana*, and methylation levels were generally higher in the leaves than in the flowers. The CHH, CG, and CHG methylation sites detected in *M. montana* flowers were 321,211,379, 317,284,791, and 257,778,276, respectively. The CHH, CG, and CHG methylation sites detected in leaves were 302,713,120, 271,316,538, and 162,325,854, respectively. The CHH, CG, and CHG methylation sites detected in *M. champaca* flowers were 53,156,789, 52,479,090, and 51,798,809, respectively. The CHH, CG, and CHG methylation sites detected in leaves were 56,094,290, 55,316,685, and 54,198,256, respectively (Table S8). The methylation levels in all three sequence motifs were higher in the leaves of *M. montana* than in the flowers. In contrast, the opposite was observed in *M. champaca*, with higher methylation in the flowers.

The regions with significant differences in methylations were studied to further understand *M. montana* and *M. champaca* methylation patterns in flowers and leaves. *M. montana* had 47,381 differentially methylated regions, while *M. champaca* had 29,907 differentially methylated regions. Moreover, the number of differentially methylated annotated genes was 531 and 343 (Table S9). Thus, *M. montana* had significantly more differentially methylated genes than *M. champaca*.

KEGG enrichment analysis of these differentially methylated regions (DMRs) revealed that the *M. champaca* DMRs were mainly enriched in the biosynthesis of secondary metabolites, biosynthesis of amino acids, and diterpenoid biosynthesis pathways. *M. montana* DMRs were mainly enriched in the RNA degradation and biosynthesis of amino acid pathways. GO (Gene Ontology) enrichment analysis revealed that *M. champaca* DMRs were mainly enriched in the carbohydrate metabolic process, response to jasmonic acid, and flavonol biosynthetic process ontologies. *M. montana* DMRs were mainly enriched in response to wounding, sesquiterpenoid biosynthetic process, and protein phosphorylation ontologies. (Fig. 3e and f, Table S10). Differentially methylated regions were distributed more frequently in the intergenic and promoter regions across the whole genome (Fig. 3d, Table S11).

### Integrative analysis of allelic expression and methylation levels reveals the evolution of flower color and flower shape in *M. alba*

In this work, we sampled *M. champaca* and *M. montana* tissues with yellow and white flower petal colors to elucidate interspecies differences in petal pigments. Using liquid chromatography–electrospray ionization tandem mass spectrometry (LC–ESI–MS/MS), 21 anthocyanin compounds were identified and quantified (against 99 anthocyanin standards) in petals, including 12 delphinidins, 7 pelargonidins, and 2 cyanidins. Eighteen differentially abundant metabolic compounds belonging to anthocyanins were identified between *M. champaca* and *M. montana*. Specifically, pelargonidin-3-O-glucoside, pelargonidin 3-sophoroside 5-glucoside, cyanidin 3-xyloside, cyanidin 3-sophoroside-5-glucoside, cyanidin 3-sambubioside, cyanidin 3-glucogalactoside, cyanidin 3-(6' '-succinyl-glucoside), and cyanidin 3-(6' '-malonylglucoside)-5-glucoside were found to be more abundant in *M. champaca* than in *M. montana*. One carotenoid compound was also identified but had no significantly different abundance between *M. champaca* and *M. montana*. Therefore, anthocyanins represent the most significant differentially abundant metabolites between *M. champaca* and *M. montana*. Among these, delphinidins were predominant in *M. champaca*, with pelargonidin-3-O-glucoside exhibiting the highest concentration (Figure. S9).

**Fig. 4:**
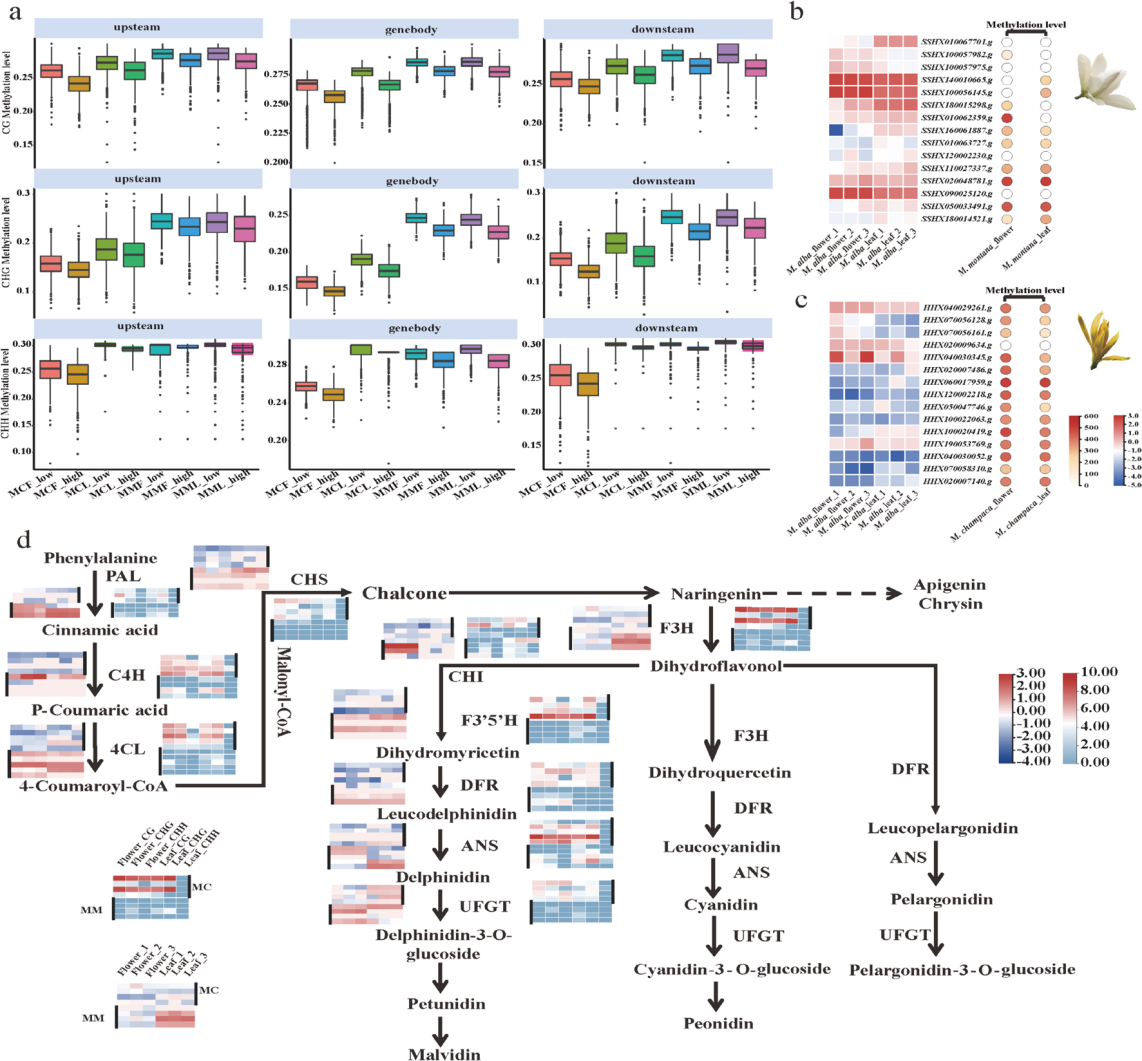
Combined allelic gene expression and methylation analysis. **a**. Allelic expression differences in relation to methylation levels in three different gene locations: the gene body region, upstream region, and downstream region. MCF_low corresponds to low expression levels in *M. champaca* flowers. MCF_high corresponds to high expression levels in *M. champaca* flowers. MCL_low corresponds to low expression levels in *M. champaca* leaves. MCL_high corresponds to high expression levels in *M. champaca* leaves. MMF_low corresponds to low expression levels in *M. montana* flowers. MMF_high corresponds to high expression levels in *M. montana* flowers. MML_low corresponds to low expression levels in *M. montana* leaves. MML_high corresponds to high expression levels in *M. montana* leaves. **b**. Heatmap of *M. montana*-related gene expression (left) and methylation levels (right) of MM homologous gene pairs in the flavonoid metabolic pathway. **c**. Heatmap of *M. montana*-related gene expression (left) and methylation levels (right) of MC homologous gene pairs in the flavonoid metabolic pathways. **d**. Gene expression and methylated sites of genes in the flavonoid metabolism pathway. The heatmap on the left illustrates the gene expression (from left to right are three replicates in flowers and leaves), and the heatmap on the right illustrates the methylation sites (from left to right are CG, CHG, and CHH methylation sites in flowers and leaves)

Haplotype-based genomic analyses have been frequently employed to understand the relationship between allelic expression and methylation. Based on synteny and annotation, 22,034 pairs were considered reliable allelic genes. Allelic pairs with differential expression in the haplotype genomes were identified using RNA-seq data from two tissues (flower and leaf) of *M. champaca* and *M. montana*. A total of 8,099 gene pairs were expressed in the flowers (36.76% of alleles), and 7,095 gene pairs were expressed in the leaves (32.2% of alleles). When differences were identified in the expression between alleles (Table S12), these were considered differentially expressed (Fig. 4a). Statistical analysis of the methylation levels among differentially expressed alleles showed that higher gene expression correlated with a lower methylation level (Sinha et al. 2020) (Fig. 4b), and this relationship was apparent in all tissues (Fig. 4c). It was further verified that the presence of methylated sites inhibits gene expression.

Anthocyanins are pigments that are soluble in water and are frequently and abundantly present in plants. Anthocyanins are responsible for most of the coloring in plant petals. For instance, *M. champaca* flowers are yellow, *M. montana* flowers are white, and the flowers of their hybrid offspring, *M. alba*, are white. In the context of the Magnoliaceae family, it has been discovered that changes in flower color are linked to the abundance of anthocyanins (Liu et al. 2020; Lang et al. 2019). Based on our findings, we compared the methylation levels of anthocyanin synthesis-related genes in *M. alba* haplotypes. We discovered that *M. champaca* had higher methylation levels in these genes than *M. montana*. Additionally, these genes were expressed at lower levels in *M. alba* flowers and leaves. For example, the homologous genes *SSHX090025120.g* (MM) and *HHX040030052.g* (MC) of the key gene *4CL* in the flavonoid metabolism pathway, the homologous genes *SSHX140010665.g* and *HHX020009634.g* encoding *PAL*, the homologous genes *SSHX100056145.g* and *HHX040030345.g* encoding *C4H*, and the *SSHX090025120.g*, *SSHX140010665.g*, *SSHX100056145.g* had a much higher expression in flowers and leaves than *HHX040030052.g*, *HHX020009634.g,* and *HHX040030345.g*. However, the methylation levels of these genes in *M. montana* flowers and leaves were much lower than those in *M. champaca* (Fig. 4d). From the evidence presented, it is reasonable to speculate that differential methylation plays a role in regulating the expression of flavonoid synthesis genes. This regulation leads to a lower expression level, resulting in the absence of the yellow color observed in the *M. montana* parental line. This finding serves as a theoretical foundation for further exploration into the mechanisms underlying flower coloration in *M. alba*.

**Fig.5:**
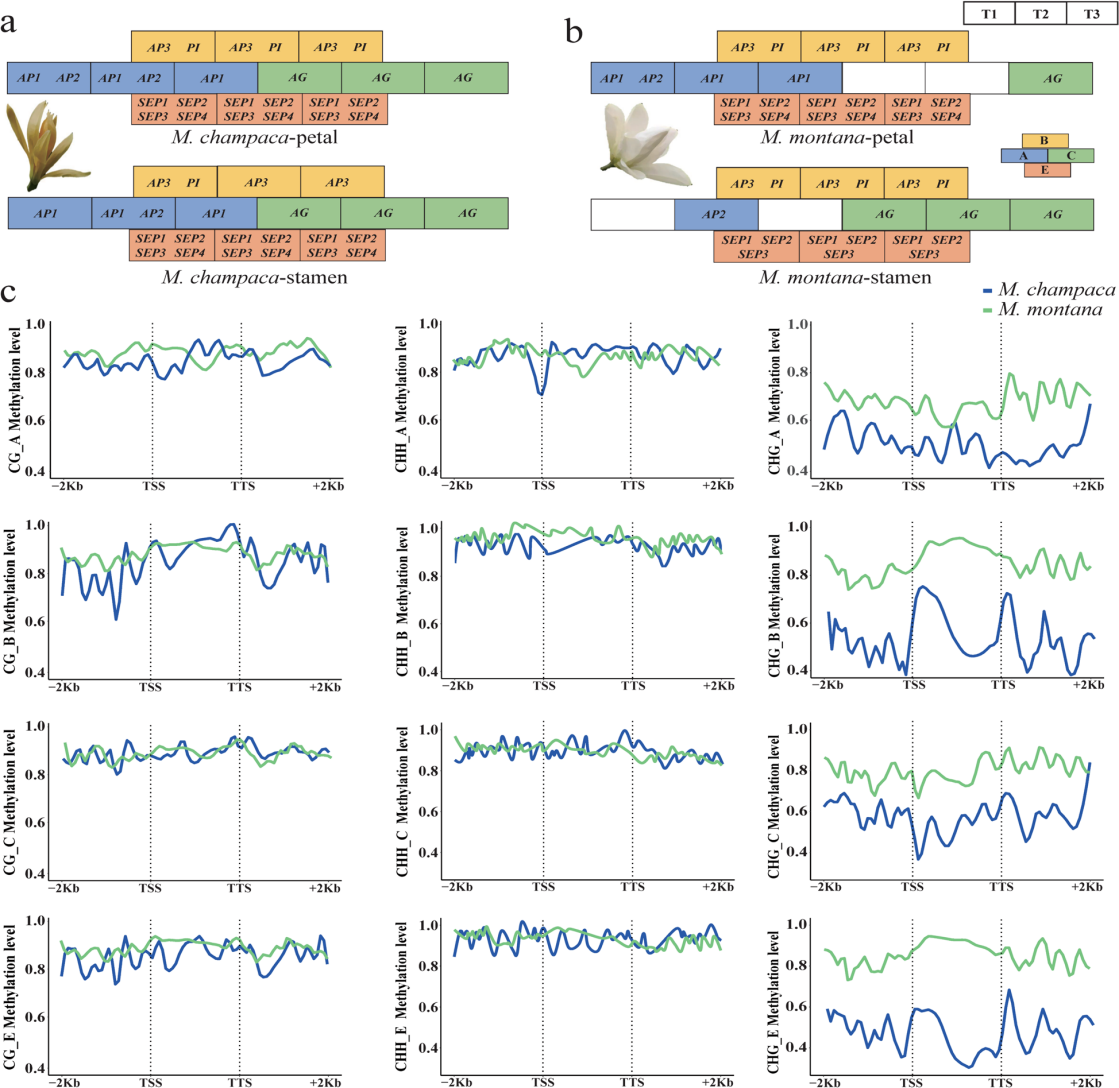
ABCE model analysis. **a**. Expression patterns of *M. champaca* ABCE genes in three developmental stages of petals and stamens. **b**. Expression patterns of *M. montana* ABCE genes in three developmental stages of petals and stamens. **c**. Methylation levels of *M. champaca* and *M. montana* ABCE genes (from top to bottom are A, B, C, and E genes, respectively)

In addition to their color, the flowers of *M. montana* and *M. champaca* differ significantly in appearance. *M. montana* typically has 9 obovate petals, while *M. champaca* has 15–20 hard petals with an oblanceolate shape. It is worth noting that the ABCE model is a well-established framework for flower pattern regulation. Four functional genes that are part of the ABCE model were identified in the genome. They were *AP1* and *AP2 in* A, *AP3* and *PI* in B, *AG* in C, and *SEP1*, *SEP2*, *SEP3*, and *SEP4 in* E. Based on the gene expression data in petals and stamens in three developmental stages, we observed that the expression of the alleles of these four genes also showed significant differences (Fig. 5a and b, Table S13). In *M. montana*, the expression of the A genes was very low. *AP1* and *AP2* were not expressed in the first and third developmental stages of the stamen, and *AP2* was not expressed in the petals in the second and third developmental stages. This indicated that A genes played a key role in the process of petal formation. The differential expression of alleles may also be responsible for the flower morphology of *M. alba* (10 flower petals, lanceolate, petals hard), which is more similar to *M. champaca*. Furthermore, the analysis of the methylation sites in the promoter regions of the four types of genes revealed that the methylation levels in the CHG sites of *M. montana* were significantly higher than those of *M. champaca*. (Fig. 5c).

### Expansion of the TPS genes leads to the generation of unique floral fragrances

The *M. montana* and *M. champaca* flowers emit a distinctive and delightful aroma. We detected a total of 35 terpenoid compounds in *M. champaca* and *M. montana* flowers, including 13 monoterpenes, 14 sesquiterpenes, 3 diterpenes, 1 triterpene, and 4 cyclic ether terpenes. There were significant differences in the composition of these terpenoids between *M. champaca* and *M. montana*, as depicted in Figure. S10. This indicates that the distinct aroma profiles of the two species are closely associated with variations in terpenoid composition. In addition, our differential methylation analysis indicated that *M. champaca* and *M. montana* had a high abundance of terpenoids, but each had a unique composition of terpenoid compounds. *M. champaca* had a higher concentration of diterpenes, while *M. montana* had a higher concentration of sesquiterpenoids. Therefore, we have intensively investigated the key gene family of terpenoid synthesis, terpene synthase (TPS).

**Fig.6:**
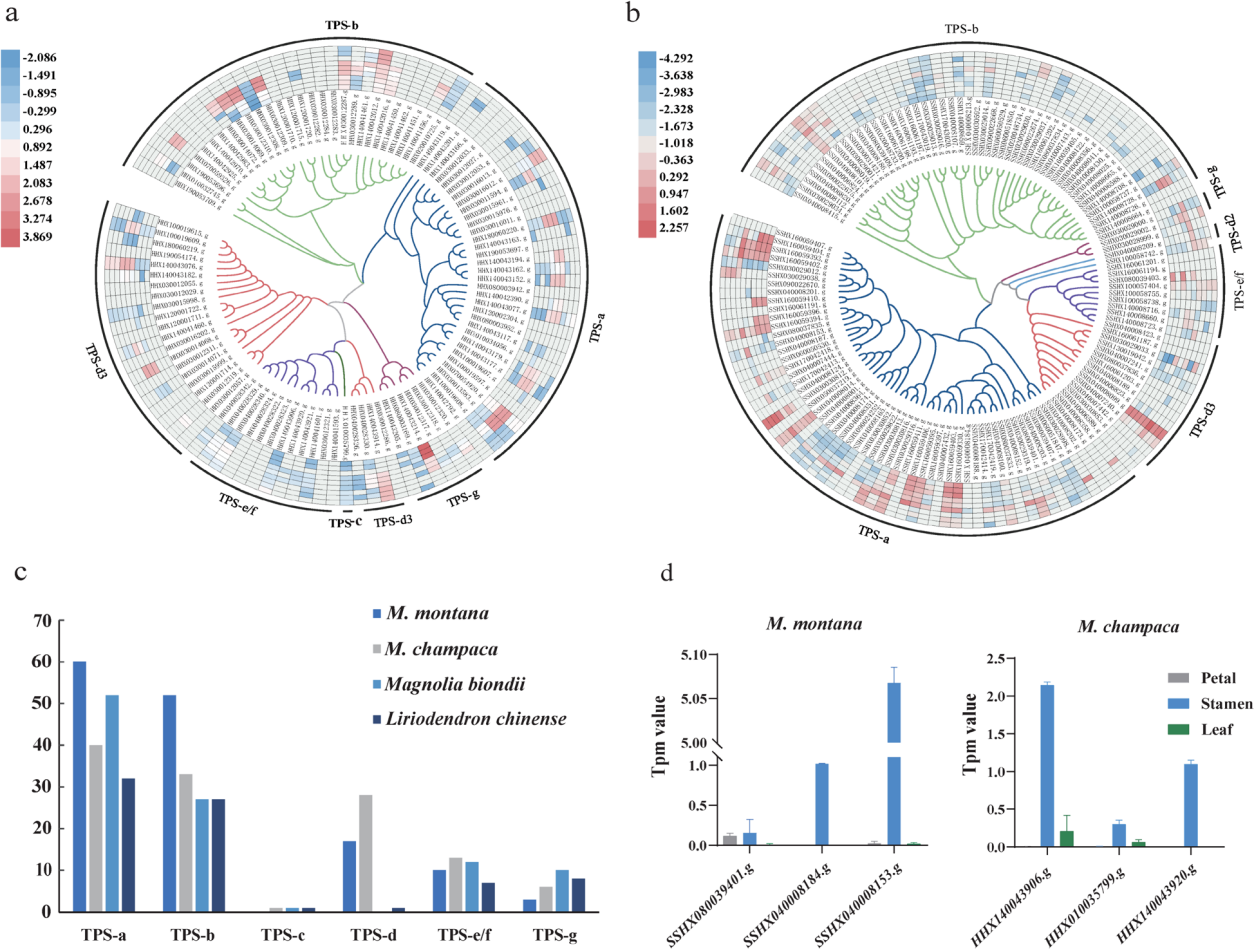
Analysis of the TPS gene family in *M. champaca* and *M. montana*. **a**. Phylogenetic tree of TPS members in *M. champaca*. The circles inward to outward correspond to the petals and stamens in three stages, from young to mature, and the mature leaves. **b**. Phylogenetic tree of TPS members in *M. montana*. **c**. Comparison of *M. montana, M. champaca, L. chinense,* and *M. biondii* TPS gene numbers. **d**. The RNA-seq expression levels of TPS genes related to terpene synthesis in *M. champaca* and *M. montana* were obtained by differential methylation analysis

We identified TPS gene family members in four species, *L. chinense*, *M. biondii*, *M. montana*, and *M. champaca,* based on high-quality gene sequence data and annotation. We found that the number of TPS genes in *M. montana* was significantly higher, with 140 genes present in the genome (Fig. 6a). *M. champaca* had 121 genes (Fig. 6b), and *L. chinense* had the lowest number of genes, with only 76 (Table S14, Fig. 6c). We classified the detected TPS genes and discovered the presence of the TPS-c gene in *M. champaca*. This gene is responsible for diterpene synthesis and is uniquely present in *M. champaca* compared to *M. montana*. Additionally, we observed a significant expansion of TPS-a genes in *M. montana*, which are primarily responsible for synthesizing sesquiterpenoids. By analyzing the transcriptome data from the petals, stamens, and leaves, we observed that gene expression in stamens tended to be higher than that in the other two organs. This suggests that the stamens may play a key role in producing the floral aroma through volatile terpenoid biosynthesis (Fig. 6a and b).

Based on the KEGG and GO enrichment results in *M. champaca* and *M. montana* flower and leaf DMR genes, We found that they were enriched in diterpenoid biosynthesis pathways in *M. champaca* and sesquiterpenoid biosynthesis pathways in *M. montana*, respectively. (Fig. 6d, Table S15).

## Discussion

Magnoliaceae is a relatively newly evolved Magnoliid family. An increasing number of Magnoliid genomes provide theoretical evidence for the evolutionary position of Magnoliids. In recent years, the completion and release of *L. chinense* and *M. biondii* genomes have increased our understanding of Magnoliaceae evolution. After hybridizing *M. montana* and *M. champaca*, we assembled two sets of *M. alba* haplotype genomes using second-generation sequencing data from the two parents and Nanopore sequencing data from their offspring.

Previous research has suggested that the evolutionary status of the *Magnoliidae* subclass remains elusive. Upon analyzing the first genome of the Magnolia species *L. chinense*, researchers hypothesized that Magnolia was the sister group of a monocotyledonous group, with that being the base case, except for species such as *A. trichopoda*. The *C. kanehirae* genome that was released later redefined the status of Magnolia genomes. This result indicated that the Magnolia group was a sister group to dicotyledonous plants, and monocotyledons were the more ancient basal group. Assessing the *A. debilis*genome revealed that the Magnolia group and monocots are sister groups, while dicots are the ancestral group. Based on the haplotype genome analyzed in this study, we can confirm that monocotyledons are the ancestral group, while Magnolia and dicotyledons are sister groups to one another. We also utilized low-copy homologous genes to construct a phylogenetic tree, revealing that Magnoliids and Eudicots are sister clades, with monocots being their sister lineage. Previously, with the release of the first genome from the last branch of angiosperm phylogeny – *Chloranthales *(Ma et al. 2021; Guo et al. 2021), researchers have assessed the evolutionary relationship between Eudicots, Monocots, *Magnolias, Ceratophyllales*, and *Chloranthales*. The conclusions drawn from the various analyses are consistent with those presented in this study.

*M. alba* is a commonly occurring hybrid in the *Michelia* L genus and exhibits traits of incompatibility resulting from distant hybridization. Its parents are *M. montana* and *M. champaca*, belonging to the *Michelia* L genus, and are distantly related. It is believed that the successful hybridization of *M. montana* and *M. champaca* may be attributed to the fact that all the species in the *Michelia* L genus have the same chromosome number, 2n = 19*.* The large amount of pollen in the Magnoliaceae family is a distinctive characteristic that plays a crucial role in the hybridization process. The unique smell and shape of *Michelia* L species flowers further encourage insects to assist pollination, ultimately reducing the impact of chromosome mismatches during meiosis (Bernhardt and Thien 1987). As a result, there is frequent successful hybridization.

Many hybrid species exist in *Michelia* L, such as *Michelia crassipes* × *M. figo, M. crassipes* × *M. maudiae*. Species of other genera of Magnoliaceae are also frequently hybridized. These include crosses within the subgenus Magnolia, such as *M. delavayi* × *M. grandiflora*; crosses within subgenus Yulania, such as *M. denudate* × *M. liliflora*; crosses between the subgenera *Magnolia* and *Yulania,* such as *M. soulangeana* cv.`Hongyun` × *M. odoratissima*; crosses between Michelia and Tsoongiodendron, such as *M. foveolate* × *T. odorum*; crosses between Manglietia and Magnolia, such as *Manglietia sp*. × *M. delay*; crosses between the subgenus Magnolia and Michelia, such as *M. odoratissima* × *M. foveolata* and crosses between Manglietia and Michelia, such as *Manglietia sp*. × *M. crassipes *(Wang and Li y., Zhang SZ. 2003). There is no clear explanation for why hybridization is prevalent in Magnoliaceae. However, with the increasing availability of high-quality genome sequences, breeders may be able to identify the key genes that facilitate successful hybridization between magnolias, resulting in stable offspring. This knowledge can then be applied to improve hybridization efficiency and hybrid breeding in other family species.

Studies of methylation in plants have become increasingly common. There has been in-depth research on methylation mechanisms in rice (Zhang et al. 2018), wheat (Geng, et al. 2019), maize (Lin et al. 2021), *Arabidopsis* (Dooren et al. 2020), and cotton (Yizan, et al. 2018) regarding growth, development, and defense mechanisms. For example, researcher examined the methylation levels and expression levels of individual *FCGs* at different temperatures, which revealed a positive relation between methylation levels and temperature, and a negative relation between the methylation levels and transcript abundances of *FCGs *(Yao et al. 2022). In a previous study, various differentially expressed alleles were identified in the diploid genome and the distribution of differential methylation in the haplotype genomes, thereby providing insights into methylation occurrence in polyploid genomes. We directly assembled and reconstructed the genome of *M. alba* at the haplotype level using Nanopore sequencing and simultaneously detected the methylation sites. This study is the first to reveal the methylation patterns in Magnolia species and the *M. alba* haplotype-resolved genome assembly. These findings provide a valuable data basis for further studying *M. alba* and other Magnolia species.

This study identified many differentially expressed alleles by analyzing the haplotype genomes. This is because *M. alba* is an allodiploid, resulting in a considerable disparity in gene expression between the two haplotype genomes. Moreover, examining methylation sites revealed a strong correlation between these sites and the differentially expressed alleles. The higher expression levels of alleles correlated with lower methylation levels; conversely, lower allele expression levels correlated with higher methylation levels in these genes. Based on this result, the gene expression data of *M. champaca* and *M. montana* were obtained, and it was demonstrated that methylation sites inhibit gene expression.

*M. alba* parents are two distinct species: *M. champaca* with yellow flowers and *M. montana* with white flowers. However, the flowers of *M. alba* do not exhibit the same yellow coloration as their *M. champaca* parent. Upon examining methylation sites, we discovered differences in the methylation patterns in the haplotype alleles of genes responsible for anthocyanin synthesis in *M. alba*. Our findings revealed that the methylation levels of these genes in *M. champaca* were significantly higher than those in *M. montana*. Moreover, based on the transcriptome expression analysis, the expression of anthocyanin synthesis-related genes in *M. champaca* was significantly lower than that in *M. montana*, indicating that increased methylation inhibits their expression in the *M. champaca* haplotype genome. This finding highlights why *M. alba* does not exhibit yellow flowers and offers important insights into improving *M. alba* varieties.

Although the flower color of *M. alba* is more similar to that of *M. montana*, its flower shape has a greater similarity to that of *M. champaca*. The ABC model has been used to study the mechanisms of flower formation in many flowering plants. For example, it was found that in *Dianthus caryophyllus,* the ectopic expression of A genes and C genes might be an important factor affecting the formation of double-perianth in *Dianthus caryophyllus *(Zhang et al. n.d.). The expression patterns of ABCE homologous genes in *Nymphaea colorata *(Zhang et al. 2020) are related to their functions in the different flower organs to a large extent. Based on the results of the above studies, we assessed the sequences of ABCE genes. The primary question was whether these genes had differences in methylation levels. It was apparent that the CHG methylation levels in *M. montana* were generally higher than those in *M. champaca*. Considering this, we compared the allele expression in the two haplotype assemblies. Notably, the expression levels of A genes in *M. montana* were considerably lower than those in *M. champaca*. In contrast, the differential expression of the C and E genes was less pronounced than that of the A genes. Numerous studies have indicated that the growth of petals is a result of the combined action of the A and B genes (Coen and Meyerowitz 1991). In the case of *M. montana* (9), the absence of A gene expression may account for the lower number of petals when compared to *M. champaca* (15–20). Additionally, *M. champaca* and *M. montana* offspring tend to resemble the former due to the superior expression of ABCE genes in *M. champaca*.

*M. alba* is a well-known and popular garden flower and tree in China and an important raw material for the spice industry. Its essential oils and dry fragrance substances are extracted and used to prepare various floral essences, cosmetic essences, perfumes, and more. The aroma is primarily derived from terpenoids. The TPS family genes are responsible for terpenoid biosynthesis and structural diversity. The phylogeny of *M. montana* and *M. champaca* TPS proteins and the comparison of TPS subfamily members with those from other magnolia-like species (Chaw et al. 2019b) revealed an expansion of TPS genes in *M. montana* and *M. champaca*, specifically of the TPS-a and TPS-b members of the subfamily. Regarding the expression profiles in different tissues, the TPS genes were substantially expressed in flowers compared to leaves. The expansion and significant expression of these TPS genes of the TPS-a and TPS-c,g subfamilies in *M. montana* and *M. champaca* are highly associated with the high accumulation of sesquiterpenoids and monoterpenoids in the volatile oils extracted from the flower buds.

## Materials and methods

### Materials and genome analyses

Fresh leaves and flowers were collected from the living collections at Hainan University (Haikou, China) and frozen on-site. High-quality genomic DNA was extracted from freshly frozen leaf and flower tissue from one individual *M. alba*, *M. montana,* and *M. champaca* plant using the Plant Genomic DNA Kit (Tiangen) following the manufacturer's instructions.

Fixed plant samples were prepared and treated with specific DNA probes that were labeled with fluorescent dyes or radioisotopes. These labeled probes were then applied to the samples, allowing them to hybridize with complementary target DNA sequences. Following stringent washing steps to remove unbound probes, the samples were examined under a fluorescence microscope or using autoradiography to visualize and detect the hybridization signals.

JELLYFISH 2.1.4 (Marçais and Kingsford 2011) was employed for the K-mer analysis, utilizing K-values of 19, 21, and 25. The analysis focused on the 25-mer oligos, which were fully visualized. The unique sequence, heterozygosity, duplication, and error rates were determined using GenomeScop (Vurture et al. 2017).

### Genome and methylome sequencing

A short paired-end Illumina DNA library from the parents *M. montana* and *M. champaca*, with a 350 bp insert size (137 × coverage), was sequenced on the Illumina HiSeq 2500 sequencer.

For the *M. alba* offspring, sequencing with the Nanopore Sequel was performed. Two library-building protocols, the Ultralong reads Protocol for *M. alba* and a Ligation Library, were used to obtain 75 Gb (13 ×) Nanopore Ultralong reads and 186 Gb (93 ×) Nanopore reads. The official tool Guppy was used for base calling, and a mean_qscore_template value greater than or equal to 7 was used to obtain pass reads. Pass reads can be directly used for the subsequent assembly. The *M. alba* library produced a total of 261 Gb of data. A PacBio library with an insert size of around 20 kb was then prepared using the SMRTbell Express Template Prep Kit 2.0 from PacBio (Pacific Biosciences, USA). We sequenced the PacBio library on the PacBio Sequel II system (CCS mode), generating 169 Gb clean data (~ 84 ×). A Hi-C library was constructed and sequenced on the Illumina NovaSeq platform for chromosome-level scaffolding, generating 145 Gb of clean data.

### Transcriptome sequencing

We collected samples from the young leaves and stamen tissues of *M. alba*, *M. montana*, and *M. champaca* for transcriptome sequencing. Each tissue sample was isolated and sequenced in three biological replicates to ensure accuracy and reproducibility. To extract the total RNA from the samples, we used the TIANGEN kit with DNASEI, which effectively removes any contaminating DNA. The library was then constructed to prepare it for further processing and analysis. After removing low-quality data, approximately 5.5 Gb of 150-nucleotide paired-end data per sample was used for further RNA-seq analysis.

### Metabolome analysis

LC‒MS-based metabolomics analysis was conducted to investigate the metabolic profiles. Six samples of open flowers from *M. champaca* and *M. montana* were collected, each in three biological replicates.

For metabolite detection, the sample was first ground into a powder using a grinder. Then, 70% aqueous methanol was added to the powder to extract the metabolites. Metabolites were measured by LC‒MS/MS 6500. Normalization was performed by dividing the relative signal intensity of the metabolites by the intensity of the internal standard (Li et al. 2022).

### Genome assembly and evaluation

Canu (Koren et al. 2018) supports using parental short-read sequencing to classify and bin the F1 reads. The long-read progeny sequences were divided into paternal and maternal groups according to haplotype-specific k-mers and assembled separately. Pure third-generation assembly of reads was performed after quality control. The initial genome de novo assembly was done using Hifiasm (Vurture et al. 2017) (v0.15.1) with Nanopore reads, Pacbio Hifi long reads and Hi-C data. To compare the three data generations with the assembled genome, we utilized the minimap2 tool with default parameters. For de-redundancy processing, we employed purge haplotigs (Li et al. 2022) software, utilizing the parameters purge_haplotigs contigcov -l 5; purge_haplotigs purge -a 98. Furthermore, we analyzed the Hi-C libraries using the Juicer pipeline and visualized the results using Juicebox (available at https://github.com/aidenlab/Juicebox).

To predict the gene presence in the existing genome sequence, we performed BUSCO (Koren et al. 2018) prediction using a single copy of the homologous gene in the plant library (eukaryota_odb10).

### Genome annotation

The prediction of repeat sequences was conducted by aligning the sequences and performing repeat-sequence masking using RepeatMasker (v4.0.6) with the default parameters. The utilized reference repeat libraries involved plant short fragment repeats and annotated repeats of MM and MC. The gene structure prediction was based on a combination of evidence-based and ab initio predictions (denovo). Trinity (v2.4.0) was used to assemble transcripts.

The alignment of RNA-seq data to the reference genome was performed using HISAT2 (v2.0.4) (Cheng et al. 2021), and the assembly was conducted using StringTie (v1.2.2) (Roach et al. 2018). The prediction of gene structures included the utilization of SNAP (Korf 2004), AUGUSTUS (v2.7) (Stanke et al. 2004), and GlimmerHMM (Majoros et al. 2004) as ab initio prediction tools. Maker software and the above results were used to carry out evidence-based assembly.

### Haplotype comparison analysis

We utilized protein sequences from MM, MC, and five other angiosperms (*A. trichopoda*, *Liriodendron chinense*, *Magnolia biondii*, and *V. vinifera*) to identify gene families. This was achieved using OrthoFinder (https://github.com/davidemms/OrthoFinder), employing an all-versus-all BLASTP alignment with an e-value cutoff of 1e-5. Subsequently, gene mapping against the KEGG database allowed us to determine potential gene pathways, while the corresponding Inter-ProScan or Pfam results were used for the extraction of GO terms. Furthermore, OrthoVenn2 was utilized to identify orthologous single-copy genes by using single-copy genes from *M. champaca*, *M. montana*, and the aforementioned five angiosperms (*A. trichopoda*, *V. vinifera*, *L. chinense*, and *M. biondii*).

rRNAs were predicted using RNAmmer (Lagesen et al. 2007) (version 1.2), tRNAs were predicted using tRNAscan-SE (Lowe and Eddy 1997) (version 1.23), and other ncRNA sequences were identified using the Perl program Rfam_scan.pl (version 1.0.4) by inner calling using Infernal (Nawrocki and Eddy 2013) (version 1.1.1). The iTAK (Zheng et al. 2016) program was used to detect known TFs in the MM and MC genomes and the other plants evaluated. The predicted gene set was then used as a query to search the database.

To assess the expansion or contraction of gene families, we utilized CAFE (version 3.0) (Tijl et al. 2006). For further analysis, we selected a minimum of five gene families from various species. Random birth and death patterns were employed to evaluate the changes in gene families across each branch of the phylogenetic tree. The Probabilistic Graphical Model was utilized to determine the probability of gene families transitioning between parent and child nodes during the evolutionary process. This allowed us to analyze the expansion and contraction of gene families across all node types.

The MCScanX (Wang et al. 2012) package was applied to construct syntenic blocks based on well-aligned genes to identify homologous regions between the two haplotypes. We screened the syntenic regions according to the identification rules in the potato diploid genome (Zhou et al. 2020). Many gene pairs may be generated during the identification process, and one gene on the complementary haplotype and its best homolog is generally considered to be an allelic gene pair.

### Gene expression analysis

HISAT2 (Kim et al. 2015) was used to construct an index file for the reference genome and mapping. Stringtie (Kovaka et al. 2019), a very commonly used transcriptome expression quantification software, was used to analyze the RNA-seq data and obtain the expression levels in transcripts per million (TPM) of genes on both haplotypes. Two parameters were used to filter the TPM values: (1) Genes showing < 50% difference in expression across three biological replicates were retained. (2) Genes with an average TPM value of 0 for all tissues in three biological replicates were discarded and identified as nonexpressed genes. We compared the differentially expressed allelic genes in two tissues based on genes retained after filtering by TPM value. We considered allelic genes with > twofold expression differences as differentially expressed alleles.

### Methylation analysis

Using tombo (Stoiber et al. 2016) software, the Nanopore sequencing data of the two tissues (flower and leaf) of *M. montana* and *M. champaca* were aligned to the reference genome. Then, deepsignal_plant (Ni et al. 2021) software was used to identify methylation sites, and the methylation sites were annotated and plotted. To define the differentially methylated regions (DMRs) between the two haplotypes, we utilized the DSS (Feng et al. 2014) package to obtain the DMRs.

### Identification of TPS genes

HMMER (Finn et al. 2011) and BLASTP (Altschul et al. 1990) software were used to identify the TPS genes in *M. champaca* and *M. montana*. The hmm files were downloaded from the http://pfam.xfam.org/database with serial numbers PF01397 and PF03936. Subsequently, the identified genes were further filtered based on an E value < 0.01. The corresponding Arabidopsis protein data were downloaded from the webpage (https://www.arabidopsis.org/), and the genes were compared based on a 30% identity filter. The intersection of the two was used to obtain the TPS gene database of *M. champaca* and *M. montana*. Known TPS sequences were added, muscle (Edgar 2004) software was used to compare the identified sequences, and iqtree (Nguyen et al. 2014) software was used to build tree files and classify TPS genes according to the tree files.

### Supplementary Information

Additional file 1: Fig. S1. Based on the Fluorescence in iitu hybridization experiment results using blue and green light comparison, it was observed that the chromosomes of Michelia champaca and M. alba were fully covered. From left to right are the original hybridization state, green light irradiation state, blue light irradiation state. Fig. S2. K-mer analysis indicated that M. alba has a large genome of approximately 1.8 Gb with 59.1% repetitive elements and is highly heterozygous (4.76%). Fig. S3. K-mer analysis indicated that M. champaca has a large genome of approximately 2.24 Gb but a degree of low heterozygosity (0.38%). Fig. S4. K-mer analysis indicated that M. montana has a large genome of approximately 1.47 Gb and has low heterozygosity (0.95%). Fig. S5. The contigs and scaffolds of the MC (M. champaca) subgenomes were further scaffolded into 19 chromosomes by Hi-C technology, and the anchored genomes were 2.03 Gb (97.19%). Fig. S6. The contigs and scaffolds of the MM (M. montana) subgenomes were further scaffolded into 19 chromosomes by Hi-C technology, and the anchored genomes were 2.06 Gb (97.96%). Fig. S7. KEGG enrichment of the genes from unique families of MC (HHX) and MM (SSHX). Fig. S8. Phylogenetic tree derived from low-copy homologous genes of 17 species. Fig. S9. Anthocyanins represent the most significant differentially abundant metabolites between M. champaca and M. montana. Among these, delphinidins were predominant in M. champaca, with pelargonidin-3-O-glucoside exhibiting the highest concentration Fig. S10. Detected a total of 35 terpenoid compounds in M. champaca and M. montana flowers, including 13 monoterpenes, 14 sesquiterpenes, 3 diterpenes, 1 triterpene, and 4 cyclic ether terpenes. There were significant differences in the composition of these terpenoids between M. champaca and M. montana.

## Data Availability

All the raw sequencing data underlying this article will be shared upon request to the corresponding author. The data supporting the findings of this work are available within the paper and its Supplementary Information files. A reporting summary for this Article is available as a Supplementary Information file. The data sets generated and analyzed during this study are available from the corresponding author upon request. All the raw sequencing data generated during this study have been deposited at National Genomics Data Center as a BioProject under accession PRJCA008087. Transcriptome sequence reads have been deposited in the GSA database under BioProject number CRA006224, CRA006225 and CRA006110. The genome assemblies and annotation files are available at the website https://ngdc.cncb.ac.cn/.

## Abbreviations

4CL: 4-Coumarate: CoA ligase
AG: Agamous
AP: Apetala
Busco: Benchmarking Universal Single-Copy Orthologs
C4H: Cinnamate 4-hydroxylase
CDS: Coding DNA sequence
DMRs: Differentially methylated regions
FISH: Fluorescence In Situ Hybridization
GO: Gene ontology
Ks: The number of substitutions per synonymous site
LC–ESI–MS/MS: Liquid chromatography–electrospray ionization tandem mass spectrometry
LINEs: Long interspersed nuclear elements
LTR: Long Terminal Repeat
ONT: Oxford Nanopore Technologies
PAL: Phenylalanine ammonia lyase
PI: Pistillata
QV: Quality value
SEP: Sepallata
snRNAs: Small nuclear RNAs
TPS: Terpene synthase
tRNAs: Transfer RNAs
TSS: Transcription start point
TTS: Transcription termination point
WGD: Whole genome duplications
