# Supplementary material for: A high-quality haplotype genome of Michelia alba DC reveals differences in methylation patterns and flower characteristics
Source: Mol Hortic. 2024 May 29;4:23. doi: 10.1186/s43897-024-00098-z (PMC11134676; doi:10.1186/s43897-024-00098-z)
Supplement: Supplementary file 1 — Additional file 1: Fig. S1. Based on the Fluorescence in iitu hybridization experiment results using blue and green light comparison, it was observed that the chromosomes of Michelia champaca and M. alba were fully covered. From left to right are the original hybridization state, green light irradiation state, blue light irradiation state. Fig. S2. K-mer analysis indicated that M. alba has a large genome of approximately 1.8 Gb with 59.1% repetitive elements and is highly heterozygous (4.76%). Fig. S3. K-mer analysis indicated that M. champaca has a large genome of approximately 2.24 Gb but a degree of low heterozygosity (0.38%). Fig. S4. K-mer analysis indicated that M. montana has a large genome of approximately 1.47 Gb and has low heterozygosity (0.95%). Fig. S5. The contigs and scaffolds of the MC (M. champaca) subgenomes were further scaffolded into 19 chromosomes by Hi-C technology, and the anchored genomes were 2.03 Gb (97.19%). Fig. S6. The contigs and scaffolds of the MM (M. montana) subgenomes were further scaffolded into 19 chromosomes by Hi-C technology, and the anchored genomes were 2.06 Gb (97.96%). Fig. S7. KEGG enrichment of the genes from unique families of MC (HHX) and MM (SSHX). Fig. S8. Phylogenetic tree derived from low-copy homologous genes of 17 species. Fig. S9. Anthocyanins represent the most significant differentially abundant metabolites between M. champaca and M. montana. Among these, delphinidins were predominant in M. champaca, with pelargonidin-3-O-glucoside exhibiting the highest concentration Fig. S10. Detected a total of 35 terpenoid compounds in M. champaca and M. montana flowers, including 13 monoterpenes, 14 sesquiterpenes, 3 diterpenes, 1 triterpene, and 4 cyclic ether terpenes. There were significant differences in the composition of these terpenoids between M. champaca and M. montana. [file 43897_2024_98_MOESM1_ESM.docx]

# Supplementary Table

## Table S1. Raw sequencing data and detailed assembly results

**A.**

| Illumina paired-end reads | R1 | R2 |
| --- | --- | --- |
| Michelia champaca | 25,246,017,905 | 25,069,966,463 |
| *Michelia montaua* | 22,522,532,311 | 22,459,286,635 |
| Nanopore reads | ONT | Ultralong |
| Michelia alba DC | 186,242,657,532 | 75,132,546,798 |

**B.**

| Hic reads |  |
| --- | --- |
| Michelia alba DC_R1 | 19,132,719,634 |
| Michelia alba DC_R2 | 19,654,971,995 |

**C.**

| **MM** | Contig Stats | Scaffold Stats |
| --- | --- | --- |
| N10 | 10,430,713 | 170,900,720 |
| N20 | 6,822,821 | 148,787,130 |
| N30 | 5,478,730 | 136,487,962 |
| N40 | 4,329,226 | 123,743,037 |
| N50 | 3,361,019 | 113,928,620 |
| gc_content | 39.67543055 | 39.67543054 |
| longest | 24,868,908 | 199,171,603 |
| mean | 1,014,262 | 108,723,896 |
| median | 275,000 | 104,116,100 |
| sequence_count | 2,036 | 19 |
| shortest | 38 | 63,368,520 |
| total_bps | 2,065,037,109 | 2,065,754,029 |

**D.**

| **MC** | Contig Stats | Scaffold Stats |
| --- | --- | --- |
| N10 | 1,581,000 | 171,854,205 |
| N20 | 929,746 | 165,189,933 |
| N30 | 672,058 | 154,091,010 |
| N40 | 526,040 | 152,351,171 |
| N50 | 415,948 | 109,650,432 |
| gc_content | 39.98902174 | 39.98902174 |
| longest | 8,732,000 | 201,520,532 |
| mean | 267,520 | 107,091,672 |
| median | 179,560 | 94,569,640 |
| sequence_count | 7,593 | 19 |
| shortest | 10 | 46,215,458 |
| total_bps | 2,031,279,813 | 2,034,741,773 |

## Table S2. Genome assembly evaluation

|  | *M. champaca* | | *M. montaua* | |
| --- | --- | --- | --- | --- |
|  | count | percentage | count | percentage |
| Complete BUSCOs (C) | 174 | 86.30% | 224 | 90.80% |
| Complete and single-copy BUSCOs (S) | 168 | 83.90% | 215 | 87.30% |
| Complete and duplicated BUSCOs (D) | 6 | 2.40% | 9 | 3.50% |
| Fragmented BUSCOs (F) | 21 | 5.20% | 16 | 8.30% |
| Missing BUSCOs (M) | 60 | 8.50% | 15 | 4.90% |
| Total BUSCO groups searched | 255 | 100% | 255 | 100% |

## Table S3 Repeat sequence prediction

|  | **MC** | | |
| --- | --- | --- | --- |
|  | number of elements | length occupied(bp) | percentage of sequence(%) |
| Retroelements | 518,567 | 719,110,243 | 35.34 |
| LINEs: | 39,540 | 17,576,811 | 0.86 |
| RTE/Bov-B | 21,872 | 5,408,841 | 0.27 |
| L1/CIN4 | 17,668 | 12,167,970 | 0.6 |
| LTR elements: | 479,027 | 701,533,432 | 34.48 |
| Ty1/Copia | 170,816 | 299,768,172 | 14.73 |
| Gypsy/DIRS1 | 307,761 | 401,119,785 | 19.71 |
| DNA transposons | 25,910 | 18,513,339 | 0.91 |
| hobo-Activator | 9,458 | 6,889,653 | 0.34 |
| Tourist/Harbinger | 2,920 | 2,655,283 | 0.13 |
| Total interspersed repeats: |  | 1,506,146,772 | 74.02 |
|  | **MM** | | |
|  | number of elements | length occupied(bp) | percentage of sequence(%) |
| Retroelements | 501,283 | 651,786,772 | 31.55 |
| LINEs: | 56,453 | 25,506,150 | 1.23 |
| RTE/Bov-B | 27,693 | 6,650,058 | 0.32 |
| L1/CIN4 | 28,760 | 18,856,092 | 0.91 |
| LTR elements: | 444,830 | 626,280,622 | 30.32 |
| Ty1/Copia | 148,872 | 253,328,495 | 12.26 |
| Gypsy/DIRS1 | 295,473 | 372,106,171 | 18.01 |
| DNA transposons | 25,257 | 16,249,762 | 0.79 |
| hobo-Activator | 13,887 | 8,925,496 | 0.43 |
| Tourist/Harbinger | 2,040 | 1,882,566 | 0.09 |
| Total interspersed repeats: |  | 1,481,725,506 | 71.73 |

## Table S4 TF prediction

**A**

| ***M. montaua*** | | | | | | | |
| --- | --- | --- | --- | --- | --- | --- | --- |
| SSHX010062015.g | | FAR1 | | | SSHX080040225.g | | FAR1 |
| SSHX010062344.g | | bHLH | | | SSHX080040516.g | | WRKY |
| SSHX010062767.g | | NAC | | | SSHX080040534.g | | WRKY |
| SSHX010062863.g | | FAR1 | | | SSHX080040573.g | | WRKY |
| SSHX010063059.g | | bHLH | | | SSHX080040690.g | | NAC |
| SSHX010063130.g | | bHLH | | | SSHX080041179.g | | WRKY |
| SSHX010063151.g | | NF-YC | | | SSHX080041202.g | | LBD |
| SSHX010063206.g | | Dof | | | SSHX080041219.g | | GATA |
| SSHX010063337.g | | LBD | | | SSHX080041230.g | | bHLH |
| SSHX010063469.g | | bHLH | | | SSHX080041301.g | | NAC |
| SSHX010063567.g | | E2F/DP | | | SSHX080041374.g | | FAR1 |
| SSHX010063737.g | | FAR1 | | | SSHX080041375.g | | FAR1 |
| SSHX010063755.g | | bHLH | | | SSHX080041418.g | | SBP |
| SSHX010063775.g | | bHLH | | | SSHX080041420.g | | SBP |
| SSHX010064351.g | | NF-YB | | | SSHX090021418.g | | GRAS |
| SSHX010064700.g | | FAR1 | | | SSHX090021427.g | | WRKY |
| SSHX010064744.g | | FAR1 | | | SSHX090021430.g | | FAR1 |
| SSHX010064839.g | | GRAS | | | SSHX090021435.g | | FAR1 |
| SSHX010065009.g | | WRKY | | | SSHX090021584.g | | WRKY |
| SSHX010065233.g | | GeBP | | | SSHX090021727.g | | FAR1 |
| SSHX010065236.g | | WRKY | | | SSHX090022756.g | | FAR1 |
| SSHX010065877.g | | bHLH | | | SSHX090022810.g | | NF-YB |
| SSHX010065884.g | | BES1 | | | SSHX090022864.g | | FAR1 |
| SSHX010066046.g | | FAR1 | | | SSHX090022865.g | | FAR1 |
| SSHX010066105.g | | FAR1 | | | SSHX090022916.g | | bHLH |
| SSHX010066113.g | | LBD | | | SSHX090023532.g | | FAR1 |
| SSHX010066536.g | | FAR1 | | | SSHX090023541.g | | FAR1 |
| SSHX010066567.g | | NF-YB | | | SSHX090024313.g | | WRKY |
| SSHX010066594.g | | Dof | | | SSHX090024321.g | | WRKY |
| SSHX010066699.g | | WRKY | | | SSHX090024328.g | | WRKY |
| SSHX010067027.g | | FAR1 | | | SSHX090024346.g | | WRKY |
| SSHX010067151.g | | Dof | | | SSHX090024480.g | | WRKY |
| SSHX010067272.g | | WRKY | | | SSHX090024510.g | | FAR1 |
| SSHX020044522.g | | FAR1 | | | SSHX090024846.g | | bZIP |
| SSHX020044693.g | | WRKY | | | SSHX090024885.g | | FAR1 |
| SSHX020044755.g | | NAC | | | SSHX090024945.g | | bHLH |
| SSHX020044920.g | | FAR1 | | | SSHX090024948.g | | GATA |
| SSHX020044922.g | | NAC | | | SSHX090025050.g | | SBP |
| SSHX020044941.g | | NAC | | | SSHX100055908.g | | FAR1 |
| SSHX020044959.g | | NAC | | | SSHX100055918.g | | GRAS |
| SSHX020045095.g | | SBP | | | SSHX100056148.g | | WRKY |
| SSHX020045211.g | | NF-YB | | | SSHX100056149.g | | WRKY |
| SSHX020045697.g | | Trihelix | | | SSHX100056150.g | | WRKY |
| SSHX020045823.g | | BES1 | | | SSHX100056303.g | | LBD |
| SSHX020046276.g | | ZF-HD | | | SSHX100056354.g | | bHLH |
| SSHX020046310.g | | GATA | | | SSHX100056551.g | | WRKY |
| SSHX020046417.g | | bHLH | | | SSHX100056697.g | | LBD |
| SSHX020046466.g | | bHLH | | | SSHX100056830.g | | LBD |
| SSHX020046530.g | | bHLH | | | SSHX100057250.g | | NAC |
| SSHX020046896.g | | GRAS | | | SSHX100057582.g | | NF-YC |
| SSHX020046995.g | | bHLH | | | SSHX100057675.g | | FAR1 |
| SSHX020047052.g | | LBD | | | SSHX100057733.g | | NAC |
| SSHX020047485.g | | E2F/DP | | | SSHX100058310.g | | WRKY |
| SSHX020047492.g | | bHLH | | | SSHX100058443.g | | NAC |
| SSHX020047739.g | | WRKY | | | SSHX100058447.g | | FAR1 |
| SSHX020048105.g | | GRAS | | | SSHX100058569.g | | BES1 |
| SSHX020048110.g | | LBD | | | SSHX100058725.g | | NAC |
| SSHX020048208.g | | GRAS | | | SSHX100058863.g | | NAC |
| SSHX020048226.g | | FAR1 | | | SSHX110025548.g | | NAC |
| SSHX020048306.g | | bZIP | | | SSHX110025905.g | | SBP |
| SSHX020048423.g | | bHLH | | | SSHX110026032.g | | Nin-like |
| SSHX020048455.g | | bHLH | | | SSHX110026163.g | | NAC |
| SSHX020048960.g | | bZIP | | | SSHX110026166.g | | NAC |
| SSHX030028483.g | | bHLH | | | SSHX110026262.g | | Trihelix |
| SSHX030028584.g | | NAC | | | SSHX110026458.g | | FAR1 |
| SSHX030028585.g | | NAC | | | SSHX110026469.g | | bHLH |
| SSHX030028946.g | | bHLH | | | SSHX110026660.g | | FAR1 |
| SSHX030029017.g | | GRAS | | | SSHX110026854.g | | ZF-HD |
| SSHX030029039.g | | GRAS | | | SSHX110026946.g | | NAC |
| SSHX030029040.g | | GRAS | | | SSHX110026977.g | | NAC |
| SSHX030029056.g | | GeBP | | | SSHX110027109.g | | NAC |
| SSHX030029099.g | | bHLH | | | SSHX110027146.g | | GRAS |
| SSHX030029682.g | | FAR1 | | | SSHX110027285.g | | WRKY |
| SSHX030029798.g | | Dof | | | SSHX110027344.g | | HSF |
| SSHX030030200.g | | FAR1 | | | SSHX110027388.g | | HSF |
| SSHX030030601.g | | NF-YA | | | SSHX110027630.g | | NAC |
| SSHX030030755.g | | NAC | | | SSHX110027631.g | | NAC |
| SSHX030030760.g | | NAC | | | SSHX110027657.g | | Dof |
| SSHX030030790.g | | bHLH | | | SSHX110027678.g | | Nin-like |
| SSHX030030797.g | | bHLH | | | SSHX110027727.g | | NAC |
| SSHX030031238.g | | LBD | | | SSHX110027765.g | | bHLH |
| SSHX030031280.g | | NAC | | | SSHX110027856.g | | NF-YB |
| SSHX030031451.g | | GRAS | | | SSHX110028390.g | | bHLH |
| SSHX030031452.g | | GRAS | | | SSHX110028399.g | | bHLH |
| SSHX030031984.g | | bHLH | | | SSHX110028435.g | | ZF-HD |
| SSHX030032434.g | | WRKY | | | SSHX110028482.g | | bHLH |
| SSHX030032439.g | | SBP | | | SSHX120000178.g | | LBD |
| SSHX030032513.g | | NF-YC | | | SSHX120000469.g | | FAR1 |
| SSHX030032678.g | | NAC | | | SSHX120000470.g | | FAR1 |
| SSHX030032808.g | | bHLH | | | SSHX120001025.g | | FAR1 |
| SSHX030032825.g | | Trihelix | | | SSHX120001035.g | | bZIP |
| SSHX040003946.g | | FAR1 | | | SSHX120001137.g | | WRKY |
| SSHX040004143.g | | LBD | | | SSHX120001208.g | | bHLH |
| SSHX040004205.g | | FAR1 | | | SSHX120001265.g | | NAC |
| SSHX040004456.g | | NF-YC | | | SSHX120001442.g | | bZIP |
| SSHX040004541.g | | LBD | | | SSHX120001448.g | | Trihelix |
| SSHX040004596.g | | NF-YC | | | SSHX120001523.g | | Trihelix |
| SSHX040004797.g | | WRKY | | | SSHX120001645.g | | NAC |
| SSHX040004846.g | | LBD | | | SSHX120001792.g | | NAC |
| SSHX040005142.g | | bHLH | | | SSHX120001921.g | | Dof |
| SSHX040005172.g | | WRKY | | | SSHX120001940.g | | HSF |
| SSHX040005285.g | | CAMTA | | | SSHX120002081.g | | TCP |
| SSHX040005443.g | | WRKY | | | SSHX120002186.g | | LBD |
| SSHX040005555.g | | GRAS | | | SSHX120002199.g | | LBD |
| SSHX040005746.g | | GATA | | | SSHX120002374.g | | NAC |
| SSHX040007479.g | | NAC | | | SSHX120002378.g | | YABBY |
| SSHX040007775.g | | FAR1 | | | SSHX120002430.g | | FAR1 |
| SSHX040007944.g | | SRS | | | SSHX120002693.g | | NAC |
| SSHX040007945.g | | SRS | | | SSHX120002866.g | | WRKY |
| SSHX040008090.g | | bHLH | | | SSHX120002887.g | | bHLH |
| SSHX040008139.g | | GRAS | | | SSHX120002971.g | | HSF |
| SSHX040008232.g | | GRAS | | | SSHX120003040.g | | BBR-BPC |
| SSHX040008233.g | | bHLH | | | SSHX120003116.g | | bHLH |
| SSHX050033318.g | | FAR1 | | | SSHX120003163.g | | FAR1 |
| SSHX050033511.g | | NAC | | | SSHX120003169.g | | FAR1 |
| SSHX050033805.g | | bZIP | | | SSHX120003251.g | | BES1 |
| SSHX050034117.g | | bZIP | | | SSHX130018483.g | | NAC |
| SSHX050034355.g | | E2F/DP | | | SSHX130018733.g | | SBP |
| SSHX050034552.g | | WRKY | | | SSHX130018779.g | | EIL |
| SSHX050034852.g | | FAR1 | | | SSHX130018780.g | | EIL |
| SSHX050035178.g | | Dof | | | SSHX130018804.g | | bHLH |
| SSHX050035266.g | | NAC | | | SSHX130018866.g | | Trihelix |
| SSHX050035308.g | | TCP | | | SSHX130018920.g | | NF-YA |
| SSHX050035812.g | | ZF-HD | | | SSHX130019013.g | | NAC |
| SSHX050035861.g | | bHLH | | | SSHX130019083.g | | Dof |
| SSHX050036105.g | | HSF | | | SSHX130019179.g | | NAC |
| SSHX050036117.g | | bZIP | | | SSHX130019447.g | | bHLH |
| SSHX050036126.g | | bHLH | | | SSHX130019481.g | | bHLH |
| SSHX050036311.g | | bHLH | | | SSHX130019517.g | | CPP |
| SSHX050036345.g | | NAC | | | SSHX130019518.g | | CPP |
| SSHX050036393.g | | GRAS | | | SSHX130019580.g | | HSF |
| SSHX050036435.g | | NAC | | | SSHX130019669.g | | NAC |
| SSHX050036575.g | | NAC | | | SSHX130019670.g | | NAC |
| SSHX050036624.g | | NAC | | | SSHX130020276.g | | WRKY |
| SSHX050036654.g | | Whirly | | | SSHX130020303.g | | WRKY |
| SSHX050037018.g | | TCP | | | SSHX130020548.g | | Trihelix |
| SSHX050037464.g | | NAC | | | SSHX140008512.g | | WRKY |
| SSHX050037530.g | | FAR1 | | | SSHX140008518.g | | bHLH |
| SSHX060049159.g | | BES1 | | | SSHX140008867.g | | LBD |
| SSHX060049183.g | | Trihelix | | | SSHX140008919.g | | NAC |
| SSHX060049392.g | | BBR-BPC | | | SSHX140008963.g | | NAC |
| SSHX060049393.g | | BBR-BPC | | | SSHX140008965.g | | NAC |
| SSHX060049394.g | | BBR-BPC | | | SSHX140008973.g | | NAC |
| SSHX060049492.g | | bHLH | | | SSHX140008976.g | | NAC |
| SSHX060049511.g | | bHLH | | | SSHX140008998.g | | LBD |
| SSHX060049532.g | | GATA | | | SSHX140009054.g | | bZIP |
| SSHX060049622.g | | STAT | | | SSHX140009062.g | | TCP |
| SSHX060049697.g | | NAC | | | SSHX140009080.g | | FAR1 |
| SSHX060049872.g | | SBP | | | SSHX140009158.g | | bHLH |
| SSHX060049874.g | | SBP | | | SSHX140009341.g | | HSF |
| SSHX060049893.g | | bHLH | | | SSHX140009508.g | | NAC |
| SSHX060049968.g | | GRAS | | | SSHX140009601.g | | WRKY |
| SSHX060049969.g | | GRAS | | | SSHX140009730.g | | WRKY |
| SSHX060050052.g | | YABBY | | | SSHX140009941.g | | bHLH |
| SSHX060050060.g | | NAC | | | SSHX140010529.g | | NAC |
| SSHX060050192.g | | LBD | | | SSHX140011123.g | | GRAS |
| SSHX060050404.g | | GRAS | | | SSHX140011161.g | | bZIP |
| SSHX060050458.g | | HSF | | | SSHX150016058.g | | FAR1 |
| SSHX060050482.g | | Dof | | | SSHX150016293.g | | GRAS |
| SSHX060050520.g | | FAR1 | | | SSHX150016320.g | | NAC |
| SSHX060050687.g | | NAC | | | SSHX150016596.g | | BBR-BPC |
| SSHX060050945.g | | NAC | | | SSHX150016692.g | | bZIP |
| SSHX060050946.g | | NAC | | | SSHX150016924.g | | FAR1 |
| SSHX060051172.g | | Trihelix | | | SSHX150017275.g | | FAR1 |
| SSHX060051173.g | | Trihelix | | | SSHX150017312.g | | bHLH |
| SSHX060051329.g | | NF-YA | | | SSHX150017735.g | | FAR1 |
| SSHX060051357.g | | NF-YA | | | SSHX150017803.g | | FAR1 |
| SSHX060051613.g | | FAR1 | | | SSHX150017893.g | | HSF |
| SSHX060051699.g | | bHLH | | | SSHX150017963.g | | bZIP |
| SSHX060051705.g | | WRKY | | | SSHX150018178.g | | ZF-HD |
| SSHX060052451.g | | GATA | | | SSHX150018300.g | | bZIP |
| SSHX060052469.g | | NAC | | | SSHX150018328.g | | bZIP |
| SSHX060052470.g | | NAC | | | SSHX150018445.g | | NAC |
| SSHX060052796.g | | CPP | | | SSHX150018449.g | | NAC |
| SSHX070053239.g | | NF-YA | | | SSHX150018469.g | | NAC |
| SSHX070053772.g | | bHLH | | | SSHX160059826.g | | E2F/DP |
| SSHX070053846.g | | TCP | | | SSHX160060007.g | | bHLH |
| SSHX070054260.g | | SRS | | | SSHX160060088.g | | Trihelix |
| SSHX070054362.g | | NAC | | | SSHX160060164.g | | HSF |
| SSHX070054373.g | | NAC | | | SSHX160060194.g | | Trihelix |
| SSHX070054374.g | | NAC | | | SSHX160060195.g | | Trihelix |
| SSHX070054425.g | | Dof | | | SSHX160060593.g | | NAC |
| SSHX070054432.g | | LBD | | | SSHX160060910.g | | TCP |
| SSHX070054532.g | | EIL | | | SSHX160061238.g | | bHLH |
| SSHX070054549.g | | FAR1 | | | SSHX160061289.g | | LBD |
| SSHX070055435.g | | FAR1 | | | SSHX160061576.g | | bHLH |
| SSHX070055748.g | | GRAS | | | SSHX160061672.g | | YABBY |
| SSHX070055749.g | | GRAS | | | SSHX160061673.g | | YABBY |
| SSHX070055751.g | | FAR1 | | | SSHX160061782.g | | LBD |
| SSHX080037767.g | | WRKY | | | SSHX160061783.g | | LBD |
| SSHX080037778.g | | GRAS | | | SSHX160061835.g | | bZIP |
| SSHX080037823.g | | SAP | | | SSHX170041548.g | | NF-YA |
| SSHX080037884.g | | NF-YB | | | SSHX170041636.g | | Trihelix |
| SSHX080037971.g | | YABBY | | | SSHX170041818.g | | bZIP |
| SSHX080038519.g | | NF-YC | | | SSHX170041819.g | | EIL |
| SSHX080039390.g | | bZIP | | | SSHX170041861.g | | SBP |
| SSHX080039621.g | | bHLH | | | SSHX170042006.g | | SBP |
| SSHX080039633.g | | Dof | | | SSHX170042323.g | | bHLH |
| SSHX080039674.g | | NF-YB | | | SSHX170042453.g | | FAR1 |
| SSHX080040056.g | | GRAS | | | SSHX170042481.g | | FAR1 |
| SSHX170042529.g | | bHLH | | SSHX020048039.g | | M-type_MADS | | |
| SSHX170042722.g | | NAC | | SSHX020048393.g | | M-type_MADS | | |
| SSHX170042951.g | | bHLH | | SSHX030032451.g | | M-type_MADS | | |
| SSHX170042984.g | | bHLH | | SSHX030032460.g | | M-type_MADS | | |
| SSHX170043000.g | | CPP | | SSHX040003640.g | | M-type_MADS | | |
| SSHX170043098.g | | TCP | | SSHX050035170.g | | M-type_MADS | | |
| SSHX170043126.g | | YABBY | | SSHX050036759.g | | M-type_MADS | | |
| SSHX170043170.g | | YABBY | | SSHX060052005.g | | M-type_MADS | | |
| SSHX170043247.g | | bZIP | | SSHX060052016.g | | M-type_MADS | | |
| SSHX170043493.g | | FAR1 | | SSHX080037957.g | | M-type_MADS | | |
| SSHX170043655.g | | WRKY | | SSHX100057599.g | | M-type_MADS | | |
| SSHX170043708.g | | WRKY | | SSHX110025564.g | | M-type_MADS | | |
| SSHX170043783.g | | SRS | | SSHX110026717.g | | M-type_MADS | | |
| SSHX170044034.g | | NAC | | SSHX110027046.g | | M-type_MADS | | |
| SSHX170044042.g | | E2F/DP | | SSHX110027968.g | | M-type_MADS | | |
| SSHX170044043.g | | E2F/DP | | SSHX120001299.g | | M-type_MADS | | |
| SSHX180014081.g | | NAC | | SSHX120002655.g | | M-type_MADS | | |
| SSHX180014516.g | | FAR1 | | SSHX140009226.g | | M-type_MADS | | |
| SSHX180014824.g | | GRAS | | SSHX140009385.g | | M-type_MADS | | |
| SSHX180014825.g | | GRAS | | SSHX180015738.g | | M-type_MADS | | |
| SSHX180014826.g | | GRAS | | SSHX190012530.g | | M-type_MADS | | |
| SSHX180014827.g | | GRAS | | SSHX190012532.g | | M-type_MADS | | |
| SSHX180014828.g | | GRAS | | SSHX190012566.g | | M-type_MADS | | |
| SSHX180014918.g | | bHLH | | SSHX190013115.g | | M-type_MADS | | |
| SSHX180015358.g | | LFY | | SSHX020046607.g | | GRF | | |
| SSHX180015360.g | | LFY | | SSHX020048309.g | | GRF | | |
| SSHX180015457.g | | bHLH | | SSHX060049221.g | | GRF | | |
| SSHX180015557.g | | NAC | | SSHX060050677.g | | GRF | | |
| SSHX180015574.g | | NAC | | SSHX070053167.g | | GRF | | |
| SSHX180015599.g | | GRAS | | SSHX010062327.g | | HB-other | | |
| SSHX180015600.g | | GRAS | | SSHX010064389.g | | HB-other | | |
| SSHX180015744.g | | SBP | | SSHX020045820.g | | HB-other | | |
| SSHX190011913.g | | LBD | | SSHX020045846.g | | HB-other | | |
| SSHX190012135.g | | GATA | | SSHX020047706.g | | HB-other | | |
| SSHX190012223.g | | FAR1 | | SSHX020048953.g | | HB-other | | |
| SSHX190012227.g | | FAR1 | | SSHX030029410.g | | HB-other | | |
| SSHX190012315.g | | NAC | | SSHX050035248.g | | HB-other | | |
| SSHX190012763.g | | WRKY | | SSHX060050424.g | | HB-other | | |
| SSHX190012840.g | | SBP | | SSHX060052043.g | | HB-other | | |
| SSHX190012862.g | | FAR1 | | SSHX080040355.g | | HB-other | | |
| SSHX190012888.g | | bHLH | | SSHX090024997.g | | HB-other | | |
| SSHX190012931.g | | WRKY | | SSHX120001371.g | | HB-other | | |
| SSHX190013014.g | | NAC | | SSHX120001833.g | | HB-other | | |
| SSHX190013033.g | | LBD | | SSHX120001954.g | | HB-other | | |
| SSHX190013290.g | | BES1 | | SSHX120003278.g | | HB-other | | |
| SSHX190013295.g | | HSF | | SSHX150016592.g | | HB-other | | |
| SSHX190013321.g | | WRKY | | SSHX170043011.g | | HB-other | | |
| SSHX190013402.g | | Trihelix | | SSHX180014979.g | | HB-other | | |
| SSHX190013506.g | | FAR1 | | SSHX190013657.g | | HB-other | | |
| SSHX190013728.g | | NAC | | SSHX190013700.g | | HB-other | | |
| SSHX190013729.g | | NAC | | SSHX010066749.g | | WOX | | |
| SSHX190013731.g | | NAC | | SSHX070053703.g | | WOX | | |
| SSHX190013780.g | | Nin-like | | SSHX050035010.g | | WOX | | |
| SSHX190013805.g | | Nin-like | | SSHX050033600.g | | WOX | | |
| SSHX190013946.g | | WRKY | | SSHX050033280.g | | WOX | | |
| SSHX050037312.g | | ARF | | SSHX070054429.g | | WOX | | |
| SSHX090024816.g | | ARF | | SSHX160059474.g | | TALE | | |
| SSHX110026804.g | | ARF | | SSHX170042672.g | | TALE | | |
| SSHX140008834.g | | ARF | | SSHX120003074.g | | HD-ZIP | | |
| SSHX020046091.g | | B3 | | SSHX110027786.g | | HD-ZIP | | |
| SSHX020046095.g | | B3 | | SSHX090023220.g | | HD-ZIP | | |
| SSHX020047048.g | | B3 | | SSHX020048378.g | | HD-ZIP | | |
| SSHX020048795.g | | B3 | | SSHX110028215.g | | HD-ZIP | | |
| SSHX020048796.g | | B3 | | SSHX020046555.g | | HD-ZIP | | |
| SSHX020048797.g | | B3 | | SSHX070053298.g | | HD-ZIP | | |
| SSHX030030455.g | | B3 | | SSHX150016458.g | | HD-ZIP | | |
| SSHX030032793.g | | B3 | | SSHX080041157.g | | HD-ZIP | | |
| SSHX050036034.g | | B3 | | SSHX140010974.g | | HD-ZIP | | |
| SSHX070053886.g | | B3 | | SSHX020048949.g | | HD-ZIP | | |
| SSHX080039945.g | | B3 | | SSHX100058567.g | | HD-ZIP | | |
| SSHX140010719.g | | B3 | | SSHX130019352.g | | HD-ZIP | | |
| SSHX180014309.g | | B3 | | SSHX030030600.g | | HD-ZIP | | |
| SSHX080041074.g | | AP2 | | SSHX170042861.g | | HD-ZIP | | |
| SSHX090025082.g | | AP2 | | SSHX010062845.g | | C2H2 | | |
| SSHX150018284.g | | AP2 | | SSHX010063034.g | | C2H2 | | |
| SSHX140011565.g | | AP2 | | SSHX010064318.g | | C3H | | |
| SSHX170041663.g | | AP2 | | SSHX010064319.g | | C3H | | |
| SSHX180014660.g | | AP2 | | SSHX010065101.g | | C3H | | |
| SSHX080040840.g | | AP2 | | SSHX010067643.g | | C3H | | |
| SSHX020046601.g | | AP2 | | SSHX010068026.g | | C2H2 | | |
| SSHX020048316.g | | AP2 | | SSHX010068151.g | | C3H | | |
| SSHX010062388.g | | ERF | | SSHX020044696.g | | C2H2 | | |
| SSHX010066387.g | | ERF | | SSHX020045553.g | | C3H | | |
| SSHX020044484.g | | ERF | | SSHX020045803.g | | C2H2 | | |
| SSHX020047460.g | | ERF | | SSHX020048114.g | | C2H2 | | |
| SSHX020047716.g | | ERF | | SSHX030029854.g | | C3H | | |
| SSHX030029863.g | | ERF | | SSHX030031401.g | | C3H | | |
| SSHX030032856.g | | ERF | | SSHX030031483.g | | C3H | | |
| SSHX030032919.g | | ERF | | SSHX030032180.g | | C3H | | |
| SSHX040005061.g | | ERF | | SSHX030032181.g | | C3H | | |
| SSHX040005907.g | | ERF | | SSHX040003818.g | | C2H2 | | |
| SSHX050036070.g | | ERF | | SSHX040005173.g | | C2H2 | | |
| SSHX050036079.g | | ERF | | SSHX040005351.g | | C3H | | |
| SSHX050036088.g | | ERF | | SSHX040006156.g | | C3H | | |
| SSHX050036150.g | | ERF | | SSHX040006157.g | | C3H | | |
| SSHX060049772.g | | ERF | | SSHX050034274.g | | C2H2 | | |
| SSHX060049773.g | | ERF | | SSHX050035025.g | | C2H2 | | |
| SSHX060049833.g | | ERF | | SSHX050036308.g | | C3H | | |
| SSHX060050358.g | | ERF | | SSHX050037303.g | | C3H | | |
| SSHX060050360.g | | ERF | | SSHX060049750.g | | C3H | | |
| SSHX060050367.g | | ERF | | SSHX060051113.g | | C2H2 | | |
| SSHX060050368.g | | ERF | | SSHX070053361.g | | C2H2 | | |
| SSHX060050369.g | | ERF | | SSHX070054097.g | | C2H2 | | |
| SSHX060051708.g | | ERF | | SSHX070054323.g | | C3H | | |
| SSHX070052977.g | | ERF | | SSHX080037623.g | | C3H | | |
| SSHX070053050.g | | ERF | | SSHX080037782.g | | C3H | | |
| SSHX080037630.g | | ERF | | SSHX080040260.g | | C2H2 | | |
| SSHX080040412.g | | ERF | | SSHX090021457.g | | C2H2 | | |
| SSHX080041097.g | | ERF | | SSHX090024722.g | | C3H | | |
| SSHX090024276.g | | ERF | | SSHX110026611.g | | C2H2 | | |
| SSHX100056083.g | | ERF | | SSHX110028134.g | | C2H2 | | |
| SSHX100056663.g | | ERF | | SSHX110028156.g | | C2H2 | | |
| SSHX100056853.g | | ERF | | SSHX120000960.g | | C2H2 | | |
| SSHX110026730.g | | ERF | | SSHX120002065.g | | C3H | | |
| SSHX110026739.g | | ERF | | SSHX120002491.g | | C2H2 | | |
| SSHX110027313.g | | ERF | | SSHX120002688.g | | LSD | | |
| SSHX110027398.g | | ERF | | SSHX130018661.g | | C3H | | |
| SSHX120002026.g | | ERF | | SSHX130018871.g | | C2H2 | | |
| SSHX120002027.g | | ERF | | SSHX130018874.g | | C3H | | |
| SSHX120002030.g | | ERF | | SSHX130019155.g | | C3H | | |
| SSHX120002031.g | | ERF | | SSHX140009499.g | | C3H | | |
| SSHX120002033.g | | ERF | | SSHX150016920.g | | C2H2 | | |
| SSHX120002034.g | | ERF | | SSHX160061584.g | | C2H2 | | |
| SSHX120002035.g | | ERF | | SSHX170041638.g | | C2H2 | | |
| SSHX120002037.g | | ERF | | SSHX170041913.g | | C3H | | |
| SSHX120002040.g | | ERF | | SSHX170042700.g | | C3H | | |
| SSHX120002046.g | | ERF | | SSHX170043645.g | | C2H2 | | |
| SSHX120002556.g | | ERF | | SSHX170044119.g | | C3H | | |
| SSHX120002624.g | | ERF | | SSHX180014836.g | | C2H2 | | |
| SSHX120002628.g | | ERF | | SSHX180015832.g | | C3H | | |
| SSHX130018827.g | | ERF | | SSHX190013021.g | | C3H | | |
| SSHX130019388.g | | ERF | | SSHX190013022.g | | C3H | | |
| SSHX140008649.g | | ERF | | SSHX010062112.g | | MYB | | |
| SSHX140009920.g | | ERF | | SSHX010063218.g | | MYB | | |
| SSHX150015988.g | | ERF | | SSHX010063233.g | | MYB | | |
| SSHX150016136.g | | ERF | | SSHX010064273.g | | MYB | | |
| SSHX150016137.g | | ERF | | SSHX010065290.g | | MYB | | |
| SSHX160061298.g | | ERF | | SSHX010065493.g | | MYB | | |
| SSHX170041590.g | | ERF | | SSHX010065628.g | | MYB | | |
| SSHX170043191.g | | ERF | | SSHX020044440.g | | MYB | | |
| SSHX170044186.g | | ERF | | SSHX020046563.g | | MYB | | |
| SSHX180014073.g | | ERF | | SSHX020047833.g | | MYB | | |
| SSHX180014872.g | | ERF | | SSHX020048062.g | | MYB | | |
| SSHX190012158.g | | ERF | | SSHX030028775.g | | MYB | | |
| SSHX180015524.g | | DBB | | SSHX030028819.g | | MYB | | |
| SSHX070054064.g | | CO-like | | SSHX030029061.g | | MYB | | |
| SSHX040005732.g | | ARR-B | | SSHX030029684.g | | MYB | | |
| SSHX010063004.g | | G2-like | | SSHX040005654.g | | MYB | | |
| SSHX010063158.g | | G2-like | | SSHX040006954.g | | MYB | | |
| SSHX010067322.g | | G2-like | | SSHX050035171.g | | MYB | | |
| SSHX020046086.g | | G2-like | | SSHX050035249.g | | MYB | | |
| SSHX020046164.g | | G2-like | | SSHX050035340.g | | MYB | | |
| SSHX020048371.g | | G2-like | | SSHX050035668.g | | MYB | | |
| SSHX020048805.g | | G2-like | | SSHX060050393.g | | MYB | | |
| SSHX030029752.g | | G2-like | | SSHX060050394.g | | MYB | | |
| SSHX030029984.g | | G2-like | | SSHX070053174.g | | MYB | | |
| SSHX030031423.g | | G2-like | | SSHX070053613.g | | MYB | | |
| SSHX050035181.g | | G2-like | | SSHX070054590.g | | MYB | | |
| SSHX050037442.g | | G2-like | | SSHX100056910.g | | MYB | | |
| SSHX060049440.g | | G2-like | | SSHX100056912.g | | MYB | | |
| SSHX060050919.g | | G2-like | | SSHX100057159.g | | MYB | | |
| SSHX060051095.g | | G2-like | | SSHX100057449.g | | MYB | | |
| SSHX070053397.g | | G2-like | | SSHX100058253.g | | MYB | | |
| SSHX070054419.g | | G2-like | | SSHX100058950.g | | MYB | | |
| SSHX070055212.g | | G2-like | | SSHX110027648.g | | MYB | | |
| SSHX080038341.g | | G2-like | | SSHX110027713.g | | MYB | | |
| SSHX080038343.g | | G2-like | | SSHX110027790.g | | MYB | | |
| SSHX080038344.g | | G2-like | | SSHX120001481.g | | MYB | | |
| SSHX090021478.g | | G2-like | | SSHX140009989.g | | MYB | | |
| SSHX090023397.g | | G2-like | | SSHX150017943.g | | MYB | | |
| SSHX090024286.g | | G2-like | | SSHX160061232.g | | MYB | | |
| SSHX090024737.g | | G2-like | | SSHX180014366.g | | MYB | | |
| SSHX100056815.g | | G2-like | | SSHX180014534.g | | MYB | | |
| SSHX100057372.g | | G2-like | | SSHX180014541.g | | MYB | | |
| SSHX100057744.g | | G2-like | | SSHX190012237.g | | MYB | | |
| SSHX110026186.g | | G2-like | | SSHX190012603.g | | MYB | | |
| SSHX110027658.g | | G2-like | | SSHX010062997.g | | MYB_related | | |
| SSHX120002845.g | | G2-like | | SSHX010063633.g | | MYB_related | | |
| SSHX120002987.g | | G2-like | | SSHX010063790.g | | MYB_related | | |
| SSHX130018722.g | | G2-like | | SSHX010064527.g | | MYB_related | | |
| SSHX140008950.g | | G2-like | | SSHX010064726.g | | MYB_related | | |
| SSHX140009243.g | | G2-like | | SSHX010065570.g | | MYB_related | | |
| SSHX140010228.g | | G2-like | | SSHX010066461.g | | MYB_related | | |
| SSHX150016184.g | | G2-like | | SSHX010066999.g | | MYB_related | | |
| SSHX150016646.g | | G2-like | | SSHX010068046.g | | MYB_related | | |
| SSHX150016752.g | | G2-like | | SSHX020046245.g | | MYB_related | | |
| SSHX150017777.g | | G2-like | | SSHX030028803.g | | MYB_related | | |
| SSHX150017973.g | | G2-like | | SSHX030028807.g | | MYB_related | | |
| SSHX150018400.g | | G2-like | | SSHX030028825.g | | MYB_related | | |
| SSHX190013379.g | | G2-like | | SSHX030028827.g | | MYB_related | | |
| SSHX190013713.g | | G2-like | | SSHX030028832.g | | MYB_related | | |
| SSHX050035475.g | | MIKC_MADS | | SSHX030031156.g | | MYB_related | | |
| SSHX170043933.g | | MIKC_MADS | | SSHX030032755.g | | MYB_related | | |
| SSHX010062397.g | | M-type_MADS | | SSHX040004334.g | | MYB_related | | |
| SSHX010065247.g | | M-type_MADS | | SSHX040005397.g | | MYB_related | | |
| SSHX020044660.g | | M-type_MADS | | SSHX040005657.g | | MYB_related | | |
| SSHX020045137.g | | M-type_MADS | | SSHX040006959.g | | MYB_related | | |
| SSHX020045144.g | | M-type_MADS | | SSHX050034486.g | | MYB_related | | |
| SSHX020046344.g | | M-type_MADS | | SSHX050034537.g | | MYB_related | | |
| SSHX020046525.g | | M-type_MADS | | SSHX050034844.g | | MYB_related | | |
| SSHX050034982.g | MYB_related | | SSHX120002929.g | | | MYB_related | | | |
| SSHX050035669.g | MYB_related | | SSHX120003155.g | | | MYB_related | | | |
| SSHX050035902.g | MYB_related | | SSHX130018899.g | | | MYB_related | | | |
| SSHX050036397.g | MYB_related | | SSHX130019141.g | | | MYB_related | | | |
| SSHX050036540.g | MYB_related | | SSHX130019157.g | | | MYB_related | | | |
| SSHX060049227.g | MYB_related | | SSHX130019534.g | | | MYB_related | | | |
| SSHX060049228.g | MYB_related | | SSHX140010641.g | | | MYB_related | | | |
| SSHX060049230.g | MYB_related | | SSHX140011660.g | | | MYB_related | | | |
| SSHX060049471.g | MYB_related | | SSHX150016350.g | | | MYB_related | | | |
| SSHX060050100.g | MYB_related | | SSHX150016504.g | | | MYB_related | | | |
| SSHX060052161.g | MYB_related | | SSHX150016888.g | | | MYB_related | | | |
| SSHX070053182.g | MYB_related | | SSHX150017772.g | | | MYB_related | | | |
| SSHX070053856.g | MYB_related | | SSHX150018052.g | | | MYB_related | | | |
| SSHX070054451.g | MYB_related | | SSHX150018184.g | | | MYB_related | | | |
| SSHX070054681.g | MYB_related | | SSHX150018393.g | | | MYB_related | | | |
| SSHX070055862.g | MYB_related | | SSHX160060043.g | | | MYB_related | | | |
| SSHX070055863.g | MYB_related | | SSHX160061301.g | | | MYB_related | | | |
| SSHX080038444.g | MYB_related | | SSHX160061627.g | | | MYB_related | | | |
| SSHX080040989.g | MYB_related | | SSHX160061843.g | | | MYB_related | | | |
| SSHX090022806.g | MYB_related | | SSHX160061930.g | | | MYB_related | | | |
| SSHX100057267.g | MYB_related | | SSHX170041903.g | | | MYB_related | | | |
| SSHX100058255.g | MYB_related | | SSHX170041970.g | | | MYB_related | | | |
| SSHX110026095.g | MYB_related | | SSHX170042251.g | | | MYB_related | | | |
| SSHX110026151.g | MYB_related | | SSHX170042686.g | | | MYB_related | | | |
| SSHX110028124.g | MYB_related | | SSHX170042789.g | | | MYB_related | | | |
| SSHX120001441.g | MYB_related | | SSHX170043027.g | | | MYB_related | | | |
| SSHX120001923.g | MYB_related | | SSHX180015104.g | | | MYB_related | | | |
| SSHX120002330.g | MYB_related | | SSHX180015115.g | | | MYB_related | | | |
| SSHX120002574.g | MYB_related | | SSHX190013119.g | | | MYB_related | | | |
| SSHX120002648.g | MYB_related | |  | | |  | | | |

**B**

| ***M. champaca*** | | | |
| --- | --- | --- | --- |
| HHX060018603.g | RAV | HHX010036055.g | HSF |
| HHX010032087.g | GATA | HHX010036089.g | bHLH |
| HHX010032953.g | WRKY | HHX010036180.g | NAC |
| HHX010033229.g | HSF | HHX010036400.g | bHLH |
| HHX010033550.g | Trihelix | HHX010036457.g | CPP |
| HHX010033551.g | Trihelix | HHX010036502.g | HSF |
| HHX010033758.g | bHLH | HHX010036596.g | NAC |
| HHX010033895.g | NAC | HHX010036655.g | FAR1 |
| HHX010034105.g | Dof | HHX010036774.g | GRAS |
| HHX010034177.g | GRAS | HHX010036775.g | GRAS |
| HHX010034266.g | TCP | HHX010037398.g | Trihelix |
| HHX010034378.g | LBD | HHX010037488.g | bHLH |
| HHX010034490.g | NAC | HHX010037547.g | LBD |
| HHX010034491.g | NAC | HHX010038016.g | WRKY |
| HHX010034495.g | YABBY | HHX020006867.g | GRAS |
| HHX010034636.g | bHLH | HHX020006868.g | GRAS |
| HHX010034820.g | NAC | HHX020007057.g | bHLH |
| HHX010034993.g | WRKY | HHX020007511.g | LFY |
| HHX010035010.g | bHLH | HHX020007513.g | LFY |
| HHX010035055.g | HSF | HHX020007590.g | bHLH |
| HHX010035110.g | BBR-BPC | HHX020007639.g | NAC |
| HHX010035145.g | BBR-BPC | HHX020007659.g | NAC |
| HHX010035147.g | BBR-BPC | HHX020007987.g | WRKY |
| HHX010035288.g | BES1 | HHX020007996.g | bHLH |
| HHX010035345.g | NAC | HHX020008241.g | NAC |
| HHX010035523.g | SBP | HHX020008242.g | NAC |
| HHX010035570.g | EIL | HHX020008271.g | NAC |
| HHX010035585.g | bZIP | HHX020008376.g | FAR1 |
| HHX010035602.g | bHLH | HHX020008411.g | LBD |
| HHX010035654.g | Trihelix | HHX020008465.g | bHLH |
| HHX010035717.g | NF-YA | HHX020008586.g | WRKY |
| HHX010035772.g | NAC | HHX020008643.g | HSF |
| HHX010035953.g | LBD | HHX020008858.g | LBD |
| HHX010036026.g | LBD | HHX020008870.g | NAC |
| HHX010036048.g | HSF | HHX020008943.g | WRKY |
| HHX010036050.g | HSF | HHX020009003.g | FAR1 |
| HHX010036054.g | FAR1 | HHX020009111.g | WRKY |
| HHX020009227.g | FAR1 | HHX040025246.g | ZF-HD |
| HHX020009526.g | NAC | HHX040025793.g | HRT-like |
| HHX020009910.g | LBD | HHX040026772.g | NAC |
| HHX020010308.g | bHLH | HHX040026774.g | NAC |
| HHX020010585.g | LBD | HHX040026792.g | GRAS |
| HHX020010664.g | NAC | HHX040027257.g | bHLH |
| HHX020010678.g | NAC | HHX040027282.g | GATA |
| HHX020010757.g | SRS | HHX040027455.g | WRKY |
| HHX030011048.g | SBP | HHX040027565.g | WRKY |
| HHX030012263.g | GeBP | HHX040027674.g | NAC |
| HHX030012276.g | GRAS | HHX040027742.g | bHLH |
| HHX030012803.g | NAC | HHX040027857.g | BES1 |
| HHX030012804.g | NAC | HHX040027859.g | HSF |
| HHX030012892.g | LBD | HHX040027921.g | WRKY |
| HHX030012903.g | NF-YA | HHX040028036.g | bHLH |
| HHX030012991.g | Trihelix | HHX040028107.g | TCP |
| HHX030013034.g | bHLH | HHX040028195.g | WRKY |
| HHX030013035.g | bHLH | HHX040028442.g | Nin-like |
| HHX030013206.g | NAC | HHX040028531.g | NAC |
| HHX030013650.g | GATA | HHX040028532.g | NAC |
| HHX030013662.g | bHLH | HHX040028542.g | LBD |
| HHX030013695.g | LBD | HHX040028582.g | bZIP |
| HHX030013719.g | bHLH | HHX040028637.g | GRAS |
| HHX030013932.g | FAR1 | HHX040028666.g | NAC |
| HHX030014013.g | bHLH | HHX040028813.g | HSF |
| HHX030014039.g | bHLH | HHX040028832.g | HSF |
| HHX030014133.g | HSF | HHX040029064.g | bHLH |
| HHX030014147.g | YABBY | HHX040029068.g | bHLH |
| HHX030014215.g | YABBY | HHX040029086.g | bHLH |
| HHX030014312.g | bZIP | HHX040029089.g | bHLH |
| HHX030014567.g | FAR1 | HHX040029113.g | ZF-HD |
| HHX030014719.g | WRKY | HHX040029307.g | NAC |
| HHX030014762.g | WRKY | HHX040029341.g | Nin-like |
| HHX030014821.g | SRS | HHX040029367.g | ZF-HD |
| HHX030014983.g | GATA | HHX040029368.g | ZF-HD |
| HHX030015058.g | FAR1 | HHX040029387.g | NAC |
| HHX030015084.g | NAC | HHX040029410.g | bZIP |
| HHX030015091.g | E2F/DP | HHX040029422.g | bHLH |
| HHX030015461.g | NAC | HHX040029438.g | bHLH |
| HHX030015493.g | LBD | HHX040029625.g | WRKY |
| HHX030016034.g | bHLH | HHX040029628.g | WRKY |
| HHX040029636.g | WRKY | HHX050047777.g | bHLH |
| HHX040029644.g | WRKY | HHX050047785.g | bHLH |
| HHX040029651.g | WRKY | HHX050047802.g | bHLH |
| HHX040029654.g | WRKY | HHX050048370.g | NF-YB |
| HHX040029660.g | WRKY | HHX050048647.g | FAR1 |
| HHX040029668.g | WRKY | HHX050048696.g | GRAS |
| HHX040029709.g | GRAS | HHX050048855.g | FAR1 |
| HHX040029756.g | SBP | HHX050048901.g | bZIP |
| HHX040029875.g | bHLH | HHX050048906.g | YABBY |
| HHX040029881.g | GATA | HHX050048961.g | WRKY |
| HHX040029906.g | LBD | HHX050049102.g | SBP |
| HHX040030031.g | FAR1 | HHX050049138.g | WRKY |
| HHX040030131.g | bHLH | HHX060016486.g | NAC |
| HHX040030163.g | bHLH | HHX060016487.g | NAC |
| HHX040030195.g | LBD | HHX060016500.g | FAR1 |
| HHX040030252.g | bZIP | HHX060016602.g | bZIP |
| HHX040030343.g | WRKY | HHX060016606.g | bZIP |
| HHX040030344.g | WRKY | HHX060016617.g | bZIP |
| HHX040030554.g | FAR1 | HHX060017046.g | FAR1 |
| HHX040030727.g | LBD | HHX060017492.g | FAR1 |
| HHX040031447.g | LBD | HHX060017945.g | bHLH |
| HHX040031450.g | LBD | HHX060018150.g | GRAS |
| HHX040031539.g | WRKY | HHX060018151.g | GRAS |
| HHX040031593.g | E2F/DP | HHX060018152.g | GRAS |
| HHX040031650.g | bHLH | HHX060018364.g | NAC |
| HHX040031657.g | FAR1 | HHX060018630.g | bHLH |
| HHX050044011.g | NAC | HHX060018701.g | bHLH |
| HHX050044026.g | NAC | HHX060018710.g | bZIP |
| HHX050044122.g | bZIP | HHX060018877.g | NF-YC |
| HHX050044436.g | ZF-HD | HHX060018897.g | bHLH |
| HHX050045139.g | bHLH | HHX060018947.g | bHLH |
| HHX050045724.g | HSF | HHX060019061.g | LBD |
| HHX050045750.g | bZIP | HHX060019066.g | NAC |
| HHX050045817.g | ZF-HD | HHX060019135.g | bHLH |
| HHX050045822.g | BBR-BPC | HHX060019357.g | WRKY |
| HHX050045823.g | BBR-BPC | HHX070054822.g | EIL |
| HHX050046118.g | NAC | HHX070055073.g | FAR1 |
| HHX050046829.g | bHLH | HHX070055238.g | FAR1 |
| HHX050047027.g | LBD | HHX070056431.g | bHLH |
| HHX050047551.g | FAR1 | HHX070056449.g | bHLH |
| HHX050047759.g | FAR1 | HHX070056619.g | NAC |
| HHX070056626.g | FAR1 | HHX100020028.g | NAC |
| HHX070056699.g | BES1 | HHX100020069.g | NAC |
| HHX070057295.g | WRKY | HHX100020145.g | NAC |
| HHX070057303.g | GRAS | HHX100020192.g | GRAS |
| HHX070057304.g | GRAS | HHX100020399.g | WRKY |
| HHX070057384.g | FAR1 | HHX100020422.g | bHLH |
| HHX070057529.g | FAR1 | HHX100020436.g | HSF |
| HHX070057686.g | bZIP | HHX100020691.g | bHLH |
| HHX070057943.g | bZIP | HHX100020777.g | Trihelix |
| HHX070058488.g | WRKY | HHX100021121.g | NAC |
| HHX070058613.g | WRKY | HHX100021169.g | Dof |
| HHX070058615.g | WRKY | HHX100021276.g | BES1 |
| HHX070058621.g | GRAS | HHX100021398.g | bHLH |
| HHX070059504.g | FAR1 | HHX100021553.g | bHLH |
| HHX070059572.g | NF-YB | HHX100021569.g | bHLH |
| HHX070059606.g | FAR1 | HHX100021591.g | WRKY |
| HHX080003156.g | bHLH | HHX100021593.g | GATA |
| HHX080003870.g | FAR1 | HHX100021761.g | NAC |
| HHX080004059.g | NAC | HHX100021872.g | GATA |
| HHX080004075.g | NAC | HHX100021885.g | bHLH |
| HHX080004081.g | FAR1 | HHX100021976.g | YABBY |
| HHX080004083.g | NAC | HHX100021979.g | NAC |
| HHX080004084.g | NAC | HHX100022088.g | LBD |
| HHX080004109.g | TCP | HHX100022162.g | TCP |
| HHX080004185.g | SBP | HHX100022274.g | HSF |
| HHX080004341.g | FAR1 | HHX100022295.g | Dof |
| HHX080004874.g | BES1 | HHX100022407.g | NAC |
| HHX080004911.g | NAC | HHX100022512.g | bHLH |
| HHX080004915.g | NAC | HHX100022557.g | NAC |
| HHX080005098.g | Dof | HHX100022717.g | Trihelix |
| HHX080005288.g | bHLH | HHX100022887.g | NAC |
| HHX080005351.g | FAR1 | HHX100022924.g | bHLH |
| HHX080005992.g | NF-YC | HHX100022937.g | bHLH |
| HHX080006470.g | GRAS | HHX100022957.g | WRKY |
| HHX080006471.g | GRAS | HHX100023097.g | HSF |
| HHX080006478.g | FAR1 | HHX100023604.g | FAR1 |
| HHX090024036.g | Dof | HHX110050584.g | WRKY |
| HHX090024539.g | LBD | HHX110050626.g | NF-YC |
| HHX090024833.g | bZIP | HHX110050627.g | NF-YC |
| HHX100019613.g | FAR1 | HHX110050628.g | NF-YC |
| HHX100020021.g | bHLH | HHX110050900.g | FAR1 |
| HHX110050931.g | LBD | HHX130039785.g | bHLH |
| HHX110051367.g | FAR1 | HHX140040347.g | HSF |
| HHX110051481.g | HSF | HHX140040678.g | NF-YC |
| HHX110051732.g | FAR1 | HHX140040733.g | TCP |
| HHX110052519.g | FAR1 | HHX140040839.g | NAC |
| HHX110052727.g | GRAS | HHX140040892.g | bHLH |
| HHX110052845.g | WRKY | HHX140040960.g | Trihelix |
| HHX120000724.g | bHLH | HHX140041201.g | LBD |
| HHX120000739.g | FAR1 | HHX140041222.g | NAC |
| HHX120000808.g | Trihelix | HHX140041384.g | NF-YC |
| HHX120000890.g | HSF | HHX140041703.g | SBP |
| HHX120000911.g | Trihelix | HHX140043095.g | bHLH |
| HHX120001245.g | NAC | HHX140043096.g | bHLH |
| HHX120001256.g | NAC | HHX140043098.g | GRAS |
| HHX120001422.g | TCP | HHX140043099.g | GRAS |
| HHX120001741.g | bHLH | HHX140043100.g | GRAS |
| HHX120001770.g | NAC | HHX140043230.g | GRAS |
| HHX120001924.g | GATA | HHX140043231.g | bHLH |
| HHX120002000.g | bHLH | HHX140043829.g | WRKY |
| HHX120002066.g | YABBY | HHX140043834.g | NAC |
| HHX120002067.g | YABBY | HHX140043839.g | WRKY |
| HHX120002068.g | YABBY | HHX140043932.g | NAC |
| HHX120002069.g | YABBY | HHX140043937.g | FAR1 |
| HHX120002149.g | LBD | HHX150061197.g | FAR1 |
| HHX120002173.g | bZIP | HHX150061615.g | Dof |
| HHX120002510.g | NF-YB | HHX150061648.g | NF-YB |
| HHX120002602.g | YABBY | HHX150061679.g | FAR1 |
| HHX120002743.g | TCP | HHX150061741.g | LBD |
| HHX120002837.g | FAR1 | HHX150062128.g | LBD |
| HHX130038668.g | NAC | HHX150062131.g | Trihelix |
| HHX130038937.g | bHLH | HHX150062310.g | bZIP |
| HHX130038986.g | NAC | HHX160062531.g | bZIP |
| HHX130039041.g | bZIP | HHX160062701.g | bHLH |
| HHX130039169.g | NAC | HHX160062865.g | NAC |
| HHX130039313.g | NAC | HHX160063221.g | bHLH |
| HHX130039342.g | NAC | HHX160063250.g | GRAS |
| HHX130039532.g | NAC | HHX160063251.g | GRAS |
| HHX130039533.g | NAC | HHX160063719.g | BBR-BPC |
| HHX130039535.g | NAC | HHX160063730.g | BBR-BPC |
| HHX130039591.g | bHLH | HHX160063870.g | NF-YA |
| HHX130039608.g | Trihelix | HHX160064017.g | FAR1 |
| HHX160064076.g | bHLH | HHX070057907.g | B3 |
| HHX170049274.g | FAR1 | HHX080005081.g | B3 |
| HHX170049525.g | FAR1 | HHX080005082.g | B3 |
| HHX170049664.g | FAR1 | HHX090023948.g | B3 |
| HHX170049758.g | FAR1 | HHX090024334.g | B3 |
| HHX170049995.g | WRKY | HHX100020492.g | B3 |
| HHX170050007.g | WRKY | HHX110052308.g | B3 |
| HHX170050015.g | WRKY | HHX140040939.g | B3 |
| HHX170050261.g | NAC | HHX190053790.g | B3 |
| HHX180060114.g | FAR1 | HHX020006999.g | AP2 |
| HHX180060152.g | FAR1 | HHX100023187.g | AP2 |
| HHX180060287.g | WRKY | HHX180061119.g | AP2 |
| HHX180060289.g | WRKY | HHX030013015.g | AP2 |
| HHX180060296.g | WRKY | HHX050044227.g | AP2 |
| HHX180060461.g | FAR1 | HHX090024963.g | AP2 |
| HHX180060499.g | bHLH | HHX010033341.g | AP2 |
| HHX180060611.g | bHLH | HHX040030016.g | AP2 |
| HHX180060981.g | FAR1 | HHX010034197.g | ERF |
| HHX180061032.g | bHLH | HHX010034198.g | ERF |
| HHX180061158.g | SBP | HHX010034200.g | ERF |
| HHX190053126.g | GRAS | HHX010034201.g | ERF |
| HHX190053127.g | GRAS | HHX010034202.g | ERF |
| HHX190053128.g | GRAS | HHX010034204.g | ERF |
| HHX190053131.g | LBD | HHX010034250.g | ERF |
| HHX190053208.g | GRAS | HHX010034693.g | ERF |
| HHX190053229.g | FAR1 | HHX010034741.g | ERF |
| HHX190053356.g | bZIP | HHX010034743.g | ERF |
| HHX190053478.g | bHLH | HHX010034744.g | ERF |
| HHX190053628.g | TCP | HHX010035680.g | ERF |
| HHX190053687.g | LBD | HHX010035851.g | ERF |
| HHX190054021.g | GRAS | HHX010036319.g | ERF |
| HHX190054130.g | FAR1 | HHX020006814.g | ERF |
| HHX190054142.g | E2F/DP | HHX020008109.g | ERF |
| HHX180060984.g | ARF | HHX020008412.g | ERF |
| HHX010034807.g | B3 | HHX030011058.g | ERF |
| HHX010035058.g | B3 | HHX030012949.g | ERF |
| HHX020006753.g | B3 | HHX030013970.g | ERF |
| HHX020010082.g | B3 | HHX030014272.g | ERF |
| HHX040028432.g | B3 | HHX030015220.g | ERF |
| HHX040030453.g | B3 | HHX030015253.g | ERF |
| HHX060018411.g | B3 | HHX040027267.g | ERF |
| HHX040028840.g | ERF | HHX020007607.g | DBB |
| HHX040030007.g | ERF | HHX060016670.g | DBB |
| HHX040030399.g | ERF | HHX070059131.g | CO-like |
| HHX050046359.g | ERF | HHX050048639.g | ARR-B |
| HHX050046360.g | ERF | HHX110052635.g | ARR-B |
| HHX050047307.g | ERF | HHX010035517.g | G2-like |
| HHX050047310.g | ERF | HHX010036786.g | G2-like |
| HHX060016794.g | ERF | HHX020008264.g | G2-like |
| HHX060017989.g | ERF | HHX020008533.g | G2-like |
| HHX070058500.g | ERF | HHX030011083.g | G2-like |
| HHX080003541.g | ERF | HHX030011670.g | G2-like |
| HHX100020447.g | ERF | HHX030015300.g | G2-like |
| HHX100020454.g | ERF | HHX040027966.g | G2-like |
| HHX100020463.g | ERF | HHX040028487.g | G2-like |
| HHX100021823.g | ERF | HHX040028519.g | G2-like |
| HHX100022182.g | ERF | HHX040029329.g | G2-like |
| HHX100022184.g | ERF | HHX050044059.g | G2-like |
| HHX100022187.g | ERF | HHX050045696.g | G2-like |
| HHX100022538.g | ERF | HHX050046419.g | G2-like |
| HHX110050924.g | ERF | HHX050046421.g | G2-like |
| HHX110051123.g | ERF | HHX050046423.g | G2-like |
| HHX110052483.g | ERF | HHX060018873.g | G2-like |
| HHX110052489.g | ERF | HHX070055741.g | G2-like |
| HHX120001615.g | ERF | HHX070058570.g | G2-like |
| HHX120001777.g | ERF | HHX080005080.g | G2-like |
| HHX120002451.g | ERF | HHX080005109.g | G2-like |
| HHX130040081.g | ERF | HHX100021166.g | G2-like |
| HHX140040401.g | ERF | HHX100021437.g | G2-like |
| HHX140040987.g | ERF | HHX100021611.g | G2-like |
| HHX140041017.g | ERF | HHX120001621.g | G2-like |
| HHX150062099.g | ERF | HHX130038646.g | G2-like |
| HHX160062629.g | ERF | HHX130039562.g | G2-like |
| HHX160062849.g | ERF | HHX140041670.g | G2-like |
| HHX160064037.g | ERF | HHX160063636.g | G2-like |
| HHX160064077.g | ERF | HHX180060265.g | G2-like |
| HHX170049811.g | ERF | HHX190053427.g | G2-like |
| HHX170049878.g | ERF | HHX190053800.g | G2-like |
| HHX180060244.g | ERF | HHX190053801.g | G2-like |
| HHX180061150.g | ERF | HHX040028571.g | MIKC_MADS |
| HHX190053357.g | ERF | HHX190053042.g | MIKC_MADS |
| HHX020006893.g | DBB | HHX010032581.g | M-type_MADS |
| HHX020008805.g | M-type_MADS | HHX060017823.g | HB-other |
| HHX020009316.g | M-type_MADS | HHX060017880.g | HB-other |
| HHX020009346.g | M-type_MADS | HHX070055116.g | HB-other |
| HHX040026103.g | M-type_MADS | HHX080004659.g | HB-other |
| HHX040027979.g | M-type_MADS | HHX080004873.g | HB-other |
| HHX050046613.g | M-type_MADS | HHX080005393.g | HB-other |
| HHX050049145.g | M-type_MADS | HHX100021261.g | HB-other |
| HHX070057938.g | M-type_MADS | HHX100021290.g | HB-other |
| HHX070057939.g | M-type_MADS | HHX160063734.g | HB-other |
| HHX080003770.g | M-type_MADS | HHX180061030.g | HB-other |
| HHX080004238.g | M-type_MADS | HHX190053439.g | HB-other |
| HHX080004246.g | M-type_MADS | HHX190053948.g | HB-other |
| HHX080004264.g | M-type_MADS | HHX090025210.g | WOX |
| HHX090025191.g | M-type_MADS | HHX160063145.g | WOX |
| HHX100019943.g | M-type_MADS | HHX070058199.g | WOX |
| HHX100020975.g | M-type_MADS | HHX190054376.g | WOX |
| HHX100021178.g | M-type_MADS | HHX070056872.g | WOX |
| HHX100021632.g | M-type_MADS | HHX060016660.g | WOX |
| HHX100021792.g | M-type_MADS | HHX030013794.g | TALE |
| HHX110051528.g | M-type_MADS | HHX100020672.g | HD-ZIP |
| HHX110051537.g | M-type_MADS | HHX040029441.g | HD-ZIP |
| HHX120002571.g | M-type_MADS | HHX160063802.g | HD-ZIP |
| HHX130040015.g | M-type_MADS | HHX100023203.g | HD-ZIP |
| HHX130040054.g | M-type_MADS | HHX100021091.g | HD-ZIP |
| HHX140040617.g | M-type_MADS | HHX060018891.g | HD-ZIP |
| HHX140040625.g | M-type_MADS | HHX080005405.g | HD-ZIP |
| HHX140041353.g | M-type_MADS | HHX080004893.g | HD-ZIP |
| HHX140041359.g | M-type_MADS | HHX060016797.g | HD-ZIP |
| HHX140041360.g | M-type_MADS | HHX100021418.g | HD-ZIP |
| HHX160062576.g | M-type_MADS | HHX010033998.g | HD-ZIP |
| HHX160063957.g | M-type_MADS | HHX120000696.g | HD-ZIP |
| HHX190053448.g | M-type_MADS | HHX190053946.g | HD-ZIP |
| HHX190053550.g | M-type_MADS | HHX100021843.g | HD-ZIP |
| HHX160063938.g | GRF | HHX190053436.g | HD-ZIP |
| HHX010032547.g | HB-other | HHX040026420.g | HD-ZIP |
| HHX010032552.g | HB-other | HHX010034707.g | HD-ZIP |
| HHX010036153.g | HB-other | HHX120000706.g | HD-ZIP |
| HHX010036309.g | HB-other | HHX180061069.g | HD-ZIP |
| HHX030011932.g | HB-other | HHX040029942.g | HD-ZIP |
| HHX050045966.g | HB-other | HHX180060193.g | TALE |
| HHX060016796.g | HB-other | HHX140041894.g | HD-ZIP |
| HHX100022261.g | HD-ZIP | HHX100021803.g | C3H |
| HHX030016193.g | HD-ZIP | HHX110051799.g | C2H2 |
| HHX050045825.g | HD-ZIP | HHX120000855.g | C3H |
| HHX010033468.g | C2H2 | HHX120001508.g | C2H2 |
| HHX010034257.g | C3H | HHX120001908.g | C3H |
| HHX010034757.g | C3H | HHX120002462.g | C3H |
| HHX010034777.g | C2H2 | HHX120002464.g | C3H |
| HHX010035640.g | C3H | HHX130038624.g | C2H2 |
| HHX010035647.g | C2H2 | HHX130039309.g | C2H2 |
| HHX010036064.g | C2H2 | HHX130039360.g | C2H2 |
| HHX010036168.g | C3H | HHX130039566.g | C2H2 |
| HHX010037675.g | C2H2 | HHX130039945.g | C2H2 |
| HHX020008232.g | LSD | HHX140040434.g | C3H |
| HHX020008295.g | C2H2 | HHX140041036.g | C2H2 |
| HHX020010306.g | C2H2 | HHX140043288.g | C3H |
| HHX030012919.g | C3H | HHX160062677.g | C2H2 |
| HHX030012994.g | C2H2 | HHX160063736.g | C2H2 |
| HHX030014706.g | C2H2 | HHX170049670.g | C2H2 |
| HHX030014707.g | C2H2 | HHX180060256.g | C2H2 |
| HHX030015115.g | C3H | HHX180060871.g | C2H2 |
| HHX040025611.g | C3H | HHX180060905.g | C3H |
| HHX040025615.g | C3H | HHX010034464.g | MYB |
| HHX040027681.g | C3H | HHX010035262.g | MYB |
| HHX040027682.g | C3H | HHX010035854.g | MYB |
| HHX040030050.g | C2H2 | HHX020007110.g | MYB |
| HHX040031244.g | C3H | HHX020009602.g | MYB |
| HHX050045250.g | C2H2 | HHX020010164.g | MYB |
| HHX050045752.g | C2H2 | HHX020010556.g | MYB |
| HHX050047532.g | C3H | HHX030013680.g | MYB |
| HHX050048347.g | C3H | HHX040025776.g | MYB |
| HHX070056846.g | C2H2 | HHX040026691.g | MYB |
| HHX070057106.g | C2H2 | HHX040026700.g | MYB |
| HHX070057511.g | C2H2 | HHX040027210.g | MYB |
| HHX070058592.g | C2H2 | HHX040027580.g | MYB |
| HHX080004871.g | C2H2 | HHX040029369.g | MYB |
| HHX090024782.g | C2H2 | HHX040029445.g | MYB |
| HHX090024799.g | C3H | HHX040029596.g | MYB |
| HHX090024804.g | C3H | HHX050044067.g | MYB |
| HHX100020810.g | C2H2 | HHX050045931.g | MYB |
| HHX100021726.g | LSD | HHX060018846.g | MYB |
| HHX100021777.g | C2H2 | HHX070054855.g | MYB |
| HHX070056879.g | MYB | HHX030012944.g | MYB_related |
| HHX070057325.g | MYB | HHX030013802.g | MYB_related |
| HHX080005421.g | MYB | HHX030013816.g | MYB_related |
| HHX080006553.g | MYB | HHX030014078.g | MYB_related |
| HHX100020580.g | MYB | HHX030015641.g | MYB_related |
| HHX100020811.g | MYB | HHX040027493.g | MYB_related |
| HHX100020812.g | MYB | HHX040027579.g | MYB_related |
| HHX100020830.g | MYB | HHX040029298.g | MYB_related |
| HHX100020833.g | MYB | HHX040029320.g | MYB_related |
| HHX100021174.g | MYB | HHX050044429.g | MYB_related |
| HHX100021353.g | MYB | HHX050044438.g | MYB_related |
| HHX100021481.g | MYB | HHX050044484.g | MYB_related |
| HHX100022009.g | MYB | HHX050045507.g | MYB_related |
| HHX120002153.g | MYB | HHX050045508.g | MYB_related |
| HHX140041536.g | MYB | HHX050046540.g | MYB_related |
| HHX150061166.g | MYB | HHX050046626.g | MYB_related |
| HHX150062023.g | MYB | HHX050046631.g | MYB_related |
| HHX160062669.g | MYB | HHX050048499.g | MYB_related |
| HHX160063048.g | MYB | HHX050048677.g | MYB_related |
| HHX160063931.g | MYB | HHX060016330.g | MYB_related |
| HHX190053077.g | MYB | HHX060016445.g | MYB_related |
| HHX190053412.g | MYB | HHX060018831.g | MYB_related |
| HHX010032439.g | MYB_related | HHX060018832.g | MYB_related |
| HHX010034752.g | MYB_related | HHX060019136.g | MYB_related |
| HHX010035047.g | MYB_related | HHX070054724.g | MYB_related |
| HHX010035258.g | MYB_related | HHX070055716.g | MYB_related |
| HHX010035695.g | MYB_related | HHX070056399.g | MYB_related |
| HHX010036164.g | MYB_related | HHX070056400.g | MYB_related |
| HHX010036171.g | MYB_related | HHX070059573.g | MYB_related |
| HHX010036242.g | MYB_related | HHX080003484.g | MYB_related |
| HHX010036464.g | MYB_related | HHX080005175.g | MYB_related |
| HHX020007317.g | MYB_related | HHX080005857.g | MYB_related |
| HHX020007320.g | MYB_related | HHX100021479.g | MYB_related |
| HHX020007344.g | MYB_related | HHX100021586.g | MYB_related |
| HHX020007355.g | MYB_related | HHX100022291.g | MYB_related |
| HHX030011738.g | MYB_related | HHX100022695.g | MYB_related |
| HHX030012259.g | MYB_related | HHX110050774.g | MYB_related |
| HHX030012504.g | MYB_related | HHX110050776.g | MYB_related |
| HHX030012516.g | MYB_related | HHX110052677.g | MYB_related |
| HHX030012517.g | MYB_related | HHX110052678.g | MYB_related |
| HHX030012546.g | MYB_related | HHX120000753.g | MYB_related |
| HHX120001271.g | MYB_related | HHX140043752.g | MYB_related |
| HHX120001785.g | MYB_related | HHX160063324.g | MYB_related |
| HHX120002037.g | MYB_related | HHX160063515.g | MYB_related |
| HHX120002038.g | MYB_related | HHX180060005.g | MYB_related |
| HHX130039522.g | MYB_related | HHX180060174.g | MYB_related |
| HHX130039879.g | MYB_related | HHX180060846.g | MYB_related |
| HHX130039882.g | MYB_related | HHX190053073.g | MYB_related |
| HHX140040900.g | MYB_related | HHX190053997.g | MYB_related |

## Table S5 Comparing the genome of six species

| Species | Proteins | Clusters | Singletons |
| --- | --- | --- | --- |
| *Amborella trichopoda* | 27,313 | 13,202 | 8,575 |
| *Vitis vinifera* | 26,556 | 13,098 | 7,657 |
| *Liriodendron chinense* | 35,269 | 16,401 | 4,039 |
| *M.champaca* | 64,168 | 24,608 | 33,615 |
| *M.montaua* | 68,392 | 25,940 | 36,883 |
| *Magnolia biondii* | 41,181 | 19,229 | 10,231 |

## Table S6 KEGG (Kyoto Encyclopedia of Genes and Genomes) analysis of the specific gene families

**A**

| **MM** | | | | |
| --- | --- | --- | --- | --- |
| Term | ID | Input number | P-Value | Corrected P-Value |
| Metabolic pathways | ath01100 | 2053 | 6.07E-119 | 3.78E-116 |
| Biosynthesis of amino acids | ath01230 | 234 | 1.28E-14 | 1.20E-12 |
| Carbon metabolism | ath01200 | 235 | 2.89E-12 | 2.08E-10 |
| Protein processing in endoplasmic reticulum | ath04141 | 182 | 6.89E-10 | 3.79E-08 |
| Glycolysis / Gluconeogenesis | ath00010 | 112 | 2.59E-08 | 1.27E-06 |
| Endocytosis | ath04144 | 128 | 6.90E-08 | 3.00E-06 |
| Amino sugar and nucleotide sugar metabolism | ath00520 | 112 | 1.56E-06 | 6.06E-05 |
| Pyruvate metabolism | ath00620 | 82 | 2.44E-06 | 9.12E-05 |
| Glycerophospholipid metabolism | ath00564 | 88 | 4.10E-06 | 0.0001448 |
| MAPK signaling pathway - plant | ath04016 | 109 | 9.80E-06 | 0.0003393 |
| Glyoxylate and dicarboxylate metabolism | ath00630 | 72 | 2.00E-05 | 0.0006329 |
| RNA transport | ath03013 | 128 | 2.87E-05 | 0.0008784 |
| Spliceosome | ath03040 | 140 | 3.09E-05 | 0.0009311 |
| Ribosome biogenesis in eukaryotes | ath03008 | 85 | 3.87E-05 | 0.0011132 |
| Ubiquitin mediated proteolysis | ath04120 | 107 | 0.0001239 | 0.0030879 |
| Arginine and proline metabolism | ath00330 | 50 | 0.0003284 | 0.0075772 |
| Pentose and glucuronate interconversions | ath00040 | 74 | 0.000706 | 0.0136042 |
| Tyrosine metabolism | ath00350 | 39 | 0.0007451 | 0.014157 |
| Citrate cycle (TCA cycle) | ath00020 | 53 | 0.0010189 | 0.0179654 |
| Pyrimidine metabolism | ath00240 | 49 | 0.0011309 | 0.0197542 |
| Glycine, serine and threonine metabolism | ath00260 | 55 | 0.0023851 | 0.0384296 |
| Peroxisome | ath04146 | 65 | 0.0024334 | 0.0388713 |
| 2-Oxocarboxylic acid metabolism | ath01210 | 57 | 0.0027316 | 0.0412752 |
| Carbon fixation in photosynthetic organisms | ath00710 | 54 | 0.0027524 | 0.0412752 |
| Arginine biosynthesis | ath00220 | 33 | 0.0035231 | 0.0509427 |
| Cysteine and methionine metabolism | ath00270 | 82 | 0.0049412 | 0.0664399 |
| Fructose and mannose metabolism | ath00051 | 49 | 0.0056123 | 0.0743931 |
| Propanoate metabolism | ath00640 | 36 | 0.0063881 | 0.079596 |
| Nucleotide excision repair | ath03420 | 51 | 0.0076758 | 0.0913504 |
| Aminoacyl-tRNA biosynthesis | ath00970 | 77 | 0.0077225 | 0.0913504 |
| Pentose phosphate pathway | ath00030 | 44 | 0.0093084 | 0.106733 |

**B**

| **MC** | | | | |
| --- | --- | --- | --- | --- |
| Term | ID | Input number | P-Value | Corrected P-Value |
| Biosynthesis of secondary metabolites | ath01110 | 876 | 5.08E-77 | 2.13E-74 |
| Metabolic pathways | ath01100 | 1390 | 5.23E-71 | 1.57E-68 |
| Starch and sucrose metabolism | ath00500 | 142 | 2.12E-15 | 1.31E-13 |
| RNA degradation | ath03018 | 97 | 5.83E-11 | 2.72E-09 |
| Endocytosis | ath04144 | 110 | 3.95E-10 | 1.69E-08 |
| Glycolysis / Gluconeogenesis | ath00010 | 95 | 5.04E-10 | 2.12E-08 |
| Glutathione metabolism | ath00480 | 87 | 6.82E-10 | 2.75E-08 |
| Spliceosome | ath03040 | 129 | 4.33E-09 | 1.56E-07 |
| Biosynthesis of amino acids | ath01230 | 147 | 2.28E-07 | 6.82E-06 |
| Peroxisome | ath04146 | 66 | 1.35E-06 | 3.68E-05 |
| Fatty acid metabolism | ath01212 | 56 | 2.00E-06 | 5.11E-05 |
| Fatty acid biosynthesis | ath00061 | 39 | 1.24E-05 | 0.0002485 |
| MAPK signaling pathway - plant | ath04016 | 84 | 1.34E-05 | 0.0002643 |
| Tryptophan metabolism | ath00380 | 47 | 2.40E-05 | 0.0004532 |
| Carotenoid biosynthesis | ath00906 | 29 | 4.97E-05 | 0.0008919 |
| DNA replication | ath03030 | 39 | 0.0001211 | 0.0018962 |
| Carbon metabolism | ath01200 | 138 | 0.0001279 | 0.0019738 |
| Phenylalanine metabolism | ath00360 | 29 | 0.0001573 | 0.0023746 |
| Valine, leucine and isoleucine degradation | ath00280 | 39 | 0.0001617 | 0.0024229 |
| Glyoxylate and dicarboxylate metabolism | ath00630 | 52 | 0.0001841 | 0.0026826 |
| Glycerolipid metabolism | ath00561 | 43 | 0.0002127 | 0.0030354 |
| Galactose metabolism | ath00052 | 41 | 0.0002791 | 0.0038528 |
| Amino sugar and nucleotide sugar metabolism | ath00520 | 75 | 0.0003041 | 0.0041435 |
| Ribosome biogenesis in eukaryotes | ath03008 | 61 | 0.0003868 | 0.0050721 |
| Base excision repair | ath03410 | 33 | 0.000477 | 0.0060783 |
| Thiamine metabolism | ath00730 | 21 | 0.0005313 | 0.0066349 |
| Pyrimidine metabolism | ath00240 | 39 | 0.0007766 | 0.0094185 |
| Protein export | ath03060 | 36 | 0.0016785 | 0.018632 |
| Ribosome | ath03010 | 164 | 0.0019306 | 0.0208615 |
| Butanoate metabolism | ath00650 | 18 | 0.0019489 | 0.0208615 |
| Other glycan degradation | ath00511 | 18 | 0.0019489 | 0.0208615 |
| Glycerophospholipid metabolism | ath00564 | 55 | 0.0020908 | 0.0222226 |
| RNA transport | ath03013 | 86 | 0.0023666 | 0.0243585 |
| Diterpenoid biosynthesis | ath00904 | 19 | 0.0029866 | 0.0294174 |
| Biosynthesis of unsaturated fatty acids | ath01040 | 20 | 0.0031066 | 0.0301746 |
| Autophagy - other | ath04136 | 28 | 0.0032026 | 0.0308213 |
| Selenocompound metabolism | ath00450 | 16 | 0.0051783 | 0.046828 |
| Taurine and hypotaurine metabolism | ath00430 | 13 | 0.0089952 | 0.0745922 |
| Protein processing in endoplasmic reticulum | ath04141 | 97 | 0.0094721 | 0.077017 |
| Biotin metabolism | ath00780 | 14 | 0.009521 | 0.077017 |

## Table S7 comparative evolutionary genomic analyses

|  | *Liriodendron chinense* | *Magnolia biondii* | *Picea abies* | *Piper nigrum* | *Aristolochia debilis* | *Michelia champaca* | *Selaginella tamariscina* | *Chimonanthus praecox* | *Arabidopsis thaliana* | *Cinnamomum kanehirae* | *Vitis vinifera* | *Oryza sativa* | *Nymphaea tetragona* | *Michelia montaua* | *Amborella trichopoda* | *Elaeis guineensis* | *Zea mays* |
| --- | --- | --- | --- | --- | --- | --- | --- | --- | --- | --- | --- | --- | --- | --- | --- | --- | --- |
| Number of genes | 35269 | 41181 | 66632 | 63466 | 21855 | 64168 | 45247 | 23559 | 48265 | 26531 | 41208 | 28555 | 31475 | 68392 | 31494 | 43551 | 58409 |
| Number of genes in orthogroups | 34567 | 38817 | 56524 | 53449 | 19228 | 49572 | 42260 | 21917 | 46046 | 25328 | 40561 | 24858 | 27475 | 54662 | 31035 | 42784 | 55384 |
| Number of unassigned genes | 702 | 2364 | 10108 | 10017 | 2627 | 14596 | 2987 | 1642 | 2219 | 1203 | 647 | 3697 | 4000 | 13730 | 459 | 767 | 3025 |
| Percentage of genes in orthogroups | 98 | 94.3 | 84.8 | 84.2 | 88 | 77.3 | 93.4 | 93 | 95.4 | 95.5 | 98.4 | 87.1 | 87.3 | 79.9 | 98.5 | 98.2 | 94.8 |
| Percentage of unassigned genes | 2 | 5.7 | 15.2 | 15.8 | 12 | 22.7 | 6.6 | 7 | 4.6 | 4.5 | 1.6 | 12.9 | 12.7 | 20.1 | 1.5 | 1.8 | 5.2 |
| Number of orthogroups containing species | 11126 | 14301 | 14616 | 14347 | 10238 | 19458 | 11483 | 10303 | 11398 | 10233 | 10896 | 11318 | 10891 | 20389 | 10196 | 10449 | 12610 |
| Percentage of orthogroups containing species | 24.4 | 31.4 | 32.1 | 31.5 | 22.5 | 42.7 | 25.2 | 22.6 | 25 | 22.5 | 23.9 | 24.8 | 23.9 | 44.8 | 22.4 | 22.9 | 27.7 |
| Number of species-specific orthogroups | 332 | 380 | 3162 | 3493 | 266 | 1402 | 3601 | 148 | 1681 | 255 | 623 | 510 | 789 | 1166 | 342 | 376 | 1665 |
| Number of genes in species-specific orthogroups | 6303 | 1569 | 18101 | 14780 | 1373 | 4171 | 13056 | 445 | 7630 | 1202 | 4316 | 1750 | 5042 | 3691 | 1317 | 1487 | 8475 |
| Percentage of genes in species-specific orthogroups | 17.9 | 3.8 | 27.2 | 23.3 | 6.3 | 6.5 | 28.9 | 1.9 | 15.8 | 4.5 | 10.5 | 6.1 | 16 | 5.4 | 4.2 | 3.4 | 14.5 |

## Table S8 Statistics of methylation sites in the two organs of flower and leaf

|  | *Michelia champaca_*flower | *Michelia champaca_*leaf | *Michelia montaua_*flower | *Michelia montaua_*leaf |
| --- | --- | --- | --- | --- |
| CHH | 321,211,379 | 302,713,120 | 53,156,789 | 56,094,290 |
| CG | 317,284,791 | 271,316,538 | 52,479,090 | 55,316,685 |
| CHG | 257,778,276 | 162,325,854 | 51,798,809 | 54,198,256 |

## Table S9 Genes in the differentially methylated regions

**A.**

| HHX010031995.g | HHX010035643.g | HHX020009526.g | HHX030014086.g |
| --- | --- | --- | --- |
| HHX010032076.g | HHX010035752.g | HHX020009651.g | HHX030014125.g |
| HHX010032195.g | HHX010035799.g | HHX020009654.g | HHX030014129.g |
| HHX010032415.g | HHX010035950.g | HHX020009718.g | HHX030014130.g |
| HHX010032978.g | HHX010036096.g | HHX020010062.g | HHX030014436.g |
| HHX010033148.g | HHX010036348.g | HHX020010809.g | HHX030014788.g |
| HHX010034179.g | HHX010036677.g | HHX020010938.g | HHX030015190.g |
| HHX010034181.g | HHX010036991.g | HHX030011462.g | HHX030015328.g |
| HHX010034206.g | HHX010037581.g | HHX030011689.g | HHX030015410.g |
| HHX010034228.g | HHX020006939.g | HHX030011818.g | HHX030015729.g |
| HHX010034244.g | HHX020007217.g | HHX030011828.g | HHX040025233.g |
| HHX010034291.g | HHX020007389.g | HHX030011852.g | HHX040025557.g |
| HHX010034292.g | HHX020007438.g | HHX030012055.g | HHX040025710.g |
| HHX010034369.g | HHX020007679.g | HHX030012302.g | HHX040025791.g |
| HHX010034493.g | HHX020007782.g | HHX030012665.g | HHX040025973.g |
| HHX010034556.g | HHX020007867.g | HHX030012723.g | HHX040029412.g |
| HHX010034821.g | HHX020008003.g | HHX030012743.g | HHX040029463.g |
| HHX010034897.g | HHX020008009.g | HHX030012786.g | HHX040029610.g |
| HHX010035021.g | HHX020008273.g | HHX030013020.g | HHX040029712.g |
| HHX010035202.g | HHX020008461.g | HHX030013021.g | HHX040029758.g |
| HHX010035226.g | HHX020008463.g | HHX030013034.g | HHX040029759.g |
| HHX010035370.g | HHX020008557.g | HHX030013131.g | HHX040029828.g |
| HHX010035407.g | HHX020008956.g | HHX030013435.g | HHX040029879.g |
| HHX010035546.g | HHX020008974.g | HHX030013638.g | HHX040029886.g |
| HHX010035548.g | HHX020009088.g | HHX030013812.g | HHX040029895.g |
| HHX010035592.g | HHX020009167.g | HHX030013897.g | HHX040029970.g |
| HHX040030151.g | HHX050048507.g | HHX070057906.g | HHX100021195.g |
| HHX040030283.g | HHX050048535.g | HHX070057997.g | HHX100021365.g |
| HHX040030382.g | HHX050048667.g | HHX070058664.g | HHX100021377.g |
| HHX040030393.g | HHX050048668.g | HHX070058947.g | HHX100021763.g |
| HHX040030398.g | HHX050049012.g | HHX070058951.g | HHX100021817.g |
| HHX040030408.g | HHX060016277.g | HHX070059017.g | HHX100021878.g |
| HHX040030665.g | HHX060016427.g | HHX070059412.g | HHX100021900.g |
| HHX040030666.g | HHX060016656.g | HHX070059530.g | HHX100021901.g |
| HHX040030795.g | HHX060017747.g | HHX070059537.g | HHX100022078.g |
| HHX040031132.g | HHX060017861.g | HHX080002967.g | HHX100022166.g |
| HHX040031443.g | HHX060018299.g | HHX080003007.g | HHX100022202.g |
| HHX040031555.g | HHX060018416.g | HHX080003250.g | HHX100022208.g |
| HHX040031590.g | HHX060018450.g | HHX080003802.g | HHX100022235.g |
| HHX040031652.g | HHX060018585.g | HHX080003910.g | HHX100022239.g |
| HHX050044240.g | HHX060018706.g | HHX080004159.g | HHX100022520.g |
| HHX050044310.g | HHX060018711.g | HHX080004170.g | HHX100023030.g |
| HHX050044354.g | HHX060018766.g | HHX080004805.g | HHX100023045.g |
| HHX050044746.g | HHX060018991.g | HHX080004872.g | HHX100023063.g |
| HHX050044920.g | HHX060019122.g | HHX080005186.g | HHX100023410.g |
| HHX050045424.g | HHX060019207.g | HHX080005452.g | HHX100023665.g |
| HHX050045670.g | HHX060019272.g | HHX080005683.g | HHX100023730.g |
| HHX050045935.g | HHX070054450.g | HHX080005713.g | HHX100023779.g |
| HHX050046028.g | HHX070054983.g | HHX080005903.g | HHX110050368.g |
| HHX050046139.g | HHX070055161.g | HHX080005998.g | HHX110050526.g |
| HHX050046181.g | HHX070055338.g | HHX080006040.g | HHX110050736.g |
| HHX050046311.g | HHX070055354.g | HHX080006063.g | HHX110050785.g |
| HHX050046427.g | HHX070055556.g | HHX080006277.g | HHX110050787.g |
| HHX050046491.g | HHX070055622.g | HHX080006375.g | HHX110050947.g |
| HHX050046492.g | HHX070055725.g | HHX080006460.g | HHX110051282.g |
| HHX050046513.g | HHX070055881.g | HHX080006572.g | HHX110052426.g |
| HHX050046768.g | HHX070056135.g | HHX090023944.g | HHX110052714.g |
| HHX050046769.g | HHX070056245.g | HHX100019881.g | HHX110052797.g |
| HHX050046802.g | HHX070056493.g | HHX100020001.g | HHX110052875.g |
| HHX050046892.g | HHX070056675.g | HHX100020212.g | HHX110052987.g |
| HHX050046896.g | HHX070056790.g | HHX100020472.g | HHX120000312.g |
| HHX050047025.g | HHX070056791.g | HHX100020492.g | HHX120001133.g |
| HHX050047129.g | HHX070056953.g | HHX100020817.g | HHX120001594.g |
| HHX050047785.g | HHX070057226.g | HHX100020834.g | HHX120001655.g |
| HHX050048359.g | HHX070057329.g | HHX100020903.g | HHX120001656.g |
| HHX050048434.g | HHX070057493.g | HHX100021054.g | HHX120001922.g |
| HHX050048454.g | HHX070057710.g | HHX100021194.g | HHX120001936.g |
| HHX120002114.g | HHX140041445.g | HHX160063483.g | HHX190053608.g |
| HHX120002185.g | HHX140041726.g | HHX160063874.g | HHX190053681.g |
| HHX120002187.g | HHX140042308.g | HHX160063937.g | HHX190054293.g |
| HHX120002202.g | HHX140042409.g | HHX160063941.g |  |
| HHX120002337.g | HHX140042746.g | HHX160064043.g |  |
| HHX130039534.g | HHX140042845.g | HHX170049610.g |  |
| HHX130039909.g | HHX140043117.g | HHX170050175.g |  |
| HHX130039910.g | HHX140043377.g | HHX170050203.g |  |
| HHX130040019.g | HHX140043549.g | HHX170050218.g |  |
| HHX130040061.g | HHX140043581.g | HHX170050231.g |  |
| HHX130040123.g | HHX140043906.g | HHX170050323.g |  |
| HHX140040513.g | HHX140043920.g | HHX170050325.g |  |
| HHX140040939.g | HHX150061350.g | HHX180060064.g |  |
| HHX140040989.g | HHX150062162.g | HHX180060597.g |  |
| HHX140041086.g | HHX150062374.g | HHX180060892.g |  |
| HHX140041104.g | HHX150062387.g | HHX180060895.g |  |
| HHX140041195.g | HHX150062423.g | HHX180060939.g |  |
| HHX140041402.g | HHX160062558.g | HHX190053078.g |  |
| HHX140041440.g | HHX160062597.g | HHX190053359.g |  |

**B.**

| SSHX010062108.g | SSHX010066750.g | SSHX020048401.g | SSHX050035400.g |
| --- | --- | --- | --- |
| SSHX010062206.g | SSHX010066845.g | SSHX020048632.g | SSHX050035477.g |
| SSHX010062363.g | SSHX010066949.g | SSHX030033067.g | SSHX050035516.g |
| SSHX010062632.g | SSHX010067381.g | SSHX040003599.g | SSHX050035602.g |
| SSHX010062673.g | SSHX010067383.g | SSHX040003823.g | SSHX050035941.g |
| SSHX010062861.g | SSHX010067424.g | SSHX040004200.g | SSHX050036027.g |
| SSHX010063085.g | SSHX010067430.g | SSHX040004224.g | SSHX050036242.g |
| SSHX010063087.g | SSHX010067516.g | SSHX040004278.g | SSHX050036346.g |
| SSHX010063265.g | SSHX010067583.g | SSHX040004685.g | SSHX050036422.g |
| SSHX010063266.g | SSHX010067714.g | SSHX040005191.g | SSHX050036472.g |
| SSHX010063267.g | SSHX010067735.g | SSHX040005239.g | SSHX050036499.g |
| SSHX010063357.g | SSHX010067736.g | SSHX040005251.g | SSHX050036500.g |
| SSHX010063529.g | SSHX010067766.g | SSHX040005376.g | SSHX050036510.g |
| SSHX010063799.g | SSHX010067786.g | SSHX040005618.g | SSHX050036624.g |
| SSHX010064115.g | SSHX010068063.g | SSHX040005679.g | SSHX050036747.g |
| SSHX010064165.g | SSHX010068092.g | SSHX040006043.g | SSHX050036749.g |
| SSHX010064409.g | SSHX020044345.g | SSHX040006191.g | SSHX050036755.g |
| SSHX010064527.g | SSHX020044444.g | SSHX040006622.g | SSHX050036772.g |
| SSHX010064546.g | SSHX020044581.g | SSHX040007088.g | SSHX050036804.g |
| SSHX010064568.g | SSHX020044638.g | SSHX040007819.g | SSHX050036918.g |
| SSHX010064620.g | SSHX020044685.g | SSHX040008075.g | SSHX050037059.g |
| SSHX010064739.g | SSHX020044814.g | SSHX040008153.g | SSHX050037223.g |
| SSHX010064782.g | SSHX020044815.g | SSHX040008184.g | SSHX060049126.g |
| SSHX010064848.g | SSHX020044884.g | SSHX040008259.g | SSHX060049234.g |
| SSHX010064906.g | SSHX020045269.g | SSHX050033141.g | SSHX060049276.g |
| SSHX010065056.g | SSHX020045550.g | SSHX050033463.g | SSHX060049287.g |
| SSHX010065112.g | SSHX020045638.g | SSHX050033494.g | SSHX060049297.g |
| SSHX010065279.g | SSHX020045923.g | SSHX050033753.g | SSHX060049388.g |
| SSHX010065372.g | SSHX020045946.g | SSHX050033966.g | SSHX060049401.g |
| SSHX010065414.g | SSHX020046061.g | SSHX050034094.g | SSHX060049506.g |
| SSHX010065427.g | SSHX020046087.g | SSHX050034329.g | SSHX060049511.g |
| SSHX010065493.g | SSHX020046325.g | SSHX050034331.g | SSHX060049586.g |
| SSHX010065537.g | SSHX020046470.g | SSHX050034730.g | SSHX060049684.g |
| SSHX010065563.g | SSHX020046516.g | SSHX050035037.g | SSHX060049686.g |
| SSHX010065642.g | SSHX020046569.g | SSHX050035056.g | SSHX060049687.g |
| SSHX010065808.g | SSHX020046826.g | SSHX050035135.g | SSHX060049750.g |
| SSHX010066337.g | SSHX020046859.g | SSHX050035141.g | SSHX060049899.g |
| SSHX010066578.g | SSHX020048054.g | SSHX050035229.g | SSHX060050022.g |
| SSHX010066613.g | SSHX020048154.g | SSHX050035387.g | SSHX060050160.g |
| SSHX060050425.g | SSHX080037852.g | SSHX090021847.g | SSHX100057725.g |
| SSHX060050566.g | SSHX080037968.g | SSHX090022135.g | SSHX100058509.g |
| SSHX060050799.g | SSHX080038035.g | SSHX090022145.g | SSHX100058585.g |
| SSHX060050966.g | SSHX080038175.g | SSHX090022193.g | SSHX100058627.g |
| SSHX060051133.g | SSHX080038460.g | SSHX090022220.g | SSHX100058798.g |
| SSHX060051253.g | SSHX080038584.g | SSHX090022315.g | SSHX100059093.g |
| SSHX060051661.g | SSHX080038677.g | SSHX090022698.g | SSHX110025383.g |
| SSHX060052089.g | SSHX080039174.g | SSHX090022911.g | SSHX110025876.g |
| SSHX060052123.g | SSHX080039229.g | SSHX090022916.g | SSHX110025888.g |
| SSHX060052327.g | SSHX080039401.g | SSHX090022940.g | SSHX110026058.g |
| SSHX060052429.g | SSHX080039621.g | SSHX090023055.g | SSHX110026453.g |
| SSHX060052456.g | SSHX080039743.g | SSHX090023096.g | SSHX110026465.g |
| SSHX060052472.g | SSHX080039962.g | SSHX090023199.g | SSHX110026584.g |
| SSHX060052761.g | SSHX080040058.g | SSHX090023209.g | SSHX110026597.g |
| SSHX060052783.g | SSHX080040134.g | SSHX090023349.g | SSHX110026745.g |
| SSHX060052821.g | SSHX080040149.g | SSHX090023641.g | SSHX110026890.g |
| SSHX070052881.g | SSHX080040351.g | SSHX090023806.g | SSHX110026940.g |
| SSHX070052924.g | SSHX080040435.g | SSHX090024514.g | SSHX110026983.g |
| SSHX070052927.g | SSHX080040496.g | SSHX090024587.g | SSHX110027022.g |
| SSHX070052964.g | SSHX080040759.g | SSHX090024747.g | SSHX110027119.g |
| SSHX070053018.g | SSHX080040769.g | SSHX090024757.g | SSHX110027296.g |
| SSHX070053044.g | SSHX080040774.g | SSHX090024772.g | SSHX110027487.g |
| SSHX070053171.g | SSHX080040909.g | SSHX090024784.g | SSHX110027732.g |
| SSHX070053232.g | SSHX080041007.g | SSHX090024854.g | SSHX110027854.g |
| SSHX070053268.g | SSHX080041042.g | SSHX090024888.g | SSHX110027857.g |
| SSHX070053451.g | SSHX080041109.g | SSHX090024959.g | SSHX110027991.g |
| SSHX070053827.g | SSHX080041120.g | SSHX090024960.g | SSHX110028070.g |
| SSHX070054021.g | SSHX080041139.g | SSHX090024963.g | SSHX110028120.g |
| SSHX070054125.g | SSHX080041290.g | SSHX090025227.g | SSHX110028168.g |
| SSHX070054514.g | SSHX080041304.g | SSHX090025285.g | SSHX110028233.g |
| SSHX070054890.g | SSHX080041392.g | SSHX100055941.g | SSHX110028246.g |
| SSHX070054927.g | SSHX080041403.g | SSHX100055967.g | SSHX120000533.g |
| SSHX070055058.g | SSHX080041416.g | SSHX100055985.g | SSHX120000549.g |
| SSHX070055215.g | SSHX080041425.g | SSHX100056245.g | SSHX120000794.g |
| SSHX070055750.g | SSHX090021385.g | SSHX100056308.g | SSHX120000827.g |
| SSHX070055795.g | SSHX090021405.g | SSHX100056638.g | SSHX120000868.g |
| SSHX070055803.g | SSHX090021594.g | SSHX100056816.g | SSHX120001000.g |
| SSHX080037600.g | SSHX090021634.g | SSHX100056829.g | SSHX120001231.g |
| SSHX080037679.g | SSHX090021635.g | SSHX100056942.g | SSHX120001407.g |
| SSHX080037719.g | SSHX090021681.g | SSHX100057477.g | SSHX120001511.g |
| SSHX080037723.g | SSHX090021691.g | SSHX100057681.g | SSHX120001525.g |
| SSHX120001561.g | SSHX130020423.g | SSHX170042618.g | SSHX190013073.g |
| SSHX120001923.g | SSHX130020544.g | SSHX170042676.g | SSHX190013170.g |
| SSHX120001982.g | SSHX130020613.g | SSHX170042898.g | SSHX190013272.g |
| SSHX120001986.g | SSHX130020675.g | SSHX170042927.g | SSHX190013300.g |
| SSHX120002053.g | SSHX130021183.g | SSHX170042975.g | SSHX190013722.g |
| SSHX120002068.g | SSHX140008625.g | SSHX170043046.g | SSHX190013956.g |
| SSHX120002112.g | SSHX150017839.g | SSHX170043207.g |  |
| SSHX120002249.g | SSHX150018095.g | SSHX170043270.g |  |
| SSHX120002357.g | SSHX150018233.g | SSHX170043313.g |  |
| SSHX120002597.g | SSHX150018401.g | SSHX170043357.g |  |
| SSHX120002660.g | SSHX150018462.g | SSHX170043371.g |  |
| SSHX120002689.g | SSHX160059665.g | SSHX170043710.g |  |
| SSHX120002832.g | SSHX160059763.g | SSHX170043728.g |  |
| SSHX120002835.g | SSHX160059904.g | SSHX170043847.g |  |
| SSHX120002840.g | SSHX160059927.g | SSHX170043873.g |  |
| SSHX120002867.g | SSHX160059977.g | SSHX170043917.g |  |
| SSHX120002892.g | SSHX160060088.g | SSHX170043979.g |  |
| SSHX120002923.g | SSHX160060205.g | SSHX180014256.g |  |
| SSHX120003052.g | SSHX160060390.g | SSHX180014488.g |  |
| SSHX120003134.g | SSHX160060559.g | SSHX180014703.g |  |
| SSHX130018625.g | SSHX160060940.g | SSHX180014754.g |  |
| SSHX130018633.g | SSHX160060956.g | SSHX180014763.g |  |
| SSHX130018734.g | SSHX160061249.g | SSHX180014776.g |  |
| SSHX130018743.g | SSHX160061322.g | SSHX180014840.g |  |
| SSHX130018912.g | SSHX160061397.g | SSHX180015291.g |  |
| SSHX130019015.g | SSHX160061579.g | SSHX180015396.g |  |
| SSHX130019178.g | SSHX160061600.g | SSHX180015449.g |  |
| SSHX130019189.g | SSHX160061610.g | SSHX180015774.g |  |
| SSHX130019352.g | SSHX160061733.g | SSHX180015924.g |  |
| SSHX130019406.g | SSHX160061845.g | SSHX190012142.g |  |
| SSHX130019407.g | SSHX170041536.g | SSHX190012195.g |  |
| SSHX130019416.g | SSHX170041575.g | SSHX190012374.g |  |
| SSHX130019577.g | SSHX170041627.g | SSHX190012499.g |  |
| SSHX130019618.g | SSHX170042030.g | SSHX190012637.g |  |
| SSHX130019640.g | SSHX170042102.g | SSHX190012646.g |  |
| SSHX130019683.g | SSHX170042166.g | SSHX190012681.g |  |
| SSHX130019860.g | SSHX170042169.g | SSHX190012743.g |  |
| SSHX130019952.g | SSHX170042264.g | SSHX190012789.g |  |
| SSHX130020058.g | SSHX170042318.g | SSHX190012790.g |  |
| SSHX130020170.g | SSHX170042425.g | SSHX190012915.g |  |
| SSHX130020374.g | SSHX170042508.g | SSHX190013003.g |  |

## Table S10 These genes in the differentially methylated regions were annotated using KEGG and Gene Ontology (GO) analysis.

**A**

| ***Michelia champaca* kegg** | | | | |
| --- | --- | --- | --- | --- |
| Term | ID | Input number | P-Value | Corrected P-Value |
| Biosynthesis of secondary metabolites | atr01110 | 34 | 2.75E-06 | 0.000223 |
| Biosynthesis of amino acids | atr01230 | 12 | 7.12E-05 | 0.0025757 |
| Metabolic pathways | atr01100 | 50 | 9.54E-05 | 0.0025757 |
| Glycolysis / Gluconeogenesis | atr00010 | 7 | 0.001030446 | 0.0208665 |
| Tropane, piperidine and pyridine alkaloid biosynthesis | atr00960 | 3 | 0.00417289 | 0.0676008 |
| Diterpenoid biosynthesis | atr00904 | 3 | 0.005429105 | 0.0732929 |
| 2-Oxocarboxylic acid metabolism | atr01210 | 4 | 0.007333061 | 0.084854 |
| Arginine biosynthesis | atr00220 | 3 | 0.011478882 | 0.0925108 |
| Linoleic acid metabolism | atr00591 | 2 | 0.01238852 | 0.0925108 |
| ABC transporters | atr02010 | 3 | 0.012563199 | 0.0925108 |
| Phenylalanine metabolism | atr00360 | 3 | 0.012563199 | 0.0925108 |

**B**

| ***Michelia montaua* kegg** | | | | |
| --- | --- | --- | --- | --- |
| #Term | ID | Input number | P-Value | Corrected P-Value |
| Biosynthesis of secondary metabolites | atr01110 | 42 | 0.0001019 | 0.0079214 |
| Metabolic pathways | atr01100 | 71 | 0.0001704 | 0.0079214 |
| RNA degradation | atr03018 | 9 | 0.0007385 | 0.0228941 |
| Galactose metabolism | atr00052 | 6 | 0.0011485 | 0.0258127 |
| Biosynthesis of amino acids | atr01230 | 13 | 0.0013878 | 0.0258127 |
| Other glycan degradation | atr00511 | 4 | 0.0031326 | 0.0485557 |
| Glycolysis / Gluconeogenesis | atr00010 | 8 | 0.0036594 | 0.0486182 |
| Carbon metabolism | atr01200 | 12 | 0.0068657 | 0.0798137 |
| ABC transporters | atr02010 | 4 | 0.0077698 | 0.0802879 |

**C**

| ***Michelia champaca* GO** | | | | |
| --- | --- | --- | --- | --- |
| #Term | ID | Input number | P-Value | Corrected P-Value |
| cytoplasm | GO:0005737 | 65 | 2.83E-07 | 7.17E-05 |
| cytosol | GO:0005829 | 53 | 4.60E-07 | 8.74E-05 |
| plasma membrane | GO:0005886 | 51 | 1.46E-06 | 0.0002222 |
| magnesium ion binding | GO:0000287 | 8 | 8.24E-06 | 0.0008655 |
| chloroplast | GO:0009507 | 64 | 9.11E-06 | 0.0008655 |
| response to herbivore | GO:0080027 | 4 | 1.39E-05 | 0.0011773 |
| carbohydrate metabolic process | GO:0005975 | 12 | 3.22E-05 | 0.0024457 |
| ATPase-coupled transmembrane transporter activity | GO:0042626 | 7 | 4.09E-05 | 0.0028289 |
| auxin influx transmembrane transporter activity | GO:0010328 | 3 | 4.84E-05 | 0.0030639 |
| response to jasmonic acid | GO:0009753 | 7 | 5.46E-05 | 0.0031891 |
| ATPase activity | GO:0016887 | 10 | 7.65E-05 | 0.0041504 |
| terpene synthase activity | GO:0010333 | 4 | 8.73E-05 | 0.0041935 |
| plastid | GO:0009536 | 19 | 0.000114707 | 0.0051281 |
| plasmodesma | GO:0009506 | 19 | 0.000174115 | 0.0070516 |
| transmembrane transport | GO:0055085 | 12 | 0.00017629 | 0.0070516 |
| plastid organization | GO:0009657 | 3 | 0.000252365 | 0.0095899 |

**D**

| ***Michelia maudiae* GO** | | | | |
| --- | --- | --- | --- | --- |
| #Term | ID | Input number | P-Value | Corrected P-Value |
| plasma membrane | GO:0005886 | 108 | 2.24E-19 | 2.21E-16 |
| cytosol | GO:0005829 | 87 | 3.35E-10 | 1.65E-07 |
| response to jasmonic acid | GO:0009753 | 14 | 1.79E-09 | 4.42E-07 |
| transmembrane transport | GO:0055085 | 23 | 2.62E-08 | 4.31E-06 |
| protein binding | GO:0005515 | 84 | 4.25E-08 | 5.98E-06 |
| cytoplasm | GO:0005737 | 94 | 9.93E-07 | 0.0001224 |
| ATP binding | GO:0005524 | 27 | 1.57E-06 | 0.0001715 |
| chloroplast | GO:0009507 | 97 | 5.34E-06 | 0.0005264 |
| response to wounding | GO:0009611 | 13 | 1.22E-05 | 0.0010957 |
| efflux transmembrane transporter activity | GO:0015562 | 4 | 4.60E-05 | 0.0037771 |
| ribonucleoprotein complex | GO:1990904 | 6 | 0.0001072 | 0.0070454 |
| transmembrane transporter activity | GO:0022857 | 14 | 0.0001259 | 0.0077563 |
| plasmodesma | GO:0009506 | 27 | 0.0001371 | 0.007949 |
| sesquiterpenoid biosynthetic process | GO:0016106 | 3 | 0.0001453 | 0.0079592 |
| ATPase-coupled transmembrane transporter activity | GO:0042626 | 8 | 0.0001648 | 0.0085519 |
| protein phosphorylation | GO:0006468 | 25 | 0.0001854 | 0.0088533 |
| oxylipin biosynthetic process | GO:0031408 | 4 | 0.0001886 | 0.0088533 |
| abscisic acid transport | GO:0080168 | 3 | 0.0002157 | 0.0096665 |

## Table S11 Genome-wide distribution of DMRs

**A.**

| ***Michelia champaca*_CG** | | | | |
| --- | --- | --- | --- | --- |
| hhx01 | 1964528 | 1975241 | transcript | HHX010031863.g |
| hhx01 | 2347500 | 2351685 | transcript | HHX010031895.g |
| hhx01 | 3331960 | 3335259 | transcript | HHX010031995.g |
| hhx01 | 3518526 | 3526203 | transcript | HHX010032011.g |
| hhx01 | 6396942 | 6404192 | transcript | HHX010032195.g |
| hhx01 | 16414424 | 16415726 | transcript | HHX010032685.g |
| hhx01 | 23669503 | 23671278 | transcript | HHX010032932.g |
| hhx01 | 77306442 | 77314000 | transcript | HHX010033802.g |
| hhx01 | 89594212 | 89596931 | transcript | HHX010034020.g |
| hhx01 | 91988762 | 91990100 | transcript | HHX010034126.g |
| hhx01 | 93149966 | 93165589 | transcript | HHX010034179.g |
| hhx01 | 93169080 | 93187875 | transcript | HHX010034181.g |
| hhx01 | 94012007 | 94021131 | transcript | HHX010034206.g |
| hhx01 | 94422673 | 94439762 | transcript | HHX010034228.g |
| hhx01 | 99812915 | 99824074 | transcript | HHX010034492.g |
| hhx01 | 106171912 | 106186854 | transcript | HHX010034795.g |
| hhx01 | 106413851 | 106419308 | transcript | HHX010034807.g |
| hhx01 | 106465104 | 106478700 | transcript | HHX010034814.g |
| hhx01 | 106817475 | 106825497 | transcript | HHX010034833.g |
| hhx01 | 108490521 | 108491063 | transcript | HHX010034923.g |
| hhx01 | 108490521 | 108490646 | exon | HHX010034923.g |
| hhx01 | 108490521 | 108490646 | CDS | HHX010034923.g |
| hhx01 | 109541646 | 109552709 | transcript | HHX010034991.g |
| hhx01 | 110439621 | 110448709 | transcript | HHX010035027.g |
| hhx01 | 112422039 | 112426550 | transcript | HHX010035110.g |
| hhx01 | 112422039 | 112422890 | exon | HHX010035110.g |
| hhx01 | 112834784 | 112836890 | transcript | HHX010035132.g |
| hhx01 | 113647751 | 113652893 | transcript | HHX010035157.g |
| hhx01 | 114845598 | 114854143 | transcript | HHX010035212.g |
| hhx01 | 115028061 | 115039748 | transcript | HHX010035226.g |
| hhx01 | 118726874 | 118735331 | transcript | HHX010035414.g |
| hhx01 | 120775880 | 120782232 | transcript | HHX010035546.g |
| hhx01 | 120846436 | 120860821 | transcript | HHX010035548.g |
| hhx01 | 120846436 | 120860821 | transcript | HHX010035548.g |
| hhx01 | 121327493 | 121334723 | transcript | HHX010035587.g |
| hhx01 | 121912229 | 121924542 | transcript | HHX010035621.g |
| hhx01 | 125914099 | 125918098 | transcript | HHX010035859.g |
| hhx01 | 125916474 | 125917006 | exon | HHX010035859.g |
| hhx01 | 125916474 | 125917006 | CDS | HHX010035859.g |
| hhx01 | 129790903 | 129801115 | transcript | HHX010036095.g |
| hhx01 | 129801173 | 129806953 | transcript | HHX010036096.g |
| hhx01 | 131963780 | 131975402 | transcript | HHX010036210.g |
| hhx01 | 132753303 | 132763186 | transcript | HHX010036247.g |
| hhx01 | 134321743 | 134338494 | transcript | HHX010036348.g |
| hhx01 | 136305701 | 136312968 | transcript | HHX010036465.g |
| hhx01 | 137215942 | 137220945 | transcript | HHX010036510.g |
| hhx01 | 139837947 | 139843643 | transcript | HHX010036677.g |
| hhx01 | 142000527 | 142007982 | transcript | HHX010036809.g |
| hhx01 | 142002723 | 142003023 | exon | HHX010036809.g |
| hhx01 | 144374592 | 144375420 | transcript | HHX010036910.g |
| hhx01 | 144374592 | 144375056 | exon | HHX010036910.g |
| hhx01 | 144374592 | 144375056 | CDS | HHX010036910.g |
| hhx01 | 146078359 | 146094682 | transcript | HHX010037050.g |
| hhx01 | 148095575 | 148103987 | transcript | HHX010037162.g |
| hhx01 | 148758922 | 148764382 | transcript | HHX010037194.g |
| hhx01 | 149678603 | 149681758 | transcript | HHX010037263.g |
| hhx01 | 149757362 | 149784202 | transcript | HHX010037272.g |
| hhx01 | 158321277 | 158372467 | transcript | HHX010037581.g |
| hhx01 | 164563077 | 164565644 | transcript | HHX010037758.g |
| hhx01 | 201128415 | 201137697 | transcript | HHX010038285.g |
| hhx01 | 201233063 | 201244278 | transcript | HHX010038292.g |
| hhx02 | 7481692 | 7482596 | transcript | HHX020006967.g |
| hhx02 | 18741825 | 18751663 | transcript | HHX020007438.g |
| hhx02 | 26115614 | 26132541 | transcript | HHX020007782.g |
| hhx02 | 26777358 | 26781824 | transcript | HHX020007818.g |
| hhx02 | 26851898 | 26868383 | transcript | HHX020007825.g |
| hhx02 | 27599458 | 27605027 | transcript | HHX020007867.g |
| hhx02 | 29353825 | 29359386 | transcript | HHX020007978.g |
| hhx02 | 29356059 | 29357066 | exon | HHX020007978.g |
| hhx02 | 29356059 | 29357066 | CDS | HHX020007978.g |
| hhx02 | 29786050 | 29794329 | transcript | HHX020008003.g |
| hhx02 | 31332388 | 31337495 | transcript | HHX020008081.g |
| hhx02 | 32846164 | 32856055 | transcript | HHX020008157.g |
| hhx02 | 40490314 | 40508716 | transcript | HHX020008557.g |
| hhx02 | 47184622 | 47207174 | transcript | HHX020008918.g |
| hhx02 | 48195142 | 48197540 | transcript | HHX020008974.g |
| hhx02 | 48196802 | 48197540 | exon | HHX020008974.g |
| hhx02 | 49168160 | 49171473 | transcript | HHX020009050.g |
| hhx02 | 49622445 | 49624837 | transcript | HHX020009082.g |
| hhx02 | 50757163 | 50764946 | transcript | HHX020009142.g |
| hhx02 | 55123972 | 55126751 | transcript | HHX020009354.g |
| hhx02 | 58032743 | 58036760 | transcript | HHX020009452.g |
| hhx02 | 58032743 | 58033457 | exon | HHX020009452.g |
| hhx02 | 58033259 | 58033457 | CDS | HHX020009452.g |
| hhx02 | 61791892 | 61798333 | transcript | HHX020009601.g |
| hhx02 | 131162725 | 131166681 | transcript | HHX020010564.g |
| hhx02 | 147836535 | 147860705 | transcript | HHX020010809.g |
| hhx03 | 4348832 | 4352992 | transcript | HHX030011319.g |
| hhx03 | 6879230 | 6880988 | transcript | HHX030011449.g |
| hhx03 | 6879359 | 6879443 | exon | HHX030011449.g |
| hhx03 | 14266031 | 14277787 | transcript | HHX030011788.g |
| hhx03 | 14705345 | 14713352 | transcript | HHX030011818.g |
| hhx03 | 15353378 | 15368764 | transcript | HHX030011852.g |
| hhx03 | 17719807 | 17727811 | transcript | HHX030011979.g |
| hhx03 | 18795104 | 18809725 | transcript | HHX030012053.g |
| hhx03 | 18795104 | 18809725 | transcript | HHX030012053.g |
| hhx03 | 18829149 | 18838479 | transcript | HHX030012055.g |
| hhx03 | 23931769 | 23939664 | transcript | HHX030012394.g |
| hhx03 | 24046296 | 24048323 | transcript | HHX030012402.g |
| hhx03 | 27786253 | 27801407 | transcript | HHX030012613.g |
| hhx03 | 28548170 | 28571635 | transcript | HHX030012665.g |
| hhx03 | 34596694 | 34612048 | transcript | HHX030013020.g |
| hhx03 | 34621157 | 34626840 | transcript | HHX030013021.g |
| hhx03 | 39677189 | 39685246 | transcript | HHX030013266.g |
| hhx03 | 40537163 | 40543921 | transcript | HHX030013309.g |
| hhx03 | 45099026 | 45108112 | transcript | HHX030013539.g |
| hhx03 | 50310945 | 50312575 | exon | HHX030013797.g |
| hhx03 | 50310945 | 50320017 | transcript | HHX030013797.g |
| hhx03 | 50782177 | 50794579 | transcript | HHX030013812.g |
| hhx03 | 51274577 | 51282382 | transcript | HHX030013836.g |
| hhx03 | 53936237 | 53940173 | transcript | HHX030013983.g |
| hhx03 | 53944058 | 53947161 | transcript | HHX030013985.g |
| hhx03 | 53958414 | 53964239 | transcript | HHX030013986.g |
| hhx03 | 55803475 | 55807919 | transcript | HHX030014069.g |
| hhx03 | 56106653 | 56115151 | transcript | HHX030014086.g |
| hhx03 | 57758156 | 57758680 | transcript | HHX030014155.g |
| hhx03 | 72008167 | 72026696 | transcript | HHX030014788.g |
| hhx03 | 75775241 | 75783494 | transcript | HHX030014897.g |
| hhx03 | 77168062 | 77179147 | transcript | HHX030014957.g |
| hhx03 | 142029312 | 142039551 | transcript | HHX030015729.g |
| hhx03 | 151242118 | 151270206 | transcript | HHX030015923.g |
| hhx03 | 157488321 | 157490676 | transcript | HHX030016061.g |
| hhx04 | 105885 | 116370 | transcript | HHX040025233.g |
| hhx04 | 4153235 | 4163648 | transcript | HHX040025354.g |
| hhx04 | 40317899 | 40328817 | transcript | HHX040025974.g |
| hhx04 | 57492928 | 57496505 | transcript | HHX040026534.g |
| hhx04 | 59149120 | 59149910 | transcript | HHX040026598.g |
| hhx04 | 63234602 | 63239924 | transcript | HHX040026799.g |
| hhx04 | 68079795 | 68086454 | transcript | HHX040027050.g |
| hhx04 | 73045479 | 73053804 | transcript | HHX040027361.g |
| hhx04 | 75114431 | 75121478 | transcript | HHX040027484.g |
| hhx04 | 77959453 | 77961522 | transcript | HHX040027666.g |
| hhx04 | 79177501 | 79181882 | transcript | HHX040027715.g |
| hhx04 | 81207339 | 81209344 | transcript | HHX040027852.g |
| hhx04 | 81372251 | 81377065 | transcript | HHX040027861.g |
| hhx04 | 82864372 | 82877380 | transcript | HHX040027945.g |
| hhx04 | 83560722 | 83561084 | transcript | HHX040027980.g |
| hhx04 | 83689263 | 83689375 | exon | HHX040027990.g |
| hhx04 | 83689263 | 83689375 | CDS | HHX040027990.g |
| hhx04 | 83689263 | 83689574 | transcript | HHX040027990.g |
| hhx04 | 83934089 | 83947557 | transcript | HHX040028001.g |
| hhx04 | 84282835 | 84283214 | exon | HHX040028019.g |
| hhx04 | 84282835 | 84283214 | CDS | HHX040028019.g |
| hhx04 | 84278626 | 84283214 | transcript | HHX040028019.g |
| hhx04 | 85316437 | 85334526 | transcript | HHX040028083.g |
| hhx04 | 85334576 | 85344478 | transcript | HHX040028084.g |
| hhx04 | 86621421 | 86636624 | transcript | HHX040028130.g |
| hhx04 | 88243663 | 88251569 | transcript | HHX040028213.g |
| hhx04 | 92823287 | 92830496 | transcript | HHX040028447.g |
| hhx04 | 97368024 | 97370882 | transcript | HHX040028690.g |
| hhx04 | 102436483 | 102436770 | transcript | HHX040028957.g |
| hhx04 | 102436483 | 102436770 | exon | HHX040028957.g |
| hhx04 | 102436483 | 102436770 | CDS | HHX040028957.g |
| hhx04 | 103918230 | 103921086 | transcript | HHX040029056.g |
| hhx04 | 103920597 | 103921086 | exon | HHX040029056.g |
| hhx04 | 103920597 | 103921086 | CDS | HHX040029056.g |
| hhx04 | 111118689 | 111149621 | transcript | HHX040029412.g |
| hhx04 | 112228587 | 112239261 | transcript | HHX040029463.g |
| hhx04 | 116522047 | 116526999 | transcript | HHX040029712.g |
| hhx04 | 117619853 | 117640966 | transcript | HHX040029792.g |
| hhx04 | 119148086 | 119153665 | transcript | HHX040029856.g |
| hhx04 | 119654517 | 119668846 | transcript | HHX040029879.g |
| hhx04 | 119671167 | 119684769 | transcript | HHX040029880.g |
| hhx04 | 121489813 | 121505981 | transcript | HHX040029998.g |
| hhx04 | 122418450 | 122428935 | transcript | HHX040030024.g |
| hhx04 | 128492305 | 128516882 | transcript | HHX040030283.g |
| hhx04 | 130402199 | 130411061 | transcript | HHX040030378.g |
| hhx04 | 136992448 | 137001971 | transcript | HHX040030795.g |
| hhx04 | 139038865 | 139040853 | transcript | HHX040030904.g |
| hhx04 | 147532941 | 147540294 | transcript | HHX040031443.g |
| hhx04 | 150817803 | 150848362 | transcript | HHX040031590.g |
| hhx04 | 150995637 | 151002153 | transcript | HHX040031593.g |
| hhx04 | 151461920 | 151478084 | transcript | HHX040031615.g |
| hhx05 | 2366288 | 2368969 | transcript | HHX050044100.g |
| hhx05 | 4034654 | 4042156 | transcript | HHX050044227.g |
| hhx05 | 4149297 | 4166281 | transcript | HHX050044240.g |
| hhx05 | 7124102 | 7136859 | transcript | HHX050044434.g |
| hhx05 | 8531471 | 8547692 | transcript | HHX050044514.g |
| hhx05 | 13566390 | 13598889 | transcript | HHX050044746.g |
| hhx05 | 17244287 | 17247593 | transcript | HHX050044920.g |
| hhx05 | 17817377 | 17821583 | transcript | HHX050044945.g |
| hhx05 | 57207740 | 57220808 | transcript | HHX050045670.g |
| hhx05 | 57207740 | 57220808 | transcript | HHX050045670.g |
| hhx05 | 61291665 | 61312918 | transcript | HHX050045797.g |
| hhx05 | 63527746 | 63535257 | transcript | HHX050045935.g |
| hhx05 | 63769878 | 63783699 | transcript | HHX050045946.g |
| hhx05 | 68711049 | 68718617 | transcript | HHX050046181.g |
| hhx05 | 71165134 | 71181402 | transcript | HHX050046300.g |
| hhx05 | 75578872 | 75580481 | transcript | HHX050046533.g |
| hhx05 | 77927401 | 77941659 | transcript | HHX050046642.g |
| hhx05 | 79779097 | 79795452 | transcript | HHX050046790.g |
| hhx05 | 81824351 | 81835155 | transcript | HHX050046877.g |
| hhx05 | 82032596 | 82044294 | transcript | HHX050046896.g |
| hhx05 | 85289411 | 85324410 | transcript | HHX050047025.g |
| hhx05 | 85289411 | 85324410 | transcript | HHX050047025.g |
| hhx05 | 87006349 | 87013107 | transcript | HHX050047129.g |
| hhx05 | 87914995 | 87925987 | transcript | HHX050047183.g |
| hhx05 | 95574043 | 95585354 | transcript | HHX050047488.g |
| hhx05 | 137183307 | 137199437 | transcript | HHX050048359.g |
| hhx05 | 138774891 | 138790043 | transcript | HHX050048434.g |
| hhx05 | 139166946 | 139180135 | transcript | HHX050048454.g |
| hhx05 | 141295743 | 141304549 | transcript | HHX050048535.g |
| hhx05 | 144517112 | 144551287 | transcript | HHX050048701.g |
| hhx05 | 144916319 | 144918040 | transcript | HHX050048723.g |
| hhx05 | 144920357 | 144925949 | transcript | HHX050048725.g |
| hhx05 | 147712675 | 147721804 | transcript | HHX050048883.g |
| hhx05 | 149507520 | 149515800 | transcript | HHX050048998.g |
| hhx05 | 150316055 | 150326275 | transcript | HHX050049048.g |
| hhx05 | 152228541 | 152233595 | transcript | HHX050049190.g |
| hhx06 | 16049395 | 16066518 | transcript | HHX060016620.g |
| hhx06 | 23676547 | 23688427 | transcript | HHX060016844.g |
| hhx06 | 30027045 | 30029546 | transcript | HHX060017027.g |
| hhx06 | 65037498 | 65042800 | transcript | HHX060017607.g |
| hhx06 | 116714885 | 116725213 | transcript | HHX060018749.g |
| hhx06 | 125605747 | 125609619 | transcript | HHX060019173.g |
| hhx06 | 127185340 | 127217856 | transcript | HHX060019272.g |
| hhx07 | 19336132 | 19347954 | transcript | HHX070055390.g |
| hhx07 | 19956107 | 19956391 | transcript | HHX070055426.g |
| hhx07 | 19956107 | 19956391 | exon | HHX070055426.g |
| hhx07 | 19956107 | 19956391 | CDS | HHX070055426.g |
| hhx07 | 21641529 | 21655723 | transcript | HHX070055556.g |
| hhx07 | 32436894 | 32446632 | transcript | HHX070056013.g |
| hhx07 | 33945451 | 33946337 | transcript | HHX070056135.g |
| hhx07 | 34391602 | 34397067 | transcript | HHX070056178.g |
| hhx07 | 34391602 | 34397786 | transcript | HHX070056179.g |
| hhx07 | 35732205 | 35746793 | transcript | HHX070056245.g |
| hhx07 | 37683503 | 37691043 | transcript | HHX070056391.g |
| hhx07 | 39223328 | 39232933 | transcript | HHX070056493.g |
| hhx07 | 42625385 | 42641494 | transcript | HHX070056658.g |
| hhx07 | 42643315 | 42649337 | transcript | HHX070056659.g |
| hhx07 | 42886751 | 42939278 | transcript | HHX070056675.g |
| hhx07 | 46100987 | 46117378 | transcript | HHX070056824.g |
| hhx07 | 49654567 | 49657904 | transcript | HHX070057069.g |
| hhx07 | 49870047 | 49880468 | transcript | HHX070057087.g |
| hhx07 | 57044639 | 57062224 | transcript | HHX070057481.g |
| hhx07 | 61166235 | 61170145 | transcript | HHX070057681.g |
| hhx07 | 61756377 | 61790047 | transcript | HHX070057710.g |
| hhx07 | 70020193 | 70027883 | transcript | HHX070057997.g |
| hhx07 | 80258577 | 80264450 | transcript | HHX070058284.g |
| hhx07 | 80264149 | 80264359 | exon | HHX070058284.g |
| hhx07 | 80264149 | 80264359 | CDS | HHX070058284.g |
| hhx07 | 88760108 | 88770209 | transcript | HHX070058503.g |
| hhx07 | 92257101 | 92279699 | transcript | HHX070058697.g |
| hhx07 | 94992349 | 95009081 | transcript | HHX070058870.g |
| hhx07 | 97042860 | 97062418 | transcript | HHX070059017.g |
| hhx07 | 99931565 | 99948067 | transcript | HHX070059175.g |
| hhx07 | 105070096 | 105076289 | transcript | HHX070059412.g |
| hhx07 | 107338987 | 107350501 | transcript | HHX070059530.g |
| hhx08 | 2824599 | 2832359 | transcript | HHX080002957.g |
| hhx08 | 8583554 | 8585753 | transcript | HHX080003113.g |
| hhx08 | 53738498 | 53741172 | transcript | HHX080003730.g |
| hhx08 | 57410663 | 57410919 | exon | HHX080003829.g |
| hhx08 | 57406885 | 57417755 | transcript | HHX080003829.g |
| hhx08 | 59481539 | 59481854 | transcript | HHX080003910.g |
| hhx08 | 59481539 | 59481750 | exon | HHX080003910.g |
| hhx08 | 59481539 | 59481750 | CDS | HHX080003910.g |
| hhx08 | 64514859 | 64531886 | transcript | HHX080004159.g |
| hhx08 | 64832353 | 64842113 | transcript | HHX080004172.g |
| hhx08 | 65036109 | 65048469 | transcript | HHX080004180.g |
| hhx08 | 65536615 | 65537516 | transcript | HHX080004203.g |
| hhx08 | 69458119 | 69461205 | transcript | HHX080004350.g |
| hhx08 | 73481100 | 73492477 | transcript | HHX080004668.g |
| hhx08 | 73893105 | 73893538 | transcript | HHX080004684.g |
| hhx08 | 74724281 | 74734056 | transcript | HHX080004730.g |
| hhx08 | 77176433 | 77183219 | transcript | HHX080004872.g |
| hhx08 | 77176433 | 77176947 | exon | HHX080004872.g |
| hhx08 | 77176433 | 77176947 | CDS | HHX080004872.g |
| hhx08 | 78413476 | 78419143 | transcript | HHX080004933.g |
| hhx08 | 78433813 | 78443096 | transcript | HHX080004934.g |
| hhx08 | 82078102 | 82080390 | transcript | HHX080005129.g |
| hhx08 | 83378466 | 83381193 | transcript | HHX080005207.g |
| hhx08 | 83869140 | 83876473 | transcript | HHX080005229.g |
| hhx08 | 85049577 | 85062913 | transcript | HHX080005287.g |
| hhx08 | 85598388 | 85602443 | transcript | HHX080005307.g |
| hhx08 | 88599936 | 88612867 | transcript | HHX080005400.g |
| hhx08 | 92488694 | 92491512 | transcript | HHX080005526.g |
| hhx08 | 92835590 | 92836346 | transcript | HHX080005543.g |
| hhx08 | 97196102 | 97201921 | transcript | HHX080005743.g |
| hhx08 | 101571151 | 101587542 | transcript | HHX080006040.g |
| hhx08 | 104997963 | 105008387 | transcript | HHX080006281.g |
| hhx08 | 105946647 | 105953091 | transcript | HHX080006375.g |
| hhx08 | 106630985 | 106634547 | transcript | HHX080006439.g |
| hhx08 | 107224192 | 107241178 | transcript | HHX080006498.g |
| hhx09 | 28619183 | 28624368 | transcript | HHX090024589.g |
| hhx10 | 3365779 | 3366713 | transcript | HHX100019633.g |
| hhx10 | 3499779 | 3502256 | transcript | HHX100019639.g |
| hhx10 | 5231731 | 5244517 | transcript | HHX100019716.g |
| hhx10 | 6996253 | 7004011 | transcript | HHX100019827.g |
| hhx10 | 6996253 | 6997328 | exon | HHX100019827.g |
| hhx10 | 12305779 | 12308206 | transcript | HHX100020069.g |
| hhx10 | 14829678 | 14831167 | transcript | HHX100020213.g |
| hhx10 | 14830369 | 14831167 | exon | HHX100020213.g |
| hhx10 | 14830369 | 14831167 | CDS | HHX100020213.g |
| hhx10 | 14867787 | 14875224 | transcript | HHX100020215.g |
| hhx10 | 19757872 | 19769084 | transcript | HHX100020472.g |
| hhx10 | 20122330 | 20123163 | transcript | HHX100020490.g |
| hhx10 | 20137249 | 20153388 | transcript | HHX100020492.g |
| hhx10 | 22727985 | 22744442 | transcript | HHX100020654.g |
| hhx10 | 26315851 | 26332934 | transcript | HHX100020834.g |
| hhx10 | 27620540 | 27622514 | transcript | HHX100020903.g |
| hhx10 | 29000539 | 29014402 | transcript | HHX100020973.g |
| hhx10 | 30443525 | 30452137 | transcript | HHX100021051.g |
| hhx10 | 30607426 | 30657154 | transcript | HHX100021060.g |
| hhx10 | 35533402 | 35538434 | transcript | HHX100021359.g |
| hhx10 | 36933265 | 36945696 | transcript | HHX100021434.g |
| hhx10 | 38286819 | 38308338 | transcript | HHX100021521.g |
| hhx10 | 42420391 | 42424352 | transcript | HHX100021763.g |
| hhx10 | 43935434 | 43944332 | transcript | HHX100021844.g |
| hhx10 | 45090783 | 45094784 | transcript | HHX100021899.g |
| hhx10 | 46660075 | 46664238 | transcript | HHX100021995.g |
| hhx10 | 48290304 | 48299023 | transcript | HHX100022078.g |
| hhx10 | 48320983 | 48331185 | transcript | HHX100022079.g |
| hhx10 | 49748921 | 49756521 | transcript | HHX100022166.g |
| hhx10 | 50418204 | 50434535 | transcript | HHX100022208.g |
| hhx10 | 51021569 | 51030513 | transcript | HHX100022239.g |
| hhx10 | 52077536 | 52079014 | transcript | HHX100022291.g |
| hhx10 | 52912840 | 52918375 | transcript | HHX100022327.g |
| hhx10 | 53591325 | 53595097 | transcript | HHX100022356.g |
| hhx10 | 56454422 | 56461064 | transcript | HHX100022498.g |
| hhx10 | 61662732 | 61668234 | transcript | HHX100022746.g |
| hhx10 | 61667905 | 61668234 | exon | HHX100022746.g |
| hhx10 | 61667905 | 61668234 | CDS | HHX100022746.g |
| hhx10 | 63893599 | 63894546 | transcript | HHX100022880.g |
| hhx10 | 63894439 | 63894546 | exon | HHX100022880.g |
| hhx10 | 63894439 | 63894546 | CDS | HHX100022880.g |
| hhx10 | 67766582 | 67770408 | transcript | HHX100023045.g |
| hhx11 | 2278116 | 2287797 | transcript | HHX110050459.g |
| hhx11 | 4121458 | 4123179 | transcript | HHX110050530.g |
| hhx11 | 8044759 | 8046696 | transcript | HHX110050714.g |
| hhx11 | 8044759 | 8045198 | exon | HHX110050714.g |
| hhx11 | 8045081 | 8045198 | CDS | HHX110050714.g |
| hhx11 | 9430239 | 9474952 | transcript | HHX110050785.g |
| hhx11 | 11109329 | 11124893 | transcript | HHX110050879.g |
| hhx11 | 12475107 | 12478767 | transcript | HHX110050947.g |
| hhx11 | 16722006 | 16733038 | transcript | HHX110051190.g |
| hhx11 | 17889171 | 17896142 | transcript | HHX110051225.g |
| hhx11 | 20007242 | 20016447 | transcript | HHX110051298.g |
| hhx11 | 22353205 | 22361084 | transcript | HHX110051372.g |
| hhx11 | 36212562 | 36216078 | transcript | HHX110051657.g |
| hhx11 | 70397962 | 70401865 | transcript | HHX110052165.g |
| hhx11 | 81233247 | 81235930 | transcript | HHX110052490.g |
| hhx11 | 83156181 | 83174048 | transcript | HHX110052585.g |
| hhx11 | 87685276 | 87697808 | transcript | HHX110052797.g |
| hhx11 | 88684193 | 88685258 | transcript | HHX110052853.g |
| hhx11 | 88833381 | 88848388 | transcript | HHX110052875.g |
| hhx12 | 2565052 | 2567690 | transcript | HHX120000129.g |
| hhx12 | 2567175 | 2567690 | exon | HHX120000129.g |
| hhx12 | 2567175 | 2567690 | CDS | HHX120000129.g |
| hhx12 | 11664969 | 11681351 | transcript | HHX120000423.g |
| hhx12 | 13736749 | 13743810 | transcript | HHX120000509.g |
| hhx12 | 36818490 | 36819799 | transcript | HHX120000911.g |
| hhx12 | 36819130 | 36819353 | exon | HHX120000911.g |
| hhx12 | 36819130 | 36819353 | CDS | HHX120000911.g |
| hhx12 | 41503456 | 41503920 | transcript | HHX120001029.g |
| hhx12 | 41503456 | 41503920 | exon | HHX120001029.g |
| hhx12 | 41503456 | 41503920 | CDS | HHX120001029.g |
| hhx12 | 46617865 | 46618123 | exon | HHX120001200.g |
| hhx12 | 46617865 | 46618123 | CDS | HHX120001200.g |
| hhx12 | 46610925 | 46618851 | transcript | HHX120001200.g |
| hhx12 | 47823825 | 47845793 | transcript | HHX120001291.g |
| hhx12 | 47960278 | 47980144 | transcript | HHX120001294.g |
| hhx12 | 53123135 | 53123374 | transcript | HHX120001537.g |
| hhx12 | 53123135 | 53123374 | exon | HHX120001537.g |
| hhx12 | 53123135 | 53123374 | CDS | HHX120001537.g |
| hhx12 | 54156881 | 54180417 | transcript | HHX120001613.g |
| hhx12 | 54987945 | 54993201 | transcript | HHX120001655.g |
| hhx12 | 58894494 | 58902574 | transcript | HHX120001816.g |
| hhx12 | 61212283 | 61227373 | transcript | HHX120001935.g |
| hhx12 | 61232076 | 61244958 | transcript | HHX120001936.g |
| hhx12 | 62675552 | 62688426 | transcript | HHX120002018.g |
| hhx12 | 64773714 | 64779072 | transcript | HHX120002114.g |
| hhx12 | 64776128 | 64776845 | exon | HHX120002114.g |
| hhx12 | 64776609 | 64776845 | CDS | HHX120002114.g |
| hhx12 | 65073086 | 65079746 | transcript | HHX120002130.g |
| hhx12 | 65651437 | 65654770 | transcript | HHX120002162.g |
| hhx12 | 72751577 | 72770823 | transcript | HHX120002415.g |
| hhx13 | 56129104 | 56129698 | transcript | HHX130039033.g |
| hhx13 | 65887714 | 65906513 | transcript | HHX130039211.g |
| hhx13 | 74632728 | 74636015 | transcript | HHX130039534.g |
| hhx13 | 75260676 | 75273732 | transcript | HHX130039564.g |
| hhx13 | 78717472 | 78729813 | transcript | HHX130039690.g |
| hhx13 | 79706134 | 79711483 | transcript | HHX130039742.g |
| hhx13 | 80963487 | 80973982 | transcript | HHX130039825.g |
| hhx13 | 82643029 | 82656957 | transcript | HHX130039909.g |
| hhx13 | 82659654 | 82667370 | transcript | HHX130039910.g |
| hhx13 | 85008236 | 85015274 | transcript | HHX130040061.g |
| hhx14 | 11179683 | 11190074 | transcript | HHX140040513.g |
| hhx14 | 20104044 | 20118990 | transcript | HHX140040989.g |
| hhx14 | 20684416 | 20696681 | transcript | HHX140041033.g |
| hhx14 | 21509605 | 21531818 | transcript | HHX140041086.g |
| hhx14 | 26109909 | 26111308 | transcript | HHX140041351.g |
| hhx14 | 26184006 | 26198680 | transcript | HHX140041355.g |
| hhx14 | 27249900 | 27250094 | transcript | HHX140041397.g |
| hhx14 | 27249900 | 27250094 | exon | HHX140041397.g |
| hhx14 | 27249900 | 27250094 | CDS | HHX140041397.g |
| hhx14 | 27361321 | 27372207 | transcript | HHX140041398.g |
| hhx14 | 27363888 | 27363988 | exon | HHX140041398.g |
| hhx14 | 27363888 | 27363988 | CDS | HHX140041398.g |
| hhx14 | 27602454 | 27613638 | transcript | HHX140041402.g |
| hhx14 | 29001367 | 29031992 | transcript | HHX140041440.g |
| hhx14 | 69943007 | 69959444 | transcript | HHX140043059.g |
| hhx14 | 70997174 | 71000396 | transcript | HHX140043117.g |
| hhx14 | 76270250 | 76279029 | transcript | HHX140043549.g |
| hhx14 | 81110644 | 81113178 | transcript | HHX140043844.g |
| hhx14 | 82216270 | 82221251 | transcript | HHX140043920.g |
| hhx15 | 4727967 | 4731247 | transcript | HHX150061350.g |
| hhx15 | 26069431 | 26082970 | transcript | HHX150061776.g |
| hhx15 | 26069431 | 26070144 | exon | HHX150061776.g |
| hhx15 | 73650037 | 73687421 | transcript | HHX150062423.g |
| hhx15 | 74359013 | 74370705 | transcript | HHX150062465.g |
| hhx16 | 1917875 | 1931346 | transcript | HHX160062597.g |
| hhx16 | 19775807 | 19783659 | transcript | HHX160062943.g |
| hhx16 | 24284446 | 24289642 | transcript | HHX160063098.g |
| hhx16 | 25241963 | 25242720 | transcript | HHX160063144.g |
| hhx16 | 25799656 | 25802071 | transcript | HHX160063154.g |
| hhx16 | 26712779 | 26716327 | transcript | HHX160063179.g |
| hhx16 | 26860704 | 26869492 | transcript | HHX160063180.g |
| hhx16 | 50835068 | 50866686 | transcript | HHX160063483.g |
| hhx16 | 64987981 | 64996077 | transcript | HHX160063800.g |
| hhx16 | 70211803 | 70245661 | transcript | HHX160064043.g |
| hhx16 | 72122545 | 72125028 | transcript | HHX160064148.g |
| hhx16 | 72123219 | 72123427 | exon | HHX160064148.g |
| hhx16 | 72123219 | 72123427 | CDS | HHX160064148.g |
| hhx17 | 44218822 | 44227255 | transcript | HHX170049957.g |
| hhx17 | 47069046 | 47080340 | transcript | HHX170050065.g |
| hhx17 | 49185078 | 49186080 | transcript | HHX170050150.g |
| hhx17 | 50477381 | 50485508 | transcript | HHX170050203.g |
| hhx17 | 50980071 | 50986854 | transcript | HHX170050218.g |
| hhx18 | 36645404 | 36650569 | transcript | HHX180060597.g |
| hhx18 | 37352262 | 37352654 | transcript | HHX180060625.g |
| hhx18 | 37352262 | 37352654 | exon | HHX180060625.g |
| hhx18 | 37352262 | 37352654 | CDS | HHX180060625.g |
| hhx18 | 42308879 | 42333139 | transcript | HHX180060939.g |
| hhx19 | 6244300 | 6247847 | transcript | HHX190053238.g |
| hhx19 | 11333716 | 11336062 | transcript | HHX190053458.g |
| hhx19 | 13982680 | 14004834 | transcript | HHX190053608.g |
| hhx19 | 20442425 | 20443768 | transcript | HHX190054004.g |
| hhx19 | 39934603 | 39951370 | transcript | HHX190054293.g |
| hhx19 | 46214141 | 46214747 | transcript | HHX190054399.g |
| hhx19 | 46214141 | 46214435 | exon | HHX190054399.g |
| hhx19 | 46214141 | 46214435 | CDS | HHX190054399.g |
| hhx08 | 106979048 | 106999638 | transcript | HHX080006471.g |
| hhx02 | 38931053 | 38936875 | transcript | HHX020008465.g |
| hhx04 | 150996574 | 150999580 | transcript | HHX040031593.g |
| hhx01 | 112422171 | 112422845 | transcript | HHX010035110.g |
| hhx07 | 98731033 | 98740527 | transcript | HHX070059131.g |
| hhx10 | 20146284 | 20151139 | transcript | HHX100020492.g |
| hhx10 | 52077973 | 52078153 | transcript | HHX100022291.g |
| hhx03 | 55075567 | 55091155 | transcript | HHX030014039.g |
| hhx05 | 4038517 | 4040157 | transcript | HHX050044227.g |
| hhx08 | 55512329 | 55512575 | transcript | HHX080003770.g |
| hhx12 | 36819241 | 36819342 | transcript | HHX120000911.g |
| hhx01 | 106416655 | 106419278 | transcript | HHX010034807.g |
| hhx02 | 34281368 | 34292822 | transcript | HHX020008232.g |
| hhx10 | 12305906 | 12305999 | transcript | HHX100020069.g |

**B.**

| ***Michelia champaca*_CHG** | | | | |
| --- | --- | --- | --- | --- |
| hhx04 | 118601996 | 118614837 | transcript | HHX040029828.g |
| hhx08 | 2997804 | 3022681 | transcript | HHX080002967.g |
| hhx14 | 58506433 | 58511473 | transcript | HHX140042308.g |
| hhx10 | 40814209 | 40840983 | transcript | HHX100021664.g |
| hhx05 | 72929380 | 72947901 | transcript | HHX050046402.g |
| hhx10 | 53598008 | 53605110 | transcript | HHX100022357.g |
| hhx05 | 141234419 | 141247191 | transcript | HHX050048534.g |
| hhx08 | 84321186 | 84338715 | transcript | HHX080005257.g |
| hhx05 | 74394768 | 74425141 | transcript | HHX050046474.g |
| hhx05 | 63527746 | 63535257 | transcript | HHX050045935.g |
| hhx05 | 79517023 | 79534005 | transcript | HHX050046769.g |
| hhx05 | 13566390 | 13598889 | transcript | HHX050044746.g |
| hhx14 | 22036879 | 22042922 | transcript | HHX140041104.g |
| hhx17 | 49873578 | 49888060 | transcript | HHX170050183.g |
| hhx17 | 53472466 | 53487164 | transcript | HHX170050323.g |
| hhx01 | 100590862 | 100610918 | transcript | HHX010034510.g |
| hhx06 | 127185340 | 127217856 | transcript | HHX060019272.g |
| hhx05 | 56282156 | 56294879 | transcript | HHX050045633.g |
| hhx18 | 42308879 | 42333139 | transcript | HHX180060939.g |
| hhx10 | 8115422 | 8135053 | transcript | HHX100019873.g |
| hhx05 | 5110309 | 5143113 | transcript | HHX050044310.g |
| hhx16 | 25438636 | 25441177 | transcript | HHX160063146.g |
| hhx10 | 49748921 | 49756521 | transcript | HHX100022166.g |
| hhx04 | 95983412 | 95999511 | transcript | HHX040028603.g |
| hhx04 | 112228587 | 112239261 | transcript | HHX040029463.g |
| hhx04 | 1256133 | 1266005 | transcript | HHX040025281.g |
| hhx11 | 9485576 | 9499210 | transcript | HHX110050787.g |
| hhx12 | 66892052 | 66899349 | transcript | HHX120002229.g |
| hhx02 | 142382347 | 142392089 | transcript | HHX020010778.g |
| hhx01 | 120846436 | 120860821 | transcript | HHX010035548.g |
| hhx02 | 31620243 | 31629897 | transcript | HHX020008099.g |
| hhx03 | 18829149 | 18838479 | transcript | HHX030012055.g |
| hhx14 | 21509605 | 21531818 | transcript | HHX140041086.g |
| hhx06 | 17918572 | 17932492 | transcript | HHX060016677.g |
| hhx04 | 145364007 | 145374672 | transcript | HHX040031329.g |
| hhx05 | 77304781 | 77310999 | transcript | HHX050046616.g |
| hhx10 | 46175479 | 46185489 | transcript | HHX100021970.g |
| hhx12 | 60829736 | 60858254 | transcript | HHX120001922.g |
| hhx14 | 76270250 | 76279029 | transcript | HHX140043549.g |
| hhx05 | 144517112 | 144551287 | transcript | HHX050048701.g |
| hhx06 | 113357758 | 113363337 | transcript | HHX060018585.g |
| hhx11 | 74257627 | 74269792 | transcript | HHX110052243.g |
| hhx05 | 140510766 | 140521444 | transcript | HHX050048507.g |
| hhx03 | 56847076 | 56861101 | transcript | HHX030014129.g |
| hhx12 | 64773714 | 64779072 | transcript | HHX120002114.g |
| hhx01 | 94719600 | 94724627 | transcript | HHX010034244.g |
| hhx02 | 52965947 | 52977867 | transcript | HHX020009249.g |
| hhx04 | 135461 | 150770 | transcript | HHX040025236.g |
| hhx10 | 20137249 | 20153388 | transcript | HHX100020492.g |
| hhx10 | 22727985 | 22744442 | transcript | HHX100020654.g |
| hhx12 | 51870400 | 51886956 | transcript | HHX120001461.g |
| hhx11 | 9430239 | 9474952 | transcript | HHX110050785.g |
| hhx01 | 24989317 | 24999098 | transcript | HHX010032978.g |
| hhx14 | 59883524 | 59889071 | transcript | HHX140042409.g |
| hhx04 | 60427978 | 60448047 | transcript | HHX040026652.g |
| hhx12 | 66979052 | 66985070 | transcript | HHX120002238.g |
| hhx04 | 52356926 | 52365825 | transcript | HHX040026296.g |
| hhx10 | 45121381 | 45152710 | transcript | HHX100021901.g |
| hhx07 | 21993799 | 22004540 | transcript | HHX070055568.g |
| hhx07 | 52723258 | 52732811 | transcript | HHX070057288.g |
| hhx03 | 78233071 | 78247827 | transcript | HHX030014984.g |
| hhx18 | 41515749 | 41531926 | transcript | HHX180060892.g |
| hhx04 | 130536127 | 130546410 | transcript | HHX040030393.g |
| hhx01 | 109956487 | 109961862 | transcript | HHX010035010.g |
| hhx11 | 86233240 | 86249129 | transcript | HHX110052707.g |
| hhx12 | 64608069 | 64633384 | transcript | HHX120002109.g |
| hhx05 | 11785034 | 11806325 | transcript | HHX050044656.g |
| hhx17 | 50477381 | 50485508 | transcript | HHX170050203.g |
| hhx03 | 35043613 | 35057878 | transcript | HHX030013038.g |
| hhx14 | 21509605 | 21531818 | transcript | HHX140041086.g |
| hhx04 | 100510426 | 100522410 | transcript | HHX040028836.g |
| hhx07 | 99206866 | 99208365 | transcript | HHX070059148.g |
| hhx01 | 134321743 | 134338494 | transcript | HHX010036348.g |
| hhx04 | 95158754 | 95179205 | transcript | HHX040028567.g |
| hhx02 | 40490314 | 40508716 | transcript | HHX020008557.g |
| hhx14 | 29193418 | 29195171 | transcript | HHX140041445.g |
| hhx07 | 39223328 | 39232933 | transcript | HHX070056493.g |
| hhx08 | 64514859 | 64531886 | transcript | HHX080004159.g |
| hhx05 | 71165134 | 71181402 | transcript | HHX050046300.g |
| hhx02 | 43185475 | 43195998 | transcript | HHX020008724.g |
| hhx01 | 6396942 | 6404192 | transcript | HHX010032195.g |
| hhx10 | 30194185 | 30234392 | transcript | HHX100021036.g |
| hhx04 | 119121420 | 119126243 | transcript | HHX040029853.g |
| hhx07 | 37683503 | 37691043 | transcript | HHX070056391.g |
| hhx03 | 147276178 | 147288395 | transcript | HHX030015802.g |
| hhx07 | 55648781 | 55669299 | transcript | HHX070057430.g |
| hhx11 | 16722006 | 16733038 | transcript | HHX110051190.g |
| hhx03 | 160972169 | 160980879 | transcript | HHX030016160.g |
| hhx06 | 109720778 | 109753396 | transcript | HHX060018416.g |
| hhx07 | 42886751 | 42939278 | transcript | HHX070056675.g |
| hhx10 | 45104260 | 45116471 | transcript | HHX100021900.g |
| hhx03 | 52443736 | 52460745 | transcript | HHX030013897.g |
| hhx01 | 124095834 | 124100099 | transcript | HHX010035769.g |
| hhx11 | 88611261 | 88632431 | transcript | HHX110052850.g |
| hhx08 | 97196102 | 97201921 | transcript | HHX080005743.g |
| hhx18 | 42365006 | 42385665 | transcript | HHX180060943.g |
| hhx06 | 63297046 | 63302330 | transcript | HHX060017576.g |
| hhx10 | 27620540 | 27622514 | transcript | HHX100020903.g |
| hhx01 | 155698741 | 155702970 | transcript | HHX010037510.g |
| hhx02 | 70462259 | 70466085 | transcript | HHX020009718.g |
| hhx10 | 54494063 | 54505856 | transcript | HHX100022402.g |
| hhx19 | 1638288 | 1657574 | transcript | HHX190053078.g |
| hhx19 | 15314066 | 15326516 | transcript | HHX190053681.g |
| hhx05 | 142119172 | 142121737 | transcript | HHX050048580.g |
| hhx05 | 142119172 | 142125112 | transcript | HHX050048581.g |
| hhx16 | 70689535 | 70693087 | transcript | HHX160064085.g |
| hhx05 | 63812308 | 63818490 | transcript | HHX050045948.g |
| hhx03 | 14206644 | 14215100 | transcript | HHX030011780.g |
| hhx08 | 88599936 | 88612867 | transcript | HHX080005400.g |
| hhx13 | 86013821 | 86028443 | transcript | HHX130040123.g |
| hhx02 | 17965715 | 17979893 | transcript | HHX020007389.g |
| hhx07 | 103712198 | 103718484 | transcript | HHX070059348.g |
| hhx04 | 62121950 | 62132141 | transcript | HHX040026743.g |
| hhx07 | 105070096 | 105076289 | transcript | HHX070059412.g |
| hhx01 | 99824248 | 99840596 | transcript | HHX010034493.g |
| hhx10 | 56117612 | 56128206 | transcript | HHX100022477.g |
| hhx10 | 56123980 | 56128929 | transcript | HHX100022478.g |
| hhx07 | 51814535 | 51824632 | transcript | HHX070057226.g |
| hhx14 | 70928469 | 70933403 | transcript | HHX140043108.g |
| hhx07 | 65347613 | 65356833 | transcript | HHX070057825.g |
| hhx05 | 150316055 | 150326275 | transcript | HHX050049048.g |
| hhx08 | 16087737 | 16096627 | transcript | HHX080003250.g |
| hhx04 | 81036286 | 81038297 | transcript | HHX040027844.g |
| hhx07 | 13114439 | 13117483 | transcript | HHX070054983.g |
| hhx05 | 77927401 | 77941659 | transcript | HHX050046642.g |
| hhx10 | 45121381 | 45152710 | transcript | HHX100021901.g |
| hhx07 | 26090151 | 26091611 | transcript | HHX070055769.g |
| hhx07 | 26091385 | 26091611 | exon | HHX070055769.g |
| hhx10 | 68776529 | 68776903 | transcript | HHX100023083.g |
| hhx10 | 68776529 | 68776720 | exon | HHX100023083.g |
| hhx10 | 68776529 | 68776720 | CDS | HHX100023083.g |
| hhx05 | 65062662 | 65070471 | transcript | HHX050046007.g |
| hhx05 | 71560942 | 71577321 | transcript | HHX050046318.g |
| hhx07 | 19944170 | 19956272 | transcript | HHX070055425.g |
| hhx05 | 103140892 | 103141108 | exon | HHX050047692.g |
| hhx05 | 103140965 | 103141108 | CDS | HHX050047692.g |
| hhx05 | 103135661 | 103141884 | transcript | HHX050047692.g |
| hhx03 | 74294240 | 74302824 | transcript | HHX030014838.g |
| hhx01 | 2287459 | 2298775 | transcript | HHX010031894.g |
| hhx10 | 28827505 | 28830548 | transcript | HHX100020963.g |
| hhx17 | 53297836 | 53313877 | transcript | HHX170050315.g |
| hhx10 | 14859558 | 14865953 | transcript | HHX100020214.g |
| hhx01 | 135709464 | 135715473 | transcript | HHX010036432.g |
| hhx05 | 141159969 | 141168163 | transcript | HHX050048526.g |
| hhx01 | 1190040 | 1193342 | transcript | HHX010031817.g |
| hhx03 | 151953540 | 151966665 | transcript | HHX030015949.g |
| hhx05 | 13566390 | 13598889 | transcript | HHX050044746.g |
| hhx10 | 81855854 | 81856726 | transcript | HHX100023553.g |
| hhx06 | 129320480 | 129329056 | transcript | HHX060019339.g |
| hhx04 | 125053787 | 125075158 | transcript | HHX040030152.g |
| hhx01 | 132460693 | 132462096 | transcript | HHX010036234.g |
| hhx10 | 62272770 | 62281556 | transcript | HHX100022776.g |
| hhx11 | 88833381 | 88848388 | transcript | HHX110052875.g |
| hhx05 | 63769878 | 63783699 | transcript | HHX050045946.g |
| hhx01 | 3530433 | 3532836 | transcript | HHX010032013.g |
| hhx04 | 150995637 | 151002153 | transcript | HHX040031593.g |
| hhx16 | 64403736 | 64411112 | transcript | HHX160063780.g |
| hhx14 | 19460614 | 19461906 | transcript | HHX140040949.g |
| hhx10 | 7161812 | 7163076 | transcript | HHX100019838.g |
| hhx10 | 7162630 | 7162765 | exon | HHX100019838.g |
| hhx10 | 7162630 | 7162765 | CDS | HHX100019838.g |
| hhx04 | 69076622 | 69094600 | transcript | HHX040027124.g |
| hhx03 | 65383661 | 65384038 | exon | HHX030014515.g |
| hhx03 | 65383661 | 65384038 | CDS | HHX030014515.g |
| hhx03 | 65377853 | 65388759 | transcript | HHX030014515.g |
| hhx01 | 137803293 | 137812115 | transcript | HHX010036543.g |
| hhx01 | 153365032 | 153379692 | transcript | HHX010037429.g |
| hhx05 | 143562378 | 143570018 | transcript | HHX050048667.g |
| hhx03 | 15353378 | 15368764 | transcript | HHX030011852.g |
| hhx07 | 39397792 | 39399188 | exon | HHX070056501.g |
| hhx07 | 39397194 | 39416893 | transcript | HHX070056501.g |
| hhx10 | 26945371 | 26948946 | transcript | HHX100020869.g |
| hhx05 | 79517023 | 79534005 | transcript | HHX050046769.g |
| hhx13 | 85008236 | 85015274 | transcript | HHX130040061.g |
| hhx11 | 13931962 | 13932906 | transcript | HHX110051075.g |
| hhx05 | 61003291 | 61016453 | transcript | HHX050045786.g |
| hhx04 | 70013440 | 70026614 | transcript | HHX040027181.g |
| hhx11 | 1615038 | 1625750 | transcript | HHX110050413.g |
| hhx14 | 19337770 | 19346691 | transcript | HHX140040939.g |
| hhx08 | 77705297 | 77724303 | transcript | HHX080004892.g |
| hhx01 | 118904331 | 118921311 | transcript | HHX010035432.g |
| hhx02 | 18741825 | 18751663 | transcript | HHX020007438.g |
| hhx04 | 99194127 | 99208529 | transcript | HHX040028769.g |
| hhx05 | 67349491 | 67352580 | transcript | HHX050046110.g |
| hhx03 | 59772376 | 59784522 | transcript | HHX030014302.g |
| hhx12 | 4146912 | 4156710 | transcript | HHX120000172.g |
| hhx10 | 61752834 | 61758234 | transcript | HHX100022748.g |
| hhx18 | 36645404 | 36650569 | transcript | HHX180060597.g |
| hhx05 | 74963941 | 74975238 | transcript | HHX050046492.g |
| hhx08 | 96219240 | 96234807 | transcript | HHX080005702.g |
| hhx04 | 128492305 | 128516882 | transcript | HHX040030283.g |
| hhx05 | 141295743 | 141304549 | transcript | HHX050048535.g |
| hhx04 | 113570542 | 113572204 | transcript | HHX040029547.g |
| hhx12 | 61133218 | 61136643 | transcript | HHX120001931.g |
| hhx10 | 36342859 | 36353468 | transcript | HHX100021406.g |
| hhx16 | 2064486 | 2077940 | transcript | HHX160062600.g |
| hhx14 | 26184006 | 26198680 | transcript | HHX140041355.g |
| hhx02 | 55959603 | 55967253 | transcript | HHX020009379.g |
| hhx04 | 116522047 | 116526999 | transcript | HHX040029712.g |
| hhx04 | 116522047 | 116523308 | exon | HHX040029712.g |
| hhx08 | 106831517 | 106839073 | transcript | HHX080006460.g |
| hhx01 | 115054234 | 115055357 | transcript | HHX010035232.g |
| hhx01 | 121252452 | 121253103 | transcript | HHX010035582.g |
| hhx03 | 28548170 | 28571635 | transcript | HHX030012665.g |
| hhx13 | 710631 | 711839 | transcript | HHX130038348.g |
| hhx13 | 710631 | 710966 | exon | HHX130038348.g |
| hhx13 | 710631 | 710966 | CDS | HHX130038348.g |
| hhx10 | 49468939 | 49477708 | transcript | HHX100022153.g |
| hhx05 | 76699538 | 76719101 | transcript | HHX050046590.g |
| hhx02 | 30061153 | 30062110 | transcript | HHX020008009.g |
| hhx16 | 50835068 | 50866686 | transcript | HHX160063483.g |
| hhx07 | 26083737 | 26084876 | transcript | HHX070055768.g |
| hhx08 | 90096823 | 90109710 | transcript | HHX080005452.g |
| hhx11 | 47824566 | 47830384 | transcript | HHX110051883.g |
| hhx05 | 137183307 | 137199437 | transcript | HHX050048359.g |
| hhx01 | 100590862 | 100610918 | transcript | HHX010034510.g |
| hhx07 | 42886751 | 42939278 | transcript | HHX070056675.g |
| hhx01 | 123846344 | 123849255 | transcript | HHX010035752.g |
| hhx04 | 2612484 | 2621207 | transcript | HHX040025315.g |
| hhx16 | 68138881 | 68152096 | transcript | HHX160063937.g |
| hhx02 | 47802818 | 47814218 | transcript | HHX020008956.g |
| hhx09 | 14496 | 17729 | transcript | HHX090023806.g |
| hhx16 | 70211803 | 70245661 | transcript | HHX160064043.g |
| hhx07 | 42886751 | 42939278 | transcript | HHX070056675.g |
| hhx02 | 23202806 | 23215468 | transcript | HHX020007638.g |
| hhx13 | 79989752 | 79997550 | transcript | HHX130039763.g |
| hhx05 | 63820367 | 63830884 | transcript | HHX050045949.g |
| hhx02 | 51247826 | 51257582 | transcript | HHX020009167.g |
| hhx05 | 75317106 | 75339828 | transcript | HHX050046513.g |
| hhx04 | 109893967 | 109899574 | transcript | HHX040029345.g |
| hhx10 | 27011449 | 27019388 | transcript | HHX100020874.g |
| hhx05 | 147723119 | 147729754 | transcript | HHX050048884.g |
| hhx01 | 108081489 | 108088803 | transcript | HHX010034897.g |
| hhx01 | 80688017 | 80693568 | transcript | HHX010033874.g |
| hhx06 | 114943097 | 114953729 | transcript | HHX060018642.g |
| hhx04 | 38710852 | 38721176 | transcript | HHX040025946.g |
| hhx07 | 51962401 | 51970814 | transcript | HHX070057245.g |
| hhx02 | 34813587 | 34813840 | transcript | HHX020008262.g |
| hhx02 | 34813587 | 34813738 | exon | HHX020008262.g |
| hhx02 | 34813587 | 34813738 | CDS | HHX020008262.g |
| hhx04 | 78295879 | 78317787 | transcript | HHX040027679.g |
| hhx01 | 115028061 | 115039748 | transcript | HHX010035226.g |
| hhx07 | 45160368 | 45186350 | transcript | HHX070056755.g |
| hhx01 | 93149966 | 93165589 | transcript | HHX010034179.g |
| hhx05 | 61291665 | 61312918 | transcript | HHX050045797.g |
| hhx03 | 22076505 | 22091363 | transcript | HHX030012291.g |
| hhx06 | 37213744 | 37221703 | transcript | HHX060017204.g |
| hhx04 | 131316374 | 131335246 | transcript | HHX040030420.g |
| hhx04 | 114624471 | 114638002 | transcript | HHX040029610.g |
| hhx04 | 120429012 | 120435499 | transcript | HHX040029918.g |
| hhx03 | 154894888 | 154899804 | transcript | HHX030016034.g |
| hhx14 | 50690054 | 50697629 | transcript | HHX140041985.g |
| hhx03 | 69557539 | 69562972 | transcript | HHX030014683.g |
| hhx04 | 75114994 | 75115215 | exon | HHX040027484.g |
| hhx04 | 75114994 | 75115215 | CDS | HHX040027484.g |
| hhx04 | 75114431 | 75121478 | transcript | HHX040027484.g |
| hhx08 | 76080638 | 76092614 | transcript | HHX080004805.g |
| hhx14 | 17373883 | 17380639 | transcript | HHX140040832.g |
| hhx04 | 104928314 | 104933898 | transcript | HHX040029100.g |
| hhx04 | 128111129 | 128120102 | transcript | HHX040030261.g |
| hhx10 | 19757872 | 19769084 | transcript | HHX100020472.g |
| hhx12 | 66469180 | 66474366 | transcript | HHX120002202.g |
| hhx05 | 151212178 | 151219829 | transcript | HHX050049126.g |
| hhx07 | 97042860 | 97062418 | transcript | HHX070059017.g |
| hhx13 | 72050914 | 72056967 | transcript | HHX130039439.g |
| hhx01 | 98029140 | 98037249 | transcript | HHX010034415.g |
| hhx01 | 154515353 | 154521981 | transcript | HHX010037466.g |
| hhx03 | 57750264 | 57751639 | transcript | HHX030014152.g |
| hhx03 | 57750808 | 57751026 | exon | HHX030014152.g |
| hhx03 | 57750808 | 57751026 | CDS | HHX030014152.g |
| hhx05 | 65582181 | 65598122 | transcript | HHX050046028.g |
| hhx16 | 1917875 | 1931346 | transcript | HHX160062597.g |
| hhx16 | 69383769 | 69391443 | transcript | HHX160064005.g |
| hhx03 | 70393123 | 70412694 | transcript | HHX030014729.g |
| hhx01 | 1964528 | 1975241 | transcript | HHX010031863.g |
| hhx01 | 4026013 | 4043565 | transcript | HHX010032053.g |
| hhx04 | 139653229 | 139653854 | transcript | HHX040030939.g |
| hhx13 | 24699301 | 24708222 | transcript | HHX130038751.g |
| hhx04 | 143080861 | 143092194 | transcript | HHX040031174.g |
| hhx16 | 71319546 | 71327980 | transcript | HHX160064118.g |
| hhx07 | 63373389 | 63382152 | transcript | HHX070057760.g |
| hhx08 | 95191479 | 95200271 | transcript | HHX080005679.g |
| hhx12 | 58894494 | 58902574 | transcript | HHX120001816.g |
| hhx12 | 19780650 | 19792302 | transcript | HHX120000598.g |
| hhx05 | 68711049 | 68718617 | transcript | HHX050046181.g |
| hhx12 | 4794531 | 4803082 | transcript | HHX120000202.g |
| hhx09 | 16770811 | 16783429 | transcript | HHX090024432.g |
| hhx07 | 26544589 | 26545405 | transcript | HHX070055807.g |
| hhx12 | 66035982 | 66046405 | transcript | HHX120002187.g |
| hhx11 | 84743381 | 84752838 | transcript | HHX110052639.g |
| hhx08 | 73251951 | 73257499 | transcript | HHX080004649.g |
| hhx04 | 105885 | 116370 | transcript | HHX040025233.g |
| hhx02 | 35061205 | 35064882 | transcript | HHX020008272.g |
| hhx10 | 36228813 | 36230505 | exon | HHX100021399.g |
| hhx10 | 36217699 | 36230505 | transcript | HHX100021399.g |
| hhx04 | 128203062 | 128227321 | transcript | HHX040030268.g |
| hhx07 | 25532458 | 25548509 | transcript | HHX070055741.g |
| hhx10 | 20149170 | 20152322 | transcript | HHX100020492.g |
| hhx01 | 109958263 | 109960718 | transcript | HHX010035010.g |
| hhx04 | 150998890 | 150998976 | transcript | HHX040031593.g |
| hhx14 | 19341838 | 19345265 | transcript | HHX140040939.g |
| hhx03 | 154898313 | 154898385 | transcript | HHX030016034.g |

**C.**

| ***Michelia champaca*_CHH** | | | | |
| --- | --- | --- | --- | --- |
| hhx10 | 47879264 | 47897105 | transcript | HHX100022062.g |
| hhx01 | 127160922 | 127191549 | transcript | HHX010035936.g |
| hhx10 | 30023741 | 30036751 | transcript | HHX100021029.g |
| hhx07 | 28917586 | 28936260 | transcript | HHX070055879.g |
| hhx04 | 98091333 | 98124009 | transcript | HHX040028724.g |
| hhx03 | 160173852 | 160207597 | transcript | HHX030016134.g |
| hhx02 | 25258210 | 25272662 | transcript | HHX020007739.g |
| hhx10 | 15989820 | 16003118 | transcript | HHX100020294.g |
| hhx04 | 71723640 | 71751119 | transcript | HHX040027306.g |
| hhx04 | 98911667 | 98925714 | transcript | HHX040028757.g |
| hhx06 | 100195448 | 100207248 | transcript | HHX060018142.g |
| hhx10 | 75566343 | 75597396 | transcript | HHX100023380.g |
| hhx17 | 40084340 | 40097390 | transcript | HHX170049868.g |
| hhx11 | 9071849 | 9098921 | transcript | HHX110050772.g |
| hhx03 | 53322587 | 53333956 | transcript | HHX030013935.g |
| hhx15 | 3281385 | 3288471 | transcript | HHX150061299.g |
| hhx05 | 145812204 | 145825791 | transcript | HHX050048764.g |
| hhx04 | 114555322 | 114595360 | transcript | HHX040029604.g |
| hhx10 | 5231731 | 5244517 | transcript | HHX100019716.g |
| hhx01 | 497988 | 517000 | transcript | HHX010031774.g |
| hhx05 | 66916384 | 66946651 | transcript | HHX050046080.g |
| hhx03 | 50369223 | 50392222 | transcript | HHX030013801.g |
| hhx03 | 31975638 | 31994641 | transcript | HHX030012884.g |
| hhx07 | 39397194 | 39416893 | transcript | HHX070056501.g |
| hhx11 | 88882790 | 88898214 | transcript | HHX110052877.g |
| hhx01 | 137999394 | 138011660 | transcript | HHX010036558.g |
| hhx02 | 44601738 | 44607374 | transcript | HHX020008807.g |
| hhx02 | 73120289 | 73136212 | transcript | HHX020009753.g |
| hhx08 | 76420786 | 76428542 | transcript | HHX080004820.g |
| hhx07 | 34427565 | 34432917 | transcript | HHX070056180.g |
| hhx04 | 2796601 | 2811029 | transcript | HHX040025317.g |
| hhx08 | 81497445 | 81525881 | transcript | HHX080005094.g |
| hhx10 | 7918348 | 7939460 | transcript | HHX100019862.g |
| hhx02 | 52823633 | 52863659 | transcript | HHX020009239.g |
| hhx10 | 41472341 | 41488209 | transcript | HHX100021709.g |
| hhx05 | 5001883 | 5010825 | transcript | HHX050044303.g |
| hhx03 | 36421476 | 36439779 | transcript | HHX030013120.g |
| hhx14 | 19485667 | 19495797 | transcript | HHX140040951.g |
| hhx11 | 65684584 | 65700277 | transcript | HHX110052084.g |
| hhx01 | 497988 | 517000 | transcript | HHX010031774.g |
| hhx11 | 80906108 | 80927852 | transcript | HHX110052474.g |
| hhx18 | 40620188 | 40642499 | transcript | HHX180060855.g |
| hhx14 | 22395715 | 22407132 | transcript | HHX140041136.g |
| hhx12 | 50667268 | 50682168 | transcript | HHX120001400.g |
| hhx08 | 75587773 | 75606639 | transcript | HHX080004783.g |
| hhx12 | 72139946 | 72170616 | transcript | HHX120002390.g |
| hhx05 | 4849465 | 4864406 | transcript | HHX050044294.g |
| hhx03 | 17128698 | 17134410 | transcript | HHX030011951.g |
| hhx11 | 51228852 | 51239900 | transcript | HHX110051921.g |
| hhx01 | 125246822 | 125259084 | transcript | HHX010035828.g |
| hhx04 | 114555322 | 114595360 | transcript | HHX040029604.g |
| hhx07 | 24959530 | 24987359 | transcript | HHX070055718.g |
| hhx01 | 137321517 | 137328666 | transcript | HHX010036516.g |
| hhx07 | 98602735 | 98615266 | transcript | HHX070059123.g |
| hhx10 | 40170402 | 40180006 | transcript | HHX100021613.g |
| hhx04 | 77609889 | 77616190 | transcript | HHX040027646.g |
| hhx10 | 64427683 | 64444763 | transcript | HHX100022901.g |
| hhx12 | 56201158 | 56208501 | transcript | HHX120001723.g |
| hhx01 | 3578567 | 3587671 | transcript | HHX010032017.g |
| hhx01 | 127160922 | 127191549 | transcript | HHX010035936.g |
| hhx03 | 44024975 | 44033975 | transcript | HHX030013497.g |
| hhx07 | 99834731 | 99852223 | transcript | HHX070059170.g |
| hhx16 | 59813773 | 59848514 | transcript | HHX160063646.g |
| hhx02 | 67912560 | 67923979 | transcript | HHX020009697.g |
| hhx12 | 61639034 | 61650562 | transcript | HHX120001953.g |
| hhx04 | 89662247 | 89668690 | transcript | HHX040028301.g |
| hhx07 | 61630162 | 61638312 | transcript | HHX070057704.g |
| hhx14 | 13053095 | 13058352 | transcript | HHX140040571.g |
| hhx14 | 71193419 | 71219058 | transcript | HHX140043126.g |
| hhx10 | 29000539 | 29014402 | transcript | HHX100020973.g |
| hhx01 | 94966104 | 94981354 | transcript | HHX010034252.g |
| hhx05 | 20266899 | 20279223 | transcript | HHX050045046.g |
| hhx11 | 9430239 | 9474952 | transcript | HHX110050785.g |
| hhx10 | 64455490 | 64469094 | transcript | HHX100022902.g |
| hhx04 | 118601996 | 118614837 | transcript | HHX040029828.g |
| hhx03 | 47721109 | 47728830 | transcript | HHX030013673.g |
| hhx10 | 41417974 | 41429221 | transcript | HHX100021701.g |
| hhx02 | 5851749 | 5872302 | transcript | HHX020006916.g |
| hhx04 | 60708360 | 60710763 | transcript | HHX040026660.g |
| hhx02 | 53715797 | 53735212 | transcript | HHX020009279.g |
| hhx15 | 72931421 | 72946065 | transcript | HHX150062401.g |
| hhx12 | 59964437 | 59985881 | transcript | HHX120001880.g |
| hhx11 | 78667182 | 78691488 | transcript | HHX110052373.g |
| hhx04 | 133670530 | 133695671 | transcript | HHX040030567.g |
| hhx10 | 66140648 | 66155133 | transcript | HHX100022983.g |
| hhx12 | 63176391 | 63178908 | transcript | HHX120002032.g |
| hhx04 | 126969042 | 126994694 | transcript | HHX040030218.g |
| hhx10 | 69570362 | 69573966 | transcript | HHX100023135.g |
| hhx14 | 63654120 | 63662160 | transcript | HHX140042651.g |
| hhx02 | 41958764 | 41959670 | transcript | HHX020008639.g |
| hhx07 | 23957089 | 23964681 | transcript | HHX070055654.g |
| hhx02 | 38449824 | 38463669 | transcript | HHX020008440.g |
| hhx04 | 120445877 | 120450277 | transcript | HHX040029919.g |
| hhx01 | 6396942 | 6404192 | transcript | HHX010032195.g |
| hhx03 | 102056716 | 102068166 | transcript | HHX030015316.g |
| hhx10 | 44269997 | 44284081 | transcript | HHX100021862.g |
| hhx05 | 80356432 | 80392066 | transcript | HHX050046814.g |
| hhx06 | 33465080 | 33478542 | transcript | HHX060017129.g |
| hhx03 | 35997550 | 36010117 | transcript | HHX030013098.g |
| hhx10 | 11012903 | 11019706 | transcript | HHX100020013.g |
| hhx01 | 105409980 | 105464071 | transcript | HHX010034754.g |
| hhx10 | 16538298 | 16540959 | transcript | HHX100020321.g |
| hhx07 | 42754482 | 42755603 | transcript | HHX070056667.g |
| hhx11 | 82752880 | 82763464 | transcript | HHX110052569.g |
| hhx07 | 97042860 | 97062418 | transcript | HHX070059017.g |
| hhx05 | 149959289 | 149972603 | transcript | HHX050049032.g |
| hhx04 | 79059425 | 79066557 | transcript | HHX040027711.g |
| hhx03 | 161354691 | 161362253 | transcript | HHX030016173.g |
| hhx12 | 8362728 | 8367375 | transcript | HHX120000308.g |
| hhx07 | 52909270 | 52929279 | transcript | HHX070057298.g |
| hhx04 | 139170355 | 139171176 | transcript | HHX040030914.g |
| hhx02 | 32821446 | 32829814 | transcript | HHX020008155.g |
| hhx05 | 110025373 | 110036869 | transcript | HHX050047846.g |
| hhx13 | 78717472 | 78729813 | transcript | HHX130039690.g |
| hhx16 | 3556670 | 3573659 | transcript | HHX160062673.g |
| hhx04 | 122602505 | 122611942 | transcript | HHX040030043.g |
| hhx03 | 53322587 | 53333956 | transcript | HHX030013935.g |
| hhx04 | 95112637 | 95135473 | transcript | HHX040028564.g |
| hhx16 | 59813773 | 59848514 | transcript | HHX160063646.g |
| hhx10 | 17811178 | 17812396 | transcript | HHX100020399.g |
| hhx03 | 21891477 | 21899830 | transcript | HHX030012282.g |
| hhx13 | 27185489 | 27197433 | transcript | HHX130038776.g |
| hhx03 | 49080199 | 49091343 | transcript | HHX030013728.g |
| hhx04 | 61601329 | 61604543 | transcript | HHX040026703.g |
| hhx11 | 925743 | 933216 | transcript | HHX110050363.g |
| hhx05 | 22472092 | 22473809 | transcript | HHX050045096.g |
| hhx04 | 82295452 | 82313118 | transcript | HHX040027917.g |
| hhx07 | 48845985 | 48858202 | transcript | HHX070057011.g |
| hhx13 | 78243798 | 78250196 | transcript | HHX130039658.g |
| hhx05 | 107046217 | 107066092 | transcript | HHX050047785.g |
| hhx10 | 29227822 | 29234941 | transcript | HHX100020986.g |
| hhx03 | 66005511 | 66015682 | transcript | HHX030014541.g |
| hhx01 | 145860434 | 145879589 | transcript | HHX010037040.g |
| hhx01 | 122639922 | 122653106 | transcript | HHX010035662.g |
| hhx02 | 39453038 | 39466512 | transcript | HHX020008489.g |
| hhx10 | 28045851 | 28050121 | transcript | HHX100020929.g |
| hhx02 | 34550087 | 34559195 | transcript | HHX020008247.g |
| hhx10 | 31529930 | 31538221 | transcript | HHX100021099.g |
| hhx03 | 8714807 | 8728193 | transcript | HHX030011540.g |
| hhx10 | 5433743 | 5444999 | transcript | HHX100019728.g |
| hhx01 | 173282487 | 173285768 | transcript | HHX010037850.g |
| hhx03 | 45831104 | 45840799 | transcript | HHX030013574.g |
| hhx05 | 58527129 | 58539798 | transcript | HHX050045698.g |
| hhx07 | 61756377 | 61790047 | transcript | HHX070057710.g |
| hhx12 | 11664969 | 11681351 | transcript | HHX120000423.g |
| hhx01 | 122327937 | 122334144 | transcript | HHX010035645.g |
| hhx01 | 107337505 | 107355258 | transcript | HHX010034870.g |
| hhx03 | 32017011 | 32021995 | transcript | HHX030012887.g |
| hhx01 | 67042477 | 67046840 | transcript | HHX010033598.g |
| hhx05 | 135405675 | 135421569 | transcript | HHX050048284.g |
| hhx03 | 42096424 | 42112421 | transcript | HHX030013416.g |
| hhx19 | 4456492 | 4469261 | transcript | HHX190053153.g |
| hhx03 | 29836216 | 29847089 | transcript | HHX030012756.g |
| hhx12 | 54926421 | 54937235 | transcript | HHX120001652.g |
| hhx05 | 89541055 | 89556422 | transcript | HHX050047275.g |
| hhx05 | 146849041 | 146861254 | transcript | HHX050048836.g |
| hhx04 | 68757832 | 68761443 | transcript | HHX040027092.g |
| hhx12 | 54394486 | 54405193 | transcript | HHX120001621.g |
| hhx19 | 11060618 | 11080281 | transcript | HHX190053445.g |
| hhx01 | 129986600 | 130017383 | transcript | HHX010036108.g |
| hhx17 | 49482484 | 49487437 | transcript | HHX170050167.g |
| hhx04 | 71407463 | 71439178 | transcript | HHX040027277.g |
| hhx14 | 3840168 | 3850098 | transcript | HHX140040257.g |
| hhx07 | 105089833 | 105114471 | transcript | HHX070059414.g |
| hhx10 | 39484692 | 39491133 | transcript | HHX100021586.g |
| hhx08 | 69013071 | 69027028 | transcript | HHX080004336.g |
| hhx03 | 40914323 | 40924084 | transcript | HHX030013351.g |
| hhx05 | 56969725 | 56974483 | transcript | HHX050045662.g |
| hhx16 | 72190660 | 72223825 | transcript | HHX160064158.g |
| hhx18 | 31488489 | 31489425 | transcript | HHX180060407.g |
| hhx07 | 44558868 | 44564257 | transcript | HHX070056724.g |
| hhx03 | 764923 | 768124 | transcript | HHX030011079.g |
| hhx10 | 41982356 | 41995714 | transcript | HHX100021736.g |
| hhx10 | 57489827 | 57492057 | transcript | HHX100022544.g |
| hhx03 | 40537163 | 40543921 | transcript | HHX030013309.g |
| hhx07 | 89879621 | 89887045 | transcript | HHX070058575.g |
| hhx03 | 22425813 | 22437326 | transcript | HHX030012308.g |
| hhx04 | 56868258 | 56868524 | exon | HHX040026506.g |
| hhx04 | 56868258 | 56868524 | CDS | HHX040026506.g |
| hhx04 | 56868258 | 56869925 | transcript | HHX040026506.g |
| hhx07 | 60602343 | 60613803 | transcript | HHX070057653.g |
| hhx14 | 15143006 | 15147432 | transcript | HHX140040691.g |
| hhx04 | 105702935 | 105711421 | transcript | HHX040029133.g |
| hhx01 | 104184230 | 104189888 | transcript | HHX010034699.g |
| hhx08 | 62066164 | 62073511 | transcript | HHX080004040.g |
| hhx10 | 53254194 | 53264987 | transcript | HHX100022350.g |
| hhx01 | 131548800 | 131558381 | transcript | HHX010036180.g |
| hhx05 | 148105203 | 148140191 | transcript | HHX050048907.g |
| hhx05 | 68027885 | 68037215 | transcript | HHX050046146.g |
| hhx12 | 83769456 | 83769783 | transcript | HHX120002739.g |
| hhx12 | 83769678 | 83769783 | exon | HHX120002739.g |
| hhx12 | 83769678 | 83769783 | CDS | HHX120002739.g |
| hhx12 | 58542141 | 58547027 | transcript | HHX120001794.g |
| hhx10 | 7918348 | 7939460 | transcript | HHX100019862.g |
| hhx01 | 124870494 | 124881031 | transcript | HHX010035803.g |
| hhx08 | 86502159 | 86517078 | transcript | HHX080005337.g |
| hhx16 | 65255898 | 65258874 | transcript | HHX160063810.g |
| hhx07 | 52909270 | 52929279 | transcript | HHX070057298.g |
| hhx01 | 128544081 | 128555540 | transcript | HHX010036029.g |
| hhx05 | 4075575 | 4075900 | transcript | HHX050044230.g |
| hhx14 | 61264208 | 61265349 | transcript | HHX140042481.g |
| hhx01 | 100512067 | 100512519 | transcript | HHX010034508.g |
| hhx01 | 100512067 | 100512519 | exon | HHX010034508.g |
| hhx01 | 100512067 | 100512519 | CDS | HHX010034508.g |
| hhx17 | 40084340 | 40097390 | transcript | HHX170049868.g |
| hhx17 | 27987242 | 27994084 | transcript | HHX170049573.g |
| hhx07 | 74684489 | 74684794 | transcript | HHX070058136.g |
| hhx07 | 74684489 | 74684794 | exon | HHX070058136.g |
| hhx07 | 74684489 | 74684794 | CDS | HHX070058136.g |
| hhx11 | 51501926 | 51517813 | transcript | HHX110051924.g |
| hhx16 | 1811038 | 1831303 | transcript | HHX160062593.g |
| hhx10 | 84150802 | 84160301 | transcript | HHX100023593.g |
| hhx08 | 55137029 | 55148443 | transcript | HHX080003758.g |
| hhx01 | 127968271 | 127980344 | transcript | HHX010035996.g |
| hhx10 | 36048203 | 36055970 | transcript | HHX100021392.g |
| hhx13 | 82643029 | 82656957 | transcript | HHX130039909.g |
| hhx13 | 83537112 | 83547619 | transcript | HHX130039960.g |
| hhx16 | 59813773 | 59848514 | transcript | HHX160063646.g |
| hhx04 | 128611032 | 128612131 | transcript | HHX040030295.g |
| hhx04 | 128611937 | 128612131 | exon | HHX040030295.g |
| hhx04 | 128611937 | 128612131 | CDS | HHX040030295.g |
| hhx07 | 45680679 | 45691618 | transcript | HHX070056791.g |
| hhx07 | 45680679 | 45681397 | exon | HHX070056791.g |
| hhx14 | 24150394 | 24152152 | transcript | HHX140041246.g |
| hhx02 | 54106999 | 54124583 | transcript | HHX020009299.g |
| hhx18 | 7741060 | 7763143 | transcript | HHX180059661.g |
| hhx01 | 155249979 | 155259669 | transcript | HHX010037485.g |
| hhx02 | 18517243 | 18518800 | transcript | HHX020007431.g |
| hhx03 | 27344365 | 27355573 | transcript | HHX030012587.g |
| hhx04 | 119483658 | 119500649 | transcript | HHX040029869.g |
| hhx08 | 61216924 | 61222366 | transcript | HHX080004025.g |
| hhx16 | 5312378 | 5313513 | transcript | HHX160062729.g |
| hhx05 | 87428731 | 87431295 | transcript | HHX050047149.g |
| hhx03 | 46487507 | 46494661 | transcript | HHX030013606.g |
| hhx03 | 34621157 | 34626840 | transcript | HHX030013021.g |
| hhx11 | 1862374 | 1868894 | transcript | HHX110050428.g |
| hhx04 | 111464503 | 111479531 | transcript | HHX040029423.g |
| hhx05 | 88167729 | 88182731 | transcript | HHX050047193.g |
| hhx02 | 38449824 | 38463669 | transcript | HHX020008440.g |
| hhx17 | 5607425 | 5613519 | transcript | HHX170049316.g |
| hhx17 | 5610940 | 5611482 | exon | HHX170049316.g |
| hhx17 | 5610940 | 5611482 | CDS | HHX170049316.g |
| hhx05 | 66973256 | 66981371 | transcript | HHX050046083.g |
| hhx08 | 103510804 | 103534467 | transcript | HHX080006165.g |
| hhx05 | 349907 | 350762 | transcript | HHX050043989.g |
| hhx07 | 96885 | 99753 | transcript | HHX070054403.g |
| hhx02 | 35821917 | 35847247 | transcript | HHX020008315.g |
| hhx10 | 17811719 | 17811902 | transcript | HHX100020399.g |
| hhx05 | 107052340 | 107052733 | transcript | HHX050047785.g |
| hhx12 | 54395217 | 54395284 | transcript | HHX120001621.g |
| hhx10 | 39486663 | 39490501 | transcript | HHX100021586.g |
| hhx01 | 131553994 | 131554148 | transcript | HHX010036180.g |

**D.**

| ***Michelia montaua*_CG** | | | | |
| --- | --- | --- | --- | --- |
| SSHX01 | 110938745 | 110952727 | transcript | SSHX010064546.g |
| SSHX17 | 42114174 | 42126071 | transcript | SSHX170043728.g |
| SSHX01 | 20094578 | 20098552 | transcript | SSHX010062108.g |
| SSHX02 | 152495230 | 152502844 | transcript | SSHX020048154.g |
| SSHX13 | 3823954 | 3836121 | transcript | SSHX130018743.g |
| SSHX17 | 10972012 | 10991301 | transcript | SSHX170042169.g |
| SSHX09 | 31621726 | 31629685 | transcript | SSHX090023199.g |
| SSHX13 | 17346136 | 17356074 | transcript | SSHX130019618.g |
| SSHX14 | 26183608 | 26190404 | transcript | SSHX140010053.g |
| SSHX12 | 77216015 | 77229239 | transcript | SSHX120002892.g |
| SSHX11 | 38529919 | 38539996 | transcript | SSHX110025876.g |
| SSHX17 | 18548550 | 18559648 | transcript | SSHX170042586.g |
| SSHX18 | 61566701 | 61577074 | transcript | SSHX180015291.g |
| SSHX03 | 11357485 | 11369960 | transcript | SSHX030029261.g |
| SSHX06 | 122743113 | 122767442 | transcript | SSHX060052783.g |
| SSHX05 | 94611351 | 94619119 | transcript | SSHX050036918.g |
| SSHX02 | 166326015 | 166335892 | transcript | SSHX020048870.g |
| SSHX15 | 78167621 | 78168325 | transcript | SSHX150018274.g |
| SSHX15 | 78167621 | 78168325 | exon | SSHX150018274.g |
| SSHX15 | 78167621 | 78168325 | CDS | SSHX150018274.g |
| SSHX07 | 6500606 | 6504595 | transcript | SSHX070053232.g |
| SSHX09 | 99414897 | 99420220 | transcript | SSHX090024901.g |
| SSHX02 | 167204047 | 167215050 | transcript | SSHX020048923.g |
| SSHX15 | 3387899 | 3394117 | transcript | SSHX150016166.g |
| SSHX02 | 71751804 | 71760158 | transcript | SSHX020046516.g |
| SSHX02 | 61168273 | 61172976 | transcript | SSHX020045886.g |
| SSHX16 | 76583278 | 76586591 | transcript | SSHX160061806.g |
| SSHX08 | 37540261 | 37558209 | transcript | SSHX080038677.g |
| SSHX18 | 72917092 | 72925190 | transcript | SSHX180015924.g |
| SSHX03 | 10280016 | 10290986 | transcript | SSHX030029165.g |
| SSHX10 | 4222797 | 4232134 | transcript | SSHX100056116.g |
| SSHX08 | 2870316 | 2878298 | transcript | SSHX080037719.g |
| SSHX14 | 17754112 | 17761998 | transcript | SSHX140009505.g |
| SSHX02 | 144101226 | 144112612 | transcript | SSHX020047835.g |
| SSHX15 | 9946184 | 9952213 | transcript | SSHX150016493.g |
| SSHX05 | 62698083 | 62702775 | transcript | SSHX050034981.g |
| SSHX01 | 124295367 | 124307052 | transcript | SSHX010065427.g |
| SSHX16 | 73487108 | 73496299 | transcript | SSHX160061610.g |
| SSHX01 | 60166954 | 60169437 | transcript | SSHX010063041.g |
| SSHX16 | 68816675 | 68818661 | transcript | SSHX160061322.g |
| SSHX17 | 29443282 | 29458640 | transcript | SSHX170043207.g |
| SSHX12 | 63469790 | 63475243 | transcript | SSHX120002042.g |
| SSHX10 | 75566222 | 75569412 | transcript | SSHX100057618.g |
| SSHX19 | 58705448 | 58717543 | transcript | SSHX190013722.g |
| SSHX13 | 45089967 | 45094090 | transcript | SSHX130020841.g |
| SSHX01 | 177079085 | 177085689 | transcript | SSHX010067083.g |
| SSHX03 | 6540667 | 6550020 | transcript | SSHX030028928.g |
| SSHX03 | 9445466 | 9457121 | transcript | SSHX030029088.g |
| SSHX17 | 2240168 | 2253080 | transcript | SSHX170041602.g |
| SSHX01 | 122029545 | 122048407 | transcript | SSHX010065279.g |
| SSHX09 | 14082205 | 14086722 | transcript | SSHX090022205.g |
| SSHX17 | 14004863 | 14005819 | transcript | SSHX170042329.g |
| SSHX15 | 69744721 | 69758495 | transcript | SSHX150017839.g |
| SSHX13 | 19071758 | 19073248 | transcript | SSHX130019676.g |
| SSHX01 | 122159917 | 122172923 | transcript | SSHX010065285.g |
| SSHX13 | 2211597 | 2214554 | transcript | SSHX130018625.g |
| SSHX04 | 50077439 | 50085802 | transcript | SSHX040004882.g |
| SSHX12 | 52276692 | 52283699 | transcript | SSHX120001493.g |
| SSHX05 | 65347211 | 65355346 | transcript | SSHX050035135.g |
| SSHX02 | 18426017 | 18434210 | transcript | SSHX020044444.g |
| SSHX10 | 93712350 | 93721166 | transcript | SSHX100058627.g |
| SSHX15 | 73641304 | 73655315 | transcript | SSHX150018016.g |
| SSHX03 | 137341500 | 137351304 | transcript | SSHX030032430.g |
| SSHX01 | 164621759 | 164624950 | transcript | SSHX010066710.g |
| SSHX02 | 13583959 | 13603855 | transcript | SSHX020044372.g |
| SSHX15 | 77769153 | 77781027 | transcript | SSHX150018233.g |
| SSHX08 | 111963538 | 111977598 | transcript | SSHX080041304.g |
| SSHX08 | 99685438 | 99689198 | transcript | SSHX080040691.g |
| SSHX04 | 33237572 | 33241159 | transcript | SSHX040003920.g |
| SSHX19 | 48500556 | 48516975 | transcript | SSHX190013073.g |
| SSHX10 | 2361232 | 2362167 | transcript | SSHX100055999.g |
| SSHX09 | 105969930 | 105977639 | transcript | SSHX090025291.g |
| SSHX02 | 161088040 | 161091218 | transcript | SSHX020048557.g |
| SSHX17 | 2870736 | 2878828 | transcript | SSHX170041642.g |
| SSHX04 | 41331103 | 41338418 | transcript | SSHX040004380.g |
| SSHX18 | 71296173 | 71297450 | transcript | SSHX180015799.g |
| SSHX18 | 71296976 | 71297450 | exon | SSHX180015799.g |
| SSHX18 | 71296976 | 71297450 | CDS | SSHX180015799.g |
| SSHX05 | 78385275 | 78396593 | transcript | SSHX050035941.g |
| SSHX07 | 115964769 | 115968060 | transcript | SSHX070055795.g |
| SSHX14 | 18812103 | 18815921 | transcript | SSHX140009545.g |
| SSHX14 | 18812103 | 18812535 | exon | SSHX140009545.g |
| SSHX14 | 18812103 | 18812535 | CDS | SSHX140009545.g |
| SSHX03 | 145208761 | 145218998 | transcript | SSHX030032845.g |
| SSHX15 | 66316917 | 66318660 | transcript | SSHX150017725.g |
| SSHX13 | 2294581 | 2302009 | transcript | SSHX130018633.g |
| SSHX12 | 50324512 | 50334158 | transcript | SSHX120001407.g |
| SSHX13 | 2264356 | 2265830 | transcript | SSHX130018631.g |
| SSHX01 | 182552507 | 182558471 | transcript | SSHX010067335.g |
| SSHX06 | 10140367 | 10142292 | transcript | SSHX060049631.g |
| SSHX03 | 25316616 | 25316854 | exon | SSHX030030108.g |
| SSHX03 | 25316616 | 25316854 | CDS | SSHX030030108.g |
| SSHX03 | 25308483 | 25319337 | transcript | SSHX030030108.g |
| SSHX10 | 87135228 | 87140384 | transcript | SSHX100058309.g |
| SSHX09 | 4744630 | 4757022 | transcript | SSHX090021635.g |
| SSHX01 | 63985020 | 63986651 | transcript | SSHX010063267.g |
| SSHX01 | 63986441 | 63986651 | exon | SSHX010063267.g |
| SSHX01 | 63986441 | 63986651 | CDS | SSHX010063267.g |
| SSHX14 | 16360996 | 16366965 | transcript | SSHX140009430.g |
| SSHX12 | 72576578 | 72585118 | transcript | SSHX120002610.g |
| SSHX07 | 86644491 | 86650180 | transcript | SSHX070054514.g |
| SSHX11 | 70089727 | 70097001 | transcript | SSHX110027511.g |
| SSHX06 | 9228936 | 9239153 | transcript | SSHX060049586.g |
| SSHX15 | 57491235 | 57499230 | transcript | SSHX150017404.g |
| SSHX04 | 124793731 | 124796952 | transcript | SSHX040007725.g |
| SSHX03 | 139510847 | 139511656 | transcript | SSHX030032527.g |
| SSHX03 | 139511093 | 139511475 | exon | SSHX030032527.g |
| SSHX03 | 139511093 | 139511475 | CDS | SSHX030032527.g |
| SSHX01 | 159845333 | 159859665 | transcript | SSHX010066613.g |
| SSHX18 | 51266882 | 51271615 | transcript | SSHX180014776.g |
| SSHX14 | 6772573 | 6780658 | transcript | SSHX140008860.g |
| SSHX12 | 63650384 | 63655129 | transcript | SSHX120002053.g |
| SSHX15 | 4614058 | 4623101 | transcript | SSHX150016237.g |
| SSHX09 | 106196187 | 106204825 | transcript | SSHX090025319.g |
| SSHX05 | 86670069 | 86678492 | transcript | SSHX050036472.g |
| SSHX16 | 52404909 | 52421692 | transcript | SSHX160060523.g |
| SSHX15 | 64108197 | 64109610 | transcript | SSHX150017657.g |
| SSHX13 | 16618824 | 16626820 | transcript | SSHX130019577.g |
| SSHX08 | 101420627 | 101438790 | transcript | SSHX080040769.g |
| SSHX02 | 114893954 | 114898304 | transcript | SSHX020047486.g |
| SSHX16 | 73299210 | 73307412 | transcript | SSHX160061600.g |
| SSHX11 | 53897982 | 53903164 | transcript | SSHX110026545.g |
| SSHX07 | 62310807 | 62317519 | transcript | SSHX070054125.g |
| SSHX08 | 10051291 | 10051707 | transcript | SSHX080037913.g |
| SSHX08 | 10051291 | 10051457 | exon | SSHX080037913.g |
| SSHX08 | 10051291 | 10051457 | CDS | SSHX080037913.g |
| SSHX16 | 63796713 | 63807651 | transcript | SSHX160061072.g |
| SSHX07 | 106733797 | 106744314 | transcript | SSHX070055385.g |
| SSHX01 | 175420260 | 175426570 | transcript | SSHX010067001.g |
| SSHX06 | 114527969 | 114542284 | transcript | SSHX060052327.g |
| SSHX12 | 53873832 | 53882007 | transcript | SSHX120001545.g |
| SSHX03 | 61427398 | 61428555 | transcript | SSHX030031119.g |
| SSHX05 | 65397700 | 65409186 | transcript | SSHX050035141.g |
| SSHX11 | 80832216 | 80849889 | transcript | SSHX110028081.g |
| SSHX17 | 23821270 | 23831361 | transcript | SSHX170042898.g |
| SSHX16 | 69979075 | 69987693 | transcript | SSHX160061397.g |
| SSHX12 | 75671556 | 75681286 | transcript | SSHX120002781.g |
| SSHX06 | 17370253 | 17379608 | transcript | SSHX060050022.g |
| SSHX01 | 108135906 | 108136775 | transcript | SSHX010064399.g |
| SSHX06 | 12111822 | 12114741 | transcript | SSHX060049750.g |
| SSHX08 | 108808157 | 108823384 | transcript | SSHX080041109.g |
| SSHX03 | 139820207 | 139823274 | transcript | SSHX030032547.g |
| SSHX08 | 37528733 | 37539134 | transcript | SSHX080038674.g |
| SSHX09 | 51243057 | 51249421 | transcript | SSHX090023641.g |
| SSHX04 | 101926357 | 101941347 | transcript | SSHX040006622.g |
| SSHX08 | 109210730 | 109215327 | transcript | SSHX080041127.g |
| SSHX03 | 15673061 | 15684184 | transcript | SSHX030029542.g |
| SSHX05 | 96897020 | 96901986 | transcript | SSHX050037060.g |
| SSHX11 | 84132172 | 84139202 | transcript | SSHX110028281.g |
| SSHX19 | 42785325 | 42788746 | transcript | SSHX190012760.g |
| SSHX14 | 22000271 | 22008835 | transcript | SSHX140009776.g |
| SSHX02 | 165292708 | 165308162 | transcript | SSHX020048817.g |
| SSHX02 | 161984534 | 161998677 | transcript | SSHX020048632.g |
| SSHX17 | 32654616 | 32663971 | transcript | SSHX170043357.g |
| SSHX09 | 15637880 | 15643726 | transcript | SSHX090022315.g |
| SSHX15 | 631091 | 644027 | transcript | SSHX150016026.g |
| SSHX02 | 161637864 | 161642463 | transcript | SSHX020048604.g |
| SSHX08 | 106930123 | 106942984 | transcript | SSHX080041007.g |
| SSHX11 | 38856761 | 38861326 | transcript | SSHX110025885.g |
| SSHX05 | 60695896 | 60699117 | transcript | SSHX050034784.g |
| SSHX11 | 54992699 | 55003930 | transcript | SSHX110026597.g |
| SSHX11 | 67726354 | 67728745 | transcript | SSHX110027366.g |
| SSHX11 | 67728224 | 67728470 | exon | SSHX110027366.g |
| SSHX11 | 67728224 | 67728470 | CDS | SSHX110027366.g |
| SSHX12 | 53601052 | 53619147 | transcript | SSHX120001525.g |
| SSHX05 | 39083343 | 39090969 | transcript | SSHX050033753.g |
| SSHX12 | 52207123 | 52208179 | transcript | SSHX120001489.g |
| SSHX09 | 40539990 | 40540791 | transcript | SSHX090023430.g |
| SSHX01 | 118399729 | 118419398 | transcript | SSHX010064985.g |
| SSHX03 | 148057358 | 148065555 | transcript | SSHX030033041.g |
| SSHX09 | 105958890 | 105969547 | transcript | SSHX090025290.g |
| SSHX13 | 10477320 | 10496097 | transcript | SSHX130019178.g |
| SSHX05 | 75059842 | 75067705 | transcript | SSHX050035735.g |
| SSHX03 | 17870220 | 17890545 | transcript | SSHX030029674.g |
| SSHX07 | 627898 | 638653 | transcript | SSHX070052881.g |
| SSHX08 | 105038264 | 105047249 | transcript | SSHX080040909.g |
| SSHX04 | 58049136 | 58056128 | transcript | SSHX040005472.g |
| SSHX14 | 32881095 | 32886232 | transcript | SSHX140010502.g |
| SSHX01 | 139605147 | 139610727 | transcript | SSHX010066337.g |
| SSHX01 | 118399729 | 118419398 | transcript | SSHX010064985.g |
| SSHX02 | 40441711 | 40452065 | transcript | SSHX020044815.g |
| SSHX07 | 102635585 | 102641590 | transcript | SSHX070055138.g |
| SSHX01 | 110662189 | 110668501 | transcript | SSHX010064533.g |
| SSHX04 | 100979234 | 100981157 | transcript | SSHX040006601.g |
| SSHX13 | 10745347 | 10755275 | transcript | SSHX130019189.g |
| SSHX04 | 54202546 | 54207291 | transcript | SSHX040005200.g |
| SSHX15 | 4635535 | 4637635 | transcript | SSHX150016239.g |
| SSHX11 | 57531573 | 57536478 | transcript | SSHX110026745.g |
| SSHX17 | 29443282 | 29458640 | transcript | SSHX170043207.g |
| SSHX18 | 58007921 | 58027399 | transcript | SSHX180015024.g |
| SSHX02 | 146770734 | 146780565 | transcript | SSHX020047937.g |
| SSHX11 | 39748157 | 39755300 | transcript | SSHX110025927.g |
| SSHX03 | 18012256 | 18014397 | transcript | SSHX030029683.g |
| SSHX11 | 69512187 | 69517326 | transcript | SSHX110027487.g |
| SSHX16 | 57064418 | 57068139 | transcript | SSHX160060766.g |
| SSHX08 | 112103609 | 112104520 | transcript | SSHX080041306.g |
| SSHX10 | 98443129 | 98451196 | transcript | SSHX100058896.g |
| SSHX11 | 66194645 | 66204132 | transcript | SSHX110027264.g |
| SSHX02 | 62231368 | 62238288 | transcript | SSHX020045946.g |
| SSHX17 | 25234115 | 25245170 | transcript | SSHX170042968.g |
| SSHX08 | 107444004 | 107453928 | transcript | SSHX080041042.g |
| SSHX12 | 77454450 | 77464035 | transcript | SSHX120002896.g |
| SSHX03 | 144877743 | 144886435 | transcript | SSHX030032822.g |
| SSHX01 | 188749969 | 188754641 | transcript | SSHX010067714.g |
| SSHX16 | 73712119 | 73714748 | transcript | SSHX160061616.g |
| SSHX06 | 15567359 | 15578633 | transcript | SSHX060049908.g |
| SSHX01 | 125525795 | 125529594 | transcript | SSHX010065516.g |
| SSHX04 | 38985930 | 39005506 | transcript | SSHX040004238.g |
| SSHX01 | 191810141 | 191813592 | transcript | SSHX010067932.g |
| SSHX05 | 40813234 | 40813833 | transcript | SSHX050033796.g |
| SSHX05 | 40813234 | 40813833 | exon | SSHX050033796.g |
| SSHX05 | 40813234 | 40813833 | CDS | SSHX050033796.g |
| SSHX10 | 80908570 | 80916291 | transcript | SSHX100057937.g |
| SSHX11 | 69363997 | 69370231 | transcript | SSHX110027478.g |
| SSHX08 | 1715826 | 1723702 | transcript | SSHX080037667.g |
| SSHX06 | 28539410 | 28543473 | transcript | SSHX060050566.g |
| SSHX09 | 73190597 | 73196410 | transcript | SSHX090023964.g |
| SSHX13 | 2271214 | 2282334 | transcript | SSHX130018632.g |
| SSHX19 | 51790328 | 51805615 | transcript | SSHX190013278.g |
| SSHX08 | 52388776 | 52395843 | transcript | SSHX080039588.g |
| SSHX06 | 5382214 | 5390309 | transcript | SSHX060049371.g |
| SSHX19 | 61787685 | 61790530 | transcript | SSHX190013929.g |
| SSHX05 | 67586725 | 67589305 | transcript | SSHX050035311.g |
| SSHX05 | 57801119 | 57805060 | transcript | SSHX050034597.g |
| SSHX09 | 25409999 | 25421736 | transcript | SSHX090022940.g |
| SSHX15 | 10447378 | 10452345 | transcript | SSHX150016523.g |
| SSHX08 | 77091431 | 77099634 | transcript | SSHX080040134.g |
| SSHX12 | 49870988 | 49871260 | exon | SSHX120001393.g |
| SSHX12 | 49870988 | 49871260 | CDS | SSHX120001393.g |
| SSHX12 | 49867702 | 49873715 | transcript | SSHX120001393.g |
| SSHX04 | 51136186 | 51142709 | transcript | SSHX040004961.g |
| SSHX19 | 39306310 | 39309587 | transcript | SSHX190012601.g |
| SSHX14 | 42362342 | 42364366 | transcript | SSHX140010989.g |
| SSHX06 | 10491055 | 10503214 | transcript | SSHX060049669.g |
| SSHX08 | 34693976 | 34705880 | transcript | SSHX080038474.g |
| SSHX01 | 58259266 | 58261243 | transcript | SSHX010062974.g |
| SSHX08 | 2024857 | 2033534 | transcript | SSHX080037681.g |
| SSHX05 | 73319510 | 73327901 | transcript | SSHX050035638.g |
| SSHX10 | 94477562 | 94483293 | transcript | SSHX100058669.g |
| SSHX09 | 102421029 | 102430616 | transcript | SSHX090025060.g |
| SSHX17 | 12578005 | 12591017 | transcript | SSHX170042264.g |
| SSHX01 | 15227970 | 15231046 | transcript | SSHX010062073.g |
| SSHX05 | 82796769 | 82805222 | transcript | SSHX050036242.g |
| SSHX18 | 68443383 | 68450137 | transcript | SSHX180015636.g |
| SSHX02 | 64981217 | 64989481 | transcript | SSHX020046120.g |
| SSHX03 | 1121291 | 1134764 | transcript | SSHX030028552.g |
| SSHX19 | 42833212 | 42833834 | transcript | SSHX190012763.g |
| SSHX06 | 107754451 | 107756866 | transcript | SSHX060052032.g |
| SSHX14 | 18932868 | 18935379 | transcript | SSHX140009555.g |
| SSHX07 | 103734194 | 103742211 | transcript | SSHX070055215.g |
| SSHX06 | 98528145 | 98532171 | transcript | SSHX060051601.g |
| SSHX14 | 15094056 | 15112632 | transcript | SSHX140009352.g |
| SSHX04 | 40406703 | 40411800 | transcript | SSHX040004329.g |
| SSHX16 | 20983390 | 20988902 | transcript | SSHX160059864.g |
| SSHX02 | 61078069 | 61083047 | transcript | SSHX020045883.g |
| SSHX04 | 37412130 | 37414386 | transcript | SSHX040004138.g |
| SSHX04 | 123229878 | 123243889 | transcript | SSHX040007634.g |
| SSHX03 | 16057973 | 16064410 | transcript | SSHX030029573.g |
| SSHX04 | 58967282 | 58968335 | transcript | SSHX040005535.g |
| SSHX04 | 58967282 | 58967542 | exon | SSHX040005535.g |
| SSHX04 | 58967282 | 58967542 | CDS | SSHX040005535.g |
| SSHX13 | 6486739 | 6492840 | transcript | SSHX130018920.g |
| SSHX02 | 158945719 | 158954237 | transcript | SSHX020048436.g |
| SSHX04 | 25280698 | 25282712 | transcript | SSHX040003692.g |
| SSHX14 | 28766640 | 28786803 | transcript | SSHX140010247.g |
| SSHX18 | 49134389 | 49141379 | transcript | SSHX180014703.g |
| SSHX02 | 158322636 | 158330758 | transcript | SSHX020048401.g |
| SSHX11 | 48615605 | 48617547 | transcript | SSHX110026285.g |
| SSHX16 | 37943521 | 37951779 | transcript | SSHX160060151.g |
| SSHX09 | 92279969 | 92285561 | transcript | SSHX090024525.g |
| SSHX09 | 92279969 | 92280407 | exon | SSHX090024525.g |
| SSHX09 | 92279969 | 92280407 | CDS | SSHX090024525.g |
| SSHX07 | 84148732 | 84150832 | transcript | SSHX070054457.g |
| SSHX06 | 33763943 | 33770543 | transcript | SSHX060050659.g |
| SSHX02 | 65482416 | 65484576 | transcript | SSHX020046159.g |
| SSHX07 | 14992519 | 14996635 | transcript | SSHX070053527.g |
| SSHX14 | 7902174 | 7903632 | transcript | SSHX140008937.g |
| SSHX10 | 12119712 | 12126327 | transcript | SSHX100056457.g |
| SSHX14 | 40079752 | 40085340 | transcript | SSHX140010886.g |
| SSHX02 | 59389484 | 59392690 | transcript | SSHX020045788.g |
| SSHX07 | 91101817 | 91103697 | transcript | SSHX070054619.g |
| SSHX09 | 98123358 | 98126146 | transcript | SSHX090024799.g |
| SSHX17 | 30112343 | 30120316 | transcript | SSHX170043251.g |
| SSHX01 | 193880147 | 193887624 | transcript | SSHX010068063.g |
| SSHX16 | 73997644 | 74002056 | transcript | SSHX160061636.g |
| SSHX07 | 105739897 | 105749394 | transcript | SSHX070055333.g |
| SSHX12 | 60656620 | 60666559 | transcript | SSHX120001891.g |
| SSHX14 | 21866370 | 21867432 | transcript | SSHX140009767.g |
| SSHX01 | 122374497 | 122377711 | transcript | SSHX010065291.g |
| SSHX02 | 164780730 | 164783588 | transcript | SSHX020048788.g |
| SSHX13 | 14081634 | 14092498 | transcript | SSHX130019416.g |
| SSHX02 | 150643691 | 150650819 | transcript | SSHX020048086.g |
| SSHX02 | 61641084 | 61651603 | transcript | SSHX020045923.g |
| SSHX08 | 104694665 | 104697554 | transcript | SSHX080040894.g |
| SSHX17 | 45790699 | 45793246 | transcript | SSHX170043821.g |
| SSHX04 | 117805200 | 117816738 | transcript | SSHX040007298.g |
| SSHX04 | 69243987 | 69245368 | transcript | SSHX040006046.g |
| SSHX08 | 37750668 | 37757971 | transcript | SSHX080038690.g |
| SSHX05 | 73086248 | 73089944 | transcript | SSHX050035621.g |
| SSHX09 | 25060492 | 25071414 | transcript | SSHX090022924.g |
| SSHX05 | 90082507 | 90086321 | transcript | SSHX050036670.g |
| SSHX08 | 35436442 | 35443840 | transcript | SSHX080038530.g |
| SSHX14 | 31528595 | 31539429 | transcript | SSHX140010415.g |
| SSHX05 | 78036982 | 78040437 | transcript | SSHX050035912.g |
| SSHX12 | 52963390 | 52970002 | transcript | SSHX120001511.g |
| SSHX07 | 1218686 | 1233087 | transcript | SSHX070052927.g |
| SSHX12 | 24742069 | 24751827 | transcript | SSHX120000394.g |
| SSHX08 | 109746331 | 109752754 | transcript | SSHX080041172.g |
| SSHX04 | 49205988 | 49208613 | transcript | SSHX040004812.g |
| SSHX10 | 1373859 | 1381665 | transcript | SSHX100055941.g |
| SSHX10 | 99537935 | 99542171 | transcript | SSHX100058972.g |
| SSHX02 | 70291553 | 70296854 | transcript | SSHX020046463.g |
| SSHX08 | 39925298 | 39935227 | transcript | SSHX080038863.g |
| SSHX02 | 41058826 | 41063421 | transcript | SSHX020044839.g |
| SSHX07 | 7367967 | 7369934 | transcript | SSHX070053268.g |
| SSHX11 | 78404542 | 78409608 | transcript | SSHX110027974.g |
| SSHX01 | 198785136 | 198785579 | transcript | SSHX010068375.g |
| SSHX01 | 198785136 | 198785579 | exon | SSHX010068375.g |
| SSHX01 | 198785136 | 198785579 | CDS | SSHX010068375.g |
| SSHX01 | 186797631 | 186802509 | transcript | SSHX010067583.g |
| SSHX19 | 57096349 | 57099530 | transcript | SSHX190013619.g |
| SSHX11 | 80199518 | 80205121 | transcript | SSHX110028043.g |
| SSHX06 | 109688722 | 109689946 | transcript | SSHX060052106.g |
| SSHX13 | 12539647 | 12544096 | transcript | SSHX130019318.g |
| SSHX07 | 120312 | 121515 | transcript | SSHX070052842.g |
| SSHX07 | 18251047 | 18251544 | transcript | SSHX070053617.g |
| SSHX01 | 193876466 | 193880138 | transcript | SSHX010068062.g |
| SSHX14 | 31950117 | 31952204 | transcript | SSHX140010442.g |
| SSHX17 | 10437600 | 10437811 | exon | SSHX170042133.g |
| SSHX17 | 10437600 | 10437811 | CDS | SSHX170042133.g |
| SSHX17 | 10433734 | 10438218 | transcript | SSHX170042133.g |
| SSHX04 | 50665601 | 50668352 | transcript | SSHX040004919.g |
| SSHX02 | 161627393 | 161635861 | transcript | SSHX020048603.g |
| SSHX08 | 182352 | 188336 | transcript | SSHX080037600.g |
| SSHX06 | 7965807 | 7970962 | transcript | SSHX060049511.g |
| SSHX19 | 53768508 | 53773846 | transcript | SSHX190013383.g |
| SSHX04 | 120307164 | 120309027 | transcript | SSHX040007443.g |
| SSHX13 | 6127029 | 6135413 | transcript | SSHX130018892.g |
| SSHX08 | 110145513 | 110153671 | transcript | SSHX080041195.g |
| SSHX06 | 109306965 | 109307697 | transcript | SSHX060052089.g |
| SSHX06 | 109306965 | 109307363 | exon | SSHX060052089.g |
| SSHX06 | 109306965 | 109307363 | CDS | SSHX060052089.g |
| SSHX17 | 23219329 | 23223637 | transcript | SSHX170042860.g |
| SSHX17 | 23221715 | 23222037 | exon | SSHX170042860.g |
| SSHX17 | 23221715 | 23222037 | CDS | SSHX170042860.g |
| SSHX10 | 10467903 | 10473136 | transcript | SSHX100056376.g |
| SSHX02 | 165730013 | 165739957 | transcript | SSHX020048837.g |
| SSHX04 | 58444281 | 58448252 | transcript | SSHX040005497.g |
| SSHX13 | 78885147 | 78893508 | transcript | SSHX130021307.g |
| SSHX16 | 63851972 | 63852298 | transcript | SSHX160061077.g |
| SSHX16 | 63851972 | 63852298 | exon | SSHX160061077.g |
| SSHX16 | 63851972 | 63852298 | CDS | SSHX160061077.g |
| SSHX16 | 62781707 | 62788914 | transcript | SSHX160061000.g |
| SSHX14 | 44461075 | 44475716 | transcript | SSHX140011098.g |
| SSHX10 | 92887726 | 92899719 | transcript | SSHX100058592.g |
| SSHX04 | 133043123 | 133043779 | transcript | SSHX040008259.g |
| SSHX14 | 47454225 | 47456844 | transcript | SSHX140011221.g |
| SSHX09 | 27822989 | 27834165 | transcript | SSHX090023055.g |
| SSHX06 | 8231417 | 8236342 | transcript | SSHX060049526.g |
| SSHX19 | 51790328 | 51805615 | transcript | SSHX190013278.g |
| SSHX05 | 46175817 | 46190822 | transcript | SSHX050034015.g |
| SSHX02 | 74066929 | 74079518 | transcript | SSHX020046584.g |
| SSHX09 | 26005039 | 26018173 | transcript | SSHX090022988.g |
| SSHX01 | 125099076 | 125102635 | transcript | SSHX010065494.g |
| SSHX10 | 92775472 | 92776024 | transcript | SSHX100058585.g |
| SSHX04 | 55040736 | 55045221 | transcript | SSHX040005251.g |
| SSHX19 | 57224548 | 57232566 | transcript | SSHX190013623.g |
| SSHX06 | 120233380 | 120239662 | transcript | SSHX060052601.g |
| SSHX04 | 103839677 | 103842613 | transcript | SSHX040006696.g |
| SSHX15 | 5750771 | 5761365 | transcript | SSHX150016304.g |
| SSHX10 | 64892907 | 64894165 | transcript | SSHX100057328.g |
| SSHX10 | 64894022 | 64894165 | exon | SSHX100057328.g |
| SSHX10 | 64894022 | 64894165 | CDS | SSHX100057328.g |
| SSHX01 | 68441102 | 68442006 | transcript | SSHX010063511.g |
| SSHX15 | 27291 | 31167 | transcript | SSHX150015981.g |
| SSHX02 | 160711948 | 160720532 | transcript | SSHX020048537.g |
| SSHX18 | 2631806 | 2639154 | transcript | SSHX180014034.g |
| SSHX05 | 64924030 | 64927087 | transcript | SSHX050035112.g |
| SSHX02 | 73559372 | 73560532 | transcript | SSHX020046566.g |
| SSHX01 | 126014005 | 126017977 | transcript | SSHX010065537.g |
| SSHX02 | 154651811 | 154652593 | transcript | SSHX020048248.g |
| SSHX09 | 13420313 | 13422625 | transcript | SSHX090022158.g |
| SSHX09 | 13422185 | 13422625 | exon | SSHX090022158.g |
| SSHX09 | 13422185 | 13422625 | CDS | SSHX090022158.g |
| SSHX14 | 24337665 | 24345358 | transcript | SSHX140009928.g |
| SSHX17 | 22674045 | 22687344 | transcript | SSHX170042828.g |
| SSHX09 | 89779439 | 89784886 | transcript | SSHX090024438.g |
| SSHX04 | 38418754 | 38427291 | transcript | SSHX040004200.g |
| SSHX15 | 66042767 | 66048381 | transcript | SSHX150017718.g |
| SSHX18 | 58007921 | 58027399 | transcript | SSHX180015024.g |
| SSHX07 | 15970185 | 15972787 | transcript | SSHX070053564.g |
| SSHX06 | 93995676 | 93996563 | transcript | SSHX060051513.g |
| SSHX06 | 93995676 | 93995819 | exon | SSHX060051513.g |
| SSHX06 | 93995676 | 93995819 | CDS | SSHX060051513.g |
| SSHX18 | 49280941 | 49288414 | transcript | SSHX180014708.g |
| SSHX13 | 15034739 | 15042654 | transcript | SSHX130019470.g |
| SSHX11 | 53343081 | 53343926 | transcript | SSHX110026519.g |
| SSHX16 | 43545625 | 43546825 | transcript | SSHX160060243.g |
| SSHX12 | 61710501 | 61714091 | transcript | SSHX120001938.g |
| SSHX08 | 44258842 | 44263123 | transcript | SSHX080039174.g |
| SSHX05 | 97583033 | 97589282 | transcript | SSHX050037095.g |
| SSHX13 | 19153515 | 19161331 | transcript | SSHX130019682.g |
| SSHX12 | 54410122 | 54412758 | transcript | SSHX120001561.g |
| SSHX17 | 14230381 | 14235724 | transcript | SSHX170042349.g |
| SSHX08 | 111594968 | 111601840 | transcript | SSHX080041285.g |
| SSHX06 | 111087250 | 111087990 | transcript | SSHX060052176.g |
| SSHX06 | 111087250 | 111087696 | exon | SSHX060052176.g |
| SSHX06 | 111087250 | 111087696 | CDS | SSHX060052176.g |
| SSHX06 | 2982294 | 2983740 | transcript | SSHX060049277.g |
| SSHX18 | 63772143 | 63776801 | transcript | SSHX180015408.g |
| SSHX10 | 98728660 | 98742194 | transcript | SSHX100058921.g |
| SSHX03 | 28349851 | 28352802 | transcript | SSHX030030272.g |
| SSHX16 | 69080576 | 69094585 | transcript | SSHX160061342.g |
| SSHX16 | 67298733 | 67301928 | transcript | SSHX160061284.g |
| SSHX16 | 67300859 | 67301191 | exon | SSHX160061284.g |
| SSHX16 | 67300859 | 67301191 | CDS | SSHX160061284.g |
| SSHX10 | 90792045 | 90802653 | transcript | SSHX100058480.g |
| SSHX14 | 74956543 | 74959990 | transcript | SSHX140011733.g |
| SSHX01 | 194402901 | 194404422 | transcript | SSHX010068092.g |
| SSHX01 | 126367898 | 126372001 | transcript | SSHX010065563.g |
| SSHX16 | 74022034 | 74028440 | transcript | SSHX160061637.g |
| SSHX01 | 51209877 | 51210176 | transcript | SSHX010062687.g |
| SSHX01 | 51209877 | 51210176 | exon | SSHX010062687.g |
| SSHX01 | 51209877 | 51210176 | CDS | SSHX010062687.g |
| SSHX04 | 22180156 | 22189170 | transcript | SSHX040003638.g |
| SSHX06 | 3546760 | 3554214 | transcript | SSHX060049310.g |
| SSHX14 | 7051260 | 7052398 | transcript | SSHX140008877.g |
| SSHX12 | 56276827 | 56285072 | transcript | SSHX120001679.g |
| SSHX01 | 180945248 | 180947794 | transcript | SSHX010067257.g |
| SSHX13 | 19356774 | 19364768 | transcript | SSHX130019696.g |
| SSHX01 | 170164790 | 170165105 | exon | SSHX010066815.g |
| SSHX01 | 170164790 | 170165105 | CDS | SSHX010066815.g |
| SSHX01 | 170162062 | 170165105 | transcript | SSHX010066815.g |
| SSHX14 | 35513821 | 35518752 | transcript | SSHX140010684.g |
| SSHX15 | 68723198 | 68724948 | transcript | SSHX150017804.g |
| SSHX11 | 83226914 | 83229473 | transcript | SSHX110028217.g |
| SSHX14 | 15236118 | 15242996 | transcript | SSHX140009358.g |
| SSHX10 | 83834617 | 83842144 | transcript | SSHX100058112.g |
| SSHX17 | 3186028 | 3189475 | transcript | SSHX170041674.g |
| SSHX15 | 17378276 | 17385415 | transcript | SSHX150016780.g |
| SSHX01 | 180397173 | 180404633 | transcript | SSHX010067236.g |
| SSHX07 | 2251629 | 2255173 | transcript | SSHX070052986.g |
| SSHX07 | 2252211 | 2252344 | exon | SSHX070052986.g |
| SSHX07 | 2252211 | 2252344 | CDS | SSHX070052986.g |
| SSHX06 | 118490862 | 118500797 | transcript | SSHX060052472.g |
| SSHX15 | 1057534 | 1060837 | transcript | SSHX150016036.g |
| SSHX01 | 66470075 | 66472443 | transcript | SSHX010063451.g |
| SSHX15 | 3524970 | 3529072 | transcript | SSHX150016173.g |
| SSHX14 | 6650413 | 6652585 | transcript | SSHX140008850.g |
| SSHX09 | 99492669 | 99499521 | transcript | SSHX090024908.g |
| SSHX12 | 74146249 | 74147825 | transcript | SSHX120002688.g |
| SSHX15 | 3899948 | 3902184 | transcript | SSHX150016196.g |
| SSHX05 | 94753365 | 94756296 | transcript | SSHX050036931.g |
| SSHX04 | 34628127 | 34633544 | transcript | SSHX040003984.g |
| SSHX07 | 104385839 | 104393413 | transcript | SSHX070055256.g |
| SSHX14 | 69390506 | 69392451 | transcript | SSHX140011643.g |
| SSHX03 | 8904867 | 8909357 | transcript | SSHX030029050.g |
| SSHX01 | 133639362 | 133643981 | transcript | SSHX010065999.g |
| SSHX11 | 61742561 | 61771048 | transcript | SSHX110027022.g |
| SSHX07 | 16427320 | 16438374 | transcript | SSHX070053573.g |
| SSHX01 | 111327973 | 111333038 | transcript | SSHX010064568.g |
| SSHX12 | 61160455 | 61165469 | transcript | SSHX120001916.g |
| SSHX17 | 33222254 | 33232870 | transcript | SSHX170043371.g |
| SSHX07 | 113865646 | 113875081 | transcript | SSHX070055693.g |
| SSHX01 | 188609421 | 188616332 | transcript | SSHX010067710.g |
| SSHX08 | 106960244 | 106969387 | transcript | SSHX080041010.g |
| SSHX08 | 3527815 | 3529334 | transcript | SSHX080037736.g |
| SSHX05 | 91184177 | 91191883 | transcript | SSHX050036735.g |
| SSHX13 | 12555574 | 12567014 | transcript | SSHX130019320.g |
| SSHX05 | 64748184 | 64748575 | transcript | SSHX050035094.g |
| SSHX16 | 1090750 | 1093331 | transcript | SSHX160059352.g |
| SSHX04 | 55507663 | 55519383 | transcript | SSHX040005300.g |
| SSHX13 | 26616438 | 26619022 | transcript | SSHX130020078.g |
| SSHX19 | 51222964 | 51223391 | transcript | SSHX190013239.g |
| SSHX06 | 1482660 | 1486129 | transcript | SSHX060049167.g |
| SSHX12 | 76080577 | 76088789 | transcript | SSHX120002818.g |
| SSHX05 | 4085963 | 4093689 | transcript | SSHX050033141.g |
| SSHX05 | 87197526 | 87205436 | transcript | SSHX050036510.g |
| SSHX05 | 27995468 | 28001067 | transcript | SSHX050033499.g |
| SSHX02 | 43035400 | 43037547 | transcript | SSHX020044908.g |
| SSHX16 | 23787102 | 23799660 | transcript | SSHX160059904.g |
| SSHX02 | 170207970 | 170215842 | transcript | SSHX020049054.g |
| SSHX17 | 26710867 | 26712501 | transcript | SSHX170043046.g |
| SSHX18 | 48165543 | 48166514 | transcript | SSHX180014659.g |
| SSHX13 | 2753391 | 2762353 | transcript | C3H |
| SSHX08 | 88840255 | 88846969 | transcript | HB-other |
| SSHX13 | 7584649 | 7588765 | transcript | NAC |
| SSHX10 | 87169308 | 87174660 | transcript | WRKY |
| SSHX06 | 12111986 | 12114639 | transcript | C3H |
| SSHX08 | 53514011 | 53518057 | transcript | bHLH |
| SSHX05 | 70379376 | 70387717 | transcript | MIKC_MADS |
| SSHX19 | 42833518 | 42833639 | transcript | WRKY |
| SSHX13 | 6488284 | 6491487 | transcript | NF-YA |
| SSHX10 | 92481002 | 92485896 | transcript | HD-ZIP |
| SSHX06 | 7969080 | 7969843 | transcript | bHLH |
| SSHX12 | 74146639 | 74146731 | transcript | LSD |

**E.**

| ***Michelia montaua*_CHG** | | | | |
| --- | --- | --- | --- | --- |
| SSHX06 | 2541330 | 2553526 | transcript | SSHX060049246.g |
| SSHX02 | 168379531 | 168384546 | transcript | SSHX020048974.g |
| SSHX18 | 64468082 | 64491883 | transcript | SSHX180015449.g |
| SSHX03 | 11357485 | 11369960 | transcript | SSHX030029261.g |
| SSHX02 | 59043216 | 59055794 | transcript | SSHX020045756.g |
| SSHX10 | 6128645 | 6137929 | transcript | SSHX100056225.g |
| SSHX16 | 73299210 | 73307412 | transcript | SSHX160061600.g |
| SSHX14 | 22000271 | 22008835 | transcript | SSHX140009776.g |
| SSHX10 | 98468666 | 98477273 | transcript | SSHX100058900.g |
| SSHX05 | 46681025 | 46688662 | transcript | SSHX050034028.g |
| SSHX16 | 16412372 | 16430919 | transcript | SSHX160059773.g |
| SSHX03 | 17870220 | 17890545 | transcript | SSHX030029674.g |
| SSHX14 | 17754112 | 17761998 | transcript | SSHX140009505.g |
| SSHX10 | 77138336 | 77147732 | transcript | SSHX100057681.g |
| SSHX14 | 42362342 | 42364366 | transcript | SSHX140010989.g |
| SSHX01 | 183359516 | 183373455 | transcript | SSHX010067381.g |
| SSHX02 | 133176952 | 133184586 | transcript | SSHX020047679.g |
| SSHX16 | 13018301 | 13024044 | transcript | SSHX160059665.g |
| SSHX07 | 4126509 | 4132259 | transcript | SSHX070053125.g |
| SSHX05 | 52992197 | 53005771 | transcript | SSHX050034329.g |
| SSHX17 | 19049809 | 19053995 | transcript | SSHX170042618.g |
| SSHX04 | 101926357 | 101941347 | transcript | SSHX040006622.g |
| SSHX16 | 74277730 | 74287283 | transcript | SSHX160061657.g |
| SSHX15 | 75913001 | 75920999 | transcript | SSHX150018143.g |
| SSHX07 | 780982 | 784313 | transcript | SSHX070052891.g |
| SSHX01 | 68682281 | 68690043 | transcript | SSHX010063518.g |
| SSHX16 | 65881463 | 65889005 | transcript | SSHX160061194.g |
| SSHX07 | 95348260 | 95350527 | transcript | SSHX070054809.g |
| SSHX05 | 11573790 | 11583141 | transcript | SSHX050033214.g |
| SSHX09 | 4744630 | 4757022 | transcript | SSHX090021635.g |
| SSHX05 | 68819018 | 68831936 | transcript | SSHX050035392.g |
| SSHX03 | 17425939 | 17428703 | transcript | SSHX030029661.g |
| SSHX05 | 83448179 | 83458873 | transcript | SSHX050036270.g |
| SSHX13 | 3823954 | 3836121 | transcript | SSHX130018743.g |
| SSHX03 | 137341500 | 137351304 | transcript | SSHX030032430.g |
| SSHX13 | 45089967 | 45094090 | transcript | SSHX130020841.g |
| SSHX03 | 10280016 | 10290986 | transcript | SSHX030029165.g |
| SSHX03 | 2725645 | 2739759 | transcript | SSHX030028668.g |
| SSHX02 | 70132343 | 70140920 | transcript | SSHX020046452.g |
| SSHX02 | 33661907 | 33670345 | transcript | SSHX020044644.g |
| SSHX08 | 102258789 | 102266642 | transcript | SSHX080040793.g |
| SSHX13 | 79775933 | 79780224 | transcript | SSHX130021318.g |
| SSHX08 | 2769459 | 2782957 | transcript | SSHX080037708.g |
| SSHX11 | 83500566 | 83506443 | transcript | SSHX110028233.g |
| SSHX01 | 63963207 | 63966363 | transcript | SSHX010063265.g |
| SSHX18 | 37880892 | 37887111 | transcript | SSHX180014385.g |
| SSHX07 | 106679514 | 106682176 | transcript | SSHX070055378.g |
| SSHX19 | 48003392 | 48013025 | transcript | SSHX190013052.g |
| SSHX19 | 51811760 | 51822589 | transcript | SSHX190013279.g |
| SSHX01 | 108358313 | 108364586 | transcript | SSHX010064409.g |
| SSHX19 | 53927714 | 53935540 | transcript | SSHX190013389.g |
| SSHX08 | 77091431 | 77099634 | transcript | SSHX080040134.g |
| SSHX08 | 46737074 | 46745197 | transcript | SSHX080039331.g |
| SSHX01 | 123608768 | 123616577 | transcript | SSHX010065374.g |
| SSHX08 | 4995175 | 5007257 | transcript | SSHX080037801.g |
| SSHX18 | 48165543 | 48166514 | transcript | SSHX180014659.g |
| SSHX16 | 60072953 | 60080627 | transcript | SSHX160060899.g |
| SSHX08 | 45016465 | 45019874 | transcript | SSHX080039225.g |
| SSHX11 | 38529919 | 38539996 | transcript | SSHX110025876.g |
| SSHX01 | 185696160 | 185717075 | transcript | SSHX010067516.g |
| SSHX14 | 28766640 | 28786803 | transcript | SSHX140010247.g |
| SSHX03 | 144546665 | 144558660 | transcript | SSHX030032797.g |
| SSHX03 | 62716214 | 62718713 | transcript | SSHX030031142.g |
| SSHX01 | 20094578 | 20098552 | transcript | SSHX010062108.g |
| SSHX10 | 23943983 | 23952805 | transcript | SSHX100056763.g |
| SSHX01 | 64341817 | 64352090 | transcript | SSHX010063308.g |
| SSHX14 | 6717446 | 6726105 | transcript | SSHX140008856.g |
| SSHX09 | 102928660 | 102935456 | transcript | SSHX090025088.g |
| SSHX03 | 117539517 | 117560053 | transcript | SSHX030031698.g |
| SSHX06 | 2309260 | 2320337 | transcript | SSHX060049230.g |
| SSHX10 | 87542816 | 87555327 | transcript | SSHX100058330.g |
| SSHX09 | 31621726 | 31629685 | transcript | SSHX090023199.g |
| SSHX04 | 51630468 | 51638074 | transcript | SSHX040005013.g |
| SSHX16 | 68816675 | 68818661 | transcript | SSHX160061322.g |
| SSHX06 | 112361596 | 112367672 | transcript | SSHX060052241.g |
| SSHX01 | 122153237 | 122159761 | transcript | SSHX010065284.g |
| SSHX18 | 69390692 | 69398119 | transcript | SSHX180015699.g |
| SSHX01 | 131571435 | 131576571 | transcript | SSHX010065886.g |
| SSHX11 | 62630744 | 62645634 | transcript | SSHX110027088.g |
| SSHX08 | 46278104 | 46279541 | transcript | SSHX080039321.g |
| SSHX17 | 12578005 | 12591017 | transcript | SSHX170042264.g |
| SSHX10 | 92475599 | 92483925 | transcript | SSHX100058567.g |
| SSHX07 | 86246712 | 86254112 | transcript | SSHX070054504.g |
| SSHX10 | 1373859 | 1381665 | transcript | SSHX100055941.g |
| SSHX03 | 144877743 | 144886435 | transcript | SSHX030032822.g |
| SSHX10 | 73979301 | 73986202 | transcript | SSHX100057556.g |
| SSHX16 | 73487108 | 73496299 | transcript | SSHX160061610.g |
| SSHX02 | 62231368 | 62238288 | transcript | SSHX020045946.g |
| SSHX17 | 18535737 | 18538712 | transcript | SSHX170042584.g |
| SSHX16 | 68636042 | 68639639 | transcript | SSHX160061307.g |
| SSHX17 | 13647362 | 13648262 | transcript | SSHX170042309.g |
| SSHX15 | 74102156 | 74106322 | transcript | SSHX150018036.g |
| SSHX01 | 132666174 | 132671748 | transcript | SSHX010065944.g |
| SSHX17 | 37987174 | 37997714 | transcript | SSHX170043526.g |
| SSHX04 | 122825542 | 122834516 | transcript | SSHX040007610.g |
| SSHX12 | 62594050 | 62607607 | transcript | SSHX120001986.g |
| SSHX14 | 26183608 | 26190404 | transcript | SSHX140010053.g |
| SSHX10 | 25975225 | 25982079 | transcript | SSHX100056816.g |
| SSHX14 | 6772573 | 6780658 | transcript | SSHX140008860.g |
| SSHX02 | 61641084 | 61651603 | transcript | SSHX020045923.g |
| SSHX19 | 31326931 | 31334773 | transcript | SSHX190012276.g |
| SSHX18 | 51298980 | 51317358 | transcript | SSHX180014784.g |
| SSHX08 | 101420627 | 101438790 | transcript | SSHX080040769.g |
| SSHX18 | 69778853 | 69786416 | transcript | SSHX180015726.g |
| SSHX09 | 105958890 | 105969547 | transcript | SSHX090025290.g |
| SSHX14 | 48779752 | 48785514 | transcript | SSHX140011262.g |
| SSHX02 | 166976639 | 166983824 | transcript | SSHX020048917.g |
| SSHX08 | 109532475 | 109537727 | transcript | SSHX080041154.g |
| SSHX17 | 5958470 | 5966447 | transcript | SSHX170041864.g |
| SSHX16 | 76941243 | 76948379 | transcript | SSHX160061843.g |
| SSHX08 | 112349353 | 112357019 | transcript | SSHX080041335.g |
| SSHX12 | 53135767 | 53142640 | transcript | SSHX120001518.g |
| SSHX05 | 65397700 | 65409186 | transcript | SSHX050035141.g |
| SSHX02 | 30470496 | 30473373 | transcript | SSHX020044581.g |
| SSHX04 | 130094583 | 130105979 | transcript | SSHX040008075.g |
| SSHX05 | 104285996 | 104286295 | transcript | SSHX050037263.g |
| SSHX05 | 104285996 | 104286295 | exon | SSHX050037263.g |
| SSHX05 | 104285996 | 104286295 | CDS | SSHX050037263.g |
| SSHX17 | 33810595 | 33819352 | transcript | SSHX170043386.g |
| SSHX15 | 4505715 | 4509836 | transcript | SSHX150016231.g |
| SSHX08 | 63278807 | 63286240 | transcript | SSHX080039962.g |
| SSHX06 | 120639966 | 120649905 | transcript | SSHX060052638.g |
| SSHX12 | 75671556 | 75681286 | transcript | SSHX120002781.g |
| SSHX05 | 43455689 | 43464269 | transcript | SSHX050033909.g |
| SSHX12 | 28151088 | 28156038 | transcript | SSHX120000497.g |
| SSHX03 | 41338838 | 41348203 | transcript | SSHX030030701.g |
| SSHX16 | 65846233 | 65853761 | transcript | SSHX160061186.g |
| SSHX13 | 2271214 | 2282334 | transcript | SSHX130018632.g |
| SSHX04 | 110337073 | 110342034 | transcript | SSHX040006867.g |
| SSHX17 | 15598985 | 15616243 | transcript | SSHX170042425.g |
| SSHX11 | 30778190 | 30778616 | transcript | SSHX110025700.g |
| SSHX03 | 147441769 | 147447471 | transcript | SSHX030032994.g |
| SSHX02 | 89757559 | 89764722 | transcript | SSHX020047016.g |
| SSHX03 | 25413065 | 25420309 | transcript | SSHX030030118.g |
| SSHX03 | 7753912 | 7755470 | transcript | SSHX030028987.g |
| SSHX03 | 7754020 | 7754521 | exon | SSHX030028987.g |
| SSHX03 | 7754020 | 7754521 | CDS | SSHX030028987.g |
| SSHX15 | 16102774 | 16112896 | transcript | SSHX150016757.g |
| SSHX08 | 68760871 | 68763412 | transcript | SSHX080040049.g |
| SSHX04 | 133043123 | 133043779 | transcript | SSHX040008259.g |
| SSHX09 | 51243057 | 51249421 | transcript | SSHX090023641.g |
| SSHX04 | 61586989 | 61592637 | transcript | SSHX040005679.g |
| SSHX04 | 130136258 | 130137181 | transcript | SSHX040008078.g |
| SSHX01 | 181547516 | 181557423 | transcript | SSHX010067275.g |
| SSHX08 | 109054484 | 109069990 | transcript | SSHX080041120.g |
| SSHX03 | 130970056 | 130980133 | transcript | SSHX030032118.g |
| SSHX01 | 81314961 | 81321468 | transcript | SSHX010063799.g |
| SSHX10 | 91385052 | 91387438 | transcript | SSHX100058526.g |
| SSHX01 | 190622933 | 190625271 | transcript | SSHX010067849.g |
| SSHX04 | 69194753 | 69209581 | transcript | SSHX040006043.g |
| SSHX14 | 8973817 | 8982246 | transcript | SSHX140008994.g |
| SSHX13 | 19610127 | 19613523 | transcript | SSHX130019716.g |
| SSHX14 | 2978459 | 2988537 | transcript | SSHX140008625.g |
| SSHX19 | 49688373 | 49696854 | transcript | SSHX190013144.g |
| SSHX04 | 117805200 | 117816738 | transcript | SSHX040007298.g |
| SSHX16 | 63796713 | 63807651 | transcript | SSHX160061072.g |
| SSHX12 | 53766160 | 53774915 | transcript | SSHX120001533.g |
| SSHX10 | 98728660 | 98742194 | transcript | SSHX100058921.g |
| SSHX04 | 124793731 | 124796952 | transcript | SSHX040007725.g |
| SSHX15 | 4206580 | 4213188 | transcript | SSHX150016216.g |
| SSHX12 | 76397787 | 76408329 | transcript | SSHX120002835.g |
| SSHX13 | 8282915 | 8287765 | transcript | SSHX130019043.g |
| SSHX09 | 8682149 | 8688549 | transcript | SSHX090021847.g |
| SSHX12 | 62814617 | 62815505 | transcript | SSHX120002008.g |
| SSHX09 | 27822989 | 27834165 | transcript | SSHX090023055.g |
| SSHX05 | 77170021 | 77176939 | transcript | SSHX050035870.g |
| SSHX01 | 83328669 | 83332739 | transcript | SSHX010063836.g |
| SSHX01 | 114945820 | 114953769 | transcript | SSHX010064782.g |
| SSHX13 | 3525922 | 3537554 | transcript | SSHX130018719.g |
| SSHX04 | 35128754 | 35138954 | transcript | SSHX040004000.g |
| SSHX11 | 66552526 | 66563994 | transcript | SSHX110027288.g |
| SSHX07 | 15798049 | 15806075 | transcript | SSHX070053559.g |
| SSHX07 | 109481059 | 109482015 | transcript | SSHX070055486.g |
| SSHX07 | 109481059 | 109481666 | exon | SSHX070055486.g |
| SSHX07 | 109481059 | 109481666 | CDS | SSHX070055486.g |
| SSHX13 | 13936259 | 13943832 | transcript | SSHX130019406.g |
| SSHX05 | 61853449 | 61855145 | transcript | SSHX050034894.g |
| SSHX17 | 62910394 | 62911736 | transcript | SSHX170044165.g |
| SSHX01 | 63969645 | 63976663 | transcript | SSHX010063266.g |
| SSHX03 | 45822217 | 45823287 | transcript | SSHX030030757.g |
| SSHX12 | 80061352 | 80063647 | transcript | SSHX120003064.g |
| SSHX11 | 52895214 | 52903735 | transcript | SSHX110026502.g |
| SSHX01 | 56486269 | 56491907 | transcript | SSHX010062916.g |
| SSHX13 | 23401976 | 23411786 | transcript | SSHX130019902.g |
| SSHX05 | 86670069 | 86678492 | transcript | SSHX050036472.g |
| SSHX13 | 1302231 | 1308410 | transcript | SSHX130018562.g |
| SSHX01 | 184131026 | 184141560 | transcript | SSHX010067424.g |
| SSHX09 | 13035471 | 13040644 | transcript | SSHX090022135.g |
| SSHX16 | 77020936 | 77029125 | transcript | SSHX160061849.g |
| SSHX03 | 40385452 | 40396409 | transcript | SSHX030030672.g |
| SSHX19 | 44198097 | 44207986 | transcript | SSHX190012829.g |
| SSHX14 | 45802433 | 45813354 | transcript | SSHX140011147.g |
| SSHX04 | 34717815 | 34723692 | transcript | SSHX040003988.g |
| SSHX10 | 84724020 | 84732812 | transcript | SSHX100058158.g |
| SSHX06 | 110068706 | 110072857 | transcript | SSHX060052123.g |
| SSHX11 | 81491259 | 81494096 | transcript | SSHX110028127.g |
| SSHX02 | 75129990 | 75134528 | transcript | SSHX020046615.g |
| SSHX02 | 67240610 | 67246531 | transcript | SSHX020046319.g |
| SSHX19 | 34130790 | 34141787 | transcript | SSHX190012374.g |
| SSHX09 | 28661609 | 28664763 | transcript | SSHX090023096.g |
| SSHX10 | 19882142 | 19885777 | transcript | SSHX100056680.g |
| SSHX06 | 3116852 | 3122542 | transcript | SSHX060049286.g |
| SSHX11 | 80832216 | 80849889 | transcript | SSHX110028081.g |
| SSHX13 | 20782506 | 20787397 | transcript | SSHX130019778.g |
| SSHX17 | 18548550 | 18559648 | transcript | SSHX170042586.g |
| SSHX09 | 65536116 | 65540502 | transcript | SSHX090023792.g |
| SSHX09 | 65536116 | 65536352 | exon | SSHX090023792.g |
| SSHX09 | 65536116 | 65536352 | CDS | SSHX090023792.g |
| SSHX18 | 51298980 | 51317358 | transcript | SSHX180014784.g |
| SSHX05 | 29725159 | 29726497 | transcript | SSHX050033517.g |
| SSHX18 | 62402539 | 62409106 | transcript | SSHX180015336.g |
| SSHX05 | 93203361 | 93217988 | transcript | SSHX050036852.g |
| SSHX14 | 44461075 | 44475716 | transcript | SSHX140011098.g |
| SSHX08 | 34693976 | 34705880 | transcript | SSHX080038474.g |
| SSHX18 | 68765459 | 68769119 | transcript | SSHX180015650.g |
| SSHX13 | 6373386 | 6377659 | transcript | SSHX130018909.g |
| SSHX13 | 6374374 | 6374890 | exon | SSHX130018909.g |
| SSHX13 | 6374374 | 6374890 | CDS | SSHX130018909.g |
| SSHX09 | 2670150 | 2671906 | transcript | SSHX090021534.g |
| SSHX18 | 59142547 | 59147971 | transcript | SSHX180015121.g |
| SSHX01 | 166745657 | 166750522 | transcript | SSHX010066750.g |
| SSHX11 | 83029423 | 83033191 | transcript | SSHX110028213.g |
| SSHX08 | 72022849 | 72023100 | transcript | SSHX080040088.g |
| SSHX15 | 72538862 | 72545058 | transcript | SSHX150017964.g |
| SSHX08 | 47819074 | 47829943 | transcript | SSHX080039383.g |
| SSHX04 | 74417196 | 74429228 | transcript | SSHX040006191.g |
| SSHX02 | 65059990 | 65064541 | transcript | SSHX020046122.g |
| SSHX12 | 80604893 | 80612034 | transcript | SSHX120003113.g |
| SSHX04 | 78538306 | 78541034 | transcript | SSHX040006290.g |
| SSHX06 | 110387276 | 110397829 | transcript | SSHX060052149.g |
| SSHX03 | 127156135 | 127158899 | transcript | SSHX030031989.g |
| SSHX18 | 50563193 | 50568246 | transcript | SSHX180014747.g |
| SSHX17 | 8275145 | 8281853 | transcript | SSHX170041993.g |
| SSHX03 | 43833973 | 43841674 | transcript | SSHX030030733.g |
| SSHX04 | 123229878 | 123243889 | transcript | SSHX040007634.g |
| SSHX14 | 19195066 | 19196897 | transcript | SSHX140009580.g |
| SSHX06 | 118490862 | 118500797 | transcript | SSHX060052472.g |
| SSHX11 | 67712444 | 67721258 | transcript | SSHX110027364.g |
| SSHX02 | 118148110 | 118153406 | transcript | SSHX020047522.g |
| SSHX08 | 98574763 | 98585062 | transcript | SSHX080040651.g |
| SSHX15 | 10206822 | 10210066 | transcript | SSHX150016510.g |
| SSHX05 | 65347211 | 65355346 | transcript | SSHX050035135.g |
| SSHX05 | 84198820 | 84202509 | transcript | SSHX050036346.g |
| SSHX01 | 110938745 | 110952727 | transcript | SSHX010064546.g |
| SSHX13 | 17346136 | 17356074 | transcript | SSHX130019618.g |
| SSHX04 | 50266525 | 50273461 | transcript | SSHX040004897.g |
| SSHX18 | 58007921 | 58027399 | transcript | SSHX180015024.g |
| SSHX15 | 72362382 | 72374427 | transcript | SSHX150017957.g |
| SSHX09 | 14181784 | 14188013 | transcript | SSHX090022214.g |
| SSHX01 | 35512543 | 35515385 | transcript | SSHX010062307.g |
| SSHX01 | 111627059 | 111633453 | transcript | SSHX010064593.g |
| SSHX13 | 13880649 | 13884662 | transcript | SSHX130019395.g |
| SSHX05 | 65347211 | 65355346 | transcript | SSHX050035135.g |
| SSHX10 | 2145063 | 2152111 | transcript | SSHX100055985.g |
| SSHX05 | 59650979 | 59658331 | transcript | SSHX050034711.g |
| SSHX11 | 74117362 | 74120932 | transcript | SSHX110027748.g |
| SSHX07 | 105739897 | 105749394 | transcript | SSHX070055333.g |
| SSHX02 | 66745339 | 66748212 | transcript | SSHX020046283.g |
| SSHX01 | 67743467 | 67746889 | transcript | SSHX010063493.g |
| SSHX05 | 87881153 | 87885115 | transcript | SSHX050036567.g |
| SSHX09 | 762350 | 771509 | transcript | SSHX090021428.g |
| SSHX05 | 72856962 | 72866441 | transcript | SSHX050035602.g |
| SSHX02 | 59198760 | 59209521 | transcript | SSHX020045774.g |
| SSHX03 | 16057973 | 16064410 | transcript | SSHX030029573.g |
| SSHX02 | 42649196 | 42653706 | transcript | SSHX020044897.g |
| SSHX04 | 31767836 | 31768831 | transcript | SSHX040003880.g |
| SSHX04 | 31767836 | 31768831 | exon | SSHX040003880.g |
| SSHX04 | 31767836 | 31768831 | CDS | SSHX040003880.g |
| SSHX01 | 111751145 | 111780176 | transcript | SSHX010064601.g |
| SSHX06 | 1952972 | 1954836 | transcript | SSHX060049217.g |
| SSHX05 | 62783351 | 62791057 | transcript | SSHX050034983.g |
| SSHX07 | 85336174 | 85339394 | transcript | SSHX070054480.g |
| SSHX15 | 77605881 | 77615016 | transcript | SSHX150018220.g |
| SSHX07 | 116625915 | 116629794 | transcript | SSHX070055850.g |
| SSHX18 | 72460487 | 72486096 | transcript | SSHX180015888.g |
| SSHX10 | 72562970 | 72564947 | transcript | SSHX100057508.g |
| SSHX05 | 75837809 | 75843486 | transcript | SSHX050035797.g |
| SSHX05 | 70154530 | 70160864 | transcript | SSHX050035456.g |
| SSHX07 | 1989659 | 1992953 | transcript | SSHX070052964.g |
| SSHX01 | 65643984 | 65652875 | transcript | SSHX010063395.g |
| SSHX02 | 108361262 | 108367175 | transcript | SSHX020047358.g |
| SSHX03 | 3376491 | 3383219 | transcript | SSHX030028705.g |
| SSHX03 | 3377689 | 3377949 | exon | SSHX030028705.g |
| SSHX03 | 3377689 | 3377949 | CDS | SSHX030028705.g |
| SSHX01 | 48862024 | 48872231 | transcript | SSHX010062631.g |
| SSHX02 | 163921674 | 163924933 | transcript | SSHX020048740.g |
| SSHX06 | 13570336 | 13582422 | transcript | SSHX060049813.g |
| SSHX17 | 8391132 | 8399179 | transcript | SSHX170041997.g |
| SSHX15 | 31368449 | 31375593 | transcript | SSHX150017086.g |
| SSHX05 | 90735946 | 90736233 | transcript | SSHX050036718.g |
| SSHX01 | 182917503 | 182927889 | transcript | SSHX010067358.g |
| SSHX01 | 55529028 | 55535056 | transcript | SSHX010062867.g |
| SSHX06 | 13228722 | 13234169 | transcript | SSHX060049795.g |
| SSHX01 | 131350146 | 131354912 | transcript | SSHX010065879.g |
| SSHX17 | 2878842 | 2880181 | transcript | SSHX170041643.g |
| SSHX02 | 59105456 | 59105728 | transcript | SSHX020045762.g |
| SSHX02 | 59105456 | 59105728 | exon | SSHX020045762.g |
| SSHX02 | 59105456 | 59105728 | CDS | SSHX020045762.g |
| SSHX04 | 53078867 | 53087350 | transcript | SSHX040005118.g |
| SSHX05 | 60023758 | 60032688 | transcript | SSHX050034729.g |
| SSHX01 | 64976432 | 64984391 | transcript | SSHX010063358.g |
| SSHX08 | 3019449 | 3024360 | transcript | SSHX080037723.g |
| SSHX09 | 25198946 | 25201679 | transcript | SSHX090022932.g |
| SSHX02 | 144101226 | 144112612 | transcript | SSHX020047835.g |
| SSHX05 | 64988939 | 64999539 | transcript | SSHX050035115.g |
| SSHX04 | 63690282 | 63695463 | transcript | SSHX040005779.g |
| SSHX10 | 7396794 | 7406813 | transcript | SSHX100056278.g |
| SSHX19 | 42681920 | 42687179 | transcript | SSHX190012757.g |
| SSHX05 | 66071566 | 66077836 | transcript | SSHX050035185.g |
| SSHX02 | 36689376 | 36693947 | transcript | SSHX020044716.g |
| SSHX04 | 74572141 | 74575677 | transcript | SSHX040006193.g |
| SSHX04 | 62824299 | 62829600 | transcript | SSHX040005743.g |
| SSHX14 | 17138305 | 17142785 | transcript | SSHX140009469.g |
| SSHX12 | 50324512 | 50334158 | transcript | SSHX120001407.g |
| SSHX01 | 127561996 | 127575953 | transcript | SSHX010065642.g |
| SSHX02 | 162692920 | 162711621 | transcript | SSHX020048684.g |
| SSHX14 | 53607539 | 53609463 | transcript | SSHX140011393.g |
| SSHX09 | 101617304 | 101620870 | transcript | SSHX090025032.g |
| SSHX01 | 47433330 | 47440838 | transcript | SSHX010062602.g |
| SSHX04 | 18981045 | 18985637 | transcript | SSHX040003599.g |
| SSHX11 | 83688782 | 83689084 | exon | SSHX110028246.g |
| SSHX11 | 83688782 | 83689084 | CDS | SSHX110028246.g |
| SSHX11 | 83687560 | 83691232 | transcript | SSHX110028246.g |
| SSHX15 | 73641304 | 73655315 | transcript | SSHX150018016.g |
| SSHX03 | 124474875 | 124477896 | transcript | SSHX030031924.g |
| SSHX04 | 59978143 | 59979994 | transcript | SSHX040005594.g |
| SSHX01 | 122555103 | 122559546 | transcript | SSHX010065310.g |
| SSHX08 | 15944396 | 15948546 | transcript | SSHX080038036.g |
| SSHX08 | 15945357 | 15945444 | exon | SSHX080038036.g |
| SSHX08 | 15945357 | 15945444 | CDS | SSHX080038036.g |
| SSHX01 | 193689004 | 193694679 | transcript | SSHX010068050.g |
| SSHX12 | 72576578 | 72585118 | transcript | SSHX120002610.g |
| SSHX01 | 180800932 | 180810050 | transcript | SSHX010067256.g |
| SSHX05 | 70090476 | 70095397 | transcript | SSHX050035451.g |
| SSHX01 | 119632907 | 119634581 | transcript | SSHX010065063.g |
| SSHX17 | 18757443 | 18762351 | transcript | SSHX170042596.g |
| SSHX02 | 74934141 | 74941968 | transcript | SSHX020046609.g |
| SSHX05 | 96835906 | 96837405 | transcript | SSHX050037059.g |
| SSHX03 | 892761 | 903691 | transcript | SSHX030028533.g |
| SSHX05 | 86031913 | 86045188 | transcript | SSHX050036446.g |
| SSHX01 | 64642863 | 64648970 | transcript | SSHX010063333.g |
| SSHX13 | 11845139 | 11849189 | transcript | SSHX130019268.g |
| SSHX17 | 5376033 | 5380519 | transcript | SSHX170041826.g |
| SSHX02 | 129228274 | 129231347 | transcript | SSHX020047615.g |
| SSHX02 | 129228274 | 129228477 | exon | SSHX020047615.g |
| SSHX02 | 129228274 | 129228477 | CDS | SSHX020047615.g |
| SSHX09 | 102052271 | 102058571 | transcript | SSHX090025049.g |
| SSHX03 | 146547393 | 146554074 | transcript | SSHX030032943.g |
| SSHX17 | 16580753 | 16584594 | transcript | SSHX170042472.g |
| SSHX06 | 114527969 | 114542284 | transcript | SSHX060052327.g |
| SSHX03 | 147722093 | 147724653 | transcript | SSHX030033027.g |
| SSHX06 | 11866318 | 11871356 | transcript | SSHX060049739.g |
| SSHX05 | 68819018 | 68831936 | transcript | SSHX050035392.g |
| SSHX04 | 113144244 | 113145230 | transcript | SSHX040006998.g |
| SSHX01 | 62826841 | 62831613 | transcript | SSHX010063188.g |
| SSHX11 | 81201788 | 81207542 | transcript | SSHX110028107.g |
| SSHX19 | 39306310 | 39309587 | transcript | SSHX190012601.g |
| SSHX19 | 39309408 | 39309587 | exon | SSHX190012601.g |
| SSHX19 | 39309408 | 39309587 | CDS | SSHX190012601.g |
| SSHX03 | 122344594 | 122350420 | transcript | SSHX030031864.g |
| SSHX06 | 11983365 | 11989191 | transcript | SSHX060049745.g |
| SSHX11 | 66623959 | 66630461 | transcript | SSHX110027298.g |
| SSHX15 | 2657427 | 2669446 | transcript | SSHX150016111.g |
| SSHX05 | 49453673 | 49457834 | transcript | SSHX050034152.g |
| SSHX09 | 98524917 | 98530030 | transcript | SSHX090024832.g |
| SSHX09 | 98524917 | 98525084 | exon | SSHX090024832.g |
| SSHX09 | 98524917 | 98525084 | CDS | SSHX090024832.g |
| SSHX19 | 43515864 | 43528356 | transcript | SSHX190012790.g |
| SSHX03 | 44848422 | 44850936 | transcript | SSHX030030749.g |
| SSHX11 | 69135076 | 69136735 | transcript | SSHX110027457.g |
| SSHX11 | 69136620 | 69136735 | exon | SSHX110027457.g |
| SSHX11 | 69136620 | 69136735 | CDS | SSHX110027457.g |
| SSHX11 | 78898703 | 78903134 | transcript | SSHX110027988.g |
| SSHX11 | 73982952 | 73988274 | transcript | SSHX110027738.g |
| SSHX09 | 10017571 | 10022589 | transcript | SSHX090021948.g |
| SSHX02 | 63852860 | 63863821 | transcript | SSHX020046044.g |
| SSHX04 | 67709451 | 67716420 | transcript | SSHX040005953.g |
| SSHX01 | 189431168 | 189433757 | transcript | SSHX010067766.g |
| SSHX10 | 688360 | 690797 | transcript | SSHX100055903.g |
| SSHX15 | 6965296 | 6970279 | transcript | SSHX150016368.g |
| SSHX11 | 75132319 | 75138073 | transcript | SSHX110027804.g |
| SSHX06 | 1919789 | 1924455 | transcript | SSHX060049213.g |
| SSHX13 | 1050543 | 1051421 | transcript | SSHX130018551.g |
| SSHX13 | 1050543 | 1050796 | exon | SSHX130018551.g |
| SSHX13 | 1050543 | 1050796 | CDS | SSHX130018551.g |
| SSHX13 | 20207215 | 20213749 | transcript | SSHX130019747.g |
| SSHX06 | 7359649 | 7361949 | transcript | SSHX060049489.g |
| SSHX11 | 59167388 | 59168078 | transcript | SSHX110026846.g |
| SSHX02 | 59822590 | 59824133 | transcript | SSHX020045816.g |
| SSHX03 | 9445466 | 9457121 | transcript | SSHX030029088.g |
| SSHX11 | 24304751 | 24308459 | transcript | SSHX110025581.g |
| SSHX09 | 20236459 | 20238227 | transcript | SSHX090022633.g |
| SSHX11 | 70022424 | 70039048 | transcript | SSHX110027506.g |
| SSHX03 | 141356523 | 141368285 | transcript | SSHX030032619.g |
| SSHX07 | 94936643 | 94940868 | transcript | SSHX070054794.g |
| SSHX07 | 94936643 | 94937250 | exon | SSHX070054794.g |
| SSHX07 | 94936643 | 94937250 | CDS | SSHX070054794.g |
| SSHX10 | 87542816 | 87555327 | transcript | SSHX100058330.g |
| SSHX12 | 74623335 | 74632318 | transcript | SSHX120002715.g |
| SSHX19 | 40413532 | 40419329 | transcript | SSHX190012646.g |
| SSHX08 | 108808157 | 108823384 | transcript | SSHX080041109.g |
| SSHX12 | 69555144 | 69556156 | transcript | SSHX120002397.g |
| SSHX02 | 92933717 | 92934685 | transcript | SSHX020047064.g |
| SSHX10 | 10053373 | 10057487 | transcript | SSHX100056361.g |
| SSHX03 | 25308483 | 25319337 | transcript | SSHX030030108.g |
| SSHX02 | 13583959 | 13603855 | transcript | SSHX020044372.g |
| SSHX19 | 33670588 | 33671077 | transcript | SSHX190012355.g |
| SSHX19 | 33670866 | 33671077 | exon | SSHX190012355.g |
| SSHX19 | 33670866 | 33671077 | CDS | SSHX190012355.g |
| SSHX01 | 99760957 | 99764556 | transcript | SSHX010064090.g |
| SSHX03 | 10935285 | 10942915 | transcript | SSHX030029211.g |
| SSHX05 | 80836516 | 80841157 | transcript | SSHX050036110.g |
| SSHX16 | 45469367 | 45474915 | transcript | SSHX160060283.g |
| SSHX05 | 91718523 | 91736303 | transcript | SSHX050036773.g |
| SSHX01 | 111221597 | 111223273 | transcript | SSHX010064563.g |
| SSHX01 | 111221597 | 111221799 | exon | SSHX010064563.g |
| SSHX01 | 111221597 | 111221799 | CDS | SSHX010064563.g |
| SSHX12 | 76150147 | 76157310 | transcript | SSHX120002826.g |
| SSHX01 | 190195603 | 190203608 | transcript | SSHX010067816.g |
| SSHX10 | 99052978 | 99063518 | transcript | SSHX100058940.g |
| SSHX13 | 792990 | 796784 | transcript | SSHX130018533.g |
| SSHX07 | 107472501 | 107479729 | transcript | SSHX070055426.g |
| SSHX16 | 48980098 | 48986323 | transcript | SSHX160060398.g |
| SSHX08 | 106135534 | 106142559 | transcript | SSHX080040957.g |
| SSHX17 | 38418980 | 38427625 | transcript | SSHX170043545.g |
| SSHX10 | 85624838 | 85633586 | transcript | SSHX100058203.g |
| SSHX01 | 186094262 | 186096883 | transcript | SSHX010067551.g |
| SSHX13 | 12996552 | 12997098 | transcript | SSHX130019348.g |
| SSHX13 | 12996686 | 12997098 | exon | SSHX130019348.g |
| SSHX13 | 12996686 | 12997098 | CDS | SSHX130019348.g |
| SSHX18 | 62400052 | 62400425 | transcript | SSHX180015334.g |
| SSHX18 | 62400168 | 62400425 | exon | SSHX180015334.g |
| SSHX18 | 62400168 | 62400425 | CDS | SSHX180015334.g |
| SSHX09 | 98251675 | 98262671 | transcript | SSHX090024816.g |
| SSHX01 | 157617472 | 157620568 | transcript | SSHX010066578.g |
| SSHX02 | 161088040 | 161091218 | transcript | SSHX020048557.g |
| SSHX09 | 84377707 | 84384679 | transcript | SSHX090024216.g |
| SSHX01 | 185696160 | 185717075 | transcript | SSHX010067516.g |
| SSHX19 | 62910119 | 62923263 | transcript | SSHX190013977.g |
| SSHX16 | 76246329 | 76248880 | transcript | SSHX160061781.g |
| SSHX15 | 3019614 | 3029377 | transcript | SSHX150016144.g |
| SSHX06 | 10773854 | 10787207 | transcript | SSHX060049687.g |
| SSHX03 | 136590694 | 136594776 | transcript | SSHX030032390.g |
| SSHX19 | 37621848 | 37626390 | transcript | SSHX190012506.g |
| SSHX11 | 63399311 | 63409840 | transcript | SSHX110027112.g |
| SSHX07 | 105443177 | 105457422 | transcript | SSHX070055316.g |
| SSHX16 | 66305739 | 66312493 | transcript | SSHX160061220.g |
| SSHX19 | 41333879 | 41337074 | transcript | SSHX190012681.g |
| SSHX08 | 46657290 | 46669020 | transcript | SSHX080039329.g |
| SSHX01 | 117215942 | 117223373 | transcript | SSHX010064906.g |
| SSHX05 | 92506597 | 92507467 | transcript | SSHX050036815.g |
| SSHX17 | 25948246 | 25953821 | transcript | SSHX170043003.g |
| SSHX06 | 122392343 | 122398181 | transcript | SSHX060052761.g |
| SSHX15 | 78199887 | 78210914 | transcript | SSHX150018279.g |
| SSHX12 | 60244665 | 60255249 | transcript | SSHX120001861.g |
| SSHX11 | 84132172 | 84139202 | transcript | SSHX110028281.g |
| SSHX06 | 28539410 | 28543473 | transcript | SSHX060050566.g |
| SSHX06 | 117174607 | 117181022 | transcript | SSHX060052429.g |
| SSHX11 | 76239736 | 76254448 | transcript | SSHX110027857.g |
| SSHX09 | 16865424 | 16871467 | transcript | SSHX090022414.g |
| SSHX09 | 16871318 | 16871467 | exon | SSHX090022414.g |
| SSHX09 | 16871318 | 16871467 | CDS | SSHX090022414.g |
| SSHX04 | 75108469 | 75110066 | transcript | SSHX040006209.g |
| SSHX09 | 97307243 | 97313478 | transcript | SSHX090024737.g |
| SSHX01 | 110707004 | 110711698 | transcript | SSHX010064534.g |
| SSHX13 | 36935973 | 36942754 | transcript | SSHX130020613.g |
| SSHX06 | 24143722 | 24155638 | transcript | SSHX060050357.g |
| SSHX16 | 42454757 | 42461373 | transcript | SSHX160060221.g |
| SSHX03 | 94376555 | 94377249 | transcript | SSHX030031388.g |
| SSHX14 | 2963100 | 2978456 | transcript | SSHX140008624.g |
| SSHX16 | 69979075 | 69987693 | transcript | SSHX160061397.g |
| SSHX12 | 70775314 | 70792729 | transcript | SSHX120002467.g |
| SSHX17 | 23821270 | 23831361 | transcript | SSHX170042898.g |
| SSHX05 | 91693106 | 91695473 | transcript | SSHX050036767.g |
| SSHX17 | 10972012 | 10991301 | transcript | SSHX170042169.g |
| SSHX10 | 103190010 | 103197676 | transcript | SSHX100059228.g |
| SSHX02 | 77110150 | 77114380 | transcript | SSHX020046690.g |
| SSHX03 | 53787920 | 53808560 | transcript | SSHX030030947.g |
| SSHX16 | 72368836 | 72373023 | transcript | SSHX160061548.g |
| SSHX13 | 31627864 | 31629582 | transcript | SSHX130020380.g |
| SSHX13 | 31629054 | 31629189 | exon | SSHX130020380.g |
| SSHX13 | 31629054 | 31629189 | CDS | SSHX130020380.g |
| SSHX01 | 122029545 | 122048407 | transcript | SSHX010065279.g |
| SSHX07 | 98176788 | 98179133 | transcript | SSHX070054924.g |
| SSHX05 | 105509748 | 105510221 | transcript | SSHX050037307.g |
| SSHX05 | 105509748 | 105509974 | exon | SSHX050037307.g |
| SSHX05 | 105509748 | 105509974 | CDS | SSHX050037307.g |
| SSHX02 | 165292708 | 165308162 | transcript | SSHX020048817.g |
| SSHX06 | 104012767 | 104014076 | transcript | SSHX060051832.g |
| SSHX04 | 120307164 | 120309027 | transcript | SSHX040007443.g |
| SSHX08 | 103932020 | 103940110 | transcript | SSHX080040858.g |
| SSHX05 | 92854435 | 92863213 | transcript | SSHX050036823.g |
| SSHX04 | 55403354 | 55413403 | transcript | SSHX040005285.g |
| SSHX13 | 33710425 | 33711001 | transcript | SSHX130020482.g |
| SSHX13 | 33710705 | 33711001 | exon | SSHX130020482.g |
| SSHX13 | 33710705 | 33711001 | CDS | SSHX130020482.g |
| SSHX18 | 40591526 | 40596962 | transcript | SSHX180014458.g |
| SSHX19 | 46337793 | 46343741 | transcript | SSHX190012962.g |
| SSHX11 | 72315674 | 72320267 | transcript | SSHX110027633.g |
| SSHX14 | 69015235 | 69016369 | transcript | SSHX140011631.g |
| SSHX09 | 84570292 | 84579579 | transcript | SSHX090024219.g |
| SSHX02 | 57402314 | 57407842 | transcript | SSHX020045673.g |
| SSHX11 | 80694983 | 80708022 | transcript | SSHX110028070.g |
| SSHX05 | 72998239 | 73000040 | transcript | SSHX050035611.g |
| SSHX05 | 72998239 | 72998731 | exon | SSHX050035611.g |
| SSHX05 | 72998239 | 72998731 | CDS | SSHX050035611.g |
| SSHX13 | 4939142 | 4949994 | transcript | SSHX130018825.g |
| SSHX11 | 23029790 | 23032821 | transcript | SSHX110025566.g |
| SSHX02 | 167204047 | 167215050 | transcript | SSHX020048923.g |
| SSHX06 | 6100357 | 6107133 | transcript | SSHX060049401.g |
| SSHX19 | 47122405 | 47135159 | transcript | SSHX190013003.g |
| SSHX01 | 110938745 | 110952727 | transcript | SSHX010064546.g |
| SSHX01 | 103635348 | 103641858 | transcript | SSHX010064198.g |
| SSHX13 | 11708933 | 11711378 | transcript | SSHX130019251.g |
| SSHX14 | 27955488 | 27958589 | transcript | SSHX140010196.g |
| SSHX13 | 73677541 | 73685323 | transcript | SSHX130021183.g |
| SSHX11 | 66731793 | 66741385 | transcript | SSHX110027304.g |
| SSHX09 | 29336200 | 29337004 | transcript | SSHX090023117.g |
| SSHX05 | 68986909 | 68993167 | transcript | SSHX050035404.g |
| SSHX06 | 3372883 | 3380027 | transcript | SSHX060049297.g |
| SSHX04 | 56079195 | 56086430 | transcript | SSHX040005347.g |
| SSHX05 | 93203361 | 93217988 | transcript | SSHX050036852.g |
| SSHX08 | 50691203 | 50696898 | transcript | SSHX080039475.g |
| SSHX15 | 68895787 | 68900029 | transcript | SSHX150017813.g |
| SSHX01 | 59522764 | 59536204 | transcript | SSHX010063017.g |
| SSHX04 | 131749200 | 131753124 | transcript | SSHX040008184.g |
| SSHX01 | 187938756 | 187942764 | transcript | SSHX010067655.g |
| SSHX03 | 142658531 | 142660208 | transcript | SSHX030032680.g |
| SSHX19 | 12071596 | 12082699 | transcript | SSHX190011977.g |
| SSHX10 | 56404733 | 56405437 | transcript | SSHX100057176.g |
| SSHX11 | 63499958 | 63503743 | transcript | SSHX110027119.g |
| SSHX08 | 73161817 | 73171594 | transcript | SSHX080040099.g |
| SSHX18 | 43692939 | 43704622 | transcript | SSHX180014549.g |
| SSHX01 | 189508078 | 189508791 | transcript | SSHX010067773.g |
| SSHX01 | 189508078 | 189508791 | exon | SSHX010067773.g |
| SSHX01 | 189508078 | 189508791 | CDS | SSHX010067773.g |
| SSHX02 | 161627393 | 161635861 | transcript | SSHX020048603.g |
| SSHX06 | 3131565 | 3141605 | transcript | SSHX060049287.g |
| SSHX11 | 58952731 | 58965780 | transcript | SSHX110026829.g |
| SSHX17 | 1853387 | 1865462 | transcript | SSHX170041575.g |
| SSHX01 | 122868965 | 122875990 | transcript | SSHX010065335.g |
| SSHX01 | 28203956 | 28212332 | transcript | SSHX010062196.g |
| SSHX19 | 59011664 | 59020969 | transcript | SSHX190013733.g |
| SSHX13 | 47705793 | 47706958 | transcript | SSHX130020939.g |
| SSHX01 | 129881139 | 129887555 | transcript | SSHX010065808.g |
| SSHX19 | 62550566 | 62558731 | transcript | SSHX190013956.g |
| SSHX03 | 145858034 | 145865561 | transcript | SSHX030032897.g |
| SSHX10 | 24125432 | 24128721 | transcript | SSHX100056778.g |
| SSHX02 | 166162129 | 166169928 | transcript | SSHX020048855.g |
| SSHX02 | 161984534 | 161998677 | transcript | SSHX020048632.g |
| SSHX01 | 139605147 | 139610727 | transcript | SSHX010066337.g |
| SSHX03 | 15673061 | 15684184 | transcript | SSHX030029542.g |
| SSHX01 | 186797631 | 186802509 | transcript | SSHX010067583.g |
| SSHX10 | 2419181 | 2425735 | transcript | SSHX100056001.g |
| SSHX03 | 25473766 | 25477465 | transcript | SSHX030030122.g |
| SSHX19 | 46518600 | 46524310 | transcript | SSHX190012973.g |
| SSHX03 | 145208761 | 145218998 | transcript | SSHX030032845.g |
| SSHX16 | 75494713 | 75508148 | transcript | SSHX160061733.g |
| SSHX05 | 49021123 | 49023346 | transcript | SSHX050034126.g |
| SSHX15 | 3184437 | 3191343 | transcript | SSHX150016153.g |
| SSHX11 | 67616804 | 67622261 | transcript | SSHX110027353.g |
| SSHX17 | 4610247 | 4613820 | transcript | SSHX170041771.g |
| SSHX02 | 50027610 | 50029487 | transcript | SSHX020045201.g |
| SSHX03 | 3440462 | 3444407 | transcript | SSHX030028707.g |
| SSHX03 | 120923639 | 120928353 | transcript | SSHX030031789.g |
| SSHX15 | 72934322 | 72943729 | transcript | SSHX150017977.g |
| SSHX02 | 65385709 | 65393962 | transcript | SSHX020046153.g |
| SSHX11 | 87933938 | 87938775 | transcript | SSHX110028423.g |
| SSHX11 | 87933938 | 87934164 | exon | SSHX110028423.g |
| SSHX11 | 87933938 | 87934164 | CDS | SSHX110028423.g |
| SSHX14 | 20375115 | 20380121 | transcript | SSHX140009660.g |
| SSHX05 | 64988939 | 64999539 | transcript | SSHX050035115.g |
| SSHX19 | 56549178 | 56553541 | transcript | SSHX190013581.g |
| SSHX03 | 140631006 | 140638105 | transcript | SSHX030032600.g |
| SSHX04 | 132937150 | 132946860 | transcript | SSHX040008248.g |
| SSHX14 | 9505848 | 9512012 | transcript | SSHX140009034.g |
| SSHX14 | 16866404 | 16870410 | transcript | SSHX140009458.g |
| SSHX06 | 120537546 | 120538873 | transcript | SSHX060052626.g |
| SSHX17 | 6464025 | 6468615 | transcript | SSHX170041902.g |
| SSHX19 | 52301061 | 52302982 | transcript | SSHX190013300.g |
| SSHX14 | 27090642 | 27091678 | transcript | SSHX140010125.g |
| SSHX14 | 27091451 | 27091678 | exon | SSHX140010125.g |
| SSHX14 | 27091451 | 27091678 | CDS | SSHX140010125.g |
| SSHX03 | 6581135 | 6582052 | transcript | SSHX030028930.g |
| SSHX07 | 99167158 | 99167378 | transcript | SSHX070054988.g |
| SSHX09 | 518221 | 525250 | transcript | SSHX090021413.g |
| SSHX18 | 52928203 | 52937696 | transcript | SSHX180014840.g |
| SSHX03 | 8242876 | 8252171 | transcript | SSHX030029016.g |
| SSHX06 | 111339003 | 111342970 | transcript | SSHX060052187.g |
| SSHX11 | 70089727 | 70097001 | transcript | SSHX110027511.g |
| SSHX03 | 51672844 | 51675574 | transcript | SSHX030030872.g |
| SSHX06 | 99372915 | 99381348 | transcript | SSHX060051621.g |
| SSHX12 | 80506078 | 80511755 | transcript | SSHX120003104.g |
| SSHX17 | 15647968 | 15654078 | transcript | SSHX170042428.g |
| SSHX01 | 128106634 | 128114768 | transcript | SSHX010065678.g |
| SSHX11 | 61419191 | 61428505 | transcript | SSHX110026998.g |
| SSHX05 | 66175581 | 66179596 | transcript | SSHX050035195.g |
| SSHX03 | 2237023 | 2246899 | transcript | SSHX030028629.g |
| SSHX05 | 107503294 | 107509301 | transcript | SSHX050037353.g |
| SSHX05 | 47641144 | 47642096 | transcript | SSHX050034057.g |
| SSHX05 | 47641916 | 47642096 | exon | SSHX050034057.g |
| SSHX05 | 47641916 | 47642096 | CDS | SSHX050034057.g |
| SSHX09 | 1577827 | 1590931 | transcript | SSHX090021468.g |
| SSHX09 | 89779439 | 89784886 | transcript | SSHX090024438.g |
| SSHX01 | 160853817 | 160854938 | transcript | SSHX010066632.g |
| SSHX13 | 12140141 | 12145397 | transcript | SSHX130019280.g |
| SSHX06 | 11390528 | 11400471 | transcript | SSHX060049718.g |
| SSHX17 | 17754898 | 17765148 | transcript | SSHX170042543.g |
| SSHX03 | 146743586 | 146748152 | transcript | SSHX030032948.g |
| SSHX12 | 53601052 | 53619147 | transcript | SSHX120001525.g |
| SSHX07 | 77035468 | 77039142 | transcript | SSHX070054303.g |
| SSHX07 | 21779911 | 21780479 | transcript | SSHX070053718.g |
| SSHX08 | 51275824 | 51276220 | transcript | SSHX080039531.g |
| SSHX08 | 51276095 | 51276220 | exon | SSHX080039531.g |
| SSHX08 | 51276095 | 51276220 | CDS | SSHX080039531.g |
| SSHX06 | 13713225 | 13718178 | transcript | SSHX060049825.g |
| SSHX19 | 24430377 | 24436992 | transcript | SSHX190012146.g |
| SSHX07 | 106057822 | 106059238 | transcript | SSHX070055349.g |
| SSHX01 | 63460734 | 63467066 | transcript | SSHX010063228.g |
| SSHX16 | 57894189 | 57895952 | transcript | SSHX160060819.g |
| SSHX01 | 60340820 | 60344307 | transcript | SSHX010063050.g |
| SSHX03 | 141356523 | 141368285 | transcript | SSHX030032619.g |
| SSHX09 | 15051537 | 15069686 | transcript | SSHX090022262.g |
| SSHX10 | 94539948 | 94543671 | transcript | SSHX100058672.g |
| SSHX08 | 107006815 | 107015669 | transcript | SSHX080041011.g |
| SSHX06 | 6625999 | 6629801 | transcript | SSHX060049445.g |
| SSHX02 | 42324111 | 42335248 | transcript | SSHX020044884.g |
| SSHX03 | 147928743 | 147928870 | exon | SSHX030033037.g |
| SSHX03 | 147928743 | 147928870 | CDS | SSHX030033037.g |
| SSHX03 | 147923630 | 147931144 | transcript | SSHX030033037.g |
| SSHX01 | 106592344 | 106598679 | transcript | SSHX010064322.g |
| SSHX07 | 111365579 | 111367886 | transcript | SSHX070055589.g |
| SSHX09 | 94822127 | 94841674 | transcript | SSHX090024629.g |
| SSHX02 | 167916374 | 167919067 | transcript | SSHX020048949.g |
| SSHX17 | 25370877 | 25373932 | transcript | SSHX170042979.g |
| SSHX12 | 61949299 | 61954124 | transcript | SSHX120001946.g |
| SSHX11 | 731673 | 731963 | transcript | SSHX110025389.g |
| SSHX11 | 731673 | 731963 | exon | SSHX110025389.g |
| SSHX11 | 731673 | 731963 | CDS | SSHX110025389.g |
| SSHX15 | 18408308 | 18410636 | transcript | SSHX150016822.g |
| SSHX12 | 78352666 | 78357387 | transcript | SSHX120002941.g |
| SSHX09 | 31984858 | 31994791 | transcript | SSHX090023209.g |
| SSHX10 | 20317060 | 20322296 | transcript | SSHX100056685.g |
| SSHX07 | 110901063 | 110912300 | transcript | SSHX070055574.g |
| SSHX13 | 15003074 | 15008044 | transcript | SSHX130019466.g |
| SSHX08 | 46716851 | 46720443 | transcript | SSHX080039330.g |
| SSHX13 | 14366612 | 14369173 | transcript | SSHX130019435.g |
| SSHX12 | 79896600 | 79903896 | transcript | SSHX120003052.g |
| SSHX11 | 39914502 | 39915474 | transcript | SSHX110025932.g |
| SSHX13 | 24803492 | 24812689 | transcript | SSHX130019952.g |
| SSHX01 | 128561510 | 128571103 | transcript | SSHX010065710.g |
| SSHX13 | 26616438 | 26619022 | transcript | SSHX130020078.g |
| SSHX04 | 130051990 | 130059814 | transcript | SSHX040008072.g |
| SSHX14 | 6263204 | 6268461 | transcript | SSHX140008818.g |
| SSHX12 | 66089793 | 66095210 | transcript | SSHX120002194.g |
| SSHX08 | 32416124 | 32422319 | transcript | SSHX080038341.g |
| SSHX03 | 134941545 | 134952539 | transcript | SSHX030032311.g |
| SSHX08 | 58926262 | 58934978 | transcript | SSHX080039844.g |
| SSHX14 | 43057941 | 43059118 | transcript | SSHX140011018.g |
| SSHX14 | 43058947 | 43059118 | exon | SSHX140011018.g |
| SSHX14 | 43058947 | 43059118 | CDS | SSHX140011018.g |
| SSHX14 | 46446984 | 46453330 | transcript | SSHX140011178.g |
| SSHX11 | 78325966 | 78332748 | transcript | SSHX110027969.g |
| SSHX04 | 41331103 | 41338418 | transcript | SSHX040004380.g |
| SSHX14 | 27012086 | 27014548 | transcript | SSHX140010115.g |
| SSHX10 | 41503096 | 41509769 | transcript | SSHX100056941.g |
| SSHX05 | 11678114 | 11683019 | transcript | SSHX050033217.g |
| SSHX04 | 39681831 | 39693839 | transcript | SSHX040004278.g |
| SSHX09 | 20679198 | 20685291 | transcript | SSHX090022662.g |
| SSHX13 | 22508156 | 22515451 | transcript | SSHX130019860.g |
| SSHX12 | 39510331 | 39513121 | transcript | SSHX120000830.g |
| SSHX18 | 52928203 | 52937696 | transcript | SSHX180014840.g |
| SSHX08 | 106930123 | 106942984 | transcript | SSHX080041007.g |
| SSHX14 | 45402813 | 45404404 | transcript | SSHX140011131.g |
| SSHX14 | 1100604 | 1105391 | transcript | SSHX140008516.g |
| SSHX18 | 70452046 | 70459180 | transcript | SSHX180015764.g |
| SSHX01 | 131289233 | 131290053 | transcript | SSHX010065871.g |
| SSHX14 | 5558653 | 5560331 | transcript | SSHX140008780.g |
| SSHX14 | 5558954 | 5559227 | exon | SSHX140008780.g |
| SSHX14 | 5558954 | 5559227 | CDS | SSHX140008780.g |
| SSHX02 | 51115368 | 51124218 | transcript | SSHX020045269.g |
| SSHX19 | 36954198 | 36958043 | transcript | SSHX190012486.g |
| SSHX12 | 44563114 | 44571268 | transcript | SSHX120001065.g |
| SSHX12 | 75793926 | 75794970 | transcript | SSHX120002792.g |
| SSHX12 | 75794824 | 75794970 | exon | SSHX120002792.g |
| SSHX12 | 75794824 | 75794970 | CDS | SSHX120002792.g |
| SSHX09 | 23898572 | 23904227 | transcript | SSHX090022860.g |
| SSHX02 | 56796563 | 56804401 | transcript | SSHX020045623.g |
| SSHX14 | 31865132 | 31874315 | transcript | SSHX140010436.g |
| SSHX12 | 73540194 | 73552567 | transcript | SSHX120002660.g |
| SSHX02 | 47225273 | 47233668 | transcript | SSHX020045083.g |
| SSHX02 | 47232357 | 47232529 | exon | SSHX020045083.g |
| SSHX02 | 47232357 | 47232529 | CDS | SSHX020045083.g |
| SSHX10 | 83847383 | 83850520 | transcript | SSHX100058113.g |
| SSHX07 | 94784848 | 94794169 | transcript | SSHX070054787.g |
| SSHX18 | 72917092 | 72925190 | transcript | SSHX180015924.g |
| SSHX03 | 20530605 | 20535787 | transcript | SSHX030029822.g |
| SSHX07 | 103668452 | 103675061 | transcript | SSHX070055212.g |
| SSHX08 | 47232816 | 47238343 | transcript | SSHX080039356.g |
| SSHX03 | 2627125 | 2636956 | transcript | SSHX030028653.g |
| SSHX19 | 41538083 | 41540061 | transcript | SSHX190012688.g |
| SSHX05 | 64668786 | 64676327 | transcript | SSHX050035082.g |
| SSHX11 | 36258112 | 36260120 | transcript | SSHX110025832.g |
| SSHX14 | 8846484 | 8855247 | transcript | SSHX140008991.g |
| SSHX16 | 26180418 | 26192457 | transcript | SSHX160059939.g |
| SSHX02 | 165730013 | 165739957 | transcript | SSHX020048837.g |
| SSHX07 | 36860019 | 36863145 | transcript | SSHX070053912.g |
| SSHX10 | 74843619 | 74859368 | transcript | SSHX100057594.g |
| SSHX15 | 76263179 | 76277534 | transcript | SSHX150018160.g |
| SSHX04 | 43946132 | 43956212 | transcript | SSHX040004532.g |
| SSHX10 | 40630240 | 40630536 | transcript | SSHX100056933.g |
| SSHX06 | 11974501 | 11978232 | transcript | SSHX060049741.g |
| SSHX11 | 79327550 | 79328857 | transcript | SSHX110028007.g |
| SSHX16 | 41746946 | 41754927 | transcript | SSHX160060203.g |
| SSHX03 | 5424782 | 5427858 | transcript | SSHX030028846.g |
| SSHX05 | 87543786 | 87551753 | transcript | SSHX050036537.g |
| SSHX02 | 162675175 | 162681222 | transcript | SSHX020048683.g |
| SSHX12 | 72633301 | 72638314 | transcript | SSHX120002616.g |
| SSHX17 | 26282676 | 26288061 | transcript | SSHX170043021.g |
| SSHX02 | 161637864 | 161642463 | transcript | SSHX020048604.g |
| SSHX01 | 22164915 | 22165180 | transcript | SSHX010062130.g |
| SSHX06 | 8745039 | 8746445 | transcript | SSHX060049550.g |
| SSHX02 | 112616777 | 112616944 | transcript | SSHX020047451.g |
| SSHX02 | 112616777 | 112616944 | exon | SSHX020047451.g |
| SSHX02 | 112616777 | 112616944 | CDS | SSHX020047451.g |
| SSHX14 | 6918135 | 6925456 | transcript | SSHX140008869.g |
| SSHX03 | 3740351 | 3740732 | transcript | SSHX030028727.g |
| SSHX11 | 59870243 | 59871736 | transcript | SSHX110026883.g |
| SSHX14 | 15022998 | 15024219 | transcript | SSHX140009345.g |
| SSHX14 | 34394802 | 34403433 | transcript | SSHX140010586.g |
| SSHX02 | 152513291 | 152516236 | transcript | SSHX020048155.g |
| SSHX01 | 183389275 | 183394973 | transcript | SSHX010067383.g |
| SSHX10 | 86808020 | 86815762 | transcript | SSHX100058293.g |
| SSHX01 | 120382213 | 120393265 | transcript | SSHX010065124.g |
| SSHX10 | 25030711 | 25034352 | transcript | SSHX100056801.g |
| SSHX06 | 113857227 | 113865128 | transcript | SSHX060052293.g |
| SSHX06 | 10479957 | 10484668 | transcript | SSHX060049668.g |
| SSHX12 | 22428906 | 22432006 | transcript | SSHX120000361.g |
| SSHX08 | 109210730 | 109215327 | transcript | SSHX080041127.g |
| SSHX03 | 2237023 | 2246899 | transcript | SSHX030028629.g |
| SSHX13 | 21010387 | 21020546 | transcript | SSHX130019794.g |
| SSHX17 | 24682352 | 24687793 | transcript | SSHX170042949.g |
| SSHX04 | 128318205 | 128318535 | transcript | SSHX040007960.g |
| SSHX04 | 128318353 | 128318535 | exon | SSHX040007960.g |
| SSHX04 | 128318353 | 128318535 | CDS | SSHX040007960.g |
| SSHX09 | 8384947 | 8387751 | transcript | SSHX090021820.g |
| SSHX01 | 187970424 | 187980470 | transcript | SSHX010067662.g |
| SSHX02 | 51040945 | 51056232 | transcript | SSHX020045264.g |
| SSHX07 | 7410169 | 7415671 | transcript | SSHX070053270.g |
| SSHX11 | 76053430 | 76064350 | transcript | SSHX110027854.g |
| SSHX01 | 120236482 | 120242302 | transcript | SSHX010065112.g |
| SSHX03 | 30424750 | 30427392 | transcript | SSHX030030373.g |
| SSHX05 | 48632171 | 48636047 | transcript | SSHX050034112.g |
| SSHX13 | 22137682 | 22145855 | transcript | SSHX130019842.g |
| SSHX04 | 60354492 | 60357676 | transcript | SSHX040005618.g |
| SSHX01 | 5789750 | 5799804 | transcript | SSHX010062010.g |
| SSHX01 | 138708825 | 138716834 | transcript | SSHX010066289.g |
| SSHX07 | 101380936 | 101386299 | transcript | SSHX070055080.g |
| SSHX10 | 7465876 | 7469470 | transcript | SSHX100056282.g |
| SSHX07 | 627898 | 638653 | transcript | SSHX070052881.g |
| SSHX11 | 38914759 | 38922302 | transcript | SSHX110025888.g |
| SSHX13 | 112882 | 113904 | transcript | SSHX130018486.g |
| SSHX13 | 113458 | 113677 | exon | SSHX130018486.g |
| SSHX13 | 113458 | 113677 | CDS | SSHX130018486.g |
| SSHX10 | 95205131 | 95206098 | transcript | SSHX100058713.g |
| SSHX10 | 95205877 | 95206098 | exon | SSHX100058713.g |
| SSHX10 | 95205877 | 95206098 | CDS | SSHX100058713.g |
| SSHX17 | 31618284 | 31625765 | transcript | SSHX170043313.g |
| SSHX04 | 54091428 | 54101527 | transcript | SSHX040005190.g |
| SSHX15 | 2669490 | 2672366 | transcript | SSHX150016112.g |
| SSHX17 | 15598985 | 15616243 | transcript | SSHX170042425.g |
| SSHX05 | 79884326 | 79891203 | transcript | SSHX050036050.g |
| SSHX15 | 81100889 | 81106686 | transcript | SSHX150018447.g |
| SSHX01 | 127243966 | 127250106 | transcript | SSHX010065619.g |
| SSHX09 | 4835687 | 4838239 | transcript | SSHX090021642.g |
| SSHX04 | 134380363 | 134388760 | transcript | SSHX040008364.g |
| SSHX19 | 34774810 | 34780860 | transcript | SSHX190012404.g |
| SSHX15 | 64860720 | 64866227 | transcript | SSHX150017676.g |
| SSHX08 | 182352 | 188336 | transcript | SSHX080037600.g |
| SSHX03 | 13422282 | 13427897 | transcript | SSHX030029388.g |
| SSHX01 | 111327973 | 111333038 | transcript | SSHX010064568.g |
| SSHX08 | 100647263 | 100657462 | transcript | SSHX080040736.g |
| SSHX19 | 40637468 | 40639235 | transcript | SSHX190012654.g |
| SSHX04 | 71006179 | 71014446 | transcript | SSHX040006090.g |
| SSHX17 | 40872833 | 40878068 | transcript | SSHX170043679.g |
| SSHX05 | 66245236 | 66254222 | transcript | SSHX050035202.g |
| SSHX17 | 35371449 | 35381673 | transcript | SSHX170043445.g |
| SSHX19 | 35415677 | 35424469 | transcript | SSHX190012431.g |
| SSHX01 | 70628907 | 70629506 | transcript | SSHX010063604.g |
| SSHX01 | 70628907 | 70629158 | exon | SSHX010063604.g |
| SSHX01 | 70628907 | 70629158 | CDS | SSHX010063604.g |
| SSHX18 | 2631806 | 2639154 | transcript | SSHX180014034.g |
| SSHX06 | 81368684 | 81371738 | transcript | SSHX060051357.g |
| SSHX17 | 4583016 | 4596326 | transcript | SSHX170041769.g |
| SSHX01 | 66555192 | 66557394 | transcript | SSHX010063460.g |
| SSHX12 | 83221255 | 83224493 | transcript | SSHX120003293.g |
| SSHX03 | 144680825 | 144685686 | transcript | SSHX030032803.g |
| SSHX12 | 45605205 | 45609762 | transcript | SSHX120001126.g |
| SSHX16 | 76976353 | 76984169 | transcript | SSHX160061845.g |
| SSHX01 | 54544596 | 54552728 | transcript | SSHX010062821.g |
| SSHX12 | 43247257 | 43257362 | transcript | SSHX120001006.g |
| SSHX13 | 10622314 | 10625622 | transcript | SSHX130019186.g |
| SSHX19 | 51201621 | 51201836 | transcript | SSHX190013238.g |
| SSHX19 | 51201621 | 51201836 | exon | SSHX190013238.g |
| SSHX19 | 51201621 | 51201836 | CDS | SSHX190013238.g |
| SSHX07 | 105814231 | 105814365 | transcript | SSHX070055340.g |
| SSHX07 | 105814231 | 105814365 | exon | SSHX070055340.g |
| SSHX07 | 105814231 | 105814365 | CDS | SSHX070055340.g |
| SSHX01 | 64147389 | 64154245 | transcript | SSHX010063283.g |
| SSHX17 | 29212451 | 29221775 | transcript | SSHX170043195.g |
| SSHX03 | 139583510 | 139586803 | transcript | SSHX030032533.g |
| SSHX03 | 126181313 | 126184507 | transcript | SSHX030031965.g |
| SSHX03 | 126181480 | 126181682 | exon | SSHX030031965.g |
| SSHX03 | 126181480 | 126181682 | CDS | SSHX030031965.g |
| SSHX10 | 66892285 | 66892622 | transcript | SSHX100057373.g |
| SSHX10 | 66892285 | 66892512 | exon | SSHX100057373.g |
| SSHX10 | 66892285 | 66892512 | CDS | SSHX100057373.g |
| SSHX02 | 146770734 | 146780565 | transcript | SSHX020047937.g |
| SSHX02 | 124270663 | 124271485 | transcript | SSHX020047573.g |
| SSHX05 | 92836330 | 92843300 | transcript | SSHX050036822.g |
| SSHX01 | 66252465 | 66254514 | transcript | SSHX010063433.g |
| SSHX17 | 26147987 | 26154856 | transcript | SSHX170043010.g |
| SSHX09 | 20034726 | 20036864 | transcript | SSHX090022617.g |
| SSHX09 | 102082394 | 102088280 | transcript | SBP |
| SSHX13 | 2752465 | 2765708 | transcript | C3H |
| SSHX13 | 14711302 | 14718446 | transcript | bHLH |
| SSHX06 | 2314018 | 2315367 | transcript | MYB_related |
| SSHX10 | 92476215 | 92480891 | transcript | HD-ZIP |
| SSHX16 | 76946042 | 76946561 | transcript | MYB_related |
| SSHX02 | 168146528 | 168152500 | transcript | bZIP |
| SSHX09 | 98256453 | 98256640 | transcript | ARF |
| SSHX08 | 53516186 | 53518065 | transcript | bHLH |
| SSHX06 | 7967529 | 7971600 | transcript | bHLH |
| SSHX09 | 97308621 | 97310438 | transcript | G2-like |
| SSHX04 | 55409310 | 55409765 | transcript | CAMTA |
| SSHX02 | 167916990 | 167917082 | transcript | HD-ZIP |
| SSHX08 | 32417134 | 32417551 | transcript | G2-like |
| SSHX07 | 103671557 | 103671738 | transcript | G2-like |
| SSHX06 | 81369452 | 81369592 | transcript | NF-YA |

**F.**

| ***Michelia montaua*_CHH** | | | | |
| --- | --- | --- | --- | --- |
| SSHX01 | 15925655 | 15926922 | transcript | SSHX010062084.g |
| SSHX01 | 32716634 | 32717942 | transcript | SSHX010062261.g |
| SSHX01 | 43461255 | 43466110 | transcript | SSHX010062509.g |
| SSHX01 | 50761157 | 50769751 | transcript | SSHX010062673.g |
| SSHX01 | 53277708 | 53282746 | transcript | SSHX010062777.g |
| SSHX01 | 61081037 | 61089010 | transcript | SSHX010063087.g |
| SSHX01 | 62950032 | 62959410 | transcript | SSHX010063195.g |
| SSHX01 | 64976432 | 64984391 | transcript | SSHX010063358.g |
| SSHX01 | 67743467 | 67746889 | transcript | SSHX010063493.g |
| SSHX01 | 105524473 | 105532156 | transcript | SSHX010064256.g |
| SSHX01 | 108306134 | 108316051 | transcript | SSHX010064406.g |
| SSHX01 | 108358313 | 108364586 | transcript | SSHX010064409.g |
| SSHX01 | 108423958 | 108429967 | transcript | SSHX010064412.g |
| SSHX01 | 114945820 | 114953769 | transcript | SSHX010064782.g |
| SSHX01 | 124295367 | 124307052 | transcript | SSHX010065427.g |
| SSHX01 | 158385029 | 158393941 | transcript | SSHX010066587.g |
| SSHX02 | 30470496 | 30473373 | transcript | SSHX020044581.g |
| SSHX02 | 33661907 | 33670345 | transcript | SSHX020044644.g |
| SSHX02 | 34565510 | 34566053 | transcript | SSHX020044671.g |
| SSHX02 | 56796563 | 56804401 | transcript | SSHX020045623.g |
| SSHX02 | 61168273 | 61172976 | transcript | SSHX020045886.g |
| SSHX02 | 81436793 | 81438258 | transcript | SSHX020046859.g |
| SSHX02 | 119432343 | 119434343 | transcript | SSHX020047540.g |
| SSHX02 | 154884156 | 154887973 | transcript | SSHX020048258.g |
| SSHX03 | 1030576 | 1034426 | transcript | SSHX030028543.g |
| SSHX03 | 2692521 | 2699456 | transcript | SSHX030028663.g |
| SSHX03 | 4196297 | 4200760 | transcript | SSHX030028772.g |
| SSHX03 | 9445466 | 9457121 | transcript | SSHX030029088.g |
| SSHX03 | 15648981 | 15654212 | transcript | SSHX030029540.g |
| SSHX03 | 15673061 | 15684184 | transcript | SSHX030029542.g |
| SSHX03 | 25308483 | 25319337 | transcript | SSHX030030108.g |
| SSHX03 | 44848422 | 44850936 | transcript | SSHX030030749.g |
| SSHX03 | 57510849 | 57511702 | transcript | SSHX030031048.g |
| SSHX03 | 57511538 | 57511702 | exon | SSHX030031048.g |
| SSHX03 | 57511538 | 57511702 | CDS | SSHX030031048.g |
| SSHX03 | 72252649 | 72262851 | transcript | SSHX030031208.g |
| SSHX03 | 117539517 | 117560053 | transcript | SSHX030031698.g |
| SSHX03 | 131977425 | 131978267 | transcript | SSHX030032159.g |
| SSHX03 | 138841664 | 138842856 | transcript | SSHX030032502.g |
| SSHX03 | 138846529 | 138849718 | transcript | SSHX030032505.g |
| SSHX03 | 139510847 | 139511656 | transcript | SSHX030032527.g |
| SSHX03 | 142554231 | 142562821 | transcript | SSHX030032675.g |
| SSHX03 | 145208761 | 145218998 | transcript | SSHX030032845.g |
| SSHX03 | 147218716 | 147225411 | transcript | SSHX030032983.g |
| SSHX03 | 148348295 | 148353724 | transcript | SSHX030033067.g |
| SSHX04 | 21180232 | 21191571 | transcript | SSHX040003628.g |
| SSHX04 | 28478656 | 28488131 | transcript | SSHX040003784.g |
| SSHX04 | 46215774 | 46225828 | transcript | SSHX040004685.g |
| SSHX04 | 51247807 | 51248501 | transcript | SSHX040004971.g |
| SSHX04 | 51247807 | 51248110 | exon | SSHX040004971.g |
| SSHX04 | 51247807 | 51248110 | CDS | SSHX040004971.g |
| SSHX04 | 55266701 | 55269116 | transcript | SSHX040005268.g |
| SSHX04 | 57208516 | 57216332 | transcript | SSHX040005417.g |
| SSHX04 | 61586989 | 61592637 | transcript | SSHX040005679.g |
| SSHX04 | 68061693 | 68066974 | transcript | SSHX040005974.g |
| SSHX04 | 68291151 | 68293788 | transcript | SSHX040005995.g |
| SSHX04 | 101926357 | 101941347 | transcript | SSHX040006622.g |
| SSHX04 | 127070148 | 127076695 | transcript | SSHX040007905.g |
| SSHX04 | 130781928 | 130782861 | transcript | SSHX040008121.g |
| SSHX05 | 9854162 | 9858788 | transcript | SSHX050033200.g |
| SSHX05 | 33996099 | 34002466 | transcript | SSHX050033607.g |
| SSHX05 | 39083343 | 39090969 | transcript | SSHX050033753.g |
| SSHX05 | 53013426 | 53023300 | transcript | SSHX050034330.g |
| SSHX05 | 53027842 | 53037321 | transcript | SSHX050034331.g |
| SSHX05 | 56494209 | 56504220 | transcript | SSHX050034520.g |
| SSHX05 | 59944639 | 59953994 | transcript | SSHX050034724.g |
| SSHX05 | 65347211 | 65355346 | transcript | SSHX050035135.g |
| SSHX05 | 70090476 | 70095397 | transcript | SSHX050035451.g |
| SSHX05 | 70810781 | 70811925 | transcript | SSHX050035498.g |
| SSHX05 | 79359681 | 79368731 | transcript | SSHX050036027.g |
| SSHX05 | 83555140 | 83562826 | transcript | SSHX050036283.g |
| SSHX05 | 83869994 | 83874270 | transcript | SSHX050036314.g |
| SSHX05 | 84198820 | 84202509 | transcript | SSHX050036346.g |
| SSHX05 | 92299912 | 92313718 | transcript | SSHX050036804.g |
| SSHX05 | 93203361 | 93217988 | transcript | SSHX050036852.g |
| SSHX05 | 96667326 | 96673545 | transcript | SSHX050037048.g |
| SSHX05 | 96835906 | 96837405 | transcript | SSHX050037059.g |
| SSHX06 | 1846040 | 1848614 | transcript | SSHX060049207.g |
| SSHX06 | 9748527 | 9752432 | transcript | SSHX060049615.g |
| SSHX06 | 13923218 | 13933244 | transcript | SSHX060049835.g |
| SSHX06 | 15265063 | 15277216 | transcript | SSHX060049899.g |
| SSHX06 | 28539410 | 28543473 | transcript | SSHX060050566.g |
| SSHX06 | 99372915 | 99381348 | transcript | SSHX060051621.g |
| SSHX06 | 104209156 | 104219579 | transcript | SSHX060051847.g |
| SSHX06 | 114397800 | 114404809 | transcript | SSHX060052320.g |
| SSHX06 | 114527969 | 114542284 | transcript | SSHX060052327.g |
| SSHX07 | 2498252 | 2502171 | transcript | SSHX070053018.g |
| SSHX07 | 2796368 | 2803800 | transcript | SSHX070053044.g |
| SSHX07 | 5096047 | 5099067 | transcript | SSHX070053171.g |
| SSHX07 | 11431259 | 11438290 | transcript | SSHX070053407.g |
| SSHX07 | 12530304 | 12536626 | transcript | SSHX070053451.g |
| SSHX07 | 29349302 | 29358929 | transcript | SSHX070053827.g |
| SSHX07 | 104268520 | 104271175 | transcript | SSHX070055248.g |
| SSHX07 | 104934007 | 104936461 | transcript | SSHX070055281.g |
| SSHX07 | 114996906 | 115007177 | transcript | SSHX070055750.g |
| SSHX08 | 2265292 | 2271567 | transcript | SSHX080037687.g |
| SSHX08 | 3019449 | 3024360 | transcript | SSHX080037723.g |
| SSHX08 | 36120198 | 36124012 | transcript | SSHX080038584.g |
| SSHX08 | 37633717 | 37644684 | transcript | SSHX080038686.g |
| SSHX08 | 39974743 | 39982753 | transcript | SSHX080038866.g |
| SSHX08 | 44646652 | 44652765 | transcript | SSHX080039196.g |
| SSHX08 | 63278807 | 63286240 | transcript | SSHX080039962.g |
| SSHX08 | 101508304 | 101515487 | transcript | SSHX080040774.g |
| SSHX08 | 103619080 | 103626135 | transcript | SSHX080040850.g |
| SSHX08 | 105038264 | 105047249 | transcript | SSHX080040909.g |
| SSHX08 | 107444004 | 107453928 | transcript | SSHX080041042.g |
| SSHX08 | 111518695 | 111519708 | transcript | SSHX080041275.g |
| SSHX08 | 111963538 | 111977598 | transcript | SSHX080041304.g |
| SSHX08 | 113485999 | 113504342 | transcript | SSHX080041425.g |
| SSHX09 | 4717165 | 4718364 | transcript | SSHX090021634.g |
| SSHX09 | 13998276 | 14002036 | transcript | SSHX090022198.g |
| SSHX09 | 21295235 | 21303917 | transcript | SSHX090022698.g |
| SSHX09 | 22644642 | 22649909 | transcript | SSHX090022796.g |
| SSHX09 | 25409999 | 25421736 | transcript | SSHX090022940.g |
| SSHX09 | 31984858 | 31994791 | transcript | SSHX090023209.g |
| SSHX09 | 84906624 | 84909467 | transcript | SSHX090024230.g |
| SSHX09 | 91881179 | 91890957 | transcript | SSHX090024514.g |
| SSHX09 | 93927162 | 93951076 | transcript | SSHX090024587.g |
| SSHX09 | 97895099 | 97906956 | transcript | SSHX090024784.g |
| SSHX09 | 105839957 | 105843162 | transcript | SSHX090025285.g |
| SSHX09 | 106196187 | 106204825 | transcript | SSHX090025319.g |
| SSHX10 | 1870542 | 1881362 | transcript | SSHX100055967.g |
| SSHX10 | 2145063 | 2152111 | transcript | SSHX100055985.g |
| SSHX10 | 2978244 | 2987247 | transcript | SSHX100056037.g |
| SSHX10 | 18918209 | 18919662 | transcript | SSHX100056669.g |
| SSHX10 | 74843619 | 74859368 | transcript | SSHX100057594.g |
| SSHX10 | 99145757 | 99148244 | transcript | SSHX100058947.g |
| SSHX10 | 101337927 | 101344972 | transcript | SSHX100059093.g |
| SSHX11 | 197055 | 200098 | transcript | SSHX110025363.g |
| SSHX11 | 36019067 | 36026359 | transcript | SSHX110025825.g |
| SSHX11 | 42837424 | 42845750 | transcript | SSHX110026058.g |
| SSHX11 | 44400064 | 44405621 | transcript | SSHX110026101.g |
| SSHX11 | 54132888 | 54155030 | transcript | SSHX110026550.g |
| SSHX11 | 55766823 | 55788844 | transcript | SSHX110026627.g |
| SSHX11 | 55787979 | 55796902 | transcript | SSHX110026630.g |
| SSHX11 | 57531573 | 57536478 | transcript | SSHX110026745.g |
| SSHX11 | 61742561 | 61771048 | transcript | SSHX110027022.g |
| SSHX11 | 70022424 | 70039048 | transcript | SSHX110027506.g |
| SSHX11 | 76053430 | 76064350 | transcript | SSHX110027854.g |
| SSHX11 | 78979800 | 78985916 | transcript | SSHX110027991.g |
| SSHX11 | 81676525 | 81684007 | transcript | SSHX110028137.g |
| SSHX11 | 82744551 | 82749500 | transcript | SSHX110028193.g |
| SSHX11 | 84738470 | 84743706 | transcript | SSHX110028318.g |
| SSHX12 | 30254390 | 30262690 | transcript | SSHX120000549.g |
| SSHX12 | 41395441 | 41399401 | transcript | SSHX120000952.g |
| SSHX12 | 42679246 | 42688688 | transcript | SSHX120001000.g |
| SSHX12 | 44231530 | 44246386 | transcript | SSHX120001050.g |
| SSHX12 | 44563114 | 44571268 | transcript | SSHX120001065.g |
| SSHX12 | 60138401 | 60153019 | transcript | SSHX120001858.g |
| SSHX12 | 63790151 | 63794092 | transcript | SSHX120002064.g |
| SSHX12 | 64872855 | 64884048 | transcript | SSHX120002111.g |
| SSHX12 | 67078574 | 67092677 | transcript | SSHX120002250.g |
| SSHX12 | 76285667 | 76294650 | transcript | SSHX120002832.g |
| SSHX12 | 76424954 | 76428222 | transcript | SSHX120002840.g |
| SSHX12 | 78107578 | 78119483 | transcript | SSHX120002923.g |
| SSHX12 | 79896600 | 79903896 | transcript | SSHX120003052.g |
| SSHX12 | 83496123 | 83501874 | transcript | SSHX120003324.g |
| SSHX13 | 5674962 | 5679847 | transcript | SSHX130018872.g |
| SSHX13 | 8023119 | 8035005 | transcript | SSHX130019032.g |
| SSHX13 | 11673669 | 11683572 | transcript | SSHX130019246.g |
| SSHX13 | 14045569 | 14051498 | transcript | SSHX130019415.g |
| SSHX13 | 21361455 | 21362755 | transcript | SSHX130019811.g |
| SSHX13 | 26256767 | 26265952 | transcript | SSHX130020044.g |
| SSHX13 | 34751015 | 34758869 | transcript | SSHX130020544.g |
| SSHX13 | 37171902 | 37176804 | transcript | SSHX130020616.g |
| SSHX13 | 37851718 | 37853516 | transcript | SSHX130020646.g |
| SSHX13 | 73677541 | 73685323 | transcript | SSHX130021183.g |
| SSHX13 | 80834401 | 80838186 | transcript | SSHX130021351.g |
| SSHX14 | 2434903 | 2446309 | transcript | SSHX140008599.g |
| SSHX14 | 6918135 | 6925456 | transcript | SSHX140008869.g |
| SSHX14 | 9911786 | 9920021 | transcript | SSHX140009056.g |
| SSHX14 | 12360749 | 12362763 | transcript | SSHX140009203.g |
| SSHX14 | 19371759 | 19373268 | transcript | SSHX140009597.g |
| SSHX14 | 28756332 | 28766586 | transcript | SSHX140010246.g |
| SSHX14 | 32098918 | 32106781 | transcript | SSHX140010451.g |
| SSHX14 | 44632916 | 44640848 | transcript | SSHX140011105.g |
| SSHX14 | 70695189 | 70699944 | transcript | SSHX140011663.g |
| SSHX15 | 1797820 | 1799739 | transcript | SSHX150016071.g |
| SSHX15 | 6918294 | 6930873 | transcript | SSHX150016366.g |
| SSHX15 | 17378276 | 17385415 | transcript | SSHX150016780.g |
| SSHX15 | 20278804 | 20281485 | transcript | SSHX150016861.g |
| SSHX15 | 57491235 | 57499230 | transcript | SSHX150017404.g |
| SSHX15 | 70416907 | 70426449 | transcript | SSHX150017873.g |
| SSHX15 | 77769153 | 77781027 | transcript | SSHX150018233.g |
| SSHX15 | 79983373 | 79986277 | transcript | SSHX150018382.g |
| SSHX15 | 80064378 | 80071532 | transcript | SSHX150018387.g |
| SSHX15 | 80510632 | 80523719 | transcript | SSHX150018401.g |
| SSHX16 | 35075914 | 35079062 | transcript | SSHX160060080.g |
| SSHX16 | 41778720 | 41786336 | transcript | SSHX160060205.g |
| SSHX16 | 70714190 | 70726689 | transcript | SSHX160061437.g |
| SSHX16 | 71795387 | 71800533 | transcript | SSHX160061516.g |
| SSHX16 | 74277730 | 74287283 | transcript | SSHX160061657.g |
| SSHX16 | 75392236 | 75402385 | transcript | SSHX160061726.g |
| SSHX16 | 76973624 | 76976350 | transcript | SSHX160061844.g |
| SSHX17 | 823899 | 829958 | transcript | SSHX170041524.g |
| SSHX17 | 5985296 | 5986262 | transcript | SSHX170041870.g |
| SSHX17 | 5985473 | 5985632 | exon | SSHX170041870.g |
| SSHX17 | 5985473 | 5985632 | CDS | SSHX170041870.g |
| SSHX17 | 6064720 | 6067154 | transcript | SSHX170041882.g |
| SSHX17 | 10012825 | 10026598 | transcript | SSHX170042110.g |
| SSHX17 | 10073167 | 10076878 | transcript | SSHX170042114.g |
| SSHX17 | 11290844 | 11295898 | transcript | SSHX170042185.g |
| SSHX17 | 13548628 | 13551536 | transcript | SSHX170042300.g |
| SSHX17 | 14038409 | 14049188 | transcript | SSHX170042333.g |
| SSHX17 | 15598985 | 15616243 | transcript | SSHX170042425.g |
| SSHX17 | 16580753 | 16584594 | transcript | SSHX170042472.g |
| SSHX17 | 29993767 | 30000351 | transcript | SSHX170043240.g |
| SSHX17 | 35371449 | 35381673 | transcript | SSHX170043445.g |
| SSHX17 | 41737436 | 41748181 | transcript | SSHX170043714.g |
| SSHX17 | 46648707 | 46651108 | transcript | SSHX170043873.g |
| SSHX18 | 50695155 | 50702184 | transcript | SSHX180014754.g |
| SSHX18 | 59350265 | 59356949 | transcript | SSHX180015141.g |
| SSHX18 | 60036875 | 60038052 | transcript | SSHX180015186.g |
| SSHX18 | 64672085 | 64677457 | transcript | SSHX180015457.g |
| SSHX18 | 68424586 | 68432652 | transcript | SSHX180015634.g |
| SSHX19 | 42890609 | 42898846 | transcript | SSHX190012766.g |
| SSHX19 | 44623088 | 44629259 | transcript | SSHX190012857.g |
| SSHX19 | 51811760 | 51822589 | transcript | SSHX190013279.g |
| SSHX19 | 52301061 | 52302982 | transcript | SSHX190013300.g |
| SSHX19 | 54692659 | 54696771 | transcript | SSHX190013448.g |
| SSHX19 | 59699127 | 59699732 | transcript | SSHX190013788.g |
| SSHX19 | 59699127 | 59699732 | exon | SSHX190013788.g |
| SSHX19 | 59699127 | 59699732 | CDS | SSHX190013788.g |
| SSHX19 | 62910119 | 62923263 | transcript | SSHX190013977.g |
| SSHX03 | 19442028 | 19448403 | transcript | G2-like |
| SSHX06 | 2318112 | 2320804 | transcript | MYB_related |
| SSHX06 | 6033031 | 6035726 | transcript | BBR-BPC |
| SSHX10 | 87169343 | 87174851 | transcript | WRKY |
| SSHX13 | 2750024 | 2762595 | transcript | C3H |
| SSHX18 | 64673582 | 64673711 | transcript | bHLH |

## Table S12 Differential allelic expression in differential tissue

**A.**

| **Differential allelic expression_flower** | | | |
| --- | --- | --- | --- |
| SSHX010061964.g | 12.60524089 | HHX040026327.g | 64.04384922 |
| SSHX010061979.g | 3.120955889 | HHX060017457.g | 0.377698222 |
| SSHX010061981.g | 0.080545667 | HHX060017458.g | 0.006555444 |
| SSHX010061988.g | 11.04088467 | HHX060017434.g | 0.061309667 |
| SSHX010062000.g | 0.012547444 | HHX060017423.g | 0.071565667 |
| SSHX010062001.g | 0.023465111 | HHX060017476.g | 0.33713 |
| SSHX010062013.g | 323.7740616 | HHX060017490.g | 3.790424333 |
| SSHX010062029.g | 66.24545667 | HHX060017504.g | 2.101320556 |
| SSHX010062034.g | 0.419827 | HHX060017551.g | 0.020048 |
| SSHX010062081.g | 6.943536111 | HHX060017630.g | 0.903232667 |
| SSHX010062084.g | 1.252718778 | HHX060017643.g | 0.058374667 |
| SSHX010062091.g | 0.049522333 | HHX060017650.g | 4.007404556 |
| SSHX010062103.g | 1.119276333 | HHX060017673.g | 9.240260889 |
| SSHX010062111.g | 285.1217196 | HHX060017689.g | 16.86013178 |
| SSHX010062125.g | 34.87715689 | HHX060017701.g | 4.154767333 |
| SSHX010062129.g | 0.741137889 | HHX060017709.g | 4.16226 |
| SSHX010062140.g | 26.27268767 | HHX060017734.g | 0.619591444 |
| SSHX010062143.g | 4.340295333 | HHX060017735.g | 0.273745556 |
| SSHX010062186.g | 7.051102667 | HHX060017783.g | 1.051581222 |
| SSHX010062191.g | 0.021610889 | HHX060017793.g | 0.187536556 |
| SSHX010062233.g | 1.415846556 | HHX060017840.g | 0.155057889 |
| SSHX010062242.g | 0.005059444 | HHX060017851.g | 0.000549444 |
| SSHX010062268.g | 1.411222333 | HHX060017888.g | 0.013937778 |
| SSHX010062315.g | 0.068297222 | HHX060017932.g | 0.343446556 |
| SSHX010062352.g | 3.435611556 | HHX060017951.g | 0.167319667 |
| SSHX010062401.g | 1.350597444 | HHX060017999.g | 0.131354667 |
| SSHX010062422.g | 0.329977 | HHX060018030.g | 0.025749333 |
| SSHX010062442.g | 0.002345556 | HHX060018057.g | 0.029463667 |
| SSHX010062467.g | 0.072561778 | HHX060018087.g | 7.569785111 |
| SSHX010062468.g | 0.881640333 | HHX060018088.g | 0.032389778 |
| SSHX010062475.g | 41.80671011 | HHX060018101.g | 6.376115667 |
| SSHX010062497.g | 8.160080333 | HHX060018120.g | 0.285946 |
| SSHX010062511.g | 4.482004556 | HHX060018134.g | 0.659149667 |
| SSHX010062548.g | 0.022769778 | HHX060018153.g | 0.249327333 |
| SSHX010062572.g | 0.167518556 | HHX060018195.g | 1.018205 |
| SSHX010062575.g | 3.754886667 | HHX060018200.g | 0.122029778 |
| SSHX010062582.g | 0.505152556 | HHX060018208.g | 0.048016889 |
| SSHX010062598.g | 23.24236922 | HHX060018215.g | 2.781008111 |
| SSHX010062599.g | 0.746458778 | HHX060018216.g | 27.65356344 |
| SSHX010062616.g | 0.587006222 | HHX060018238.g | 0.035994 |
| SSHX010062619.g | 0.157391222 | HHX060018243.g | 5.329628111 |
| SSHX010062627.g | 0.018758333 | HHX060018255.g | 0.251704889 |
| SSHX010062644.g | 946.2806734 | HHX060018266.g | 0.428632111 |
| SSHX010062647.g | 0.369249667 | HHX060018267.g | 2.698882667 |
| SSHX010062652.g | 30.32129489 | HHX060018272.g | 2.933420778 |
| SSHX010062655.g | 8.832460333 | HHX060018275.g | 0.068491556 |
| SSHX010062658.g | 0.033377778 | HHX060018280.g | 35.64817178 |
| SSHX010062672.g | 3.236434 | HHX060018299.g | 0.380553111 |
| SSHX010062686.g | 0.107741667 | HHX060018303.g | 1.173169667 |
| SSHX010062710.g | 0.279261556 | HHX060018340.g | 0.012005667 |
| SSHX010062712.g | 1.880765444 | HHX060018315.g | 0.039780111 |
| SSHX010062718.g | 1.244513556 | HHX060018337.g | 0.102089556 |
| SSHX010062723.g | 0.884691556 | HHX060018318.g | 0.110778889 |
| SSHX010062729.g | 0.021212222 | HHX060018329.g | 0.141169444 |
| SSHX010062783.g | 0.310169667 | HHX060018375.g | 65.36666911 |
| SSHX010062796.g | 0.008735444 | HHX060018385.g | 0.052141444 |
| SSHX010062800.g | 0.897536 | HHX060018391.g | 7.215000333 |
| SSHX010062801.g | 2.025257111 | HHX060018394.g | 0.191445889 |
| SSHX010062815.g | 0.138676333 | HHX060018405.g | 7.655790222 |
| SSHX010062821.g | 0.239663 | HHX120001479.g | 4.480533778 |
| SSHX010062823.g | 0.145679889 | HHX120001489.g | 10.73903189 |
| SSHX010062824.g | 0.045499778 | HHX060018416.g | 0.807077333 |
| SSHX010062836.g | 0.072198778 | HHX060018429.g | 0.831456 |
| SSHX010062848.g | 4.676731667 | HHX120001509.g | 0.096487556 |
| SSHX010062870.g | 0.727762 | HHX060018465.g | 4.914432556 |
| SSHX010062871.g | 0.009504111 | HHX060018462.g | 0.207039889 |
| SSHX010062877.g | 8.341994222 | HHX060018462.g | 0.207039889 |
| SSHX010062879.g | 0.019876444 | HHX060018493.g | 63.93898489 |
| SSHX010062880.g | 0.000877444 | HHX060018472.g | 0.940043444 |
| SSHX010062884.g | 0.239511778 | HHX060018470.g | 0.045717556 |
| SSHX010062886.g | 0.011648778 | HHX060018493.g | 63.93898489 |
| SSHX010062887.g | 6.686814111 | HHX060018491.g | 0.494294444 |
| SSHX010062888.g | 1.707375111 | HHX010034307.g | 0.016594778 |
| SSHX010062891.g | 0.004094111 | HHX060018492.g | 0.403265 |
| SSHX010062893.g | 37.71486933 | HHX010034299.g | 0.386862889 |
| SSHX010062894.g | 17.71849956 | HHX010034297.g | 0.195297778 |
| SSHX010062902.g | 341.5402111 | HHX010034282.g | 0.000635778 |
| SSHX010062903.g | 576.1237521 | HHX060018477.g | 0.099573778 |
| SSHX010062976.g | 0.803602667 | HHX060018597.g | 10.77508178 |
| SSHX010062977.g | 1.715863889 | HHX060018571.g | 0.203541222 |
| SSHX010062978.g | 7.058733889 | HHX060018572.g | 0.197865333 |
| SSHX010062983.g | 32.16277511 | HHX060018583.g | 1.449571667 |
| SSHX010062988.g | 0.751486667 | HHX060018594.g | 0.116711778 |
| SSHX010062991.g | 0.667632222 | HHX060018595.g | 0.011986667 |
| SSHX010062992.g | 0.503964778 | HHX060018597.g | 10.77508178 |
| SSHX010063037.g | 0.001079111 | HHX060018963.g | 0.158232556 |
| SSHX010063040.g | 2.852369889 | HHX060018962.g | 0.442743333 |
| SSHX010063045.g | 0.513829 | HHX060018960.g | 17.34086 |
| SSHX010063046.g | 1.656797222 | HHX050044530.g | 0.085850333 |
| SSHX010063047.g | 0.103521667 | HHX050044531.g | 3.689854778 |
| SSHX010063049.g | 0.049260667 | HHX060018957.g | 0.527944333 |
| SSHX010063050.g | 0.109839889 | HHX050044535.g | 5.039793889 |
| SSHX010063105.g | 5.834984778 | HHX060018919.g | 0.488280333 |
| SSHX010063107.g | 0.223091889 | HHX060018914.g | 0.000567222 |
| SSHX010063116.g | 2.105034778 | HHX120001960.g | 62.21539244 |
| SSHX010063128.g | 0.184987889 | HHX060018901.g | 1.776323778 |
| SSHX010063131.g | 0.955861889 | HHX060018894.g | 5.692136889 |
| SSHX010063135.g | 0.350527 | HHX060018887.g | 7.055748556 |
| SSHX010063141.g | 0.288486889 | HHX120001932.g | 14.60829033 |
| SSHX010063142.g | 0.206479 | HHX060018881.g | 6.868666 |
| SSHX010063143.g | 0.165753 | HHX060018880.g | 2.497149556 |
| SSHX010063150.g | 0.264553556 | HHX060018878.g | 5.015114556 |
| SSHX010063160.g | 12.16132056 | HHX060018871.g | 72.969376 |
| SSHX010063161.g | 0.899083778 | HHX060018867.g | 8.665657444 |
| SSHX010063163.g | 76.87237956 | HHX120001900.g | 6.921268667 |
| SSHX010063195.g | 0.989045444 | HHX060018855.g | 191.4565794 |
| SSHX010063198.g | 6.267372444 | HHX060018853.g | 1.146365333 |
| SSHX010063205.g | 0.785608556 | HHX060018848.g | 0.032718667 |
| SSHX010063206.g | 15.978527 | HHX120002028.g | 0.415586 |
| SSHX010063218.g | 32.21341389 | HHX120002037.g | 1080.795139 |
| SSHX010063220.g | 0.064013333 | HHX060018842.g | 4.591279 |
| SSHX010063221.g | 13.980447 | HHX060018841.g | 0.797088667 |
| SSHX010063226.g | 0.430894667 | HHX120002045.g | 8.335012444 |
| SSHX010063228.g | 0.070295 | HHX060018837.g | 0.574083667 |
| SSHX010063230.g | 0.117504222 | HHX060018836.g | 8.816256111 |
| SSHX010063234.g | 2.202994333 | HHX120002062.g | 0.197077667 |
| SSHX010063241.g | 6.241671556 | HHX060018822.g | 0.401352222 |
| SSHX010063248.g | 0.26195 | HHX060018819.g | 2.516578 |
| SSHX010063251.g | 4.664772222 | HHX060018818.g | 0.526423 |
| SSHX010063285.g | 0.004551556 | HHX060018786.g | 0.197786111 |
| SSHX010063290.g | 0.090964222 | HHX060018777.g | 17.51636422 |
| SSHX010063292.g | 0.643426222 | HHX060018775.g | 5.728608667 |
| SSHX010063309.g | 0.044647667 | HHX060018766.g | 0.298510667 |
| SSHX010063312.g | 1.017294667 | HHX120002116.g | 0.057066333 |
| SSHX010063314.g | 6.246673111 | HHX060018760.g | 0.738707889 |
| SSHX010063315.g | 3.833297111 | HHX060018758.g | 0.327105778 |
| SSHX010063317.g | 10.50750022 | HHX060018757.g | 0.170450556 |
| SSHX010063337.g | 43.526301 | HHX120002149.g | 0.430709333 |
| SSHX010063338.g | 0.255761556 | HHX060018744.g | 0.043420333 |
| SSHX010063341.g | 0.242943111 | HHX060018742.g | 2.625705333 |
| SSHX010063364.g | 22.57891456 | HHX060018715.g | 1.099383889 |
| SSHX010063372.g | 72.88710522 | HHX060018711.g | 3.631604111 |
| SSHX010063375.g | 2.905423889 | HHX060018708.g | 0.014108778 |
| SSHX010063382.g | 0.198462333 | HHX060018704.g | 1.002916889 |
| SSHX010063389.g | 7.938246 | HHX060018701.g | 1.054449333 |
| SSHX010063395.g | 2.069626667 | HHX120002182.g | 11.54746667 |
| SSHX010063397.g | 1.133346222 | HHX120002184.g | 18.48602733 |
| SSHX010063417.g | 0.165103333 | HHX120002201.g | 0.001620667 |
| SSHX010063419.g | 0.070233889 | HHX060018678.g | 0.449518778 |
| SSHX010063421.g | 1.746001444 | HHX060018675.g | 153.204172 |
| SSHX010063422.g | 1.324424111 | HHX060018674.g | 9.437901111 |
| SSHX010063423.g | 39.31096089 | HHX060018670.g | 1.814534667 |
| SSHX010063424.g | 107.7512089 | HHX060018669.g | 4.843384 |
| SSHX010063429.g | 0.407497333 | HHX120002213.g | 2.733901444 |
| SSHX010063431.g | 44.93659178 | HHX120002216.g | 0.448258222 |
| SSHX010063432.g | 1.459473 | HHX120002217.g | 0.017228778 |
| SSHX010063433.g | 0.565271222 | HHX120002220.g | 0.086719333 |
| SSHX010063434.g | 7.813292 | HHX060018661.g | 0.112126778 |
| SSHX010063443.g | 30.88812933 | HHX120002238.g | 0.366545111 |
| SSHX010063445.g | 71.17906944 | HHX060018647.g | 9.243683889 |
| SSHX010063454.g | 0.133450667 | HHX060018642.g | 7.330148889 |
| SSHX010063455.g | 0.518406556 | HHX120002242.g | 0.045812889 |
| SSHX010063459.g | 0.712423556 | HHX060018639.g | 0.141355333 |
| SSHX010063460.g | 0.776849222 | HHX060018638.g | 5.931305556 |
| SSHX010063477.g | 0.048299222 | HHX060018622.g | 7.486192444 |
| SSHX010063497.g | 4.925115 | HHX050047488.g | 0.152684222 |
| SSHX010063518.g | 0.113912444 | HHX050047526.g | 2.582772556 |
| SSHX010063529.g | 12.23717311 | HHX050047547.g | 1.971649778 |
| SSHX010063546.g | 4.085419111 | HHX050047534.g | 39.35857256 |
| SSHX010063549.g | 0.014096111 | HHX050047586.g | 0.001724889 |
| SSHX010063550.g | 0.014845 | HHX050047517.g | 0.001893667 |
| SSHX010063564.g | 0.005188667 | HHX050047516.g | 0.582548111 |
| SSHX010063602.g | 14.26869267 | HHX050047637.g | 106.1806513 |
| SSHX010063609.g | 3.099571444 | HHX050047642.g | 1658.447652 |
| SSHX010063646.g | 2.609956889 | HHX050047677.g | 0.308710444 |
| SSHX010063667.g | 0.000713889 | HHX050047695.g | 0.012931 |
| SSHX010063668.g | 0.026408667 | HHX080005526.g | 1.850183667 |
| SSHX010063680.g | 0.605010889 | HHX080005499.g | 5.124235 |
| SSHX010063719.g | 0.921193 | HHX050047738.g | 0.035503667 |
| SSHX010063727.g | 0.052641333 | HHX050047746.g | 1.692255333 |
| SSHX010063730.g | 0.204250889 | HHX050047749.g | 1.639722778 |
| SSHX010063735.g | 0.068518444 | HHX050047756.g | 0.365297667 |
| SSHX010063743.g | 14.63429578 | HHX050047762.g | 1.295625667 |
| SSHX010063752.g | 4.506758222 | HHX050047777.g | 26.71783556 |
| SSHX010063762.g | 0.005803556 | HHX050047807.g | 0.271646111 |
| SSHX010063763.g | 0.651045444 | HHX050047808.g | 8.361639 |
| SSHX010063771.g | 4.995272667 | HHX110051246.g | 318.7475062 |
| SSHX010063781.g | 0.753980222 | HHX050047826.g | 8.387809556 |
| SSHX010063785.g | 0.096819778 | HHX050047831.g | 2.197059778 |
| SSHX010063794.g | 11.23309856 | HHX050047850.g | 0.159400556 |
| SSHX010063795.g | 0.155862111 | HHX110051281.g | 1.649003444 |
| SSHX010063799.g | 0.381632111 | HHX050047854.g | 0.023863111 |
| SSHX010063815.g | 0.462738556 | HHX050047871.g | 0.067476667 |
| SSHX010063820.g | 0.058938444 | HHX050047878.g | 0.002970778 |
| SSHX010063822.g | 28.77099244 | HHX050047887.g | 0.143363667 |
| SSHX010063834.g | 3.264968667 | HHX050047897.g | 0.103486222 |
| SSHX010063841.g | 0.057990889 | HHX050047904.g | 0.629069222 |
| SSHX010063871.g | 4.335618222 | HHX050047953.g | 0.497022556 |
| SSHX010063874.g | 0.068683222 | HHX050047958.g | 0.001309111 |
| SSHX010063892.g | 4.986860667 | HHX050047985.g | 0.006104778 |
| SSHX010063894.g | 0.024824333 | HHX090024386.g | 4.122119889 |
| SSHX010063896.g | 0.058214889 | HHX090024401.g | 0.697509222 |
| SSHX010063932.g | 1.086968556 | HHX050048025.g | 12.46079256 |
| SSHX010063977.g | 0.611439222 | HHX050048060.g | 56.360275 |
| SSHX010063983.g | 0.049192333 | HHX050048066.g | 9.373695111 |
| SSHX010063989.g | 0.519108111 | HHX050048078.g | 2.937271444 |
| SSHX010064005.g | 0.704117444 | HHX050048088.g | 3.783983556 |
| SSHX010064019.g | 1.705904222 | HHX050048116.g | 0.321364556 |
| SSHX010064046.g | 75.38653744 | HHX050048127.g | 2.238577667 |
| SSHX010064052.g | 0.047340444 | HHX050048134.g | 5.657796778 |
| SSHX010064057.g | 4.870771667 | HHX050048140.g | 0.224156 |
| SSHX010064076.g | 0.326653556 | HHX050048155.g | 8.046063333 |
| SSHX010064090.g | 3.731732778 | HHX050048180.g | 0.304748222 |
| SSHX010064105.g | 0.012827556 | HHX050048194.g | 0.001732556 |
| SSHX010064135.g | 0.003764778 | HHX050048216.g | 0.039475444 |
| SSHX010064141.g | 0.131245889 | HHX050048222.g | 3.765015444 |
| SSHX010064147.g | 0.001828222 | HHX050048230.g | 0.126672556 |
| SSHX010064152.g | 1.670379 | HHX050048243.g | 81.99164833 |
| SSHX010064167.g | 0.472726667 | HHX050048260.g | 0.074422778 |
| SSHX010064173.g | 0.000524667 | HHX050048272.g | 4.962948333 |
| SSHX010064223.g | 0.040843 | HHX050048290.g | 2.890505778 |
| SSHX010064241.g | 0.036232333 | HHX050048300.g | 0.713079111 |
| SSHX010064313.g | 0.452435222 | HHX110052257.g | 51.90712144 |
| SSHX010064317.g | 0.237643889 | HHX110052270.g | 3.954352556 |
| SSHX010064319.g | 0.155273333 | HHX050048347.g | 1.082338333 |
| SSHX010064332.g | 0.159011889 | HHX050048351.g | 0.003107333 |
| SSHX010064340.g | 0.013068111 | HHX110052285.g | 0.540944111 |
| SSHX010064346.g | 0.004255556 | HHX050048371.g | 0.205242556 |
| SSHX010064359.g | 0.010945667 | HHX110052288.g | 6.877570778 |
| SSHX010064381.g | 0.058572 | HHX050048392.g | 0.556214667 |
| SSHX010064387.g | 4.319864444 | HHX050048395.g | 0.343005222 |
| SSHX010064392.g | 23.701961 | HHX050048398.g | 0.418670444 |
| SSHX010064402.g | 0.028821889 | HHX050048405.g | 0.160217778 |
| SSHX010064406.g | 0.855125556 | HHX050048406.g | 7.624175 |
| SSHX010064436.g | 0.439909889 | HHX050048438.g | 0.017629556 |
| SSHX010064438.g | 1.278034889 | HHX050048440.g | 0.097383111 |
| SSHX010064443.g | 2.659752 | HHX050048442.g | 0.236443444 |
| SSHX010064444.g | 1.213794556 | HHX050048443.g | 0.023966222 |
| SSHX010064459.g | 1.289117556 | HHX050048451.g | 0.147012778 |
| SSHX010064475.g | 0.008622667 | HHX050048465.g | 0.142482111 |
| SSHX010064480.g | 0.027095444 | HHX050048468.g | 0.549095556 |
| SSHX010064486.g | 0.804787222 | HHX050048470.g | 0.032006333 |
| SSHX010064529.g | 0.190996111 | HHX010035534.g | 0.000488444 |
| SSHX010064530.g | 17.78134767 | HHX010035538.g | 0.085883889 |
| SSHX010064532.g | 1.383042444 | HHX010035550.g | 0.006926778 |
| SSHX010064534.g | 2.6028 | HHX010035548.g | 0.057898111 |
| SSHX010064535.g | 0.105520778 | HHX010035541.g | 0.008920667 |
| SSHX010064542.g | 4.647836556 | HHX010035550.g | 0.006926778 |
| SSHX010064556.g | 4.730575444 | HHX050048522.g | 38.05784633 |
| SSHX010064559.g | 39.16083344 | HHX050048526.g | 6.134277333 |
| SSHX010064570.g | 28.23127789 | HHX110052429.g | 302.4030559 |
| SSHX010064574.g | 0.071180889 | HHX050048538.g | 0.013077889 |
| SSHX010064583.g | 0.032047667 | HHX110052461.g | 0.212753667 |
| SSHX010064586.g | 0.008516889 | HHX110052462.g | 2.228634778 |
| SSHX010064587.g | 0.015106 | HHX110052463.g | 0.180706333 |
| SSHX010064592.g | 0.145632222 | HHX110052468.g | 18.36684822 |
| SSHX010064593.g | 13.60356389 | HHX050048555.g | 2.367173667 |
| SSHX010064596.g | 0.022552444 | HHX050048557.g | 0.166033333 |
| SSHX010064598.g | 1.522432444 | HHX110052475.g | 51.65855989 |
| SSHX010064599.g | 0.131971333 | HHX050048558.g | 1.555019778 |
| SSHX010064603.g | 0.006007 | HHX050048566.g | 0.153571667 |
| SSHX010064606.g | 10.23363689 | HHX050048571.g | 179.5804647 |
| SSHX010064621.g | 0.327933889 | HHX050048587.g | 0.049797111 |
| SSHX010064624.g | 1.931760111 | HHX050048589.g | 25.45833911 |
| SSHX010064635.g | 0.263398889 | HHX050048601.g | 0.042476667 |
| SSHX010064665.g | 1.618121889 | HHX110052565.g | 0.226621889 |
| SSHX010064680.g | 0.134958444 | HHX050048615.g | 1.698793444 |
| SSHX010064685.g | 0.003919556 | HHX110052613.g | 1.941117667 |
| SSHX010064700.g | 27.75556156 | HHX050048647.g | 0.368001111 |
| SSHX010064705.g | 0.018350222 | HHX110052639.g | 0.098363778 |
| SSHX010064707.g | 4.842568333 | HHX110052654.g | 0.810783556 |
| SSHX010064712.g | 4.761604333 | HHX050048667.g | 0.381355778 |
| SSHX010064721.g | 0.215231222 | HHX050048671.g | 1.432139667 |
| SSHX010064722.g | 23.58596011 | HHX050048672.g | 2.205490444 |
| SSHX010064741.g | 8.127990667 | HHX050048849.g | 0.519992556 |
| SSHX010064744.g | 10.56952989 | HHX050048855.g | 0.250596 |
| SSHX010064745.g | 1.425593778 | HHX050048857.g | 17.33279378 |
| SSHX010064748.g | 0.865524556 | HHX050048883.g | 0.076794444 |
| SSHX010064752.g | 4.503613556 | HHX110052691.g | 25.12861411 |
| SSHX010064770.g | 0.257300222 | HHX110052692.g | 1.429672556 |
| SSHX010064772.g | 56.42405856 | HHX050048884.g | 0.333296778 |
| SSHX010064774.g | 125.5762618 | HHX050048885.g | 23.71337344 |
| SSHX010064776.g | 97.85115833 | HHX050048886.g | 3.050257778 |
| SSHX010064778.g | 0.008141 | HHX050048770.g | 8.830866333 |
| SSHX010064782.g | 0.065513778 | HHX050048891.g | 0.004285222 |
| SSHX010064792.g | 0.022852333 | HHX110052696.g | 0.485648556 |
| SSHX010064795.g | 0.125474778 | HHX050048726.g | 26.37522733 |
| SSHX010064796.g | 0.134177889 | HHX050048911.g | 0.016361889 |
| SSHX010064798.g | 55.45398178 | HHX050048915.g | 0.245576556 |
| SSHX010064799.g | 9.266119 | HHX050048686.g | 0.336006667 |
| SSHX010064800.g | 16.28185056 | HHX050048688.g | 0.005329778 |
| SSHX010064801.g | 0.353931333 | HHX050048746.g | 5.443518556 |
| SSHX010064806.g | 0.203206889 | HHX050048726.g | 26.37522733 |
| SSHX010064807.g | 0.597482222 | HHX050048745.g | 6.420230333 |
| SSHX010064808.g | 0.066089 | HHX050048926.g | 25.99300233 |
| SSHX010064810.g | 0.017207667 | HHX050048704.g | 2.064891889 |
| SSHX010064812.g | 0.326895889 | HHX050048914.g | 1.653496778 |
| SSHX010064813.g | 0.000933778 | HHX050048694.g | 0.147337778 |
| SSHX010064819.g | 8.123691778 | HHX050048907.g | 0.758573222 |
| SSHX010064827.g | 12.62848944 | HHX050048905.g | 2.181067 |
| SSHX010064829.g | 2.426737778 | HHX050048903.g | 0.286309556 |
| SSHX010064837.g | 0.207115333 | HHX050048896.g | 1.423333444 |
| SSHX010064840.g | 6.531843889 | HHX050048698.g | 0.751726333 |
| SSHX010064842.g | 0.400464333 | HHX050048700.g | 3.996386111 |
| SSHX010064848.g | 0.009578 | HHX050048701.g | 0.090988667 |
| SSHX010064849.g | 6.825551444 | HHX050048929.g | 0.476447222 |
| SSHX010064850.g | 2.327752444 | HHX050048684.g | 0.152504556 |
| SSHX010064853.g | 0.064041 | HHX050048679.g | 0.369601778 |
| SSHX010064854.g | 0.083419778 | HHX050048693.g | 8.604899667 |
| SSHX010064855.g | 0.058311111 | HHX050048704.g | 2.064891889 |
| SSHX010064856.g | 1.701136333 | HHX050048764.g | 0.153049444 |
| SSHX010064857.g | 0.026423333 | HHX050048687.g | 1.206604778 |
| SSHX010064858.g | 0.220747 | HHX050048705.g | 2.325711556 |
| SSHX010064859.g | 0.079585444 | HHX050048756.g | 2.122222222 |
| SSHX010064864.g | 0.043166444 | HHX050048693.g | 8.604899667 |
| SSHX010064866.g | 0.111576667 | HHX050048722.g | 0.935615556 |
| SSHX010064876.g | 6.792093444 | HHX050048711.g | 0.090297222 |
| SSHX010064878.g | 31.68021167 | HHX050048919.g | 0.096986444 |
| SSHX010064879.g | 45.359568 | HHX050048704.g | 2.064891889 |
| SSHX010064888.g | 22.360174 | HHX050048711.g | 0.090297222 |
| SSHX010064898.g | 9.848029111 | HHX050048763.g | 0.272699778 |
| SSHX010064899.g | 0.044135667 | HHX050048778.g | 1.642447 |
| SSHX010064905.g | 0.309341111 | HHX050048757.g | 0.038284222 |
| SSHX010064907.g | 0.548932222 | HHX050048762.g | 6.764158 |
| SSHX010064908.g | 0.454140778 | HHX050048793.g | 0.045086 |
| SSHX010064910.g | 1.686701111 | HHX050048714.g | 0.171199444 |
| SSHX010064911.g | 0.007856333 | HHX050048687.g | 1.206604778 |
| SSHX010064913.g | 8.411341778 | HHX050048889.g | 0.027321222 |
| SSHX010064916.g | 0.731439667 | HHX050048772.g | 0.067507111 |
| SSHX010064918.g | 0.208498778 | HHX050048773.g | 6.305416111 |
| SSHX010064919.g | 4.740459556 | HHX050048793.g | 0.045086 |
| SSHX010064920.g | 2.200352444 | HHX050048757.g | 0.038284222 |
| SSHX010064926.g | 0.013557111 | HHX050048763.g | 0.272699778 |
| SSHX010064929.g | 0.020945778 | HHX050048764.g | 0.153049444 |
| SSHX010064931.g | 0.399448444 | HHX110052729.g | 30.81037189 |
| SSHX010064945.g | 0.319125 | HHX050048810.g | 33.87719933 |
| SSHX010064947.g | 0.010797111 | HHX050048817.g | 0.668821444 |
| SSHX010064975.g | 4.996054 | HHX110052807.g | 0.484501111 |
| SSHX010064999.g | 0.723243 | HHX050048945.g | 0.084405444 |
| SSHX010065001.g | 0.094885222 | HHX050048948.g | 30.90001933 |
| SSHX010065012.g | 8.388024222 | HHX050048963.g | 0.689089444 |
| SSHX010065019.g | 0.088431 | HHX050048971.g | 0.486954889 |
| SSHX010065020.g | 0.510798778 | HHX050048972.g | 4.098571778 |
| SSHX010065024.g | 0.162109333 | HHX050048977.g | 5.648051556 |
| SSHX010065028.g | 0.780776667 | HHX050048980.g | 0.146711 |
| SSHX010065034.g | 0.001174778 | HHX050048989.g | 0.010091111 |
| SSHX010065042.g | 0.094862 | HHX050048997.g | 3.686813333 |
| SSHX010065051.g | 2.189831111 | HHX110052886.g | 0.116116111 |
| SSHX010065061.g | 0.167162111 | HHX050049008.g | 1.283807444 |
| SSHX010065095.g | 3.970835889 | HHX050049035.g | 0.790018111 |
| SSHX010065096.g | 18.71484978 | HHX050049036.g | 1.578253778 |
| SSHX010065103.g | 17.58671022 | HHX050049037.g | 0.717056889 |
| SSHX010065124.g | 0.000364333 | HHX030014440.g | 0.318002444 |
| SSHX010065126.g | 0.000197444 | HHX030014411.g | 0.188714222 |
| SSHX010065127.g | 0.000122889 | HHX030014410.g | 0.018612222 |
| SSHX010065128.g | 0.002438333 | HHX030014419.g | 1.572310333 |
| SSHX010065136.g | 0.061140111 | HHX030014422.g | 1.55175 |
| SSHX010065137.g | 0.045099111 | HHX050049067.g | 0.006421889 |
| SSHX010065144.g | 0.293853 | HHX050049064.g | 0.002809111 |
| SSHX010065155.g | 0.005277 | HHX030014422.g | 1.55175 |
| SSHX010065156.g | 0.013601889 | HHX010036809.g | 0.255443222 |
| SSHX010065158.g | 0.003361667 | HHX030014410.g | 0.018612222 |
| SSHX010065160.g | 0.017278333 | HHX030014471.g | 3.478594111 |
| SSHX010065161.g | 0.299919444 | HHX010036818.g | 0.003328667 |
| SSHX010065165.g | 7.617867667 | HHX050049097.g | 0.550370333 |
| SSHX010065166.g | 0.501144667 | HHX050049098.g | 16.26082322 |
| SSHX010065167.g | 0.147963778 | HHX050049099.g | 1.193510444 |
| SSHX010065186.g | 1.409169111 | HHX050049114.g | 12.64159111 |
| SSHX010065190.g | 0.042092333 | HHX050049118.g | 0.258681222 |
| SSHX010065203.g | 6.260782667 | HHX050049130.g | 0.335474111 |
| SSHX010065207.g | 0.005911889 | HHX050049136.g | 0.120677222 |
| SSHX010065238.g | 1.774322778 | HHX050049139.g | 0.273650889 |
| SSHX010065239.g | 0.209019778 | HHX050049140.g | 1.123541889 |
| SSHX010065242.g | 36.54323733 | HHX050049142.g | 2.871068111 |
| SSHX010065248.g | 0.051677444 | HHX050049146.g | 0.343770222 |
| SSHX010065253.g | 0.053841 | HHX050049152.g | 0.004769556 |
| SSHX010065259.g | 1.515884222 | HHX050049156.g | 0.264082 |
| SSHX010065301.g | 0.079820222 | HHX050049194.g | 0.449595333 |
| SSHX010065308.g | 0.581986222 | HHX050049196.g | 4.479992778 |
| SSHX010065398.g | 7.395380667 | HHX050046465.g | 0.067343222 |
| SSHX010065401.g | 0.204377111 | HHX050046466.g | 4.358782333 |
| SSHX010065410.g | 0.082123556 | HHX050046472.g | 0.872597111 |
| SSHX010065411.g | 2.534182778 | HHX050046473.g | 0.049781333 |
| SSHX010065418.g | 0.775480222 | HHX050046476.g | 0.135170667 |
| SSHX010065421.g | 43.76516156 | HHX050046481.g | 1.508331778 |
| SSHX010065437.g | 0.609305444 | HHX050046493.g | 0.054954667 |
| SSHX010065439.g | 0.002642444 | HHX050046496.g | 0.044488333 |
| SSHX010065449.g | 0.398660778 | HHX050046504.g | 0.038990556 |
| SSHX010065453.g | 0.656017222 | HHX050046509.g | 0.123327667 |
| SSHX010065456.g | 1.082812222 | HHX050046513.g | 0.132989444 |
| SSHX010065465.g | 0.045028667 | HHX050046516.g | 0.696282111 |
| SSHX010065468.g | 0.780409444 | HHX100023259.g | 109.2909987 |
| SSHX010065469.g | 0.012344222 | HHX050046522.g | 0.467923111 |
| SSHX010065473.g | 0.388702556 | HHX100023245.g | 0.012542111 |
| SSHX010065474.g | 6.306203222 | HHX100023223.g | 0.121447667 |
| SSHX010065484.g | 2.347679 | HHX100023251.g | 0.019458778 |
| SSHX010065485.g | 1.417932 | HHX100023211.g | 0.010413444 |
| SSHX010065491.g | 1.739821889 | HHX050046540.g | 0.054645444 |
| SSHX010065498.g | 17.44524422 | HHX050046535.g | 2.553833111 |
| SSHX010065503.g | 0.997797556 | HHX050046595.g | 21.78590044 |
| SSHX010065509.g | 0.017809222 | HHX050046589.g | 0.311968778 |
| SSHX010065510.g | 3.967157111 | HHX050046588.g | 0.168951556 |
| SSHX010065535.g | 79.96315833 | HHX050046563.g | 0.602875889 |
| SSHX010065551.g | 3.244942889 | HHX050046612.g | 0.265588778 |
| SSHX010065554.g | 1.391590778 | HHX050046615.g | 0.146482444 |
| SSHX010065564.g | 4.565483333 | HHX050046622.g | 0.623998667 |
| SSHX010065579.g | 0.042530444 | HHX050046637.g | 0.359314778 |
| SSHX010065581.g | 2.349583889 | HHX050046638.g | 0.367634111 |
| SSHX010065582.g | 0.633616667 | HHX050046639.g | 9.260522 |
| SSHX010065591.g | 0.045002556 | HHX050046639.g | 9.260522 |
| SSHX010065613.g | 0.701293889 | HHX050046647.g | 0.046154889 |
| SSHX010065616.g | 0.197587444 | HHX050046755.g | 1.516247778 |
| SSHX010065618.g | 0.001777556 | HHX050046738.g | 3.363565333 |
| SSHX010065619.g | 0.043736778 | HHX050046743.g | 0.308717333 |
| SSHX010065620.g | 0.340056444 | HHX050046762.g | 5.971221222 |
| SSHX010065626.g | 0.036715111 | HHX050046677.g | 1.182347111 |
| SSHX010065638.g | 0.744631 | HHX050046651.g | 0.124285556 |
| SSHX010065639.g | 0.445528667 | HHX050046641.g | 0.060713556 |
| SSHX010065641.g | 1.856171 | HHX050046652.g | 0.295918333 |
| SSHX010065647.g | 0.066900667 | HHX050046655.g | 0.786668444 |
| SSHX010065652.g | 0.234267333 | HHX050046648.g | 0.029561333 |
| SSHX010065655.g | 0.995568556 | HHX050046641.g | 0.060713556 |
| SSHX010065659.g | 0.097149667 | HHX050046671.g | 2.477153556 |
| SSHX010065664.g | 0.254224222 | HHX050046647.g | 0.046154889 |
| SSHX010065680.g | 0.946837667 | HHX050046651.g | 0.124285556 |
| SSHX010065684.g | 0.212230889 | HHX050046648.g | 0.029561333 |
| SSHX010065700.g | 0.058448222 | HHX050046639.g | 9.260522 |
| SSHX010065702.g | 0.465372333 | HHX050046671.g | 2.477153556 |
| SSHX010065710.g | 0.723850111 | HHX080002845.g | 0.077947444 |
| SSHX010065715.g | 0.231812889 | HHX050046730.g | 0.023337667 |
| SSHX010065736.g | 0.014585889 | HHX050046677.g | 1.182347111 |
| SSHX010065741.g | 14.00750522 | HHX050046695.g | 0.085405556 |
| SSHX010065742.g | 1.198977333 | HHX050046715.g | 0.173661 |
| SSHX010065744.g | 5.616194667 | HHX010034275.g | 0.010501889 |
| SSHX010065752.g | 0.804220778 | HHX050046695.g | 0.085405556 |
| SSHX010065753.g | 0.954423333 | HHX050046715.g | 0.173661 |
| SSHX010065756.g | 0.098953333 | HHX010034288.g | 0.006315333 |
| SSHX010065757.g | 0.000785667 | HHX010034274.g | 0.005076444 |
| SSHX010065758.g | 0.076848222 | HHX010034275.g | 0.010501889 |
| SSHX010065766.g | 0.219507111 | HHX050046755.g | 1.516247778 |
| SSHX010065768.g | 0.328327556 | HHX050046739.g | 0.038450889 |
| SSHX010065770.g | 1.435704111 | HHX050046722.g | 0.047993111 |
| SSHX010065771.g | 1.218882556 | HHX010034288.g | 0.006315333 |
| SSHX010065773.g | 12.25324944 | HHX050046686.g | 0.627624556 |
| SSHX010065780.g | 11.04129911 | HHX050046755.g | 1.516247778 |
| SSHX010065782.g | 0.078652778 | HHX010034288.g | 0.006315333 |
| SSHX010065784.g | 1.673823444 | HHX050046766.g | 0.201002111 |
| SSHX010065786.g | 1.197824444 | HHX050046769.g | 0.109820778 |
| SSHX010065787.g | 0.942018556 | HHX050046722.g | 0.047993111 |
| SSHX010065789.g | 2.671501111 | HHX050046729.g | 0.170780556 |
| SSHX010065790.g | 3.726710889 | HHX050046730.g | 0.023337667 |
| SSHX010065791.g | 1.067183333 | HHX050046760.g | 0.207684222 |
| SSHX010065792.g | 0.146408778 | HHX010034307.g | 0.016594778 |
| SSHX010065793.g | 0.045262667 | HHX010034282.g | 0.000635778 |
| SSHX010065794.g | 0.01471 | HHX050046761.g | 1.378575333 |
| SSHX010065795.g | 0.329971333 | HHX010034275.g | 0.010501889 |
| SSHX010065797.g | 1.369424222 | HHX050046760.g | 0.207684222 |
| SSHX010065803.g | 21.091756 | HHX050046770.g | 0.313888111 |
| SSHX010065813.g | 0.012461 | HHX050046779.g | 0.354391222 |
| SSHX010065831.g | 2.236507889 | HHX050046799.g | 15.39398011 |
| SSHX010065832.g | 0.024343444 | HHX050046802.g | 0.254003889 |
| SSHX010065840.g | 0.014237111 | HHX050046811.g | 1.236784889 |
| SSHX010065854.g | 0.034190111 | HHX050046816.g | 0.364346667 |
| SSHX010065884.g | 25.78797656 | HHX050046846.g | 1.316581667 |
| SSHX010065888.g | 19.72712478 | HHX050046848.g | 0.064409 |
| SSHX010065891.g | 3.821381333 | HHX050046852.g | 0.080894 |
| SSHX010065896.g | 1.344204556 | HHX050046858.g | 113.7870687 |
| SSHX010065897.g | 0.005956111 | HHX050046860.g | 0.044375889 |
| SSHX010065898.g | 0.002458556 | HHX050046861.g | 0.027592222 |
| SSHX010065915.g | 11.98861989 | HHX050046874.g | 2.388831222 |
| SSHX010065925.g | 0.162961667 | HHX050046879.g | 0.025160111 |
| SSHX010065946.g | 0.336622556 | HHX050046893.g | 6.751267444 |
| SSHX010065947.g | 10.17722544 | HHX050046895.g | 1.716323778 |
| SSHX010065948.g | 16.11512789 | HHX050046896.g | 1.377752222 |
| SSHX010065952.g | 4.768646556 | HHX050046899.g | 0.332807556 |
| SSHX010065957.g | 21.32986944 | HHX050046901.g | 0.027991111 |
| SSHX010065967.g | 41.734373 | HHX050046917.g | 2.962154667 |
| SSHX010065972.g | 0.090906333 | HHX050046921.g | 0.735667111 |
| SSHX010066001.g | 4.154789222 | HHX050046943.g | 87.54093456 |
| SSHX010066002.g | 21.44845178 | HHX050046944.g | 0.261001111 |
| SSHX010066006.g | 8.282105778 | HHX050046948.g | 43.50798811 |
| SSHX010066009.g | 0.839518889 | HHX050046953.g | 6.261224222 |
| SSHX010066034.g | 0.031791889 | HHX050046963.g | 0.589298444 |
| SSHX010066040.g | 0.003559667 | HHX050046970.g | 0.024351222 |
| SSHX010066057.g | 0.309740333 | HHX050046981.g | 3.135206889 |
| SSHX010066065.g | 0.073114778 | HHX040027016.g | 0.486414333 |
| SSHX010066066.g | 0.020748667 | HHX040027028.g | 2.135278556 |
| SSHX010066078.g | 0.125005556 | HHX050046995.g | 1.392492778 |
| SSHX010066104.g | 0.134515667 | HHX050047021.g | 1.319947889 |
| SSHX010066107.g | 3.132509222 | HHX050047025.g | 0.021349444 |
| SSHX010066127.g | 0.068123111 | HHX050047033.g | 3.486721222 |
| SSHX010066129.g | 0.007662 | HHX040027069.g | 0.488640667 |
| SSHX010066130.g | 0.005506667 | HHX050047034.g | 3.423674556 |
| SSHX010066137.g | 4.912447556 | HHX050047054.g | 0.056001444 |
| SSHX010066143.g | 0.021332444 | HHX050047076.g | 4.247308556 |
| SSHX010066153.g | 177.5324301 | HHX050047076.g | 4.247308556 |
| SSHX010066154.g | 97.21806511 | HHX050047064.g | 1.123554889 |
| SSHX010066155.g | 152.8489126 | HHX050047069.g | 8.693778 |
| SSHX010066157.g | 1.205297778 | HHX050047079.g | 12.33968011 |
| SSHX010066158.g | 0.540415556 | HHX050047060.g | 0.052324889 |
| SSHX010066169.g | 0.473891778 | HHX050047054.g | 0.056001444 |
| SSHX010066170.g | 0.568249889 | HHX050047077.g | 18.62217611 |
| SSHX010066171.g | 0.006049889 | HHX050047079.g | 12.33968011 |
| SSHX010066186.g | 0.520399111 | HHX050047079.g | 12.33968011 |
| SSHX010066187.g | 0.01759 | HHX040027008.g | 9.804280333 |
| SSHX010066202.g | 0.332672667 | HHX050047147.g | 7.151237667 |
| SSHX010066207.g | 0.174661889 | HHX050047165.g | 8.593138 |
| SSHX010066216.g | 0.052998444 | HHX050047131.g | 23.46458044 |
| SSHX010066217.g | 3.779138 | HHX050047146.g | 0.665955667 |
| SSHX010066224.g | 1.648017111 | HHX050047182.g | 0.011376111 |
| SSHX010066225.g | 6.213985222 | HHX050047540.g | 0.730122778 |
| SSHX010066226.g | 0.046281667 | HHX050047541.g | 6.178934111 |
| SSHX010066228.g | 0.075082778 | HHX050047099.g | 1.636735111 |
| SSHX010066239.g | 6.836722889 | HHX050046986.g | 0.200698444 |
| SSHX010066242.g | 0.042823333 | HHX050047148.g | 1.621954667 |
| SSHX010066244.g | 0.014075444 | HHX050047136.g | 0.073639444 |
| SSHX010066252.g | 0.110209889 | HHX050047182.g | 0.011376111 |
| SSHX010066253.g | 0.277977111 | HHX050047161.g | 0.026182333 |
| SSHX010066256.g | 0.023553889 | HHX050046978.g | 0.942262778 |
| SSHX010066258.g | 0.040064111 | HHX050047125.g | 0.205261 |
| SSHX010066266.g | 68.78751389 | HHX050047165.g | 8.593138 |
| SSHX010066268.g | 2.202698 | HHX050047168.g | 15.10706711 |
| SSHX010066269.g | 0.001204444 | HHX050047138.g | 0.467657 |
| SSHX010066270.g | 0.026455333 | HHX050047567.g | 3.876624778 |
| SSHX010066278.g | 0.387682 | HHX050047176.g | 3.875679778 |
| SSHX010066279.g | 0.242916444 | HHX050047148.g | 1.621954667 |
| SSHX010066286.g | 0.015558778 | HHX040027106.g | 2.982741778 |
| SSHX010066288.g | 0.620961 | HHX050047182.g | 0.011376111 |
| SSHX010066289.g | 0.856915667 | HHX050047183.g | 0.120294444 |
| SSHX010066304.g | 1.152472889 | HHX050047196.g | 0.186730222 |
| SSHX010066338.g | 0.539124 | HHX050047230.g | 0.086524222 |
| SSHX010066344.g | 0.079399 | HHX040027028.g | 2.135278556 |
| SSHX010066356.g | 0.028673889 | HHX050047279.g | 0.003024556 |
| SSHX010066395.g | 0.941332 | HHX050047314.g | 9.671613778 |
| SSHX010066444.g | 3.933832333 | HHX150061805.g | 43.45179511 |
| SSHX010066445.g | 12.79039033 | HHX150061803.g | 1.033207 |
| SSHX010066467.g | 1.760406667 | HHX150061764.g | 10.66056978 |
| SSHX010066481.g | 1.588227333 | HHX150061752.g | 15.78437678 |
| SSHX010066482.g | 0.722926333 | HHX150061764.g | 10.66056978 |
| SSHX010066507.g | 5.643933444 | HHX150061735.g | 0.282284 |
| SSHX010066513.g | 0.006211 | HHX150061703.g | 0.126423444 |
| SSHX010066537.g | 2.289354333 | HHX150061672.g | 0.299943667 |
| SSHX010066571.g | 0.823579444 | HHX150061643.g | 16.64339144 |
| SSHX010066580.g | 0.014224333 | HHX150061633.g | 0.084988778 |
| SSHX010066598.g | 7.037642 | HHX150061609.g | 43.60559489 |
| SSHX010066615.g | 0.219354 | HHX150061593.g | 79.190316 |
| SSHX010066667.g | 0.045255333 | HHX150061536.g | 0.731221667 |
| SSHX010066684.g | 0.028726778 | HHX150061502.g | 0.250712556 |
| SSHX010066750.g | 0.234883111 | HHX150061449.g | 1.976729889 |
| SSHX010066757.g | 0.083011889 | HHX150061437.g | 8.123062889 |
| SSHX010066782.g | 0.692356556 | HHX150061396.g | 0.006263 |
| SSHX010066803.g | 0.060036111 | HHX150061376.g | 0.003393333 |
| SSHX010066807.g | 0.022727889 | HHX150061369.g | 0.453067889 |
| SSHX010066815.g | 0.168230444 | HHX150061352.g | 0.010366556 |
| SSHX010066819.g | 0.007402667 | HHX150061343.g | 0.037411 |
| SSHX010066833.g | 7.017265444 | HHX150061328.g | 1.086248778 |
| SSHX010066836.g | 0.861924889 | HHX150061324.g | 33.765038 |
| SSHX010066846.g | 0.604396667 | HHX150061313.g | 7.340059556 |
| SSHX010066847.g | 0.038966889 | HHX150061312.g | 1.270760111 |
| SSHX010066854.g | 1.928758667 | HHX150061294.g | 19.41020789 |
| SSHX010066867.g | 3.775009333 | HHX150061231.g | 0.233102111 |
| SSHX010066869.g | 0.015679444 | HHX150061228.g | 0.083639111 |
| SSHX010066875.g | 0.828960778 | HHX150061225.g | 0.077732222 |
| SSHX010066890.g | 43.48975378 | HHX150061210.g | 2.551434111 |
| SSHX010066894.g | 0.010665333 | HHX150061215.g | 0.130542222 |
| SSHX010066914.g | 24.28316533 | HHX150061227.g | 0.048618111 |
| SSHX010066915.g | 0.017378222 | HHX150061299.g | 0.131003667 |
| SSHX010066923.g | 0.019626111 | HHX150061308.g | 2.124699111 |
| SSHX010066952.g | 1.515783778 | HHX150061269.g | 0.276919778 |
| SSHX010066976.g | 2.666857556 | HHX150061188.g | 38.71251722 |
| SSHX010066983.g | 5.923015667 | HHX150061183.g | 0.476031667 |
| SSHX010066986.g | 2.291399444 | HHX150061179.g | 16.64035011 |
| SSHX010066996.g | 11.80992489 | HHX150061167.g | 1.282162556 |
| SSHX010067004.g | 11.30926944 | HHX070057511.g | 1.753312778 |
| SSHX010067006.g | 0.578990889 | HHX070057506.g | 3.710586556 |
| SSHX010067011.g | 5.018663889 | HHX070057495.g | 0.137147111 |
| SSHX010067021.g | 67.70422556 | HHX070057492.g | 0.835577556 |
| SSHX010067033.g | 42.80759156 | HHX040031662.g | 1.832446444 |
| SSHX010067037.g | 1.009335 | HHX040031664.g | 0.165048889 |
| SSHX010067042.g | 0.139328111 | HHX040031665.g | 2.896871111 |
| SSHX010067044.g | 63.85062422 | HHX040031669.g | 9.908245222 |
| SSHX010067045.g | 5.853647111 | HHX040031672.g | 0.078068667 |
| SSHX010067051.g | 2.093365111 | HHX040031680.g | 0.288513889 |
| SSHX010067056.g | 6.249270333 | HHX070057477.g | 104.2850162 |
| SSHX010067063.g | 102.5385918 | HHX040031685.g | 7.325465889 |
| SSHX010067082.g | 6.858008444 | HHX040031690.g | 0.156505 |
| SSHX010067096.g | 0.160822222 | HHX040031707.g | 15.734442 |
| SSHX010067097.g | 0.679658222 | HHX040031708.g | 0.023928556 |
| SSHX010067119.g | 0.088311333 | HHX040031715.g | 0.538248222 |
| SSHX010067127.g | 6.183911333 | HHX070057451.g | 2373.538425 |
| SSHX010067128.g | 807.0610758 | HHX070057449.g | 10.95715611 |
| SSHX010067129.g | 874.2004396 | HHX070057447.g | 4741.218045 |
| SSHX010067140.g | 0.116765667 | HHX040031643.g | 1.154409778 |
| SSHX010067149.g | 0.704887667 | HHX040031636.g | 0.003316667 |
| SSHX010067150.g | 444.4748839 | HHX070057429.g | 30.69466933 |
| SSHX010067155.g | 0.289661444 | HHX040031634.g | 1.635153444 |
| SSHX010067157.g | 0.005332778 | HHX070057350.g | 0.073201 |
| SSHX010067161.g | 0.211882222 | HHX070057341.g | 2.110921222 |
| SSHX010067169.g | 0.579916111 | HHX040031613.g | 14.54565856 |
| SSHX010067186.g | 10.00037344 | HHX040031611.g | 0.513264222 |
| SSHX010067214.g | 0.062849667 | HHX040031590.g | 0.911292444 |
| SSHX010067237.g | 8.204959222 | HHX040031574.g | 0.200939778 |
| SSHX010067245.g | 7.721954667 | HHX040031569.g | 0.970747222 |
| SSHX010067252.g | 2.81347 | HHX040031562.g | 30.10040633 |
| SSHX010067254.g | 2.538488111 | HHX040031558.g | 31.96877867 |
| SSHX010067263.g | 2.755876556 | HHX040031550.g | 15.15698656 |
| SSHX010067265.g | 1.820374444 | HHX040031543.g | 0.054469333 |
| SSHX010067270.g | 0.010927778 | HHX040031541.g | 0.136721 |
| SSHX010067272.g | 0.086548667 | HHX040031539.g | 0.611669556 |
| SSHX010067276.g | 7.558326222 | HHX040031535.g | 0.550210889 |
| SSHX010067279.g | 0.070402667 | HHX070057289.g | 0.472853667 |
| SSHX010067306.g | 0.148758556 | HHX040031516.g | 0.015977444 |
| SSHX010067319.g | 9.344786778 | HHX040031497.g | 0.457023889 |
| SSHX010067332.g | 12.426578 | HHX040031485.g | 1.544177111 |
| SSHX010067337.g | 0.005999778 | HHX040031481.g | 0.068818333 |
| SSHX010067342.g | 3.166212889 | HHX070057177.g | 0.030733778 |
| SSHX010067343.g | 5.254460778 | HHX070057183.g | 0.033659667 |
| SSHX010067344.g | 6.766619778 | HHX070057184.g | 0.017632444 |
| SSHX010067345.g | 8.449023778 | HHX070057197.g | 0.312065 |
| SSHX010067355.g | 0.012666889 | HHX040031463.g | 0.071694889 |
| SSHX010067357.g | 2.403766778 | HHX070057196.g | 0.162229111 |
| SSHX010067360.g | 1.572948 | HHX070057213.g | 0.104620111 |
| SSHX010067363.g | 0.200479111 | HHX070057183.g | 0.033659667 |
| SSHX010067367.g | 0.008302333 | HHX040031441.g | 0.072791667 |
| SSHX010067371.g | 1.479691333 | HHX040031444.g | 0.149174889 |
| SSHX010067373.g | 1.041515667 | HHX040031451.g | 0.034989444 |
| SSHX010067402.g | 0.048206889 | HHX070057102.g | 3.897890889 |
| SSHX010067406.g | 0.363498444 | HHX040031423.g | 6.354342778 |
| SSHX010067407.g | 54.76504511 | HHX040031422.g | 3.985626889 |
| SSHX010067410.g | 0.076240667 | HHX070057098.g | 1.650798556 |
| SSHX010067411.g | 1.144146667 | HHX040031419.g | 0.019107667 |
| SSHX010067412.g | 27.41810433 | HHX040031417.g | 1.774878333 |
| SSHX010067416.g | 5.506674444 | HHX070057097.g | 39.24960378 |
| SSHX010067422.g | 3.938226556 | HHX040031410.g | 0.321044444 |
| SSHX010067428.g | 0.029164778 | HHX070057094.g | 0.339651778 |
| SSHX010067429.g | 34.56333033 | HHX040031400.g | 3.412111667 |
| SSHX010067430.g | 0.063048444 | HHX040031399.g | 0.531759778 |
| SSHX010067446.g | 2.006416556 | HHX040031392.g | 0.071029667 |
| SSHX010067456.g | 0.813316889 | HHX040031386.g | 29.03620256 |
| SSHX010067465.g | 0.234649222 | HHX040031369.g | 5.172880889 |
| SSHX010067481.g | 3.380083889 | HHX040031357.g | 0.205156444 |
| SSHX010067487.g | 2.063143889 | HHX040031365.g | 0.220805111 |
| SSHX010067516.g | 0.003814 | HHX010034299.g | 0.386862889 |
| SSHX010067518.g | 0.241727667 | HHX010034275.g | 0.010501889 |
| SSHX010067525.g | 1.558414111 | HHX040031331.g | 0.304008 |
| SSHX010067546.g | 0.052746778 | HHX040031313.g | 0.003680889 |
| SSHX010067547.g | 0.025915556 | HHX070057059.g | 1.122389444 |
| SSHX010067559.g | 3.459977556 | HHX040031306.g | 0.207217444 |
| SSHX010067560.g | 54.31926356 | HHX070057048.g | 0.54261 |
| SSHX010067561.g | 37.480121 | HHX040031190.g | 0.24831 |
| SSHX010067562.g | 0.044115444 | HHX040031202.g | 0.004603222 |
| SSHX010067563.g | 0.301146111 | HHX040031198.g | 0.045511222 |
| SSHX010067564.g | 4.672172556 | HHX040029263.g | 0.022609778 |
| SSHX010067569.g | 0.093587889 | HHX040031286.g | 4.371737111 |
| SSHX010067571.g | 0.006162333 | HHX040031204.g | 0.042438333 |
| SSHX010067576.g | 3.017242889 | HHX040031253.g | 0.004594111 |
| SSHX010067579.g | 0.107217667 | HHX040031202.g | 0.004603222 |
| SSHX010067580.g | 26.25989689 | HHX040031260.g | 0.027087889 |
| SSHX010067584.g | 2.441684667 | HHX070057030.g | 0.173712889 |
| SSHX010067594.g | 21.139753 | HHX040031190.g | 0.24831 |
| SSHX010067597.g | 0.139237333 | HHX040031229.g | 0.006374 |
| SSHX010067602.g | 0.012470556 | HHX040029221.g | 0.171858333 |
| SSHX010067603.g | 0.210122111 | HHX040031213.g | 3.448033333 |
| SSHX010067607.g | 2.047297667 | HHX040031198.g | 0.045511222 |
| SSHX010067613.g | 5.683689111 | HHX040031202.g | 0.004603222 |
| SSHX010067614.g | 31.78685 | HHX040031213.g | 3.448033333 |
| SSHX010067616.g | 21.74316233 | HHX040031260.g | 0.027087889 |
| SSHX010067619.g | 0.631726444 | HHX040031198.g | 0.045511222 |
| SSHX010067633.g | 1.306443556 | HHX040031229.g | 0.006374 |
| SSHX010067638.g | 0.007746778 | HHX040031212.g | 0.116079444 |
| SSHX010067639.g | 0.145985222 | HHX040031260.g | 0.027087889 |
| SSHX010067645.g | 0.198761222 | HHX040031213.g | 3.448033333 |
| SSHX010067647.g | 0.044046556 | HHX040029233.g | 9.047353778 |
| SSHX010067648.g | 0.280676556 | HHX040031256.g | 4.73489 |
| SSHX010067655.g | 0.007518 | HHX040031241.g | 0.272187778 |
| SSHX010067656.g | 0.044276556 | HHX040031303.g | 447.3840518 |
| SSHX010067657.g | 0.027073889 | HHX040031222.g | 0.410679111 |
| SSHX010067658.g | 0.992055333 | HHX040031262.g | 0.179545778 |
| SSHX010067659.g | 1.596662889 | HHX040029235.g | 0.302651667 |
| SSHX010067660.g | 0.223415 | HHX040031202.g | 0.004603222 |
| SSHX010067661.g | 0.284294111 | HHX040031252.g | 0.049371778 |
| SSHX010067663.g | 0.013688778 | HHX040031205.g | 0.744348111 |
| SSHX010067665.g | 23.49952222 | HHX070057026.g | 0.743648222 |
| SSHX010067672.g | 62.16500333 | HHX040029241.g | 1.203712111 |
| SSHX010067674.g | 3.860914889 | HHX040031234.g | 0.119503556 |
| SSHX010067675.g | 0.028572222 | HHX040031256.g | 4.73489 |
| SSHX010067677.g | 0.562996889 | HHX040031204.g | 0.042438333 |
| SSHX010067678.g | 1.454303333 | HHX040031260.g | 0.027087889 |
| SSHX010067679.g | 0.04511 | HHX040031202.g | 0.004603222 |
| SSHX010067680.g | 0.115348333 | HHX040031288.g | 2.995597333 |
| SSHX010067681.g | 0.051025111 | HHX040029232.g | 13.54128067 |
| SSHX010067685.g | 0.059369556 | HHX040031221.g | 2.137383222 |
| SSHX010067686.g | 0.473081222 | HHX040031252.g | 0.049371778 |
| SSHX010067687.g | 1.316925444 | HHX040031260.g | 0.027087889 |
| SSHX010067689.g | 0.042427889 | HHX040031202.g | 0.004603222 |
| SSHX010067692.g | 0.000855889 | HHX040031211.g | 0.136766222 |
| SSHX010067696.g | 4.495134111 | HHX040031222.g | 0.410679111 |
| SSHX010067697.g | 45.66870578 | HHX040031270.g | 3.292391889 |
| SSHX010067700.g | 0.273020556 | HHX040031221.g | 2.137383222 |
| SSHX010067701.g | 0.179891333 | HHX040029261.g | 15.86564678 |
| SSHX010067702.g | 0.319039333 | HHX040031189.g | 26.34213311 |
| SSHX010067704.g | 28.68705367 | HHX070057012.g | 1.829555889 |
| SSHX010067706.g | 410.7827747 | HHX040031185.g | 17.84379311 |
| SSHX010067710.g | 1.932175444 | HHX070057011.g | 0.360477 |
| SSHX010067712.g | 1.405133667 | HHX040031180.g | 22.68877611 |
| SSHX010067714.g | 0.170092889 | HHX070056999.g | 0.000479222 |
| SSHX010067728.g | 0.462618444 | HHX040031175.g | 0.067136111 |
| SSHX010067729.g | 0.158861667 | HHX040031174.g | 27.10152822 |
| SSHX010067730.g | 62.22809811 | HHX040031172.g | 9.192609222 |
| SSHX010067740.g | 13.62739122 | HHX040031170.g | 1.969299333 |
| SSHX010067774.g | 32.45476711 | HHX100021748.g | 1.629408667 |
| SSHX010067775.g | 1.880129444 | HHX040031097.g | 0.021203444 |
| SSHX010067776.g | 9.076342667 | HHX040031126.g | 0.001416333 |
| SSHX010067785.g | 0.171969222 | HHX040031021.g | 5.005290333 |
| SSHX010067788.g | 0.097054889 | HHX040031080.g | 0.017038 |
| SSHX010067792.g | 0.001821333 | HHX040031111.g | 0.020019222 |
| SSHX010067794.g | 7.199953333 | HHX040031039.g | 0.827563778 |
| SSHX010067799.g | 0.847833 | HHX040031041.g | 0.105424 |
| SSHX010067801.g | 0.041837333 | HHX040031099.g | 0.002223111 |
| SSHX010067817.g | 0.092813667 | HHX040031045.g | 0.737562 |
| SSHX010067830.g | 0.397961444 | HHX040031049.g | 0.036693222 |
| SSHX010067834.g | 7.287956778 | HHX040030718.g | 1.209319111 |
| SSHX010067836.g | 4.856978556 | HHX040031077.g | 0.683273111 |
| SSHX010067837.g | 9.208305667 | HHX040031059.g | 0.945366667 |
| SSHX010067838.g | 8.260021333 | HHX040031046.g | 1.198657778 |
| SSHX010067839.g | 0.168238222 | HHX040031080.g | 0.017038 |
| SSHX010067842.g | 4.265979667 | HHX040030793.g | 0.445444222 |
| SSHX010067843.g | 0.749492889 | HHX040030807.g | 0.008075 |
| SSHX010067845.g | 0.032848333 | HHX040030594.g | 0.00084 |
| SSHX010067846.g | 0.003598889 | HHX040030747.g | 3.821131444 |
| SSHX010067854.g | 0.044336556 | HHX040030807.g | 0.008075 |
| SSHX010067859.g | 3.661234111 | HHX040030755.g | 0.148911 |
| SSHX010067872.g | 1.018326889 | HHX040031041.g | 0.105424 |
| SSHX010067875.g | 0.105951889 | HHX040030807.g | 0.008075 |
| SSHX010067877.g | 1.149001778 | HHX040030635.g | 0.038355778 |
| SSHX010067878.g | 0.015474444 | HHX040030765.g | 0.289341556 |
| SSHX010067879.g | 0.011252778 | HHX040030594.g | 0.00084 |
| SSHX010067886.g | 0.605632444 | HHX040030604.g | 0.020033444 |
| SSHX010067887.g | 0.107317778 | HHX040030807.g | 0.008075 |
| SSHX010067890.g | 0.032117556 | HHX040030830.g | 0.534535778 |
| SSHX010067896.g | 6.223114222 | HHX040030835.g | 1.132385333 |
| SSHX010067903.g | 0.027852556 | HHX040030838.g | 0.500701222 |
| SSHX010067910.g | 0.026897889 | HHX040030870.g | 1.407634222 |
| SSHX010067941.g | 0.074798889 | HHX040030878.g | 0.441002111 |
| SSHX010067953.g | 0.053351444 | HHX040030921.g | 0.363498222 |
| SSHX010067955.g | 0.005544111 | HHX040030923.g | 0.040261889 |
| SSHX010067957.g | 0.213582222 | HHX040030925.g | 1.186682556 |
| SSHX010067965.g | 0.036139222 | HHX040031056.g | 1.083197 |
| SSHX010067967.g | 0.258447222 | HHX040031032.g | 3.657746556 |
| SSHX010067969.g | 2.790264556 | HHX040030906.g | 0.108321111 |
| SSHX010067976.g | 0.011531889 | HHX040031059.g | 0.945366667 |
| SSHX010067977.g | 0.360495667 | HHX040031049.g | 0.036693222 |
| SSHX010067983.g | 0.007554 | HHX040031079.g | 0.233487444 |
| SSHX010067984.g | 0.039095111 | HHX040031050.g | 0.955703444 |
| SSHX010067988.g | 0.013173667 | HHX040031071.g | 0.090388667 |
| SSHX010067989.g | 4.377708889 | HHX040031114.g | 0.623473556 |
| SSHX010067990.g | 3.058404556 | HHX040031029.g | 0.309030889 |
| SSHX010068006.g | 0.152330667 | HHX040031003.g | 0.023171778 |
| SSHX010068007.g | 1.900560778 | HHX040031080.g | 0.017038 |
| SSHX010068012.g | 0.100195 | HHX040031050.g | 0.955703444 |
| SSHX010068019.g | 0.046901444 | HHX040031093.g | 0.33231 |
| SSHX010068029.g | 13.63607211 | HHX040030967.g | 2.715401889 |
| SSHX010068034.g | 206.0458041 | HHX070056845.g | 1.363125 |
| SSHX010068035.g | 101.8606778 | HHX070056844.g | 0.966130222 |
| SSHX010068038.g | 3.196855111 | HHX040030958.g | 47.58446311 |
| SSHX010068039.g | 2.871064889 | HHX040030959.g | 0.105045778 |
| SSHX010068040.g | 24.21220711 | HHX040030957.g | 162.4545867 |
| SSHX010068048.g | 0.196536 | HHX070056840.g | 8.009537667 |
| SSHX010068056.g | 7.867366 | HHX040030944.g | 0.524913333 |
| SSHX010068057.g | 3.906003333 | HHX040030943.g | 354.5454069 |
| SSHX010068064.g | 0.021076778 | HHX040030925.g | 1.186682556 |
| SSHX010068076.g | 2.624183222 | HHX070056823.g | 0.013295222 |
| SSHX010068078.g | 0.953164778 | HHX040030920.g | 0.046063333 |
| SSHX010068080.g | 0.129516 | HHX040030636.g | 0.911191111 |
| SSHX010068082.g | 0.120918444 | HHX040030907.g | 0.754106111 |
| SSHX010068088.g | 0.396010111 | HHX040030919.g | 2.339697889 |
| SSHX010068093.g | 0.404480222 | HHX040030920.g | 0.046063333 |
| SSHX010068094.g | 0.022105889 | HHX040030905.g | 0.473130444 |
| SSHX010068096.g | 0.05986 | HHX040030718.g | 1.209319111 |
| SSHX010068102.g | 0.016772333 | HHX040030799.g | 3.903657778 |
| SSHX010068103.g | 0.312907333 | HHX040030614.g | 0.031193889 |
| SSHX010068111.g | 1.144114889 | HHX070057904.g | 0.010829444 |
| SSHX010068115.g | 0.207826778 | HHX040030847.g | 1.944484222 |
| SSHX010068118.g | 0.275267778 | HHX040030864.g | 0.013073333 |
| SSHX010068121.g | 0.701766222 | HHX040030780.g | 0.010975778 |
| SSHX010068125.g | 0.081135444 | HHX040030851.g | 0.677055 |
| SSHX010068127.g | 0.637191667 | HHX040030785.g | 0.093301667 |
| SSHX010068129.g | 1.005831889 | HHX040030843.g | 54.55944544 |
| SSHX010068132.g | 2.907241556 | HHX040030864.g | 0.013073333 |
| SSHX010068134.g | 0.033382111 | HHX040030849.g | 0.622900889 |
| SSHX010068140.g | 0.009678889 | HHX070057871.g | 3.758653444 |
| SSHX010068141.g | 3.413608222 | HHX040030863.g | 25.33433711 |
| SSHX010068157.g | 8.576158667 | HHX040030828.g | 0.514228556 |
| SSHX010068159.g | 0.505105889 | HHX040030807.g | 0.008075 |
| SSHX010068170.g | 8.684524222 | HHX040030588.g | 1.055043333 |
| SSHX010068171.g | 2.731262 | HHX040030716.g | 0.131628111 |
| SSHX010068175.g | 0.011767111 | HHX040030732.g | 2.884868 |
| SSHX010068176.g | 0.004372333 | HHX040030738.g | 0.107276 |
| SSHX010068177.g | 9.499489556 | HHX040030723.g | 1.162816111 |
| SSHX010068189.g | 0.047266 | HHX040030736.g | 1.089887333 |
| SSHX010068202.g | 0.004899111 | HHX040030747.g | 3.821131444 |
| SSHX010068203.g | 0.085594111 | HHX040030780.g | 0.010975778 |
| SSHX010068210.g | 6.832666556 | HHX040030700.g | 1.268425111 |
| SSHX010068214.g | 0.599223667 | HHX040030807.g | 0.008075 |
| SSHX010068219.g | 0.959735111 | HHX040030614.g | 0.031193889 |
| SSHX010068221.g | 0.050052 | HHX150062246.g | 0.002550444 |
| SSHX010068224.g | 0.010820444 | HHX040030753.g | 0.065006889 |
| SSHX010068228.g | 2.812521556 | HHX040030826.g | 0.370058222 |
| SSHX010068229.g | 0.030179222 | HHX040030594.g | 0.00084 |
| SSHX010068233.g | 4.764544222 | HHX040030785.g | 0.093301667 |
| SSHX010068234.g | 6.430598778 | HHX040030691.g | 1.279803889 |
| SSHX010068235.g | 1.686102778 | HHX040030807.g | 0.008075 |
| SSHX010068240.g | 0.015390556 | HHX040030799.g | 3.903657778 |
| SSHX010068247.g | 0.622042778 | HHX040030811.g | 0.073995 |
| SSHX010068252.g | 0.149148333 | HHX040030807.g | 0.008075 |
| SSHX010068254.g | 0.110059 | HHX040030724.g | 1.030139222 |
| SSHX010068259.g | 2.960910444 | HHX040030614.g | 0.031193889 |
| SSHX010068263.g | 0.665402778 | HHX040031022.g | 6.552543111 |
| SSHX010068265.g | 0.048036222 | HHX040030736.g | 1.089887333 |
| SSHX010068269.g | 0.006264222 | HHX040030765.g | 0.289341556 |
| SSHX010068271.g | 0.692855111 | HHX040030750.g | 0.083870667 |
| SSHX010068273.g | 0.086081778 | HHX040031050.g | 0.955703444 |
| SSHX010068280.g | 2.730945667 | HHX040030785.g | 0.093301667 |
| SSHX010068285.g | 2.761687667 | HHX040030682.g | 59.809135 |
| SSHX010068287.g | 0.028503667 | HHX030014391.g | 0.001187556 |
| SSHX010068288.g | 0.069423333 | HHX030014397.g | 0.011059889 |
| SSHX010068294.g | 0.022371667 | HHX030014412.g | 0.661658556 |
| SSHX010068298.g | 0.002849556 | HHX030014420.g | 2.915506222 |
| SSHX010068299.g | 0.040784111 | HHX030014422.g | 1.55175 |
| SSHX010068301.g | 8.392023444 | HHX040030666.g | 0.095861778 |
| SSHX010068305.g | 35.03314444 | HHX040030652.g | 0.228545 |
| SSHX010068311.g | 0.016406 | HHX040030607.g | 0.530190444 |
| SSHX010068317.g | 0.022685 | HHX040030633.g | 0.001063111 |
| SSHX010068338.g | 0.016563778 | HHX040030732.g | 2.884868 |
| SSHX010068341.g | 0.875889222 | HHX040030600.g | 7.690451111 |
| SSHX010068348.g | 0.718683333 | HHX040030632.g | 0.036611 |
| SSHX010068350.g | 5.661975556 | HHX040030586.g | 0.805170556 |
| SSHX010068351.g | 0.152081778 | HHX040030585.g | 5.884709333 |
| SSHX010068356.g | 0.060255222 | HHX040030747.g | 3.821131444 |
| SSHX010068357.g | 3.863559333 | HHX040030699.g | 0.098147 |
| SSHX010068361.g | 0.068909333 | HHX150062246.g | 0.002550444 |
| SSHX010068364.g | 0.042530556 | HHX040030799.g | 3.903657778 |
| SSHX010068365.g | 0.089381444 | HHX040030779.g | 0.521478111 |
| SSHX010068370.g | 0.384565556 | HHX040030593.g | 0.060679444 |
| SSHX010068371.g | 0.161135333 | HHX040030732.g | 2.884868 |
| SSHX010068372.g | 0.002141667 | HHX040030738.g | 0.107276 |
| SSHX010068375.g | 0.112702444 | HHX040030715.g | 0.772901556 |
| SSHX010068376.g | 0.118209111 | HHX040030792.g | 15.353892 |
| SSHX010068378.g | 2.161250556 | HHX040031049.g | 0.036693222 |
| SSHX010068381.g | 0.039863778 | HHX040030717.g | 2.683966778 |
| SSHX010068382.g | 0.014327889 | HHX040030792.g | 15.353892 |
| SSHX010068383.g | 0.003367444 | HHX040030738.g | 0.107276 |
| SSHX010068385.g | 3.315837111 | HHX040030716.g | 0.131628111 |
| SSHX010068386.g | 2.738115667 | HHX040030604.g | 0.020033444 |
| SSHX010068387.g | 0.070948778 | HHX040030807.g | 0.008075 |
| SSHX010068389.g | 1.783425222 | HHX040030713.g | 0.327380667 |
| SSHX010068390.g | 0.090042222 | HHX040030626.g | 0.009306222 |
| SSHX010068391.g | 0.223773778 | HHX040030964.g | 3.019349 |
| SSHX020044365.g | 16.75403033 | HHX080003401.g | 2.497188111 |
| SSHX020044372.g | 1.877597778 | HHX080003405.g | 25.42563044 |
| SSHX020044397.g | 3.383425444 | HHX080003429.g | 30.86083667 |
| SSHX020044402.g | 0.083960444 | HHX080003436.g | 1.267961778 |
| SSHX020044412.g | 0.189257556 | HHX080003455.g | 1.505937444 |
| SSHX020044424.g | 5.021436333 | HHX080003461.g | 0.111052889 |
| SSHX020044430.g | 3.327140333 | HHX080003466.g | 0.162199667 |
| SSHX020044478.g | 12.26063011 | HHX080003527.g | 0.009558111 |
| SSHX020044497.g | 3.049640778 | HHX080003577.g | 0.567042556 |
| SSHX020044503.g | 1.006434333 | HHX080003587.g | 37.38478344 |
| SSHX020044528.g | 11.08453978 | HHX080003605.g | 0.197497333 |
| SSHX020044544.g | 0.040878556 | HHX080003630.g | 0.706059 |
| SSHX020044606.g | 5.682659333 | HHX080003697.g | 0.068971111 |
| SSHX020044616.g | 0.573926111 | HHX080003707.g | 0.078695 |
| SSHX020044628.g | 2.399368 | HHX080003728.g | 13.28799756 |
| SSHX020044630.g | 1.429120889 | HHX080003730.g | 30.81393156 |
| SSHX020044632.g | 10.85974411 | HHX080003732.g | 1.002926889 |
| SSHX020044643.g | 23.97648478 | HHX080003740.g | 2.067790889 |
| SSHX020044664.g | 0.214665333 | HHX080003756.g | 4.296920889 |
| SSHX020044764.g | 3.363702667 | HHX080003831.g | 0.002308333 |
| SSHX020044772.g | 44.41610122 | HHX080003833.g | 0.03883 |
| SSHX020044775.g | 0.294151556 | HHX080003825.g | 0.027847667 |
| SSHX020044798.g | 2.080380889 | HHX080003873.g | 0.243238778 |
| SSHX020044804.g | 0.052036778 | HHX080003863.g | 16.63720244 |
| SSHX020044805.g | 1.003234333 | HHX040029221.g | 0.171858333 |
| SSHX020044808.g | 6.298343111 | HHX040029225.g | 0.501090222 |
| SSHX020044809.g | 2.148326889 | HHX040029262.g | 0.186330778 |
| SSHX020044813.g | 9.191600889 | HHX080003825.g | 0.027847667 |
| SSHX020044814.g | 4.490446222 | HHX080003891.g | 0.304661889 |
| SSHX020044815.g | 0.050322889 | HHX040029251.g | 19.20023922 |
| SSHX020044827.g | 24.62806811 | HHX080003890.g | 1.495643667 |
| SSHX020044830.g | 4.886529667 | HHX040029235.g | 0.302651667 |
| SSHX020044831.g | 5.454079 | HHX040029262.g | 0.186330778 |
| SSHX020044832.g | 20.66827356 | HHX080003874.g | 2.141192333 |
| SSHX020044833.g | 15.28974411 | HHX040029223.g | 0.000233111 |
| SSHX020044835.g | 44.89101333 | HHX080003901.g | 3.049613111 |
| SSHX020044841.g | 0.048511111 | HHX080003904.g | 23.79394389 |
| SSHX020044842.g | 101.1416223 | HHX080003910.g | 1.898717889 |
| SSHX020044843.g | 4.903625333 | HHX080003911.g | 0.600215889 |
| SSHX020044856.g | 4.034430667 | HHX080003965.g | 0.172741444 |
| SSHX020044867.g | 0.069710111 | HHX080003976.g | 1.767579556 |
| SSHX020044868.g | 0.074673222 | HHX080003978.g | 1.534542556 |
| SSHX020044892.g | 0.052748333 | HHX080004008.g | 0.527507111 |
| SSHX020044896.g | 38.992512 | HHX080004022.g | 0.725874778 |
| SSHX020044907.g | 0.037042778 | HHX080004032.g | 0.357723111 |
| SSHX020044913.g | 0.084275667 | HHX080004040.g | 0.472753111 |
| SSHX020044924.g | 0.025056778 | HHX080004067.g | 1.301414778 |
| SSHX020044934.g | 0.039347444 | HHX080004040.g | 0.472753111 |
| SSHX020044935.g | 17.49285378 | HHX080004067.g | 1.301414778 |
| SSHX020044936.g | 8.847329111 | HHX080004054.g | 1.004798333 |
| SSHX020044940.g | 0.156809222 | HHX080004074.g | 1.228670111 |
| SSHX020044948.g | 12.79110756 | HHX080004091.g | 0.455462333 |
| SSHX020044975.g | 0.023487667 | HHX080004098.g | 1.329198444 |
| SSHX020044982.g | 22.16715678 | HHX080004104.g | 0.307949667 |
| SSHX020045012.g | 29.71186433 | HHX080004114.g | 2.274623889 |
| SSHX020045025.g | 0.702236889 | HHX080004134.g | 0.02188 |
| SSHX020045038.g | 0.623597444 | HHX080004142.g | 26.24668533 |
| SSHX020045054.g | 0.782349222 | HHX080004159.g | 0.131704778 |
| SSHX020045089.g | 1.329251889 | HHX080004182.g | 0.080788111 |
| SSHX020045092.g | 0.017477667 | HHX080004183.g | 0.132155778 |
| SSHX020045094.g | 0.028841667 | HHX080004185.g | 0.667604 |
| SSHX020045143.g | 1.505757 | HHX080004242.g | 297.1262309 |
| SSHX020045157.g | 0.764480333 | HHX080004279.g | 0.019550889 |
| SSHX020045224.g | 6.056974222 | HHX080004348.g | 0.399739778 |
| SSHX020045237.g | 2.642258667 | HHX080004351.g | 19.39586967 |
| SSHX020045258.g | 2.148367889 | HHX080004377.g | 17.11183622 |
| SSHX020045273.g | 0.012072444 | HHX080004377.g | 17.11183622 |
| SSHX020045287.g | 41.37501944 | HHX080004392.g | 3.121571222 |
| SSHX020045288.g | 0.634832556 | HHX080004393.g | 4.369823111 |
| SSHX020045289.g | 0.232549 | HHX080004394.g | 1.304069667 |
| SSHX020045301.g | 0.141661111 | HHX080004411.g | 10.19245444 |
| SSHX020045336.g | 98.23697244 | HHX080004450.g | 0.023383667 |
| SSHX020045337.g | 119.1244762 | HHX080004466.g | 0.057612556 |
| SSHX020045342.g | 0.394204778 | HHX080004467.g | 0.043663778 |
| SSHX020045346.g | 1.244777111 | HHX080004578.g | 23.28804267 |
| SSHX020045347.g | 0.008803111 | HHX080004504.g | 0.000123778 |
| SSHX020045349.g | 2.629063778 | HHX080004475.g | 0.035705778 |
| SSHX020045351.g | 9.546763889 | HHX080004450.g | 0.023383667 |
| SSHX020045355.g | 0.306529333 | HHX080004561.g | 3.015937111 |
| SSHX020045356.g | 1.313936778 | HHX080004519.g | 28.28450811 |
| SSHX020045362.g | 3.273994556 | HHX080004474.g | 0.007391333 |
| SSHX020045363.g | 0.630662 | HHX080004450.g | 0.023383667 |
| SSHX020045364.g | 0.481147222 | HHX080004588.g | 6.235435222 |
| SSHX020045365.g | 1.446553 | HHX080004557.g | 0.056245778 |
| SSHX020045367.g | 0.020595778 | HHX080004562.g | 2.439793 |
| SSHX020045368.g | 29.45305178 | HHX080004515.g | 1.731384889 |
| SSHX020045371.g | 0.958020778 | HHX080004593.g | 0.003327778 |
| SSHX020045374.g | 0.207522778 | HHX080004580.g | 1.366767 |
| SSHX020045378.g | 0.0071 | HHX080004475.g | 0.035705778 |
| SSHX020045382.g | 0.443354556 | HHX080004512.g | 0.004799222 |
| SSHX020045390.g | 0.001174667 | HHX080004552.g | 1.501330778 |
| SSHX020045395.g | 0.572874778 | HHX080004450.g | 0.023383667 |
| SSHX020045396.g | 0.001236556 | HHX080004491.g | 0.153854111 |
| SSHX020045408.g | 0.006239 | HHX080004504.g | 0.000123778 |
| SSHX020045410.g | 0.00428 | HHX080004507.g | 0.371689444 |
| SSHX020045425.g | 0.009141778 | HHX080004579.g | 35.56153567 |
| SSHX020045426.g | 2.632265222 | HHX080004548.g | 0.484118222 |
| SSHX020045427.g | 1.409984 | HHX080004523.g | 0.000522556 |
| SSHX020045428.g | 33.90484589 | HHX080004515.g | 1.731384889 |
| SSHX020045430.g | 0.405022556 | HHX080004551.g | 0.039010444 |
| SSHX020045431.g | 2.095490111 | HHX080004590.g | 0.035264222 |
| SSHX020045432.g | 0.152652556 | HHX080004593.g | 0.003327778 |
| SSHX020045433.g | 0.180998 | HHX080004563.g | 1.113838667 |
| SSHX020045436.g | 1.027550333 | HHX080004466.g | 0.057612556 |
| SSHX020045437.g | 0.933668111 | HHX080004522.g | 9.430864 |
| SSHX020045454.g | 0.131857333 | HHX080004474.g | 0.007391333 |
| SSHX020045455.g | 0.564368222 | HHX080004450.g | 0.023383667 |
| SSHX020045457.g | 3.619649111 | HHX080004578.g | 23.28804267 |
| SSHX020045458.g | 6.132055222 | HHX080004548.g | 0.484118222 |
| SSHX020045460.g | 0.019339444 | HHX080004532.g | 0.158089556 |
| SSHX020045464.g | 0.310173556 | HHX080004520.g | 2.106202667 |
| SSHX020045467.g | 0.006146667 | HHX080004563.g | 1.113838667 |
| SSHX020045473.g | 0.001526444 | HHX080004567.g | 1.606261 |
| SSHX020045474.g | 0.056685111 | HHX080004579.g | 35.56153567 |
| SSHX020045481.g | 0.217999889 | HHX080004458.g | 1.149398556 |
| SSHX020045489.g | 0.009982222 | HHX080004584.g | 1.390570333 |
| SSHX020045503.g | 76.92520411 | HHX080004559.g | 7.70201 |
| SSHX020045521.g | 0.019342111 | HHX080004598.g | 0.380387333 |
| SSHX020045550.g | 8.454250889 | HHX080004660.g | 0.417706778 |
| SSHX020045563.g | 0.191314556 | HHX080004672.g | 1.294398778 |
| SSHX020045564.g | 13.41066844 | HHX120002366.g | 79.64294589 |
| SSHX020045565.g | 2.226145111 | HHX120002369.g | 14.36125222 |
| SSHX020045576.g | 0.497729 | HHX080004678.g | 2.488763333 |
| SSHX020045597.g | 0.389289111 | HHX080004704.g | 0.016590667 |
| SSHX020045600.g | 0.289957444 | HHX080004712.g | 0.033996444 |
| SSHX020045601.g | 0.182092778 | HHX080004713.g | 0.033396333 |
| SSHX020045605.g | 1.428774667 | HHX080004718.g | 0.186698222 |
| SSHX020045610.g | 0.331359111 | HHX080004724.g | 0.016031778 |
| SSHX020045612.g | 0.026774444 | HHX080004726.g | 0.664929556 |
| SSHX020045632.g | 22.00090867 | HHX080004732.g | 0.782398 |
| SSHX020045638.g | 0.115818 | HHX120002420.g | 1.298907889 |
| SSHX020045661.g | 2.670816556 | HHX080004749.g | 0.282023667 |
| SSHX020045686.g | 0.165064111 | HHX080004771.g | 2.899543111 |
| SSHX020045689.g | 1.744220111 | HHX080004774.g | 13.42643189 |
| SSHX020045691.g | 48.24259589 | HHX080004779.g | 4.556575556 |
| SSHX020045701.g | 0.023595444 | HHX080004781.g | 0.368850444 |
| SSHX020045705.g | 13.14927389 | HHX080004783.g | 0.514473778 |
| SSHX020045728.g | 38.82541689 | HHX080004803.g | 7.611734556 |
| SSHX020045740.g | 5.500286889 | HHX080004812.g | 30.66849267 |
| SSHX020045745.g | 14.34211622 | HHX080004819.g | 2.809290444 |
| SSHX020045751.g | 16.98162933 | HHX080004826.g | 2.834102889 |
| SSHX020045755.g | 24.97274156 | HHX080004830.g | 2.779927444 |
| SSHX020045759.g | 0.346781111 | HHX080004835.g | 2.012631222 |
| SSHX020045791.g | 0.029251778 | HHX080004858.g | 0.153278889 |
| SSHX020045794.g | 4.143070111 | HHX080004862.g | 0.510956444 |
| SSHX020045797.g | 0.475441444 | HHX080004865.g | 2.446152889 |
| SSHX020045798.g | 5.328754333 | HHX080004866.g | 0.064534 |
| SSHX020045838.g | 144.8351092 | HHX080004887.g | 0.010987222 |
| SSHX020045846.g | 0.693066333 | HHX190053946.g | 0.005925111 |
| SSHX020045861.g | 0.258051667 | HHX080004912.g | 1.380087889 |
| SSHX020045878.g | 10.56422489 | HHX190053926.g | 1.700860889 |
| SSHX020045881.g | 6.165714111 | HHX080004929.g | 0.151912444 |
| SSHX020045883.g | 0.008408778 | HHX080004933.g | 0.049721444 |
| SSHX020045902.g | 1.366603778 | HHX190053915.g | 99.07330111 |
| SSHX020045904.g | 0.038848111 | HHX190053885.g | 1.705581111 |
| SSHX020045911.g | 56.83898244 | HHX190053875.g | 3.177242667 |
| SSHX020045926.g | 3.908707333 | HHX080004958.g | 0.681533222 |
| SSHX020045928.g | 0.118820556 | HHX190053849.g | 1.727623889 |
| SSHX020045961.g | 3.344715 | HHX190053833.g | 0.002395556 |
| SSHX020045981.g | 31.080722 | HHX080005509.g | 3.952505222 |
| SSHX020046000.g | 0.010852667 | HHX080005056.g | 17.84403989 |
| SSHX020046013.g | 0.066893778 | HHX080005055.g | 1.896406 |
| SSHX020046038.g | 43.41593222 | HHX080005019.g | 0.727570444 |
| SSHX020046044.g | 3.756088778 | HHX080005014.g | 0.132151222 |
| SSHX020046058.g | 14.580584 | HHX080004998.g | 2.532004889 |
| SSHX020046060.g | 0.100000444 | HHX080005000.g | 2.479773333 |
| SSHX020046061.g | 0.778638556 | HHX080005001.g | 9.463607222 |
| SSHX020046084.g | 1.979718889 | HHX190053803.g | 0.112334 |
| SSHX020046086.g | 0.002305 | HHX190053800.g | 0.214380889 |
| SSHX020046095.g | 6.534754889 | HHX190053790.g | 1.269822333 |
| SSHX020046097.g | 8.297677444 | HHX080005087.g | 0.191136333 |
| SSHX020046103.g | 0.114385556 | HHX080005092.g | 0.018838111 |
| SSHX020046111.g | 0.072081667 | HHX080005094.g | 0.527314778 |
| SSHX020046113.g | 0.232910222 | HHX190053777.g | 1.769660444 |
| SSHX020046119.g | 0.048817333 | HHX080005099.g | 6.181632333 |
| SSHX020046121.g | 1.981621111 | HHX080005102.g | 0.308273222 |
| SSHX020046127.g | 0.639354667 | HHX190053769.g | 21.56890778 |
| SSHX020046176.g | 0.113727 | HHX080005118.g | 0.011928333 |
| SSHX020046179.g | 0.017057778 | HHX080005120.g | 3.099466222 |
| SSHX020046180.g | 0.780687778 | HHX080005124.g | 76.103901 |
| SSHX020046189.g | 0.819999444 | HHX080005136.g | 0.110983556 |
| SSHX020046195.g | 12.16506278 | HHX080005143.g | 1.324421111 |
| SSHX020046196.g | 2.194264889 | HHX080005144.g | 0.259156333 |
| SSHX020046197.g | 0.934545333 | HHX080005145.g | 5.100902444 |
| SSHX020046216.g | 0.004664333 | HHX080005152.g | 0.053940667 |
| SSHX020046265.g | 0.052311889 | HHX080005183.g | 0.369383667 |
| SSHX020046284.g | 5.180815778 | HHX080005186.g | 0.2698 |
| SSHX020046354.g | 41.40933956 | HHX080005206.g | 0.742489667 |
| SSHX020046358.g | 2.275401111 | HHX080005210.g | 20.21355944 |
| SSHX020046362.g | 2.423715556 | HHX080005215.g | 13.82643911 |
| SSHX020046366.g | 40.47283833 | HHX080005232.g | 1.667257222 |
| SSHX020046367.g | 1.179086 | HHX080005239.g | 12.03081867 |
| SSHX020046371.g | 0.963994667 | HHX080005204.g | 0.022746889 |
| SSHX020046375.g | 0.003770778 | HHX080005243.g | 1.557985111 |
| SSHX020046387.g | 1.212455111 | HHX080005194.g | 0.081659 |
| SSHX020046394.g | 0.008131556 | HHX190053522.g | 0.154049667 |
| SSHX020046395.g | 0.008806 | HHX190053521.g | 12.89688711 |
| SSHX020046396.g | 0.287043111 | HHX190053520.g | 0.043432111 |
| SSHX020046403.g | 1.504211667 | HHX080005279.g | 0.278259778 |
| SSHX020046407.g | 35.143525 | HHX080005281.g | 1.407038778 |
| SSHX020046414.g | 32.29843611 | HHX080005286.g | 198.6388362 |
| SSHX020046415.g | 5.820364444 | HHX080005287.g | 0.276598333 |
| SSHX020046417.g | 0.004841333 | HHX190053494.g | 0.408061667 |
| SSHX020046429.g | 2.036754 | HHX080005298.g | 0.065662667 |
| SSHX020046437.g | 98.32247667 | HHX190053489.g | 7.023577556 |
| SSHX020046451.g | 5.529515 | HHX080005324.g | 0.496447667 |
| SSHX020046454.g | 0.052173222 | HHX080005326.g | 0.292192556 |
| SSHX020046462.g | 7.025794333 | HHX080005332.g | 0.422543667 |
| SSHX020046476.g | 1.172823444 | HHX080005337.g | 0.044916667 |
| SSHX020046477.g | 13.197664 | HHX190053472.g | 1.760359 |
| SSHX020046479.g | 17.11267556 | HHX080005341.g | 0.310605778 |
| SSHX020046488.g | 13.19203944 | HHX190053468.g | 0.632896111 |
| SSHX020046507.g | 0.891260889 | HHX080005357.g | 0.009069222 |
| SSHX020046519.g | 0.025805222 | HHX080005365.g | 0.196357667 |
| SSHX020046538.g | 0.560288333 | HHX190053440.g | 35.39068111 |
| SSHX020046580.g | 6.591362778 | HHX190053390.g | 1.143946778 |
| SSHX020046597.g | 0.756932444 | HHX080005448.g | 20.16583056 |
| SSHX020046618.g | 0.988602778 | HHX080005480.g | 6.755187 |
| SSHX020046645.g | 0.040236556 | HHX080005526.g | 1.850183667 |
| SSHX020046682.g | 0.006005333 | HHX080002906.g | 0.047493444 |
| SSHX020046685.g | 0.015230556 | HHX080005537.g | 0.001979 |
| SSHX020046691.g | 0.002982444 | HHX080002944.g | 0.077226778 |
| SSHX020046707.g | 0.000565 | HHX080002943.g | 1.076187889 |
| SSHX020046715.g | 0.335317111 | HHX080002968.g | 0.016370556 |
| SSHX020046721.g | 0.238982556 | HHX080002906.g | 0.047493444 |
| SSHX020046722.g | 0.044673222 | HHX080002969.g | 0.514430222 |
| SSHX020046724.g | 0.279222667 | HHX080002972.g | 0.033754111 |
| SSHX020046733.g | 0.006470333 | HHX080002888.g | 0.590987444 |
| SSHX020046740.g | 0.034979556 | HHX080005537.g | 0.001979 |
| SSHX020046742.g | 0.036379 | HHX080002876.g | 0.287708778 |
| SSHX020046749.g | 0.035314667 | HHX080002974.g | 0.276036333 |
| SSHX020046766.g | 0.280042444 | HHX080002972.g | 0.033754111 |
| SSHX020046785.g | 0.022832778 | HHX080002945.g | 0.255334111 |
| SSHX020046794.g | 0.385113 | HHX080002972.g | 0.033754111 |
| SSHX020046821.g | 0.53376 | HHX080002987.g | 0.083861333 |
| SSHX020046853.g | 67.97925344 | HHX190053298.g | 0.062688444 |
| SSHX020046860.g | 1.708988667 | HHX080003022.g | 0.241151 |
| SSHX020046862.g | 3.430067444 | HHX080003024.g | 0.240546444 |
| SSHX020046865.g | 0.908697667 | HHX080003027.g | 0.127573222 |
| SSHX020046878.g | 0.563212222 | HHX190053239.g | 0.038742889 |
| SSHX020046896.g | 0.963463778 | HHX190053208.g | 4.978333889 |
| SSHX020046913.g | 0.038588667 | HHX190053200.g | 0.267059667 |
| SSHX020046927.g | 0.134041556 | HHX080003076.g | 0.688787333 |
| SSHX020046934.g | 0.032359 | HHX080003080.g | 0.288071333 |
| SSHX020046936.g | 2.177837444 | HHX190053195.g | 0.018751667 |
| SSHX020046979.g | 0.262017556 | HHX080003125.g | 3.236307889 |
| SSHX020046981.g | 1.171148667 | HHX080003127.g | 0.040577333 |
| SSHX020047038.g | 0.856352667 | HHX080003191.g | 0.087559889 |
| SSHX020047039.g | 0.060539222 | HHX080003192.g | 0.498486556 |
| SSHX020047040.g | 0.262545667 | HHX080003193.g | 1.898956556 |
| SSHX020047042.g | 16.01779033 | HHX080003194.g | 2.653869667 |
| SSHX020047045.g | 1.904490111 | HHX080003200.g | 9.921180556 |
| SSHX020047052.g | 5.547620778 | HHX080003209.g | 55.71315167 |
| SSHX020047057.g | 0.012116222 | HHX190053129.g | 0.066741222 |
| SSHX020047062.g | 0.029867222 | HHX080003226.g | 0.381406778 |
| SSHX020047083.g | 0.002507778 | HHX080003236.g | 0.026478333 |
| SSHX020047106.g | 10.26264178 | HHX080003250.g | 0.098785 |
| SSHX020047134.g | 2.721652444 | HHX080003273.g | 23.19577367 |
| SSHX020047149.g | 82.305896 | HHX080003338.g | 15.35575344 |
| SSHX020047156.g | 414.9059279 | HHX080003331.g | 2.731817111 |
| SSHX020047164.g | 2.101653111 | HHX080003318.g | 0.352510222 |
| SSHX020047182.g | 0.324004 | HHX090024689.g | 0.049168667 |
| SSHX020047231.g | 0.003811889 | HHX120002771.g | 0.057142556 |
| SSHX020047236.g | 1.819489111 | HHX120002781.g | 0.057205 |
| SSHX020047243.g | 52.27952567 | HHX120002787.g | 9.583464111 |
| SSHX020047354.g | 1.238736222 | HHX120002698.g | 23.83680467 |
| SSHX020047355.g | 24.46156644 | HHX120002697.g | 0.879269889 |
| SSHX020047394.g | 1.969886111 | HHX120002829.g | 0.323572444 |
| SSHX020047395.g | 0.939422111 | HHX120002831.g | 0.042912111 |
| SSHX020047440.g | 1.701257333 | HHX190054081.g | 0.062232222 |
| SSHX020047456.g | 3.272148 | HHX190054109.g | 0.365892889 |
| SSHX020047494.g | 0.008187222 | HHX190054149.g | 0.151566667 |
| SSHX020047502.g | 0.118567333 | HHX190054152.g | 1.057854333 |
| SSHX020047506.g | 0.623431556 | HHX190054157.g | 64.24676467 |
| SSHX020047539.g | 0.033764444 | HHX190054166.g | 1.373274556 |
| SSHX020047551.g | 2.027937556 | HHX190054182.g | 0.005733667 |
| SSHX020047571.g | 230.2888013 | HHX190054213.g | 1.055212222 |
| SSHX020047600.g | 5.419223667 | HHX190054241.g | 0.007605667 |
| SSHX020047653.g | 20.53608844 | HHX190054295.g | 1.734668667 |
| SSHX020047666.g | 0.001723778 | HHX190054305.g | 0.086170333 |
| SSHX020047674.g | 69.58561322 | HHX190054321.g | 0.019479333 |
| SSHX020047675.g | 1.318769333 | HHX190054322.g | 0.085823444 |
| SSHX020047681.g | 0.912889222 | HHX190054332.g | 10.417057 |
| SSHX020047703.g | 1.993021889 | HHX190054368.g | 12.19962711 |
| SSHX020047713.g | 28.39854122 | HHX190054395.g | 0.030906889 |
| SSHX020047752.g | 36.99253922 | HHX060019332.g | 201.8302681 |
| SSHX020047777.g | 832.678816 | HHX060019380.g | 21.05920667 |
| SSHX020047779.g | 1.030635111 | HHX060019385.g | 6.542220444 |
| SSHX020047808.g | 0.529919556 | HHX060019418.g | 38.39876356 |
| SSHX020047829.g | 0.148050111 | HHX060019450.g | 1.240361667 |
| SSHX020047839.g | 0.144418111 | HHX060019461.g | 1.061523778 |
| SSHX020047847.g | 0.006424111 | HHX060019467.g | 0.045665333 |
| SSHX020047884.g | 0.449667111 | HHX060019506.g | 36.63710589 |
| SSHX020047920.g | 53.35637389 | HHX050046153.g | 0.534529778 |
| SSHX020047925.g | 0.113123667 | HHX050046155.g | 12.52094067 |
| SSHX020047927.g | 0.564131333 | HHX090023820.g | 4.908509 |
| SSHX020047932.g | 2.396146556 | HHX050046163.g | 0.344797889 |
| SSHX020047933.g | 279.0420056 | HHX050046165.g | 21.60431667 |
| SSHX020047934.g | 193.09218 | HHX090023819.g | 0.690653333 |
| SSHX020047936.g | 3.5878 | HHX090023813.g | 0.045710333 |
| SSHX020047939.g | 0.126287444 | HHX090023830.g | 0.004059778 |
| SSHX020047945.g | 0.708801 | HHX090023836.g | 656.8392361 |
| SSHX020047947.g | 476.1745588 | HHX090023819.g | 0.690653333 |
| SSHX020047959.g | 4.450318333 | HHX090023837.g | 122.7544339 |
| SSHX020048057.g | 1.991413889 | HHX190053063.g | 39.46755133 |
| SSHX020048059.g | 7.414050556 | HHX190053069.g | 0.082085333 |
| SSHX020048102.g | 0.060671556 | HHX080003166.g | 0.539087889 |
| SSHX020048110.g | 0.242988333 | HHX190053131.g | 3.931180222 |
| SSHX020048124.g | 0.820988778 | HHX080003151.g | 0.072307556 |
| SSHX020048129.g | 0.027414222 | HHX080003149.g | 0.450890889 |
| SSHX020048130.g | 39.80759278 | HHX190053151.g | 0.019146889 |
| SSHX020048155.g | 0.706975556 | HHX080003144.g | 0.125920222 |
| SSHX020048164.g | 0.096643111 | HHX080003142.g | 0.003715111 |
| SSHX020048170.g | 6.500135889 | HHX190053174.g | 0.401057556 |
| SSHX020048171.g | 5.424468111 | HHX080003133.g | 0.064345444 |
| SSHX020048199.g | 0.163727778 | HHX190053198.g | 0.025783889 |
| SSHX020048200.g | 0.166544667 | HHX190053251.g | 0.023087889 |
| SSHX020048205.g | 1.629093 | HHX190053203.g | 0.106160444 |
| SSHX020048207.g | 1.741684333 | HHX190053206.g | 0.053617556 |
| SSHX020048210.g | 1.825609889 | HHX190053213.g | 0.142507444 |
| SSHX020048217.g | 0.742176111 | HHX190053283.g | 0.065939778 |
| SSHX020048219.g | 0.334829333 | HHX190053262.g | 0.004237111 |
| SSHX020048231.g | 0.046683667 | HHX190053236.g | 0.247217333 |
| SSHX020048240.g | 13.07578978 | HHX190053259.g | 0.319477111 |
| SSHX020048243.g | 0.003687778 | HHX190053275.g | 0.024690444 |
| SSHX020048248.g | 0.007216333 | HHX190053250.g | 0.088971222 |
| SSHX020048262.g | 0.004689333 | HHX190053283.g | 0.065939778 |
| SSHX020048271.g | 0.014678556 | HHX190053327.g | 0.000766444 |
| SSHX020048278.g | 18.88653511 | HHX190053315.g | 1.045221778 |
| SSHX020048280.g | 0.395530444 | HHX190053317.g | 8.814091444 |
| SSHX020048290.g | 0.004417778 | HHX190053283.g | 0.065939778 |
| SSHX020048311.g | 0.351235667 | HHX190053350.g | 4.407856111 |
| SSHX020048324.g | 0.017447 | HHX190053372.g | 1.890195111 |
| SSHX020048335.g | 4.103672 | HHX190053386.g | 0.816048667 |
| SSHX020048336.g | 5.153803111 | HHX190053387.g | 0.400002111 |
| SSHX020048345.g | 0.167573889 | HHX190053393.g | 0.897244222 |
| SSHX020048346.g | 0.274140778 | HHX080005420.g | 2.326885 |
| SSHX020048363.g | 0.013461 | HHX190053413.g | 131.0637559 |
| SSHX020048370.g | 5.670048444 | HHX190053426.g | 0.283846333 |
| SSHX020048373.g | 26.59205211 | HHX080005402.g | 0.177289333 |
| SSHX020048374.g | 2.289866222 | HHX190053432.g | 0.301284 |
| SSHX020048382.g | 25.06329989 | HHX080005382.g | 1.930791 |
| SSHX020048386.g | 5.940773556 | HHX190053445.g | 0.146410556 |
| SSHX020048395.g | 0.233509333 | HHX080005357.g | 0.009069222 |
| SSHX020048399.g | 0.738083111 | HHX190053453.g | 13.809208 |
| SSHX020048401.g | 0.076312889 | HHX190053457.g | 0.479068778 |
| SSHX020048405.g | 0.575622333 | HHX080005344.g | 20.26722722 |
| SSHX020048409.g | 92.11896667 | HHX190053468.g | 0.632896111 |
| SSHX020048412.g | 0.076557111 | HHX190053472.g | 1.760359 |
| SSHX020048416.g | 29.88683822 | HHX080005338.g | 2.916871889 |
| SSHX020048418.g | 5.092179444 | HHX080005337.g | 0.044916667 |
| SSHX020048419.g | 1.424914333 | HHX190053475.g | 11.16917222 |
| SSHX020048420.g | 273.2525142 | HHX190053476.g | 7.383477556 |
| SSHX020048449.g | 0.003692778 | HHX080005309.g | 0.967334222 |
| SSHX020048451.g | 1.570877333 | HHX080005307.g | 51.53982833 |
| SSHX020048453.g | 32.140878 | HHX190053492.g | 1.558598556 |
| SSHX020048455.g | 0.260254444 | HHX080005288.g | 0.007171111 |
| SSHX020048458.g | 1.689850778 | HHX080005287.g | 0.276598333 |
| SSHX020048470.g | 1.732702556 | HHX190053505.g | 0.254418667 |
| SSHX020048474.g | 0.715850444 | HHX190053507.g | 0.056875 |
| SSHX020048479.g | 0.440960222 | HHX190053513.g | 2.404313444 |
| SSHX020048480.g | 3.643719111 | HHX190053514.g | 19.61852411 |
| SSHX020048490.g | 1.939841111 | HHX080005269.g | 0.018548667 |
| SSHX020048492.g | 75.309157 | HHX080005260.g | 0.798864556 |
| SSHX020048509.g | 1.121319444 | HHX190053557.g | 0.083888 |
| SSHX020048523.g | 0.010980111 | HHX190053574.g | 0.384004889 |
| SSHX020048526.g | 0.348431778 | HHX190053577.g | 0.026941444 |
| SSHX020048542.g | 8.359614667 | HHX190053586.g | 0.173295222 |
| SSHX020048544.g | 0.035440667 | HHX190053588.g | 10.45612256 |
| SSHX020048550.g | 0.018856889 | HHX190053593.g | 0.436239778 |
| SSHX020048594.g | 13.40494889 | HHX190053615.g | 1.685503778 |
| SSHX020048605.g | 29.36003789 | HHX190053621.g | 3.635530444 |
| SSHX020048632.g | 0.531642556 | HHX190053627.g | 105.8207 |
| SSHX020048635.g | 4.407957667 | HHX190053634.g | 0.620075333 |
| SSHX020048638.g | 0.578254111 | HHX190053635.g | 18.71542111 |
| SSHX020048661.g | 19.64897211 | HHX190053664.g | 0.907537444 |
| SSHX020048675.g | 34.04859533 | HHX190053679.g | 1.365203667 |
| SSHX020048709.g | 2.788901778 | HHX190053681.g | 0.551833 |
| SSHX020048744.g | 3.22825 | HHX190053721.g | 0.010293333 |
| SSHX020048745.g | 0.354542333 | HHX190053768.g | 3158.695136 |
| SSHX020048747.g | 9.782847333 | HHX190053760.g | 0.578588222 |
| SSHX020048750.g | 0.020404444 | HHX190053756.g | 1.633691778 |
| SSHX020048753.g | 1.345516 | HHX190053735.g | 0.057900444 |
| SSHX020048757.g | 0.002669222 | HHX190053745.g | 0.210717111 |
| SSHX020048760.g | 0.000820778 | HHX190053743.g | 0.009059222 |
| SSHX020048765.g | 8.437406889 | HHX190053739.g | 0.558274333 |
| SSHX020048767.g | 2.029172 | HHX190053735.g | 0.057900444 |
| SSHX020048771.g | 0.019733111 | HHX190053755.g | 0.831805444 |
| SSHX020048791.g | 0.004709667 | HHX190053787.g | 2.500944667 |
| SSHX020048793.g | 0.018071556 | HHX190053784.g | 0.358083778 |
| SSHX020048799.g | 1.707675 | HHX190053792.g | 0.047054778 |
| SSHX020048800.g | 0.011642556 | HHX190053793.g | 0.097006222 |
| SSHX020048805.g | 0.047671111 | HHX190053799.g | 0.455081778 |
| SSHX020048814.g | 0.049943 | HHX190053805.g | 1.697568667 |
| SSHX020048830.g | 4.327480333 | HHX190053808.g | 0.715554111 |
| SSHX020048844.g | 29.86370578 | HHX190053821.g | 1.553605556 |
| SSHX020048870.g | 0.029224556 | HHX080004961.g | 95.977987 |
| SSHX020048885.g | 0.247874111 | HHX190053868.g | 0.047012333 |
| SSHX020048893.g | 6.692757111 | HHX080004948.g | 69.70048767 |
| SSHX020048900.g | 1.056661667 | HHX190053882.g | 18.59891944 |
| SSHX020048928.g | 139.6970478 | HHX190053914.g | 20.30403278 |
| SSHX020048929.g | 2.587617333 | HHX190053916.g | 0.487374333 |
| SSHX020048934.g | 10.05405444 | HHX190053919.g | 1.539979222 |
| SSHX020048936.g | 44.24008678 | HHX190053920.g | 3.099431333 |
| SSHX020048938.g | 9.083077889 | HHX080004917.g | 1.190481778 |
| SSHX020048949.g | 0.004519333 | HHX080004893.g | 0.267765889 |
| SSHX020048952.g | 0.324304 | HHX190053947.g | 9.357898667 |
| SSHX020048953.g | 0.016962 | HHX080004873.g | 0.339708889 |
| SSHX020048961.g | 0.140611333 | HHX190053956.g | 13.76943956 |
| SSHX020049002.g | 0.191145556 | HHX190054004.g | 1.825943889 |
| SSHX020049005.g | 3.697618667 | HHX190054006.g | 20.45039333 |
| SSHX020049014.g | 0.154509778 | HHX190054017.g | 1.558669667 |
| SSHX020049021.g | 6.235094 | HHX190054027.g | 44.16900411 |
| SSHX020049033.g | 0.060265667 | HHX190054037.g | 0.86227 |
| SSHX020049036.g | 0.170116667 | HHX190054040.g | 4.165016667 |
| SSHX020049044.g | 47.98256744 | HHX190054052.g | 1.193046111 |
| SSHX020049059.g | 0.479791444 | HHX190054069.g | 6.865330778 |
| SSHX030028514.g | 0.051620222 | HHX030012737.g | 7.091052111 |
| SSHX030028516.g | 1.436123444 | HHX030012741.g | 0.046573667 |
| SSHX030028522.g | 0.094786 | HHX030012752.g | 5.755785333 |
| SSHX030028526.g | 0.188844889 | HHX030012754.g | 3.698000333 |
| SSHX030028543.g | 2.781404889 | HHX030012765.g | 0.071127 |
| SSHX030028567.g | 8.110795 | HHX030012783.g | 1.180487556 |
| SSHX030028570.g | 19.01390089 | HHX030012786.g | 2.178410222 |
| SSHX030028591.g | 0.305452667 | HHX030012809.g | 1.985712222 |
| SSHX030028600.g | 0.740781556 | HHX030012820.g | 22.08755244 |
| SSHX030028611.g | 0.924341889 | HHX030012825.g | 0.121276 |
| SSHX030028632.g | 2.809438556 | HHX030012715.g | 81.02518556 |
| SSHX030028633.g | 10.29085344 | HHX030012714.g | 134.608613 |
| SSHX030028643.g | 0.232618556 | HHX030012702.g | 0.043873667 |
| SSHX030028649.g | 0.297250889 | HHX030012671.g | 0.018352222 |
| SSHX030028656.g | 0.978212889 | HHX030012665.g | 0.122483778 |
| SSHX030028657.g | 71.38349944 | HHX030012664.g | 8.905702 |
| SSHX030028659.g | 0.418850333 | HHX030012656.g | 0.055897444 |
| SSHX030028661.g | 22.58305367 | HHX030012659.g | 0.309703889 |
| SSHX030028662.g | 16.98730433 | HHX030012623.g | 0.043002222 |
| SSHX030028690.g | 1.352938333 | HHX030012582.g | 0.204113556 |
| SSHX030028698.g | 0.003669556 | HHX030012613.g | 0.205307111 |
| SSHX030028700.g | 0.039176889 | HHX030012604.g | 0.260032222 |
| SSHX030028702.g | 0.671701111 | HHX030012615.g | 0.034325778 |
| SSHX030028706.g | 1.298290222 | HHX030012587.g | 0.005677111 |
| SSHX030028709.g | 1.530965556 | HHX030012611.g | 22.21355956 |
| SSHX030028710.g | 0.230464444 | HHX030012610.g | 5.368746778 |
| SSHX030028728.g | 160.0917029 | HHX030012607.g | 15.90349378 |
| SSHX030028748.g | 3.888211444 | HHX030012570.g | 0.560876111 |
| SSHX030028752.g | 0.692115556 | HHX030012565.g | 5.103314667 |
| SSHX030028779.g | 45.78976733 | HHX030012542.g | 4.340865889 |
| SSHX030028782.g | 0.048027889 | HHX030012540.g | 0.424341556 |
| SSHX030028825.g | 0.051200333 | HHX030012506.g | 0.001246667 |
| SSHX030028849.g | 428.9681224 | HHX030012475.g | 1.077141556 |
| SSHX030028865.g | 17.76964767 | HHX140043498.g | 2.746840556 |
| SSHX030028866.g | 25.961406 | HHX140043541.g | 2.842057111 |
| SSHX030028869.g | 0.706046889 | HHX030012430.g | 0.018922778 |
| SSHX030028874.g | 0.844766444 | HHX030012398.g | 0.138269111 |
| SSHX030028875.g | 5.680789111 | HHX030012430.g | 0.018922778 |
| SSHX030028879.g | 0.171087222 | HHX140043299.g | 0.005711 |
| SSHX030028880.g | 0.073478333 | HHX030012442.g | 4.964064 |
| SSHX030028882.g | 16.56178 | HHX030012404.g | 1.922501 |
| SSHX030028883.g | 4.811152778 | HHX140043376.g | 0.020466 |
| SSHX030028889.g | 0.365299889 | HHX030012419.g | 3.936802 |
| SSHX030028890.g | 0.044192556 | HHX140043428.g | 0.395292778 |
| SSHX030028892.g | 1.745577111 | HHX140043376.g | 0.020466 |
| SSHX030028893.g | 1.349811667 | HHX140043335.g | 0.100676222 |
| SSHX030028895.g | 2.326214889 | HHX030012430.g | 0.018922778 |
| SSHX030028912.g | 2.755787333 | HHX030012459.g | 0.544613333 |
| SSHX030028913.g | 0.089868111 | HHX030012453.g | 5.476838222 |
| SSHX030028920.g | 3.994693 | HHX030012394.g | 0.141447111 |
| SSHX030028921.g | 0.292581222 | HHX030012453.g | 5.476838222 |
| SSHX030028922.g | 0.974509667 | HHX030012395.g | 0.127747556 |
| SSHX030028925.g | 0.133480667 | HHX140043445.g | 2.612890556 |
| SSHX030028926.g | 0.063731111 | HHX140043338.g | 0.011380333 |
| SSHX030028928.g | 0.010431889 | HHX030012395.g | 0.127747556 |
| SSHX030028929.g | 0.113097667 | HHX030012458.g | 3.995523333 |
| SSHX030028931.g | 9.172486222 | HHX140043558.g | 0.283162556 |
| SSHX030028932.g | 0.476635889 | HHX140043376.g | 0.020466 |
| SSHX030028933.g | 0.759690556 | HHX030012419.g | 3.936802 |
| SSHX030028934.g | 0.065129889 | HHX140043537.g | 0.005451444 |
| SSHX030028935.g | 0.285352556 | HHX030012453.g | 5.476838222 |
| SSHX030028936.g | 7.637766667 | HHX140043563.g | 0.273521111 |
| SSHX030028944.g | 0.294217778 | HHX030012390.g | 2.646373222 |
| SSHX030028946.g | 0.200040778 | HHX030012388.g | 1.407892778 |
| SSHX030028948.g | 0.152001333 | HHX140043577.g | 0.008749333 |
| SSHX030028952.g | 16.24323556 | HHX030012381.g | 3.009683889 |
| SSHX030028964.g | 0.980197333 | HHX030012363.g | 0.131412111 |
| SSHX030028975.g | 0.505774778 | HHX030012354.g | 3.096068889 |
| SSHX030028982.g | 0.282267 | HHX030012346.g | 6.985752333 |
| SSHX030028984.g | 0.606484 | HHX030012342.g | 0.072126667 |
| SSHX030028998.g | 1.277081889 | HHX030012317.g | 0.163967444 |
| SSHX030028999.g | 0.380477444 | HHX030012308.g | 0.034891667 |
| SSHX030029002.g | 0.127657 | HHX030012309.g | 0.004775889 |
| SSHX030029013.g | 0.002390222 | HHX030012317.g | 0.163967444 |
| SSHX030029014.g | 0.007312111 | HHX030012287.g | 1.392228889 |
| SSHX030029016.g | 0.002440889 | HHX030012318.g | 0.053917444 |
| SSHX030029020.g | 0.601064333 | HHX030012305.g | 38.63177578 |
| SSHX030029022.g | 2.090225 | HHX030012304.g | 10.47498189 |
| SSHX030029023.g | 0.718894667 | HHX030012303.g | 4.482104444 |
| SSHX030029025.g | 0.136675 | HHX030012291.g | 1.149719444 |
| SSHX030029028.g | 13.85512922 | HHX030012308.g | 0.034891667 |
| SSHX030029029.g | 39.05691344 | HHX030012289.g | 0.935052222 |
| SSHX030029033.g | 2.456682889 | HHX030012309.g | 0.004775889 |
| SSHX030029058.g | 0.1997 | HHX030012261.g | 2.689275 |
| SSHX030029062.g | 151.4680076 | HHX030012258.g | 8.035029778 |
| SSHX030029063.g | 2.791554667 | HHX030012249.g | 0.206834222 |
| SSHX030029072.g | 0.335934556 | HHX030012147.g | 0.025834333 |
| SSHX030029076.g | 78.36317767 | HHX030012248.g | 14.37761911 |
| SSHX030029088.g | 0.085175333 | HHX030012198.g | 1.387594333 |
| SSHX030029090.g | 0.745328889 | HHX030012222.g | 0.002827889 |
| SSHX030029096.g | 0.004421778 | HHX030012211.g | 0.127371111 |
| SSHX030029104.g | 0.406087111 | HHX030012119.g | 0.009955889 |
| SSHX030029105.g | 6.846280222 | HHX030012146.g | 0.120896444 |
| SSHX030029110.g | 1.994690333 | HHX030012148.g | 0.14285 |
| SSHX030029111.g | 1.046914333 | HHX140042623.g | 0.037711556 |
| SSHX030029117.g | 0.154580111 | HHX030012126.g | 0.007946444 |
| SSHX030029128.g | 0.060847667 | HHX030012119.g | 0.009955889 |
| SSHX030029129.g | 0.501212 | HHX140042504.g | 0.001533111 |
| SSHX030029136.g | 1.424767556 | HHX030012109.g | 0.077959778 |
| SSHX030029137.g | 0.103327556 | HHX030012119.g | 0.009955889 |
| SSHX030029140.g | 0.042305 | HHX030012126.g | 0.007946444 |
| SSHX030029144.g | 0.215587556 | HHX140042504.g | 0.001533111 |
| SSHX030029145.g | 0.039333111 | HHX030012162.g | 9.538087111 |
| SSHX030029147.g | 1.173499778 | HHX030012148.g | 0.14285 |
| SSHX030029148.g | 1.477162222 | HHX030012187.g | 0.192806222 |
| SSHX030029150.g | 0.134424222 | HHX030012127.g | 5.835089 |
| SSHX030029151.g | 18.68386711 | HHX030012103.g | 0.153791889 |
| SSHX030029152.g | 0.264078556 | HHX140042795.g | 0.000463889 |
| SSHX030029153.g | 0.089631667 | HHX030012239.g | 0.011351889 |
| SSHX030029154.g | 0.136363889 | HHX030012137.g | 0.000870889 |
| SSHX030029155.g | 0.160718 | HHX030012154.g | 9.150593333 |
| SSHX030029162.g | 0.094158889 | HHX030012154.g | 9.150593333 |
| SSHX030029163.g | 0.000492222 | HHX030012176.g | 0.129625111 |
| SSHX030029164.g | 0.132704 | HHX030012119.g | 0.009955889 |
| SSHX030029165.g | 0.057677889 | HHX030012162.g | 9.538087111 |
| SSHX030029166.g | 0.551340778 | HHX140042651.g | 0.011169556 |
| SSHX030029173.g | 0.024616111 | HHX030012165.g | 0.863477222 |
| SSHX030029180.g | 0.019471556 | HHX140042768.g | 0.129420333 |
| SSHX030029181.g | 0.812674222 | HHX030012109.g | 0.077959778 |
| SSHX030029182.g | 0.439646333 | HHX030012137.g | 0.000870889 |
| SSHX030029183.g | 1.051816889 | HHX030012155.g | 0.132893333 |
| SSHX030029184.g | 52.21286056 | HHX030012163.g | 0.771323889 |
| SSHX030029188.g | 2.514002222 | HHX030012137.g | 0.000870889 |
| SSHX030029189.g | 8.669822222 | HHX030012194.g | 0.179194889 |
| SSHX030029195.g | 0.087010333 | HHX030012119.g | 0.009955889 |
| SSHX030029196.g | 0.602062444 | HHX030012154.g | 9.150593333 |
| SSHX030029197.g | 0.158311111 | HHX030012111.g | 14.86654844 |
| SSHX030029199.g | 0.062112 | HHX140042615.g | 3.852221556 |
| SSHX030029204.g | 0.030417444 | HHX030012149.g | 2.350552444 |
| SSHX030029205.g | 0.038607444 | HHX030012137.g | 0.000870889 |
| SSHX030029206.g | 0.047161444 | HHX030012198.g | 1.387594333 |
| SSHX030029207.g | 0.102820778 | HHX030012163.g | 0.771323889 |
| SSHX030029209.g | 0.052118333 | HHX030012143.g | 0.328886 |
| SSHX030029210.g | 0.005985667 | HHX030012122.g | 0.695630222 |
| SSHX030029211.g | 0.008645 | HHX030012137.g | 0.000870889 |
| SSHX030029212.g | 0.021327222 | HHX030012165.g | 0.863477222 |
| SSHX030029218.g | 0.220013444 | HHX030012138.g | 0.038215556 |
| SSHX030029219.g | 0.020429667 | HHX030012206.g | 7.133784556 |
| SSHX030029221.g | 0.013048778 | HHX140042795.g | 0.000463889 |
| SSHX030029229.g | 0.478441333 | HHX030012138.g | 0.038215556 |
| SSHX030029230.g | 0.012112 | HHX030012165.g | 0.863477222 |
| SSHX030029236.g | 0.012871 | HHX030012182.g | 0.159744556 |
| SSHX030029241.g | 21.25263922 | HHX030012115.g | 2.697239333 |
| SSHX030029244.g | 0.451538889 | HHX030012112.g | 2.414325333 |
| SSHX030029246.g | 20.49304978 | HHX030012194.g | 0.179194889 |
| SSHX030029249.g | 0.030377556 | HHX030012163.g | 0.771323889 |
| SSHX030029250.g | 0.270952222 | HHX030012147.g | 0.025834333 |
| SSHX030029251.g | 1.681980556 | HHX030012103.g | 0.153791889 |
| SSHX030029256.g | 4.394749222 | HHX030012105.g | 0.740941889 |
| SSHX030029267.g | 0.242276889 | HHX030012063.g | 0.039178667 |
| SSHX030029269.g | 1.892941667 | HHX030012064.g | 0.002265444 |
| SSHX030029271.g | 0.273711444 | HHX030012074.g | 2.112649444 |
| SSHX030029274.g | 0.086818 | HHX030012073.g | 11.205034 |
| SSHX030029293.g | 0.017547556 | HHX030012064.g | 0.002265444 |
| SSHX030029307.g | 37.67203511 | HHX030012051.g | 7.166997222 |
| SSHX030029311.g | 4.096066333 | HHX030012044.g | 0.425343778 |
| SSHX030029316.g | 1.156178889 | HHX030012033.g | 0.022736778 |
| SSHX030029324.g | 0.320149556 | HHX030012021.g | 1.624576444 |
| SSHX030029342.g | 0.183815556 | HHX030011998.g | 1.603046778 |
| SSHX030029374.g | 331.6297013 | HHX030011963.g | 1.540408333 |
| SSHX030029376.g | 0.002456222 | HHX030011961.g | 0.044963222 |
| SSHX030029378.g | 32.47122022 | HHX030011956.g | 0.891160889 |
| SSHX030029379.g | 67.158684 | HHX030011954.g | 0.032784778 |
| SSHX030029395.g | 0.104900667 | HHX030011912.g | 8.394886778 |
| SSHX030029396.g | 0.069740556 | HHX030011878.g | 8.095287 |
| SSHX030029398.g | 0.271260444 | HHX030011919.g | 2.735800111 |
| SSHX030029401.g | 0.09151 | HHX030011868.g | 0.003585556 |
| SSHX030029412.g | 60.80470456 | HHX030011893.g | 3.173168556 |
| SSHX030029418.g | 0.122798444 | HHX030011878.g | 8.095287 |
| SSHX030029419.g | 0.046779556 | HHX030011908.g | 1.068065 |
| SSHX030029422.g | 7.339483 | HHX030011868.g | 0.003585556 |
| SSHX030029427.g | 3.610554 | HHX030011896.g | 0.245267444 |
| SSHX030029428.g | 22.64246811 | HHX030011893.g | 3.173168556 |
| SSHX030029431.g | 5.895512667 | HHX030011908.g | 1.068065 |
| SSHX030029442.g | 0.071271 | HHX030011868.g | 0.003585556 |
| SSHX030029452.g | 7.646503333 | HHX030011908.g | 1.068065 |
| SSHX030029453.g | 10.08470067 | HHX030011945.g | 0.027783111 |
| SSHX030029458.g | 0.004215889 | HHX030011919.g | 2.735800111 |
| SSHX030029462.g | 0.985594 | HHX030011868.g | 0.003585556 |
| SSHX030029463.g | 0.723431 | HHX030011923.g | 0.023647778 |
| SSHX030029475.g | 0.037255333 | HHX030011926.g | 6.049712556 |
| SSHX030029502.g | 2.566068222 | HHX030011844.g | 0.041192889 |
| SSHX030029503.g | 8.950451667 | HHX030011845.g | 0.430993889 |
| SSHX030029510.g | 5.765557222 | HHX030011842.g | 39.06441178 |
| SSHX030029512.g | 0.689635333 | HHX030011839.g | 4.019333333 |
| SSHX030029523.g | 0.030532222 | HHX030011832.g | 0.199163778 |
| SSHX030029524.g | 0.006950667 | HHX060016964.g | 0.000360111 |
| SSHX030029540.g | 0.074758889 | HHX060017000.g | 0.011965556 |
| SSHX030029542.g | 0.168906333 | HHX060016982.g | 3.849418111 |
| SSHX030029543.g | 0.022652778 | HHX060016989.g | 5.168094111 |
| SSHX030029544.g | 0.144822778 | HHX030011813.g | 51.50908211 |
| SSHX030029546.g | 0.381880333 | HHX060016981.g | 4.513641556 |
| SSHX030029547.g | 8.698936556 | HHX030011816.g | 0.910119778 |
| SSHX030029550.g | 0.001948667 | HHX060017003.g | 0.417434333 |
| SSHX030029551.g | 0.002482667 | HHX030011818.g | 0.017005333 |
| SSHX030029553.g | 0.236008 | HHX060016989.g | 5.168094111 |
| SSHX030029563.g | 0.010730222 | HHX030011811.g | 0.339411 |
| SSHX030029568.g | 4.744526667 | HHX030011813.g | 51.50908211 |
| SSHX030029569.g | 16.30080411 | HHX030011825.g | 0.063087889 |
| SSHX030029578.g | 0.920381222 | HHX030011810.g | 71.03368533 |
| SSHX030029594.g | 11.03427956 | HHX030011792.g | 1.343753556 |
| SSHX030029600.g | 0.195506556 | HHX030011789.g | 0.018998444 |
| SSHX030029603.g | 11.96638556 | HHX030011788.g | 1.858198667 |
| SSHX030029606.g | 36.42212289 | HHX140041764.g | 0.930962 |
| SSHX030029618.g | 21.57505589 | HHX030011774.g | 2.509165111 |
| SSHX030029619.g | 116.3723306 | HHX140041782.g | 9.502619222 |
| SSHX030029631.g | 0.089280778 | HHX140041810.g | 16.17472244 |
| SSHX030029635.g | 0.256033111 | HHX140041836.g | 71.58409756 |
| SSHX030029648.g | 15.51738433 | HHX030011754.g | 2.615181556 |
| SSHX030029656.g | 125.8551957 | HHX030011746.g | 11.70632078 |
| SSHX030029657.g | 2.595630889 | HHX030011745.g | 40.02964289 |
| SSHX030029660.g | 0.216285444 | HHX030011743.g | 0.042754667 |
| SSHX030029661.g | 0.034010778 | HHX030011741.g | 1.216926889 |
| SSHX030029666.g | 0.168333111 | HHX030011739.g | 0.026172778 |
| SSHX030029696.g | 2.757081889 | HHX030011721.g | 0.046259333 |
| SSHX030029697.g | 1.985435889 | HHX030011719.g | 0.136039222 |
| SSHX030029703.g | 8.732108333 | HHX030011712.g | 0.598793778 |
| SSHX030029713.g | 9.160390667 | HHX030011703.g | 0.102464778 |
| SSHX030029739.g | 5.735877111 | HHX030011684.g | 0.881809333 |
| SSHX030029743.g | 0.202917889 | HHX030011676.g | 1.677898444 |
| SSHX030029745.g | 0.215569 | HHX030011674.g | 0.025939222 |
| SSHX030029757.g | 0.875951667 | HHX030011667.g | 6.710862444 |
| SSHX030029758.g | 0.667316556 | HHX030011666.g | 0.126149333 |
| SSHX030029767.g | 0.020915333 | HHX030011657.g | 0.109566889 |
| SSHX030029801.g | 0.107389778 | HHX030011619.g | 0.632819778 |
| SSHX030029815.g | 106.9579151 | HHX030011603.g | 4.500109778 |
| SSHX030029825.g | 2.392798889 | HHX030011592.g | 0.103104444 |
| SSHX030029837.g | 0.156995778 | HHX030011580.g | 0.855564889 |
| SSHX030029848.g | 37.27327533 | HHX030011048.g | 6.426885556 |
| SSHX030029865.g | 0.003941111 | HHX060016568.g | 6.518921667 |
| SSHX030029877.g | 19.76451333 | HHX030011059.g | 170.8140844 |
| SSHX030029879.g | 0.084997111 | HHX030011065.g | 1.003574889 |
| SSHX030029885.g | 0.533902333 | HHX030011067.g | 4.030339889 |
| SSHX030029897.g | 0.818817778 | HHX030011074.g | 0.147269444 |
| SSHX030029902.g | 12.54449422 | HHX030011078.g | 0.458832444 |
| SSHX030029918.g | 19.44323333 | HHX030011059.g | 170.8140844 |
| SSHX030029922.g | 0.168113778 | HHX030011065.g | 1.003574889 |
| SSHX030029923.g | 2.750454556 | HHX030011100.g | 0.184643111 |
| SSHX030029925.g | 0.115052333 | HHX030011191.g | 4.190251889 |
| SSHX030029926.g | 0.157186556 | HHX030011236.g | 1.435354778 |
| SSHX030029927.g | 0.150879667 | HHX030011194.g | 6.950048444 |
| SSHX030029928.g | 0.048942111 | HHX030011208.g | 0.006356111 |
| SSHX030029940.g | 0.034373556 | HHX030011230.g | 2.833744556 |
| SSHX030029946.g | 0.142996 | HHX030011236.g | 1.435354778 |
| SSHX030029982.g | 0.504901667 | HHX030011109.g | 0.060999 |
| SSHX030029997.g | 0.863877333 | HHX030011088.g | 0.002118222 |
| SSHX030030000.g | 3.421174778 | HHX030011090.g | 0.458737222 |
| SSHX030030026.g | 0.088245111 | HHX030011242.g | 0.953393556 |
| SSHX030030035.g | 3.070071778 | HHX030011109.g | 0.060999 |
| SSHX030030036.g | 4.800602667 | HHX030011242.g | 0.953393556 |
| SSHX030030037.g | 2.738726222 | HHX030011214.g | 0.071210222 |
| SSHX030030039.g | 2.062680111 | HHX030011110.g | 0.289688889 |
| SSHX030030046.g | 0.085003556 | HHX030011067.g | 4.030339889 |
| SSHX030030047.g | 0.237518889 | HHX030011236.g | 1.435354778 |
| SSHX030030048.g | 0.125300222 | HHX030011238.g | 0.024506222 |
| SSHX030030060.g | 0.083400444 | HHX030011136.g | 0.796762 |
| SSHX030030102.g | 6.32222E-05 | HHX030011179.g | 0.007562222 |
| SSHX030030106.g | 14.58718756 | HHX030011184.g | 141.3027501 |
| SSHX030030107.g | 0.561211222 | HHX030011186.g | 12.69755389 |
| SSHX030030126.g | 0.014531667 | HHX030011236.g | 1.435354778 |
| SSHX030030129.g | 0.111923 | HHX030011208.g | 0.006356111 |
| SSHX030030131.g | 3.683398556 | HHX030011070.g | 0.569922667 |
| SSHX030030132.g | 3.068668222 | HHX030011224.g | 0.138630222 |
| SSHX030030138.g | 0.119373667 | HHX030011236.g | 1.435354778 |
| SSHX030030139.g | 0.083334444 | HHX030011274.g | 0.530332889 |
| SSHX030030140.g | 5.485243222 | HHX030011280.g | 0.248534889 |
| SSHX030030150.g | 0.451047444 | HHX030011254.g | 0.011630556 |
| SSHX030030153.g | 0.303246556 | HHX030011268.g | 1.626978889 |
| SSHX030030159.g | 0.105322667 | HHX030011230.g | 2.833744556 |
| SSHX030030160.g | 0.698630444 | HHX030011108.g | 0.032697111 |
| SSHX030030162.g | 4.918329222 | HHX030011218.g | 0.740620778 |
| SSHX030030169.g | 5.233511889 | HHX030011272.g | 0.163478444 |
| SSHX030030181.g | 0.010301889 | HHX030011285.g | 0.335050556 |
| SSHX030030211.g | 0.893950444 | HHX030011316.g | 0.050006667 |
| SSHX030030212.g | 0.043820111 | HHX030011317.g | 2.473761444 |
| SSHX030030222.g | 16.97011911 | HHX030011323.g | 0.171104444 |
| SSHX030030224.g | 1.021013111 | HHX030011327.g | 0.174911333 |
| SSHX030030235.g | 0.982204778 | HHX030011334.g | 0.047227667 |
| SSHX030030272.g | 0.095228444 | HHX030011390.g | 16.625653 |
| SSHX030030274.g | 0.024910111 | HHX030011425.g | 0.301777444 |
| SSHX030030303.g | 6.031492 | HHX030011391.g | 0.282351 |
| SSHX030030313.g | 0.099125778 | HHX030011382.g | 1.550621667 |
| SSHX030030315.g | 0.005042667 | HHX030011380.g | 0.066500667 |
| SSHX030030319.g | 221.2078703 | HHX030011431.g | 6.460497889 |
| SSHX030030323.g | 0.019033556 | HHX030011434.g | 0.306384778 |
| SSHX030030324.g | 4.464008667 | HHX030011435.g | 0.657604667 |
| SSHX030030345.g | 0.042741444 | HHX030011456.g | 0.001664222 |
| SSHX030030365.g | 0.068079778 | HHX030011456.g | 0.001664222 |
| SSHX030030370.g | 0.062283667 | HHX030011450.g | 29.61587778 |
| SSHX030030377.g | 40.60428489 | HHX030011501.g | 1.580710222 |
| SSHX030030379.g | 2.692826778 | HHX030011502.g | 0.501256889 |
| SSHX030030402.g | 0.002959556 | HHX030011537.g | 0.023583667 |
| SSHX030030409.g | 0.329147111 | HHX030011540.g | 2.55111 |
| SSHX030030420.g | 0.002136667 | HHX030011546.g | 0.016041889 |
| SSHX030030422.g | 6.742212111 | HHX030011547.g | 0.136263222 |
| SSHX030030434.g | 0.155542 | HHX030011552.g | 5.388025556 |
| SSHX030030438.g | 0.068705889 | HHX030011553.g | 0.425134111 |
| SSHX030030464.g | 0.898725 | HHX030011577.g | 0.149703 |
| SSHX030030509.g | 0.165412556 | HHX030016260.g | 4.987540889 |
| SSHX030030512.g | 0.043993556 | HHX030016255.g | 0.745583556 |
| SSHX030030513.g | 1.927117 | HHX030016253.g | 0.054847333 |
| SSHX030030534.g | 0.085539556 | HHX020006649.g | 0.509137667 |
| SSHX030030561.g | 0.001951444 | HHX030016233.g | 8.77778E-05 |
| SSHX030030566.g | 3.217535889 | HHX030016230.g | 33.70090178 |
| SSHX030030587.g | 3.955986556 | HHX030016213.g | 0.050445778 |
| SSHX030030588.g | 3.368328778 | HHX030016212.g | 0.295171667 |
| SSHX030030601.g | 0.538060111 | HHX070056694.g | 7.145473556 |
| SSHX030030611.g | 0.404119667 | HHX030016186.g | 0.009737889 |
| SSHX030030615.g | 0.003914333 | HHX030016177.g | 0.020698556 |
| SSHX030030623.g | 28.299661 | HHX030016173.g | 3.485608333 |
| SSHX030030624.g | 0.197906444 | HHX070056673.g | 0.030910556 |
| SSHX030030625.g | 0.888722556 | HHX030016169.g | 6.550258667 |
| SSHX030030644.g | 8.026945556 | HHX030016146.g | 1.253229889 |
| SSHX030030648.g | 11.42303878 | HHX070056662.g | 1.935919667 |
| SSHX030030659.g | 16.524975 | HHX030016136.g | 133.4972914 |
| SSHX030030681.g | 3.154841333 | HHX030016120.g | 0.072966778 |
| SSHX030030705.g | 0.019213 | HHX030016079.g | 0.003616 |
| SSHX030030716.g | 12.41464633 | HHX030016073.g | 0.020737667 |
| SSHX030030727.g | 0.021011444 | HHX030016079.g | 0.003616 |
| SSHX030030733.g | 0.008180778 | HHX030016067.g | 1.396385889 |
| SSHX030030740.g | 0.072676222 | HHX030016069.g | 3.558071556 |
| SSHX030030741.g | 0.053273667 | HHX030016068.g | 1.940288 |
| SSHX030030790.g | 0.200640444 | HHX030016034.g | 1.441878111 |
| SSHX030030820.g | 23.40840111 | HHX030015990.g | 0.489964778 |
| SSHX030030861.g | 2.180034889 | HHX030015953.g | 0.064819444 |
| SSHX030030863.g | 1.129367444 | HHX030015943.g | 12.20899567 |
| SSHX030030872.g | 1.363711222 | HHX030015843.g | 13.03644822 |
| SSHX030030875.g | 0.883959778 | HHX030015858.g | 0.036711778 |
| SSHX030030886.g | 5.195166111 | HHX030015930.g | 0.180011111 |
| SSHX030030887.g | 3.640569111 | HHX030015932.g | 0.021300222 |
| SSHX030030888.g | 2.066709333 | HHX030015887.g | 0.376000333 |
| SSHX030030895.g | 0.240394556 | HHX030015917.g | 1.880739778 |
| SSHX030030912.g | 0.113897222 | HHX030015822.g | 4.798146333 |
| SSHX030030914.g | 1.426064222 | HHX030015898.g | 0.107651111 |
| SSHX030030925.g | 2.946492222 | HHX030015858.g | 0.036711778 |
| SSHX030030926.g | 0.026447778 | HHX030015815.g | 2.73727 |
| SSHX030030932.g | 0.105098444 | HHX030015879.g | 2.276330778 |
| SSHX030030947.g | 0.07275 | HHX030015884.g | 6.315573889 |
| SSHX030030950.g | 0.932062667 | HHX030015932.g | 0.021300222 |
| SSHX030030951.g | 0.424754667 | HHX030015843.g | 13.03644822 |
| SSHX030030960.g | 0.725661889 | HHX030015858.g | 0.036711778 |
| SSHX030030961.g | 0.001679778 | HHX030015884.g | 6.315573889 |
| SSHX030030963.g | 0.021932 | HHX030015843.g | 13.03644822 |
| SSHX030030969.g | 0.001499667 | HHX030015858.g | 0.036711778 |
| SSHX030030971.g | 0.019436889 | HHX030015873.g | 1.534997556 |
| SSHX030030972.g | 0.072132667 | HHX030015843.g | 13.03644822 |
| SSHX030030981.g | 0.286548222 | HHX030015810.g | 0.032598 |
| SSHX030030983.g | 0.049955222 | HHX030015884.g | 6.315573889 |
| SSHX030030987.g | 0.209790222 | HHX030015858.g | 0.036711778 |
| SSHX030030988.g | 0.053213667 | HHX030015899.g | 0.573809111 |
| SSHX030030990.g | 0.008182556 | HHX030015847.g | 0.290316667 |
| SSHX030030997.g | 0.087545444 | HHX030015869.g | 3.700240444 |
| SSHX030030998.g | 1.340639889 | HHX030015872.g | 0.030410333 |
| SSHX030030999.g | 7.419064222 | HHX030015931.g | 0.054091556 |
| SSHX030031013.g | 0.301987444 | HHX030015872.g | 0.030410333 |
| SSHX030031014.g | 5.938424222 | HHX030015931.g | 0.054091556 |
| SSHX030031017.g | 0.304873778 | HHX030015825.g | 1.954375333 |
| SSHX030031019.g | 0.053378 | HHX030015919.g | 3.037276556 |
| SSHX030031020.g | 0.330268333 | HHX030015933.g | 3.875419 |
| SSHX030031025.g | 0.003374 | HHX030015843.g | 13.03644822 |
| SSHX030031026.g | 0.187813667 | HHX030015886.g | 10.014699 |
| SSHX030031028.g | 0.398488111 | HHX030015872.g | 0.030410333 |
| SSHX030031032.g | 5.346167333 | HHX030015858.g | 0.036711778 |
| SSHX030031037.g | 0.108274111 | HHX030015800.g | 1.124424667 |
| SSHX030031088.g | 0.111904444 | HHX030015758.g | 6.398088 |
| SSHX030031106.g | 0.043519 | HHX030015731.g | 0.313382444 |
| SSHX030031107.g | 0.155153222 | HHX030015729.g | 0.012281 |
| SSHX030031113.g | 1.127132333 | HHX030015712.g | 15.28703578 |
| SSHX030031122.g | 0.009215111 | HHX030015696.g | 0.071894778 |
| SSHX030031126.g | 11.96075356 | HHX030015686.g | 0.533659667 |
| SSHX030031129.g | 0.330811667 | HHX030015684.g | 0.030247 |
| SSHX030031145.g | 0.005088333 | HHX030015656.g | 0.053451111 |
| SSHX030031165.g | 0.025972444 | HHX030015635.g | 0.001456667 |
| SSHX030031169.g | 1.632983 | HHX030015629.g | 16.72235289 |
| SSHX030031213.g | 1.990094111 | HHX030015529.g | 37.93429533 |
| SSHX030031214.g | 23.30530367 | HHX030015525.g | 0.442854889 |
| SSHX030031249.g | 190.1446691 | HHX030015501.g | 14.24886922 |
| SSHX030031272.g | 205.4058961 | HHX030015475.g | 25.38764767 |
| SSHX030031278.g | 43.956299 | HHX030015469.g | 0.350540778 |
| SSHX030031295.g | 21.36469633 | HHX030015449.g | 0.447903444 |
| SSHX030031297.g | 1.296562333 | HHX030015443.g | 0.048533778 |
| SSHX030031310.g | 1.197391111 | HHX030015436.g | 6.488000111 |
| SSHX030031324.g | 43.43884522 | HHX030015428.g | 0.338007333 |
| SSHX030031377.g | 0.288567444 | HHX090024773.g | 3.162917111 |
| SSHX030031396.g | 0.200706667 | HHX090024791.g | 0.033559 |
| SSHX030031401.g | 0.657485778 | HHX090024799.g | 9.305619444 |
| SSHX030031412.g | 2.494050667 | HHX090024820.g | 0.191119889 |
| SSHX030031414.g | 0.019142444 | HHX090024822.g | 0.446276222 |
| SSHX030031427.g | 0.078124889 | HHX090024825.g | 0.598485889 |
| SSHX030031434.g | 2.122747556 | HHX090024835.g | 0.086089222 |
| SSHX030031440.g | 0.224971444 | HHX090024847.g | 0.041402 |
| SSHX030031455.g | 0.076053444 | HHX040025646.g | 0.936775111 |
| SSHX030031482.g | 5.019256444 | HHX040025613.g | 0.070685556 |
| SSHX030031543.g | 9.127300444 | HHX040025571.g | 0.867594889 |
| SSHX030031559.g | 1.501201556 | HHX040025565.g | 0.167804667 |
| SSHX030031571.g | 0.925578222 | HHX040025550.g | 0.001774333 |
| SSHX030031634.g | 0.024180444 | HHX040025472.g | 0.001958 |
| SSHX030031657.g | 2.404250444 | HHX040025420.g | 0.255066556 |
| SSHX030031691.g | 0.043397889 | HHX040025296.g | 17.22136456 |
| SSHX030031695.g | 0.14842 | HHX040025285.g | 1.357203222 |
| SSHX030031704.g | 2.886554667 | HHX040025281.g | 0.021297333 |
| SSHX030031710.g | 0.615983556 | HHX040025275.g | 0.041398222 |
| SSHX030031895.g | 0.397648444 | HHX140040303.g | 44.91474311 |
| SSHX030031946.g | 5.326690889 | HHX140040264.g | 0.411480889 |
| SSHX030031949.g | 0.047992889 | HHX140040260.g | 13.872521 |
| SSHX030031956.g | 25.90211056 | HHX140040256.g | 1.056499778 |
| SSHX030031965.g | 11.23301911 | HHX140040243.g | 1.842641667 |
| SSHX030031979.g | 14.41404311 | HHX140040222.g | 1.239158889 |
| SSHX030031988.g | 1.179481889 | HHX140040216.g | 7.136696889 |
| SSHX030031993.g | 8.935921333 | HHX140040209.g | 0.479421889 |
| SSHX030031994.g | 1.298993889 | HHX140040208.g | 9.789756 |
| SSHX030032021.g | 0.007373111 | HHX140040185.g | 0.196159444 |
| SSHX030032024.g | 1.507368111 | HHX140040179.g | 11.28168778 |
| SSHX030032025.g | 0.148100667 | HHX140040178.g | 2.433471 |
| SSHX030032027.g | 0.0016 | HHX140040133.g | 4.219532111 |
| SSHX030032049.g | 168.8309467 | HHX140040152.g | 8.441346333 |
| SSHX030032060.g | 9.729826667 | HHX140040164.g | 0.573288 |
| SSHX030032064.g | 0.015215222 | HHX140040166.g | 0.464219333 |
| SSHX030032065.g | 0.047645111 | HHX140040169.g | 0.297532222 |
| SSHX030032066.g | 8.169289667 | HHX140040170.g | 1.287230667 |
| SSHX030032067.g | 41.36632267 | HHX140040173.g | 0.200161667 |
| SSHX030032075.g | 0.000734778 | HHX140040336.g | 0.090538 |
| SSHX030032077.g | 0.136409444 | HHX140040337.g | 0.022251111 |
| SSHX030032081.g | 33.70478533 | HHX140040341.g | 0.444032 |
| SSHX030032090.g | 40.95338333 | HHX140040350.g | 0.065100889 |
| SSHX030032139.g | 0.515974778 | HHX140040395.g | 0.023277 |
| SSHX030032140.g | 0.720201556 | HHX140040396.g | 0.021123667 |
| SSHX030032148.g | 0.037878889 | HHX140040402.g | 0.801039444 |
| SSHX030032154.g | 0.753897556 | HHX140040407.g | 0.002661444 |
| SSHX030032156.g | 0.473822111 | HHX140040409.g | 8.003682333 |
| SSHX030032166.g | 0.009538556 | HHX140040413.g | 0.083247222 |
| SSHX030032214.g | 0.017529444 | HHX140040441.g | 0.102916778 |
| SSHX030032228.g | 0.442613444 | HHX140040458.g | 0.016516111 |
| SSHX030032245.g | 0.168462444 | HHX140040476.g | 1.101189111 |
| SSHX030032249.g | 4.029370889 | HHX140040479.g | 35.98768344 |
| SSHX030032255.g | 10.43366744 | HHX140040482.g | 1.122367778 |
| SSHX030032257.g | 14.95769433 | HHX140040483.g | 0.292337 |
| SSHX030032269.g | 0.256386667 | HHX100023378.g | 3.346670333 |
| SSHX030032273.g | 0.042474889 | HHX100023393.g | 5.511328556 |
| SSHX030032274.g | 0.024336667 | HHX100023394.g | 0.503719667 |
| SSHX030032297.g | 6.839952889 | HHX140040514.g | 0.223531222 |
| SSHX030032299.g | 0.028136778 | HHX140040517.g | 0.469997778 |
| SSHX030032307.g | 2.871849667 | HHX140040523.g | 31.02194322 |
| SSHX030032308.g | 27.65473133 | HHX140040524.g | 1.018939 |
| SSHX030032359.g | 0.023960222 | HHX140040550.g | 0.003978444 |
| SSHX030032363.g | 6.462503444 | HHX140040555.g | 0.029330222 |
| SSHX030032371.g | 0.419772111 | HHX140040562.g | 29.76038311 |
| SSHX030032385.g | 0.152152444 | HHX140040573.g | 0.959335889 |
| SSHX030032387.g | 1.823935667 | HHX140040574.g | 0.097851333 |
| SSHX030032390.g | 0.036596111 | HHX140040582.g | 0.677984556 |
| SSHX030032413.g | 0.229175778 | HHX140040598.g | 2.863044444 |
| SSHX030032414.g | 0.656093556 | HHX140040600.g | 16.29946033 |
| SSHX030032425.g | 55.90095911 | HHX100020411.g | 0.168546222 |
| SSHX030032430.g | 0.203160222 | HHX100020405.g | 2.557925444 |
| SSHX030032436.g | 6.037645778 | HHX100020396.g | 0.272231 |
| SSHX030032437.g | 0.031413667 | HHX140040602.g | 0.210442778 |
| SSHX030032442.g | 0.005797222 | HHX140040607.g | 0.117958222 |
| SSHX030032469.g | 0.012343778 | HHX140040630.g | 0.908717889 |
| SSHX030032481.g | 131.4493433 | HHX020007789.g | 25.22331156 |
| SSHX030032489.g | 0.026168 | HHX020007783.g | 18.979596 |
| SSHX030032490.g | 4.904959556 | HHX140040658.g | 0.897231778 |
| SSHX030032508.g | 24.52024111 | HHX020007774.g | 1.030096889 |
| SSHX030032516.g | 0.082523556 | HHX140040682.g | 6.584345222 |
| SSHX030032520.g | 2.556689 | HHX140040690.g | 41.34297744 |
| SSHX030032533.g | 0.178440111 | HHX020007745.g | 83.97004311 |
| SSHX030032540.g | 0.012851 | HHX140040712.g | 0.137238333 |
| SSHX030032547.g | 0.220436444 | HHX140040729.g | 2.411028333 |
| SSHX030032555.g | 8.728987667 | HHX140040728.g | 0.057260556 |
| SSHX030032559.g | 2.839529111 | HHX140040811.g | 0.005464222 |
| SSHX030032560.g | 0.013970556 | HHX140040734.g | 0.094551 |
| SSHX030032574.g | 0.127247556 | HHX140040744.g | 0.958820667 |
| SSHX030032576.g | 7.487191556 | HHX140040746.g | 0.482958222 |
| SSHX030032581.g | 0.077271333 | HHX140040752.g | 2.958193333 |
| SSHX030032592.g | 0.254060444 | HHX140040771.g | 14.37972844 |
| SSHX030032600.g | 1.674732556 | HHX140040776.g | 0.160563 |
| SSHX030032656.g | 0.986098667 | HHX140040777.g | 5.125314556 |
| SSHX030032659.g | 0.260765444 | HHX140040780.g | 1.863124333 |
| SSHX030032675.g | 0.500790111 | HHX140040835.g | 6.945261111 |
| SSHX030032676.g | 0.305969444 | HHX140040837.g | 6.114692 |
| SSHX030032685.g | 33.54946444 | HHX140040845.g | 1.288574778 |
| SSHX030032686.g | 1.491978111 | HHX140040846.g | 10.19501322 |
| SSHX030032687.g | 0.127193556 | HHX140040847.g | 2.160421222 |
| SSHX030032689.g | 3.636968333 | HHX140040803.g | 79.54755033 |
| SSHX030032694.g | 1.205309 | HHX140040855.g | 0.083667778 |
| SSHX030032704.g | 0.045126444 | HHX140040865.g | 2.832234556 |
| SSHX030032705.g | 14.372852 | HHX140040909.g | 208.4535183 |
| SSHX030032711.g | 1.036832778 | HHX140040871.g | 43.02838167 |
| SSHX030032714.g | 48.25805922 | HHX140040876.g | 5.766825556 |
| SSHX030032722.g | 8.11218 | HHX140040890.g | 0.262837889 |
| SSHX030032745.g | 0.427150889 | HHX140040893.g | 7.790950111 |
| SSHX030032755.g | 0.060772222 | HHX140040899.g | 6.908694111 |
| SSHX030032757.g | 17.10802433 | HHX140040901.g | 1.096934111 |
| SSHX030032761.g | 0.010918556 | HHX140040906.g | 0.153064444 |
| SSHX030032786.g | 0.052447222 | HHX140040933.g | 0.272047667 |
| SSHX030032793.g | 1.229995889 | HHX140040939.g | 18.95945833 |
| SSHX030032799.g | 0.333825333 | HHX140040941.g | 2.744455778 |
| SSHX030032803.g | 0.015422 | HHX140040947.g | 0.191549222 |
| SSHX030032808.g | 0.538325222 | HHX140040953.g | 6.238195222 |
| SSHX030032812.g | 0.912581 | HHX140040956.g | 5.984643556 |
| SSHX030032828.g | 59.08921211 | HHX140040965.g | 8.792010333 |
| SSHX030032831.g | 0.001882111 | HHX140040967.g | 0.080600889 |
| SSHX030032842.g | 2.713012556 | HHX140040976.g | 0.183622667 |
| SSHX030032845.g | 0.229740778 | HHX140040977.g | 0.020538667 |
| SSHX030032855.g | 17.68840767 | HHX140040985.g | 0.230916333 |
| SSHX030032882.g | 0.044171889 | HHX020007504.g | 2.205314333 |
| SSHX030032883.g | 5.661793444 | HHX020007503.g | 51.43424911 |
| SSHX030032884.g | 0.755636444 | HHX020007498.g | 0.008358 |
| SSHX030032887.g | 3.622097222 | HHX140040996.g | 19.084846 |
| SSHX030032894.g | 1.111017444 | HHX140040998.g | 10.99473722 |
| SSHX030032896.g | 53.35602589 | HHX020007495.g | 5.998026444 |
| SSHX030032907.g | 79.87581678 | HHX140041009.g | 0.830756111 |
| SSHX030032915.g | 0.498599444 | HHX140041012.g | 17.39243933 |
| SSHX030032921.g | 6.454512222 | HHX140041018.g | 0.582511889 |
| SSHX030032931.g | 9.286744333 | HHX020007435.g | 1.589288889 |
| SSHX030032941.g | 20.88282822 | HHX020007412.g | 2.182022556 |
| SSHX030032942.g | 0.247611111 | HHX020007401.g | 6.088344333 |
| SSHX030032943.g | 0.049346111 | HHX140041037.g | 0.306544222 |
| SSHX030032954.g | 0.180398 | HHX020007380.g | 3.506632333 |
| SSHX030032958.g | 0.458976 | HHX140041049.g | 0.080213556 |
| SSHX030032966.g | 0.030625667 | HHX140041056.g | 0.218395111 |
| SSHX030032967.g | 0.114560778 | HHX140041057.g | 1.072145778 |
| SSHX030032968.g | 2.775273889 | HHX140041058.g | 0.480788889 |
| SSHX030032975.g | 4.949154556 | HHX140041065.g | 0.234577222 |
| SSHX030032978.g | 0.249926778 | HHX140041069.g | 0.049223778 |
| SSHX030033001.g | 0.293691556 | HHX140041070.g | 0.028396222 |
| SSHX030033004.g | 0.128401667 | HHX140041123.g | 0.017570222 |
| SSHX030033029.g | 0.007811222 | HHX140041109.g | 0.075649222 |
| SSHX030033034.g | 0.591300222 | HHX140041136.g | 0.032094889 |
| SSHX030033038.g | 108.0946731 | HHX140041140.g | 1.776487 |
| SSHX030033040.g | 0.356323333 | HHX140041142.g | 4.231725111 |
| SSHX030033050.g | 8.334496333 | HHX020006731.g | 0.145749667 |
| SSHX030033056.g | 0.009905778 | HHX020006740.g | 4.494717222 |
| SSHX030033057.g | 0.466502222 | HHX020006741.g | 6.605530889 |
| SSHX030033058.g | 0.227020222 | HHX020006742.g | 22.53071867 |
| SSHX030033064.g | 0.927761889 | HHX140041148.g | 7.121857778 |
| SSHX030033066.g | 0.070456778 | HHX140041151.g | 0.446646778 |
| SSHX030033068.g | 2.935076667 | HHX140041155.g | 98.95981767 |
| SSHX030033069.g | 0.812351 | HHX140041156.g | 4.201297778 |
| SSHX030033070.g | 3.161565889 | HHX020006746.g | 0.036407222 |
| SSHX030033072.g | 6.287195333 | HHX140041160.g | 1.028811889 |
| SSHX030033074.g | 1.346211 | HHX140041162.g | 11.30551011 |
| SSHX030033075.g | 7.305169667 | HHX140041164.g | 51.10926944 |
| SSHX040003343.g | 0.010614 | HHX110051945.g | 4.773058556 |
| SSHX040003349.g | 0.113625222 | HHX110051938.g | 4.068770444 |
| SSHX040003358.g | 0.201086333 | HHX110051893.g | 0.009153222 |
| SSHX040003363.g | 0.024606778 | HHX110051901.g | 0.401610556 |
| SSHX040003382.g | 6.932854333 | HHX110051924.g | 0.053138667 |
| SSHX040003392.g | 19.62174789 | HHX110051915.g | 0.902796111 |
| SSHX040003433.g | 0.062956222 | HHX110051866.g | 0.367607778 |
| SSHX040003460.g | 0.054419778 | HHX110051845.g | 0.569155889 |
| SSHX040003472.g | 0.349409889 | HHX110051800.g | 21.71809011 |
| SSHX040003506.g | 0.201990556 | HHX110051779.g | 2.018317 |
| SSHX040003510.g | 0.142680333 | HHX110051774.g | 2.051926778 |
| SSHX040003515.g | 0.180207111 | HHX110051763.g | 0.030821222 |
| SSHX040003521.g | 0.421785556 | HHX110051750.g | 9.711837556 |
| SSHX040003526.g | 283.2703486 | HHX110051737.g | 7.407926889 |
| SSHX040003528.g | 0.153359111 | HHX110051742.g | 324.4156522 |
| SSHX040003529.g | 3.567817889 | HHX110051741.g | 231.11883 |
| SSHX040003534.g | 11.98926267 | HHX110051718.g | 0.458873333 |
| SSHX040003548.g | 0.024595333 | HHX110051722.g | 0.768439111 |
| SSHX040003556.g | 92.06826622 | HHX110051718.g | 0.458873333 |
| SSHX040003575.g | 0.046222556 | HHX110051682.g | 0.567146667 |
| SSHX040003604.g | 2.315831222 | HHX110051640.g | 0.038287222 |
| SSHX040003611.g | 7.643031333 | HHX110051654.g | 0.529912222 |
| SSHX040003615.g | 1.549013222 | HHX110051660.g | 20.59998422 |
| SSHX040003644.g | 0.061651556 | HHX110051492.g | 0.004611889 |
| SSHX040003655.g | 0.009056111 | HHX110051497.g | 0.000651333 |
| SSHX040003656.g | 0.280498556 | HHX110051500.g | 0.039712889 |
| SSHX040003662.g | 0.086418 | HHX110051492.g | 0.004611889 |
| SSHX040003712.g | 64.37838078 | HHX110051411.g | 2.646248 |
| SSHX040003720.g | 2.104856 | HHX110051410.g | 11.09140089 |
| SSHX040003750.g | 0.957229222 | HHX110051394.g | 0.152625444 |
| SSHX040003758.g | 1.362712556 | HHX110051387.g | 0.088970111 |
| SSHX040003759.g | 53.49482011 | HHX110051386.g | 0.282074556 |
| SSHX040003760.g | 0.455317889 | HHX110051385.g | 43.10822711 |
| SSHX040003765.g | 4.299688 | HHX110051383.g | 106.9132967 |
| SSHX040003767.g | 54.95903956 | HHX110051381.g | 0.158049 |
| SSHX040003816.g | 1.002493111 | HHX110051343.g | 0.168432 |
| SSHX040003824.g | 0.113421667 | HHX110051333.g | 0.016039444 |
| SSHX040003835.g | 0.266601111 | HHX110051325.g | 0.048799444 |
| SSHX040003854.g | 0.149466 | HHX110051300.g | 3.46635 |
| SSHX040003856.g | 34.42395233 | HHX110051298.g | 3.320476 |
| SSHX040003867.g | 3.613458444 | HHX110051283.g | 50.20824833 |
| SSHX040003874.g | 0.003175111 | HHX110051274.g | 0.302997 |
| SSHX040003877.g | 28.87481289 | HHX110051272.g | 1.725045444 |
| SSHX040003887.g | 0.000269778 | HHX110051268.g | 0.025374556 |
| SSHX040003907.g | 0.811013778 | HHX110051245.g | 16.40963411 |
| SSHX040003911.g | 0.095507222 | HHX110051240.g | 2.824584333 |
| SSHX040003931.g | 13.95582922 | HHX110051218.g | 2.052763778 |
| SSHX040003933.g | 1.961927444 | HHX110051216.g | 14.71588189 |
| SSHX040003954.g | 0.011391444 | HHX110051203.g | 1.529649556 |
| SSHX040003972.g | 52.305166 | HHX110051198.g | 5.247549333 |
| SSHX040003980.g | 0.430719222 | HHX110051194.g | 0.008652444 |
| SSHX040003986.g | 0.095510778 | HHX110051189.g | 0.698095444 |
| SSHX040003987.g | 0.132477111 | HHX110051188.g | 1.158058667 |
| SSHX040003991.g | 0.017062889 | HHX110051182.g | 0.216455444 |
| SSHX040003993.g | 0.119656333 | HHX110051179.g | 0.708742556 |
| SSHX040003998.g | 0.207433111 | HHX110051172.g | 358.9918061 |
| SSHX040004000.g | 15.36029478 | HHX110051171.g | 2.911339444 |
| SSHX040004006.g | 0.101338111 | HHX110051164.g | 0.512491667 |
| SSHX040004008.g | 2.775604556 | HHX110051132.g | 0.494708667 |
| SSHX040004041.g | 0.000308444 | HHX110051060.g | 0.002255333 |
| SSHX040004049.g | 0.220694 | HHX110051070.g | 0.008085556 |
| SSHX040004071.g | 0.015650333 | HHX110051091.g | 6.144086667 |
| SSHX040004078.g | 0.000787778 | HHX110051100.g | 0.021764333 |
| SSHX040004085.g | 0.014216 | HHX110051090.g | 10.57947489 |
| SSHX040004088.g | 0.236857333 | HHX110051091.g | 6.144086667 |
| SSHX040004093.g | 0.290041 | HHX110051033.g | 1.566844667 |
| SSHX040004096.g | 0.162892778 | HHX110051030.g | 8.748106111 |
| SSHX040004105.g | 0.310353333 | HHX110051022.g | 0.028470889 |
| SSHX040004118.g | 0.040989556 | HHX110050973.g | 0.006910556 |
| SSHX040004126.g | 0.002424444 | HHX110051007.g | 0.024217444 |
| SSHX040004140.g | 0.008596667 | HHX110050936.g | 0.053576 |
| SSHX040004160.g | 0.209213444 | HHX110050922.g | 1.394213667 |
| SSHX040004168.g | 0.418636778 | HHX110050915.g | 10.65911533 |
| SSHX040004187.g | 0.019951889 | HHX110050909.g | 2.587546889 |
| SSHX040004211.g | 0.041224556 | HHX110050898.g | 1.041498444 |
| SSHX040004212.g | 0.035098778 | HHX110050897.g | 0.260790444 |
| SSHX040004229.g | 0.139110444 | HHX110050883.g | 0.011493222 |
| SSHX040004239.g | 0.183422222 | HHX110050871.g | 4.801395889 |
| SSHX040004251.g | 31.93959811 | HHX110050857.g | 385.4140507 |
| SSHX040004254.g | 1.291188 | HHX110050852.g | 30.08752333 |
| SSHX040004278.g | 1.50339 | HHX110050815.g | 12.32452444 |
| SSHX040004286.g | 1.751869222 | HHX110050811.g | 0.018651556 |
| SSHX040004312.g | 3.261801667 | HHX110050787.g | 0.291143444 |
| SSHX040004324.g | 22.31226078 | HHX110050782.g | 0.453050333 |
| SSHX040004328.g | 0.012249889 | HHX110050780.g | 0.000481889 |
| SSHX040004335.g | 1.162439889 | HHX110050771.g | 56.64213044 |
| SSHX040004342.g | 36.16054611 | HHX110050765.g | 1.857846333 |
| SSHX040004348.g | 0.030356444 | HHX110050761.g | 0.878460111 |
| SSHX040004355.g | 1.656294333 | HHX110050756.g | 17.05882567 |
| SSHX040004382.g | 0.000794333 | HHX110050708.g | 1.521301333 |
| SSHX040004425.g | 0.341568333 | HHX110050607.g | 2.405752778 |
| SSHX040004441.g | 0.152602889 | HHX110050611.g | 2.910261778 |
| SSHX040004462.g | 0.008057667 | HHX110050670.g | 0.162910333 |
| SSHX040004467.g | 0.090028667 | HHX110050690.g | 0.685594556 |
| SSHX040004475.g | 3.734483778 | HHX110050697.g | 0.003852333 |
| SSHX040004489.g | 0.580377333 | HHX110050672.g | 0.103201 |
| SSHX040004493.g | 0.042022667 | HHX110050685.g | 0.224099222 |
| SSHX040004494.g | 0.003179556 | HHX110050684.g | 0.040112667 |
| SSHX040004503.g | 0.007432444 | HHX110050661.g | 0.300430667 |
| SSHX040004514.g | 0.085152444 | HHX110050697.g | 0.003852333 |
| SSHX040004523.g | 13.71965511 | HHX110050736.g | 0.822600778 |
| SSHX040004525.g | 0.947767222 | HHX110050672.g | 0.103201 |
| SSHX040004530.g | 0.042282889 | HHX110050697.g | 0.003852333 |
| SSHX040004545.g | 0.168651667 | HHX110050698.g | 1.976205667 |
| SSHX040004558.g | 1.661296333 | HHX110050674.g | 0.312673667 |
| SSHX040004574.g | 0.101872667 | HHX110050703.g | 6.790562556 |
| SSHX040004609.g | 0.013917556 | HHX110050614.g | 0.242677111 |
| SSHX040004631.g | 6.646758667 | HHX110050600.g | 0.408480667 |
| SSHX040004649.g | 0.031698778 | HHX110050558.g | 0.939118778 |
| SSHX040004676.g | 0.022064778 | HHX110050541.g | 2.902304667 |
| SSHX040004681.g | 6.172351333 | HHX110050535.g | 32.43968178 |
| SSHX040004687.g | 0.026546111 | HHX110050516.g | 1.062829556 |
| SSHX040004704.g | 13.855031 | HHX110050526.g | 674.6837541 |
| SSHX040004718.g | 0.087963222 | HHX110050516.g | 1.062829556 |
| SSHX040004731.g | 1.138408 | HHX110050504.g | 0.059816222 |
| SSHX040004732.g | 1.064117667 | HHX110050505.g | 15.23951078 |
| SSHX040004734.g | 0.103569667 | HHX050046954.g | 1.386793333 |
| SSHX040004748.g | 4.035409333 | HHX050046945.g | 31.75425589 |
| SSHX040004750.g | 5.667567778 | HHX110050504.g | 0.059816222 |
| SSHX040004800.g | 0.136232444 | HHX110050498.g | 0.953268889 |
| SSHX040004813.g | 0.029615333 | HHX110050486.g | 1.874247222 |
| SSHX040004814.g | 0.050624111 | HHX110050485.g | 0.432114 |
| SSHX040004838.g | 0.178797444 | HHX110050444.g | 15.65372167 |
| SSHX040004851.g | 0.436714444 | HHX110050433.g | 27.028369 |
| SSHX040004852.g | 32.61340567 | HHX110050453.g | 2.287230889 |
| SSHX040004853.g | 2.088736333 | HHX110050447.g | 0.011753778 |
| SSHX040004874.g | 21.60256833 | HHX110050470.g | 0.149159889 |
| SSHX040004900.g | 1.294938444 | HHX110050412.g | 7.936508111 |
| SSHX040004903.g | 0.229977222 | HHX110050409.g | 15.07850622 |
| SSHX040004910.g | 0.008249 | HHX110050398.g | 0.001526222 |
| SSHX040004914.g | 0.492151333 | HHX110050396.g | 12.98516478 |
| SSHX040004964.g | 0.806116111 | HHX110050378.g | 0.134677667 |
| SSHX040004992.g | 0.005439778 | HHX110050344.g | 2.596605333 |
| SSHX040004994.g | 0.029584444 | HHX110050349.g | 0.321426889 |
| SSHX040005010.g | 0.484415 | HHX110050358.g | 0.095124556 |
| SSHX040005042.g | 0.047663222 | HHX010038301.g | 0.251753222 |
| SSHX040005045.g | 21.00757978 | HHX010038297.g | 3.227774889 |
| SSHX040005060.g | 34.29304989 | HHX010038291.g | 0.92173 |
| SSHX040005065.g | 36.50990933 | HHX010038278.g | 0.17612 |
| SSHX040005118.g | 0.006268222 | HHX050049189.g | 0.042189222 |
| SSHX040005123.g | 108.6639648 | HHX050049187.g | 0.015674667 |
| SSHX040005124.g | 1.140311889 | HHX050049186.g | 0.009758222 |
| SSHX040005142.g | 0.350248444 | HHX050049155.g | 2.547770111 |
| SSHX040005146.g | 0.044678111 | HHX050049152.g | 0.004769556 |
| SSHX040005160.g | 0.473628111 | HHX050049150.g | 17.25520822 |
| SSHX040005162.g | 19.72644789 | HHX050049145.g | 0.244382222 |
| SSHX040005166.g | 2.172778 | HHX050049141.g | 0.210474667 |
| SSHX040005170.g | 9.179077556 | HHX050049140.g | 1.123541889 |
| SSHX040005172.g | 0.036951444 | HHX050049138.g | 0.227729 |
| SSHX040005211.g | 17.98142422 | HHX050049125.g | 3.261225889 |
| SSHX040005212.g | 0.521649111 | HHX050049124.g | 3.414704333 |
| SSHX040005214.g | 1.18399 | HHX050049114.g | 12.64159111 |
| SSHX040005223.g | 16.12615122 | HHX050049113.g | 0.515063778 |
| SSHX040005228.g | 2.442689556 | HHX050049112.g | 0.274086111 |
| SSHX040005237.g | 1.233007778 | HHX050049107.g | 6.469387444 |
| SSHX040005279.g | 6.467654889 | HHX110052984.g | 0.855628889 |
| SSHX040005289.g | 29.89633644 | HHX110052975.g | 0.855103778 |
| SSHX040005299.g | 0.059211444 | HHX110052964.g | 0.354432444 |
| SSHX040005301.g | 0.767185 | HHX110052959.g | 0.071123444 |
| SSHX040005303.g | 0.425433222 | HHX110052956.g | 0.058464889 |
| SSHX040005305.g | 0.014108556 | HHX110052948.g | 0.621893444 |
| SSHX040005331.g | 432.1332159 | HHX110052917.g | 4.818069667 |
| SSHX040005332.g | 247.9198923 | HHX110052916.g | 4.273276444 |
| SSHX040005342.g | 353.5302972 | HHX050049031.g | 35.87961678 |
| SSHX040005349.g | 0.738846556 | HHX110052904.g | 6.693729889 |
| SSHX040005357.g | 0.651206444 | HHX050049025.g | 6.392103889 |
| SSHX040005364.g | 2.923240778 | HHX110052902.g | 0.164021111 |
| SSHX040005365.g | 0.05386 | HHX050049022.g | 0.785415333 |
| SSHX040005367.g | 0.034882444 | HHX110052901.g | 0.940929778 |
| SSHX040005369.g | 1.774088556 | HHX110052899.g | 0.131527556 |
| SSHX040005373.g | 8.377500889 | HHX110052898.g | 1.530210333 |
| SSHX040005382.g | 14.99893278 | HHX050049017.g | 0.011958 |
| SSHX040005390.g | 0.184016222 | HHX050049008.g | 1.283807444 |
| SSHX040005399.g | 10.63084078 | HHX110052886.g | 0.116116111 |
| SSHX040005408.g | 13.56114256 | HHX110052880.g | 0.292203111 |
| SSHX040005409.g | 1.570631444 | HHX050048990.g | 0.194019222 |
| SSHX040005424.g | 7.749759111 | HHX110052872.g | 60.42820833 |
| SSHX040005425.g | 29.58309389 | HHX050048975.g | 164.3393682 |
| SSHX040005432.g | 0.776221556 | HHX110052859.g | 4.157957333 |
| SSHX040005433.g | 58.42079422 | HHX050048967.g | 0.662577889 |
| SSHX040005437.g | 10.71680067 | HHX110052852.g | 1.178282667 |
| SSHX040005443.g | 2.010456111 | HHX110052845.g | 0.366891111 |
| SSHX040005451.g | 48.024981 | HHX110052829.g | 3.087067778 |
| SSHX040005471.g | 0.032309889 | HHX110052816.g | 0.505657556 |
| SSHX040005501.g | 0.054055222 | HHX110052789.g | 0.311846667 |
| SSHX040005506.g | 0.503327222 | HHX110052739.g | 0.059991222 |
| SSHX040005508.g | 0.036530556 | HHX110052750.g | 0.453144 |
| SSHX040005512.g | 0.016062889 | HHX110052738.g | 0.146065333 |
| SSHX040005516.g | 1.377888444 | HHX050048680.g | 0.178974667 |
| SSHX040005520.g | 0.027920333 | HHX110052775.g | 0.227800778 |
| SSHX040005521.g | 0.322362778 | HHX050048777.g | 3.208835556 |
| SSHX040005527.g | 14.64954811 | HHX050048705.g | 2.325711556 |
| SSHX040005531.g | 1.092520556 | HHX050048720.g | 0.047681556 |
| SSHX040005532.g | 0.014242444 | HHX050048686.g | 0.336006667 |
| SSHX040005534.g | 0.107739444 | HHX050048777.g | 3.208835556 |
| SSHX040005535.g | 0.048582333 | HHX050048745.g | 6.420230333 |
| SSHX040005539.g | 1.678441667 | HHX110052737.g | 0.125461333 |
| SSHX040005545.g | 0.153183222 | HHX110052731.g | 1.446335556 |
| SSHX040005584.g | 15.91085456 | HHX110052716.g | 0.031964889 |
| SSHX040005591.g | 22.76174067 | HHX110052711.g | 2.39333 |
| SSHX040005593.g | 0.994513778 | HHX110052709.g | 17.87612244 |
| SSHX040005605.g | 0.020493444 | HHX110052694.g | 0.191311333 |
| SSHX040005661.g | 6.940480778 | HHX110052670.g | 0.031476222 |
| SSHX040005664.g | 7.098997333 | HHX110052669.g | 1.095368222 |
| SSHX040005675.g | 1.585434667 | HHX110052660.g | 8.480185111 |
| SSHX040005676.g | 0.514570889 | HHX110052658.g | 3.105260222 |
| SSHX040005694.g | 0.595778111 | HHX110052648.g | 22.78126556 |
| SSHX040005701.g | 3.940511889 | HHX110052642.g | 0.089441556 |
| SSHX040005704.g | 0.073396778 | HHX110052641.g | 0.461255444 |
| SSHX040005707.g | 6.130968889 | HHX110052640.g | 1.076933889 |
| SSHX040005716.g | 0.345594444 | HHX110052636.g | 534.7103643 |
| SSHX040005719.g | 14.22751044 | HHX050048649.g | 1.095649222 |
| SSHX040005729.g | 2.225591556 | HHX050048645.g | 53.24649922 |
| SSHX040005730.g | 15.27977578 | HHX050048643.g | 2.499409889 |
| SSHX040005742.g | 267.956104 | HHX050048637.g | 1.807584556 |
| SSHX040005755.g | 0.644761111 | HHX110052612.g | 22.14370044 |
| SSHX040005776.g | 12.75017344 | HHX110052593.g | 1.833686556 |
| SSHX040005778.g | 0.132938889 | HHX110052590.g | 16.48907133 |
| SSHX040005792.g | 78.14492644 | HHX110052578.g | 8.020379444 |
| SSHX040005795.g | 0.759271111 | HHX110052577.g | 524.5911154 |
| SSHX040005796.g | 0.120428667 | HHX110052568.g | 0.004761556 |
| SSHX040005802.g | 3.105639889 | HHX110052565.g | 0.226621889 |
| SSHX040005810.g | 2.002255889 | HHX110052558.g | 12.71622 |
| SSHX040005812.g | 0.027403889 | HHX110052568.g | 0.004761556 |
| SSHX040005825.g | 0.369551444 | HHX110052542.g | 4.625035778 |
| SSHX040005833.g | 0.863865 | HHX110052544.g | 0.125625222 |
| SSHX040005836.g | 0.095799 | HHX110052541.g | 0.697339333 |
| SSHX040005842.g | 4.063703889 | HHX110052536.g | 0.265402778 |
| SSHX040005900.g | 0.093944333 | HHX110052489.g | 0.964820222 |
| SSHX040005901.g | 0.008223444 | HHX110052488.g | 0.344816889 |
| SSHX040005902.g | 0.018309222 | HHX110052487.g | 0.665960111 |
| SSHX040005911.g | 0.025248444 | HHX110052477.g | 0.908004 |
| SSHX040005914.g | 0.228572111 | HHX050048559.g | 14.60792333 |
| SSHX040005919.g | 0.739045778 | HHX050048547.g | 0.003909889 |
| SSHX040005920.g | 0.019568333 | HHX110052462.g | 2.228634778 |
| SSHX040005930.g | 41.38406589 | HHX110052453.g | 0.162613333 |
| SSHX040005933.g | 0.024327778 | HHX110052451.g | 0.401949 |
| SSHX040005935.g | 4.831442444 | HHX110052447.g | 0.478945778 |
| SSHX040005939.g | 0.107459 | HHX110052445.g | 1.144512889 |
| SSHX040005942.g | 8.768817778 | HHX110052442.g | 0.666470889 |
| SSHX040005944.g | 0.559315778 | HHX110052440.g | 0.055683556 |
| SSHX040005945.g | 0.169866444 | HHX050048538.g | 0.013077889 |
| SSHX040005946.g | 29.66474867 | HHX110052439.g | 2.274658444 |
| SSHX040005962.g | 0.475755889 | HHX110052430.g | 0.013991556 |
| SSHX040005964.g | 4.186894 | HHX050048532.g | 0.615935 |
| SSHX040005969.g | 0.014498778 | HHX050048527.g | 0.244793556 |
| SSHX040005971.g | 1.188005444 | HHX050048526.g | 6.134277333 |
| SSHX040005983.g | 0.008695 | HHX110052413.g | 0.057210778 |
| SSHX040005986.g | 1.767885444 | HHX110052417.g | 0.029673556 |
| SSHX040005993.g | 0.451987111 | HHX110052413.g | 0.057210778 |
| SSHX040006001.g | 0.001240111 | HHX110052399.g | 1.338650889 |
| SSHX040006076.g | 0.027896 | HHX110052331.g | 0.000304778 |
| SSHX040006104.g | 0.007113778 | HHX110052307.g | 0.095884 |
| SSHX040006107.g | 176.4929487 | HHX110052306.g | 0.239021889 |
| SSHX040006113.g | 0.010921556 | HHX050048401.g | 181.499694 |
| SSHX040006131.g | 5.249567667 | HHX050048379.g | 0.012653333 |
| SSHX040006132.g | 0.105455667 | HHX110052285.g | 0.540944111 |
| SSHX040006133.g | 0.190664333 | HHX110052284.g | 4.418354 |
| SSHX040006140.g | 7.078001 | HHX050048349.g | 0.795368889 |
| SSHX040006161.g | 22.84916344 | HHX050048344.g | 0.176691 |
| SSHX040006178.g | 45.21248367 | HHX050048318.g | 1.858784667 |
| SSHX040006180.g | 0.189508111 | HHX110052254.g | 2.001575222 |
| SSHX040006190.g | 0.178530556 | HHX110052244.g | 7.490041111 |
| SSHX040006191.g | 0.079783889 | HHX110052243.g | 1.460116444 |
| SSHX040006195.g | 4.006046444 | HHX050048295.g | 73.43612967 |
| SSHX040006200.g | 0.159971667 | HHX110052237.g | 1.099194 |
| SSHX040006208.g | 3.121569222 | HHX110052229.g | 20.78337033 |
| SSHX040006219.g | 91.32093767 | HHX110052224.g | 4.985825 |
| SSHX040006228.g | 0.543152111 | HHX110052215.g | 4.546084778 |
| SSHX040006245.g | 0.250001222 | HHX110052201.g | 1.841430111 |
| SSHX040006296.g | 0.180887222 | HHX110052162.g | 2.125158889 |
| SSHX040006309.g | 1.651346222 | HHX110052135.g | 21.94428267 |
| SSHX040006317.g | 3.763976 | HHX110052126.g | 0.354853667 |
| SSHX040006318.g | 18.41381856 | HHX110052123.g | 0.615912222 |
| SSHX040006347.g | 0.187466556 | HHX110052098.g | 0.014438 |
| SSHX040006356.g | 0.318225333 | HHX110052088.g | 15.55353211 |
| SSHX040006384.g | 0.447929889 | HHX110052069.g | 2.783316 |
| SSHX040006398.g | 0.17057 | HHX110052047.g | 16.29822733 |
| SSHX040006414.g | 1.482770889 | HHX110052033.g | 0.008889889 |
| SSHX040006422.g | 4.380518333 | HHX110052021.g | 0.587481333 |
| SSHX040006450.g | 0.020577333 | HHX110051999.g | 0.00372 |
| SSHX040006462.g | 1.777624778 | HHX110051969.g | 11.797017 |
| SSHX040006517.g | 0.018545667 | HHX140041848.g | 0.487814 |
| SSHX040006527.g | 0.374352222 | HHX140041835.g | 20.50113367 |
| SSHX040006555.g | 28.80238967 | HHX140041786.g | 3.519071667 |
| SSHX040006561.g | 0.157120444 | HHX030011773.g | 84.71513322 |
| SSHX040006565.g | 0.425426333 | HHX030011774.g | 2.509165111 |
| SSHX040006573.g | 0.001834889 | HHX140041775.g | 0.020007667 |
| SSHX040006584.g | 27.36936533 | HHX140041766.g | 1.972324778 |
| SSHX040006599.g | 0.785913111 | HHX140041754.g | 0.030931667 |
| SSHX040006608.g | 0.574944111 | HHX140041739.g | 22.20456867 |
| SSHX040006620.g | 20.31048033 | HHX140041730.g | 123.2487936 |
| SSHX040006625.g | 0.091058222 | HHX020009995.g | 0.483251667 |
| SSHX040006653.g | 0.208719667 | HHX020010021.g | 0.039509222 |
| SSHX040006671.g | 0.019778222 | HHX020010010.g | 1.487165444 |
| SSHX040006673.g | 0.001176778 | HHX020010026.g | 0.023976444 |
| SSHX040006674.g | 0.801758667 | HHX020010021.g | 0.039509222 |
| SSHX040006677.g | 1.272630556 | HHX020009994.g | 0.050687111 |
| SSHX040006679.g | 1.011188556 | HHX020010026.g | 0.023976444 |
| SSHX040006705.g | 246.544237 | HHX140041984.g | 11.69084267 |
| SSHX040006712.g | 0.064318333 | HHX140041973.g | 1.933569778 |
| SSHX040006714.g | 0.021329444 | HHX140041972.g | 0.127213222 |
| SSHX040006732.g | 0.027477889 | HHX140041912.g | 0.444887889 |
| SSHX040006733.g | 0.010227889 | HHX140041914.g | 0.255569667 |
| SSHX040006758.g | 0.374444444 | HHX140041917.g | 0.045728222 |
| SSHX040006766.g | 0.165288111 | HHX140041929.g | 0.013142778 |
| SSHX040006767.g | 0.005233222 | HHX140041932.g | 0.307911667 |
| SSHX040006775.g | 0.057151222 | HHX140042019.g | 0.403717333 |
| SSHX040006776.g | 0.036634667 | HHX140041939.g | 0.219421222 |
| SSHX040006779.g | 0.008424778 | HHX140041949.g | 0.097167667 |
| SSHX040006783.g | 0.167160111 | HHX140042048.g | 5.525214111 |
| SSHX040006802.g | 0.000556667 | HHX140042037.g | 1.410113667 |
| SSHX040006835.g | 805.4151206 | HHX140042058.g | 2.485196111 |
| SSHX040006838.g | 0.052218778 | HHX140042059.g | 1.274718889 |
| SSHX040006839.g | 246.7957272 | HHX140042061.g | 1442.80976 |
| SSHX040006848.g | 0.108800889 | HHX140042072.g | 0.019434889 |
| SSHX040006864.g | 0.173456111 | HHX140042045.g | 1.247900778 |
| SSHX040006878.g | 0.001567333 | HHX140042086.g | 0.221327667 |
| SSHX040006879.g | 0.126806333 | HHX140042091.g | 0.005337444 |
| SSHX040006880.g | 13.42238167 | HHX140042092.g | 1.214046444 |
| SSHX040006887.g | 0.038358111 | HHX140042120.g | 0.594071444 |
| SSHX040006888.g | 0.001554889 | HHX140042121.g | 0.012433667 |
| SSHX040006889.g | 2.920223 | HHX030011869.g | 0.028396556 |
| SSHX040006896.g | 0.046998 | HHX010037216.g | 2.268348111 |
| SSHX040006897.g | 0.161386 | HHX030011868.g | 0.003585556 |
| SSHX040006901.g | 0.032949222 | HHX140042145.g | 0.255837556 |
| SSHX040006904.g | 0.012432778 | HHX010031937.g | 5.866742333 |
| SSHX040006905.g | 0.149842556 | HHX030011920.g | 21.22802467 |
| SSHX040006917.g | 0.090715778 | HHX030011920.g | 21.22802467 |
| SSHX040006919.g | 0.846874444 | HHX140042069.g | 0.010812667 |
| SSHX040006920.g | 0.667423222 | HHX010031926.g | 0.048744889 |
| SSHX040006921.g | 0.474992667 | HHX030011923.g | 0.023647778 |
| SSHX040006922.g | 0.466676556 | HHX140042093.g | 0.036560556 |
| SSHX040006923.g | 0.134234889 | HHX030011908.g | 1.068065 |
| SSHX040006929.g | 0.013422889 | HHX140042114.g | 0.877219444 |
| SSHX040006931.g | 0.071788889 | HHX010031885.g | 0.551201222 |
| SSHX040006932.g | 0.099411222 | HHX030011908.g | 1.068065 |
| SSHX040006935.g | 0.235655556 | HHX140042112.g | 2.832131889 |
| SSHX040006937.g | 0.979397778 | HHX030011945.g | 0.027783111 |
| SSHX040006938.g | 0.329356556 | HHX030011923.g | 0.023647778 |
| SSHX040006976.g | 0.385037333 | HHX140042152.g | 5.574943556 |
| SSHX040006988.g | 0.122515 | HHX140042155.g | 24.97044011 |
| SSHX040006990.g | 0.749094111 | HHX140042223.g | 0.01286 |
| SSHX040006998.g | 1.755686556 | HHX140042217.g | 0.067413111 |
| SSHX040007002.g | 0.035374222 | HHX140042166.g | 0.003224889 |
| SSHX040007010.g | 1.530358111 | HHX140042311.g | 0.039428889 |
| SSHX040007011.g | 0.073359778 | HHX140042299.g | 0.454145556 |
| SSHX040007012.g | 0.058276889 | HHX140042251.g | 0.008756889 |
| SSHX040007015.g | 0.615062556 | HHX140042252.g | 0.059702222 |
| SSHX040007016.g | 0.025673556 | HHX140042207.g | 0.002983889 |
| SSHX040007019.g | 0.464083111 | HHX140042382.g | 56.19850867 |
| SSHX040007020.g | 0.215737222 | HHX140042286.g | 0.015820444 |
| SSHX040007021.g | 0.519931 | HHX140042217.g | 0.067413111 |
| SSHX040007024.g | 0.167465333 | HHX140042235.g | 0.028154556 |
| SSHX040007025.g | 0.022693444 | HHX140042226.g | 0.879585333 |
| SSHX040007034.g | 0.046599444 | HHX140042279.g | 1.555292111 |
| SSHX040007035.g | 0.014223778 | HHX140042267.g | 0.324290444 |
| SSHX040007036.g | 0.036939333 | HHX140042193.g | 1.025446778 |
| SSHX040007039.g | 0.049859333 | HHX140042207.g | 0.002983889 |
| SSHX040007041.g | 0.240638667 | HHX140042249.g | 0.045838556 |
| SSHX040007042.g | 0.147154778 | HHX140042317.g | 0.001812778 |
| SSHX040007044.g | 0.025677778 | HHX140042267.g | 0.324290444 |
| SSHX040007045.g | 0.004331111 | HHX140042276.g | 0.416128444 |
| SSHX040007046.g | 0.096650889 | HHX140042370.g | 0.011135111 |
| SSHX040007051.g | 0.013735444 | HHX140042264.g | 0.486720444 |
| SSHX040007052.g | 0.005888667 | HHX140042203.g | 0.339692111 |
| SSHX040007057.g | 0.015505444 | HHX140042207.g | 0.002983889 |
| SSHX040007062.g | 0.665851333 | HHX140042286.g | 0.015820444 |
| SSHX040007063.g | 0.110734111 | HHX140042277.g | 0.676990111 |
| SSHX040007064.g | 0.006513889 | HHX140042290.g | 0.097004556 |
| SSHX040007068.g | 0.959716111 | HHX140042384.g | 8.678257778 |
| SSHX040007070.g | 0.17332 | HHX140042163.g | 0.016347111 |
| SSHX040007071.g | 0.155087111 | HHX140042223.g | 0.01286 |
| SSHX040007073.g | 0.005318 | HHX140042279.g | 1.555292111 |
| SSHX040007075.g | 0.033525444 | HHX140042213.g | 0.251358778 |
| SSHX040007076.g | 0.717505222 | HHX140042204.g | 0.140823667 |
| SSHX040007081.g | 0.001083778 | HHX140042226.g | 0.879585333 |
| SSHX040007082.g | 0.018464333 | HHX140042304.g | 3.483747889 |
| SSHX040007085.g | 0.029412778 | HHX140042365.g | 0.230359667 |
| SSHX040007087.g | 0.204481 | HHX140042175.g | 0.038980556 |
| SSHX040007091.g | 0.202723778 | HHX140042353.g | 1.040010889 |
| SSHX040007096.g | 0.205612889 | HHX140042380.g | 0.021795889 |
| SSHX040007102.g | 0.261749889 | HHX140042370.g | 0.011135111 |
| SSHX040007103.g | 0.052114 | HHX140042183.g | 0.008495889 |
| SSHX040007105.g | 0.056717333 | HHX140042207.g | 0.002983889 |
| SSHX040007110.g | 0.059896333 | HHX140042186.g | 0.803478778 |
| SSHX040007113.g | 0.040509222 | HHX140042207.g | 0.002983889 |
| SSHX040007120.g | 1.271796889 | HHX140042290.g | 0.097004556 |
| SSHX040007125.g | 0.241592778 | HHX140042219.g | 0.031819889 |
| SSHX040007126.g | 0.024549556 | HHX140042299.g | 0.454145556 |
| SSHX040007129.g | 0.155976444 | HHX140042223.g | 0.01286 |
| SSHX040007133.g | 0.008956667 | HHX140042362.g | 0.668069889 |
| SSHX040007134.g | 0.025575556 | HHX140042317.g | 0.001812778 |
| SSHX040007135.g | 0.692594222 | HHX140042282.g | 0.053072111 |
| SSHX040007136.g | 0.285589889 | HHX140042370.g | 0.011135111 |
| SSHX040007138.g | 0.248132778 | HHX140042423.g | 0.043444111 |
| SSHX040007150.g | 0.027061778 | HHX140042310.g | 0.176866667 |
| SSHX040007152.g | 0.005231444 | HHX140042195.g | 0.034155889 |
| SSHX040007154.g | 0.002728667 | HHX140042362.g | 0.668069889 |
| SSHX040007156.g | 1.288797111 | HHX140042177.g | 0.239043778 |
| SSHX040007159.g | 0.926193556 | HHX140042262.g | 0.065017 |
| SSHX040007165.g | 0.226607778 | HHX140042423.g | 0.043444111 |
| SSHX040007166.g | 0.988039556 | HHX140042286.g | 0.015820444 |
| SSHX040007169.g | 5.300690556 | HHX140042322.g | 0.836162556 |
| SSHX040007170.g | 1.965595889 | HHX140042269.g | 0.230351667 |
| SSHX040007174.g | 0.093964333 | HHX140042401.g | 0.680860222 |
| SSHX040007175.g | 0.091250889 | HHX140042383.g | 2.519490778 |
| SSHX040007179.g | 0.630869556 | HHX140042329.g | 0.067342333 |
| SSHX040007180.g | 0.289371111 | HHX140042183.g | 0.008495889 |
| SSHX040007181.g | 0.413846556 | HHX140042375.g | 0.017824778 |
| SSHX040007188.g | 0.021598556 | HHX140042279.g | 1.555292111 |
| SSHX040007191.g | 0.099065222 | HHX140042351.g | 0.793024222 |
| SSHX040007194.g | 0.036307667 | HHX140042226.g | 0.879585333 |
| SSHX040007196.g | 0.013209222 | HHX140042299.g | 0.454145556 |
| SSHX040007201.g | 0.526238333 | HHX140042330.g | 0.024104556 |
| SSHX040007203.g | 0.001335333 | HHX140042291.g | 0.033576778 |
| SSHX040007206.g | 0.100649111 | HHX140042286.g | 0.015820444 |
| SSHX040007217.g | 0.307568222 | HHX140042282.g | 0.053072111 |
| SSHX040007218.g | 0.010575889 | HHX140042371.g | 0.060787667 |
| SSHX040007219.g | 0.053600889 | HHX140042267.g | 0.324290444 |
| SSHX040007220.g | 0.046020889 | HHX140042224.g | 0.006456444 |
| SSHX040007221.g | 0.240456778 | HHX140042235.g | 0.028154556 |
| SSHX040007233.g | 0.000664 | HHX140042311.g | 0.039428889 |
| SSHX040007235.g | 0.021250333 | HHX140042236.g | 0.002283667 |
| SSHX040007236.g | 0.054381444 | HHX140042299.g | 0.454145556 |
| SSHX040007237.g | 27.00594544 | HHX140042282.g | 0.053072111 |
| SSHX040007239.g | 40.74496844 | HHX140042228.g | 0.948233444 |
| SSHX040007248.g | 0.086761778 | HHX140042392.g | 1.660047 |
| SSHX040007252.g | 0.274326 | HHX140042223.g | 0.01286 |
| SSHX040007260.g | 0.105160444 | HHX140042236.g | 0.002283667 |
| SSHX040007261.g | 0.009686667 | HHX140042371.g | 0.060787667 |
| SSHX040007262.g | 0.023153 | HHX140042351.g | 0.793024222 |
| SSHX040007267.g | 0.190702 | HHX140042359.g | 0.995938667 |
| SSHX040007269.g | 0.161731 | HHX140042361.g | 1.827657333 |
| SSHX040007270.g | 0.236672444 | HHX140042297.g | 0.013181667 |
| SSHX040007271.g | 0.139256667 | HHX140042224.g | 0.006456444 |
| SSHX040007277.g | 0.796201667 | HHX140042262.g | 0.065017 |
| SSHX040007283.g | 1.527713111 | HHX140042429.g | 0.136683667 |
| SSHX040007285.g | 0.030167111 | HHX140042383.g | 2.519490778 |
| SSHX040007286.g | 0.003364889 | HHX140042279.g | 1.555292111 |
| SSHX040007289.g | 0.002965444 | HHX140042371.g | 0.060787667 |
| SSHX040007291.g | 0.005654444 | HHX140042372.g | 0.068584778 |
| SSHX040007298.g | 0.329692333 | HHX140042316.g | 0.041908556 |
| SSHX040007302.g | 0.084208222 | HHX140042286.g | 0.015820444 |
| SSHX040007307.g | 0.025053444 | HHX140042382.g | 56.19850867 |
| SSHX040007308.g | 0.264198111 | HHX140042316.g | 0.041908556 |
| SSHX040007309.g | 0.228953111 | HHX140042286.g | 0.015820444 |
| SSHX040007315.g | 0.145330222 | HHX140042361.g | 1.827657333 |
| SSHX040007317.g | 0.054588556 | HHX140042176.g | 0.304179222 |
| SSHX040007318.g | 0.029126111 | HHX140042366.g | 0.208885111 |
| SSHX040007319.g | 0.644580333 | HHX140042375.g | 0.017824778 |
| SSHX040007320.g | 0.036798111 | HHX140042193.g | 1.025446778 |
| SSHX040007321.g | 0.175259444 | HHX140042380.g | 0.021795889 |
| SSHX040007327.g | 0.004363667 | HHX140042372.g | 0.068584778 |
| SSHX040007331.g | 0.009570222 | HHX140042264.g | 0.486720444 |
| SSHX040007334.g | 0.013794111 | HHX140042405.g | 0.415565444 |
| SSHX040007335.g | 0.000890333 | HHX140042330.g | 0.024104556 |
| SSHX040007339.g | 0.010491 | HHX140042362.g | 0.668069889 |
| SSHX040007341.g | 0.000404111 | HHX140042363.g | 0.183381 |
| SSHX040007348.g | 0.347683556 | HHX140042370.g | 0.011135111 |
| SSHX040007349.g | 0.014800556 | HHX140042276.g | 0.416128444 |
| SSHX040007351.g | 0.137089222 | HHX140042297.g | 0.013181667 |
| SSHX040007352.g | 0.094256222 | HHX140042317.g | 0.001812778 |
| SSHX040007359.g | 0.115502778 | HHX140042265.g | 0.634150556 |
| SSHX040007363.g | 0.041488889 | HHX140042236.g | 0.002283667 |
| SSHX040007367.g | 0.001936333 | HHX140042330.g | 0.024104556 |
| SSHX040007369.g | 0.003185556 | HHX140042335.g | 0.120001667 |
| SSHX040007370.g | 0.015118556 | HHX140042414.g | 0.083024222 |
| SSHX040007373.g | 0.016155556 | HHX140042382.g | 56.19850867 |
| SSHX040007392.g | 0.017308556 | HHX140042441.g | 0.003260222 |
| SSHX040007393.g | 0.096138 | HHX030012126.g | 0.007946444 |
| SSHX040007395.g | 0.003677667 | HHX030012147.g | 0.025834333 |
| SSHX040007409.g | 0.112204222 | HHX030012165.g | 0.863477222 |
| SSHX040007416.g | 0.005733667 | HHX030012174.g | 0.088776556 |
| SSHX040007417.g | 0.060673111 | HHX030012126.g | 0.007946444 |
| SSHX040007419.g | 0.343288556 | HHX140042473.g | 0.027297444 |
| SSHX040007421.g | 0.377123333 | HHX140042795.g | 0.000463889 |
| SSHX040007430.g | 0.088317444 | HHX140042463.g | 0.003678667 |
| SSHX040007431.g | 3.423670222 | HHX140042473.g | 0.027297444 |
| SSHX040007432.g | 0.057544444 | HHX140042792.g | 0.005907111 |
| SSHX040007442.g | 0.000856222 | HHX140042792.g | 0.005907111 |
| SSHX040007450.g | 0.026760889 | HHX030012163.g | 0.771323889 |
| SSHX040007471.g | 0.153557556 | HHX030012119.g | 0.009955889 |
| SSHX040007472.g | 1.214271333 | HHX030012109.g | 0.077959778 |
| SSHX040007476.g | 2.629467 | HHX030012126.g | 0.007946444 |
| SSHX040007478.g | 0.15911 | HHX140042712.g | 2.857503778 |
| SSHX040007503.g | 8.682112778 | HHX030012174.g | 0.088776556 |
| SSHX040007515.g | 0.010204111 | HHX030012182.g | 0.159744556 |
| SSHX040007516.g | 1.18889E-05 | HHX030012148.g | 0.14285 |
| SSHX040007518.g | 0.000722667 | HHX140042647.g | 0.021833556 |
| SSHX040007519.g | 1.184517222 | HHX030012126.g | 0.007946444 |
| SSHX040007520.g | 0.205485667 | HHX030012147.g | 0.025834333 |
| SSHX040007521.g | 0.163769444 | HHX030012198.g | 1.387594333 |
| SSHX040007539.g | 0.008688778 | HHX030012182.g | 0.159744556 |
| SSHX040007546.g | 1.488671111 | HHX030012188.g | 0.227208667 |
| SSHX040007550.g | 0.266139111 | HHX030012195.g | 0.051432111 |
| SSHX040007551.g | 0.050032667 | HHX030012198.g | 1.387594333 |
| SSHX040007561.g | 0.001218 | HHX030012165.g | 0.863477222 |
| SSHX040007562.g | 0.031450556 | HHX030012167.g | 0.551131778 |
| SSHX040007574.g | 0.031614556 | HHX140042236.g | 0.002283667 |
| SSHX040007579.g | 0.035433889 | HHX140042382.g | 56.19850867 |
| SSHX040007581.g | 0.198330111 | HHX140042236.g | 0.002283667 |
| SSHX040007590.g | 0.006085 | HHX140042423.g | 0.043444111 |
| SSHX040007593.g | 0.038035556 | HHX140042425.g | 0.412733667 |
| SSHX040007596.g | 0.524206 | HHX140042261.g | 0.029519222 |
| SSHX040007597.g | 0.050561222 | HHX140042426.g | 0.508777444 |
| SSHX040007602.g | 0.230397778 | HHX140042354.g | 2.029485667 |
| SSHX040007614.g | 0.028742889 | HHX030012105.g | 0.740941889 |
| SSHX040007615.g | 0.032074222 | HHX140042438.g | 0.465863 |
| SSHX040007616.g | 0.164692 | HHX140042504.g | 0.001533111 |
| SSHX040007617.g | 0.027863444 | HHX140042732.g | 0.162536111 |
| SSHX040007618.g | 0.418805 | HHX030012109.g | 0.077959778 |
| SSHX040007622.g | 0.033531444 | HHX030012158.g | 147.507788 |
| SSHX040007634.g | 0.013748 | HHX030012198.g | 1.387594333 |
| SSHX040007640.g | 0.139749444 | HHX140042463.g | 0.003678667 |
| SSHX040007641.g | 0.298719333 | HHX030012126.g | 0.007946444 |
| SSHX040007651.g | 0.000349222 | HHX030012182.g | 0.159744556 |
| SSHX040007654.g | 0.044517222 | HHX140042463.g | 0.003678667 |
| SSHX040007655.g | 0.009784333 | HHX030012189.g | 0.202274444 |
| SSHX040007656.g | 0.001709 | HHX140042499.g | 0.154569444 |
| SSHX040007657.g | 0.018928667 | HHX140042441.g | 0.003260222 |
| SSHX040007664.g | 0.485606778 | HHX140042511.g | 3.520282222 |
| SSHX040007665.g | 1.410578556 | HHX030012139.g | 0.093553556 |
| SSHX040007666.g | 0.048328111 | HHX140042636.g | 2.706988889 |
| SSHX040007670.g | 0.015676111 | HHX030012182.g | 0.159744556 |
| SSHX040007671.g | 0.031566 | HHX030012162.g | 9.538087111 |
| SSHX040007673.g | 0.039924778 | HHX140042795.g | 0.000463889 |
| SSHX040007674.g | 0.017697111 | HHX140042474.g | 0.228291444 |
| SSHX040007688.g | 0.021659778 | HHX140042540.g | 0.196952 |
| SSHX040007690.g | 0.013154778 | HHX140042795.g | 0.000463889 |
| SSHX040007697.g | 0.248297 | HHX030012206.g | 7.133784556 |
| SSHX040007704.g | 24.17346989 | HHX140042515.g | 0.271317667 |
| SSHX040007705.g | 0.735253222 | HHX030012148.g | 0.14285 |
| SSHX040007710.g | 0.048151333 | HHX140042563.g | 0.493605889 |
| SSHX040007711.g | 0.273681111 | HHX030012119.g | 0.009955889 |
| SSHX040007714.g | 0.231693556 | HHX140042557.g | 6.553135556 |
| SSHX040007716.g | 0.010810889 | HHX030012155.g | 0.132893333 |
| SSHX040007717.g | 0.046668889 | HHX140042463.g | 0.003678667 |
| SSHX040007719.g | 1.108230222 | HHX140042574.g | 0.003908667 |
| SSHX040007723.g | 0.071913556 | HHX140042585.g | 0.57818 |
| SSHX040007725.g | 0.77617 | HHX030012174.g | 0.088776556 |
| SSHX040007726.g | 1.903125333 | HHX140042643.g | 0.255305444 |
| SSHX040007727.g | 0.384222778 | HHX030012147.g | 0.025834333 |
| SSHX040007747.g | 0.482855778 | HHX030012109.g | 0.077959778 |
| SSHX040007750.g | 0.242595444 | HHX030012206.g | 7.133784556 |
| SSHX040007751.g | 0.983545667 | HHX030012119.g | 0.009955889 |
| SSHX040007752.g | 0.067019667 | HHX030012137.g | 0.000870889 |
| SSHX040007756.g | 0.935228111 | HHX030012147.g | 0.025834333 |
| SSHX040007757.g | 6.589173778 | HHX030012188.g | 0.227208667 |
| SSHX040007759.g | 0.071709556 | HHX030012154.g | 9.150593333 |
| SSHX040007768.g | 0.095204667 | HHX030012158.g | 147.507788 |
| SSHX040007769.g | 0.002982778 | HHX140042616.g | 0.578001444 |
| SSHX040007770.g | 0.023874889 | HHX030012198.g | 1.387594333 |
| SSHX040007777.g | 0.150410222 | HHX030012198.g | 1.387594333 |
| SSHX040007778.g | 1.986490778 | HHX030012158.g | 147.507788 |
| SSHX040007779.g | 0.026100778 | HHX030012162.g | 9.538087111 |
| SSHX040007780.g | 0.030679333 | HHX030012182.g | 0.159744556 |
| SSHX040007781.g | 1.112954333 | HHX140042571.g | 0.045905667 |
| SSHX040007790.g | 0.004310889 | HHX030012165.g | 0.863477222 |
| SSHX040007796.g | 9.141691333 | HHX030012174.g | 0.088776556 |
| SSHX040007819.g | 0.06188 | HHX140042767.g | 0.311756778 |
| SSHX040007821.g | 0.002775889 | HHX030012174.g | 0.088776556 |
| SSHX040007829.g | 1.874530222 | HHX140042668.g | 19.994122 |
| SSHX040007830.g | 0.153838444 | HHX030012137.g | 0.000870889 |
| SSHX040007831.g | 2.1268 | HHX140042749.g | 0.008132 |
| SSHX040007844.g | 1.390260333 | HHX030012182.g | 0.159744556 |
| SSHX040007845.g | 0.063349444 | HHX030012119.g | 0.009955889 |
| SSHX040007850.g | 0.079492333 | HHX030012130.g | 7.836096111 |
| SSHX040007855.g | 0.031244889 | HHX140042730.g | 1.791429778 |
| SSHX040007857.g | 0.070203222 | HHX030012119.g | 0.009955889 |
| SSHX040007867.g | 3.411402222 | HHX030012194.g | 0.179194889 |
| SSHX040007868.g | 0.044396778 | HHX140042795.g | 0.000463889 |
| SSHX040007873.g | 0.674431111 | HHX030012174.g | 0.088776556 |
| SSHX040007877.g | 1.603581 | HHX030012182.g | 0.159744556 |
| SSHX040007878.g | 0.208201333 | HHX140042795.g | 0.000463889 |
| SSHX040007881.g | 1.879403 | HHX030012188.g | 0.227208667 |
| SSHX040007882.g | 0.318177111 | HHX030012164.g | 0.051333333 |
| SSHX040007894.g | 0.126393444 | HHX140042795.g | 0.000463889 |
| SSHX040007901.g | 19.16092533 | HHX140042784.g | 1.328215222 |
| SSHX040007904.g | 10.768833 | HHX030012163.g | 0.771323889 |
| SSHX040007905.g | 0.944251444 | HHX030012182.g | 0.159744556 |
| SSHX040007907.g | 0.021824444 | HHX010031866.g | 0.151412444 |
| SSHX040007908.g | 0.014375333 | HHX030012224.g | 0.099913111 |
| SSHX040007909.g | 0.029843889 | HHX010031884.g | 0.749968 |
| SSHX040007911.g | 0.007575333 | HHX030012174.g | 0.088776556 |
| SSHX040007915.g | 0.998162 | HHX030012206.g | 7.133784556 |
| SSHX040007916.g | 0.133303333 | HHX140042795.g | 0.000463889 |
| SSHX040007919.g | 1.410236333 | HHX030012211.g | 0.127371111 |
| SSHX040007920.g | 3.100669222 | HHX010031899.g | 0.009416111 |
| SSHX040007935.g | 0.752139111 | HHX140042843.g | 3.866446556 |
| SSHX040007947.g | 0.043625667 | HHX010031908.g | 1.077088111 |
| SSHX040007958.g | 14.78643356 | HHX010031918.g | 0.317899222 |
| SSHX040007982.g | 5.935157444 | HHX140042899.g | 69.30698644 |
| SSHX040007985.g | 0.096384667 | HHX140042904.g | 0.001375 |
| SSHX040007995.g | 0.570770556 | HHX080003956.g | 0.093326 |
| SSHX040007998.g | 0.885282778 | HHX080003953.g | 0.117523556 |
| SSHX040008024.g | 0.063033778 | HHX140042926.g | 0.001019778 |
| SSHX040008025.g | 1.526247333 | HHX140042970.g | 16.680018 |
| SSHX040008026.g | 0.010925333 | HHX140042914.g | 0.790014111 |
| SSHX040008048.g | 0.251065111 | HHX140043038.g | 0.007985667 |
| SSHX040008058.g | 0.004740222 | HHX140043059.g | 0.095212 |
| SSHX040008059.g | 0.102086333 | HHX140043028.g | 0.016090333 |
| SSHX040008060.g | 6.464633333 | HHX140043033.g | 0.028403333 |
| SSHX040008068.g | 0.161302222 | HHX140043033.g | 0.028403333 |
| SSHX040008074.g | 0.006916333 | HHX140043061.g | 0.218623667 |
| SSHX040008078.g | 0.004414556 | HHX140043062.g | 0.686072778 |
| SSHX040008101.g | 0.020733111 | HHX140043077.g | 0.002641222 |
| SSHX040008148.g | 1.112402889 | HHX140043145.g | 27.13544144 |
| SSHX040008152.g | 0.341956333 | HHX140043177.g | 0.048192222 |
| SSHX040008153.g | 0.375651778 | HHX140043162.g | 0.022854889 |
| SSHX040008158.g | 3.621608 | HHX140043147.g | 0.584396444 |
| SSHX040008173.g | 0.006640556 | HHX140043177.g | 0.048192222 |
| SSHX040008174.g | 0.014632111 | HHX140043194.g | 0.208564222 |
| SSHX040008183.g | 0.180378333 | HHX140043162.g | 0.022854889 |
| SSHX040008184.g | 0.095271333 | HHX140043201.g | 0.003542 |
| SSHX040008205.g | 202.8933877 | HHX140043202.g | 3.542098111 |
| SSHX040008212.g | 0.120451778 | HHX140043201.g | 0.003542 |
| SSHX040008233.g | 0.163797778 | HHX030012388.g | 1.407892778 |
| SSHX040008234.g | 0.363580778 | HHX030012389.g | 2.325471556 |
| SSHX040008235.g | 0.111627 | HHX140043511.g | 0.019969778 |
| SSHX040008237.g | 0.220422 | HHX140043571.g | 2.978612333 |
| SSHX040008238.g | 0.327725 | HHX140043498.g | 2.746840556 |
| SSHX040008248.g | 0.158519111 | HHX140043383.g | 0.000439889 |
| SSHX040008256.g | 0.357016 | HHX140043521.g | 0.010437444 |
| SSHX040008257.g | 4.275304222 | HHX030012430.g | 0.018922778 |
| SSHX040008260.g | 80.159794 | HHX140043233.g | 0.051421222 |
| SSHX040008261.g | 4.590760222 | HHX140043273.g | 0.059970333 |
| SSHX040008265.g | 77.22637156 | HHX030012445.g | 1.349929778 |
| SSHX040008278.g | 0.052067111 | HHX140043299.g | 0.005711 |
| SSHX040008284.g | 0.139073556 | HHX140043424.g | 0.017878889 |
| SSHX040008296.g | 18.04142333 | HHX140043273.g | 0.059970333 |
| SSHX040008297.g | 1.885308889 | HHX140043377.g | 0.157696 |
| SSHX040008305.g | 9.236041333 | HHX140043557.g | 0.678685 |
| SSHX040008306.g | 17.322916 | HHX140043367.g | 3.249906444 |
| SSHX040008307.g | 12.33742944 | HHX140043290.g | 1.430866556 |
| SSHX040008308.g | 0.835891667 | HHX140043383.g | 0.000439889 |
| SSHX040008309.g | 6.852915556 | HHX030012459.g | 0.544613333 |
| SSHX040008310.g | 3.409277333 | HHX140043405.g | 0.024196778 |
| SSHX040008315.g | 0.081874111 | HHX140043295.g | 0.695940333 |
| SSHX040008316.g | 0.031243444 | HHX030012458.g | 3.995523333 |
| SSHX040008318.g | 0.019036333 | HHX030012457.g | 6.304078556 |
| SSHX040008319.g | 0.022649 | HHX140043383.g | 0.000439889 |
| SSHX040008324.g | 0.007797556 | HHX140043307.g | 0.055326444 |
| SSHX040008326.g | 0.064280444 | HHX140043435.g | 0.983613889 |
| SSHX040008341.g | 0.147821444 | HHX140043435.g | 0.983613889 |
| SSHX040008346.g | 0.256485 | HHX140043376.g | 0.020466 |
| SSHX040008347.g | 0.111021222 | HHX030012447.g | 1.065271889 |
| SSHX040008351.g | 0.045191111 | HHX140043077.g | 0.002641222 |
| SSHX040008356.g | 0.000458111 | HHX140043163.g | 0.049631444 |
| SSHX040008360.g | 3.393174 | HHX030012422.g | 0.083922667 |
| SSHX040008364.g | 0.037638556 | HHX140043077.g | 0.002641222 |
| SSHX040008379.g | 0.076997556 | HHX140043062.g | 0.686072778 |
| SSHX040008380.g | 1.070824 | HHX140043028.g | 0.016090333 |
| SSHX040008381.g | 0.015229444 | HHX140043056.g | 1.656040444 |
| SSHX040008395.g | 0.021726111 | HHX140043056.g | 1.656040444 |
| SSHX040008415.g | 1.245772778 | HHX140043077.g | 0.002641222 |
| SSHX040008428.g | 0.026807667 | HHX140042995.g | 0.695843222 |
| SSHX040008429.g | 0.006586333 | HHX140043013.g | 0.090658222 |
| SSHX040008434.g | 17.73247267 | HHX140042900.g | 0.017703 |
| SSHX050033093.g | 0.451668444 | HHX080006618.g | 3.857199333 |
| SSHX050033099.g | 0.363909333 | HHX080006623.g | 2.163665111 |
| SSHX050033114.g | 0.371948222 | HHX010031768.g | 2.566239444 |
| SSHX050033128.g | 0.278161333 | HHX090024958.g | 56.71878944 |
[truncated: 832,365 more chars]
